# Phylogenetic analysis of the cytochrome P450 (CYP450) nucleotide sequences of the horse and predicted CYP450s of the white rhinoceros (*Ceratotherium simum*) and other mammalian species

**DOI:** 10.7717/peerj.5718

**Published:** 2018-10-09

**Authors:** Marion Leiberich, Hendrik Johannes Marais, Vinny Naidoo

**Affiliations:** 1 Department of Companion Animal Clinical Studies, Faculty of Veterinary Science, University of Pretoria, Pretoria, South Africa; 2 Saving the Survivors, Pretoria, South Africa; 3 Department of Paraclinical Science, Faculty of Veterinary Science, University of Pretoria, Pretoria, South Africa

**Keywords:** Drug metabolism, White rhinoceros, Horse, Cytochrome P450, Phylogenetics

## Abstract

**Background:**

The plight of the white rhinoceros (*Ceratotherium simum*) and the increasing need of treatment options for injured poaching victims led to the necessity to expand the knowledge on applicable drugs in this endangered species. With very little information available on drug pharmacokinetics in rhino, veterinarians have to rely on information generated from other species. The horse being a closely related species, has served as the model for dose extrapolations. However, from recent research on enrofloxacin and carprofen, the white rhino showed considerable differences in the pharmacokinetic properties of these drugs in comparison to the horse. While the reason for the differences is unknown, a likely cause may be a difference in present cytochrome P450 (CYP450), which may result in the rhino being genetically deficient in certain enzyme families.

**Methods:**

For this paper we assess the degree of similarity of the CYP genome sequences across the different species, using BLAT (BLAST-like alignment tool) for the alignment of the nucleotide sequences of the equine CYP450 with potential homologous nucleotide sequences of the published database from white rhinos and other mammalian species (cow, pig, dog, sheep, elephant, mouse and human).

**Results:**

The white rhino nucleotide sequences were 90.74% identical to the equine sequences. This was higher than the degree of similarity between any of the other evaluated species sequences. While no specific CYP family were found to be deficient in the published rhino genome, the horse genome contained additional genetic sequence for a larger number of iso-enzymes that were not present in the rhino.

**Discussion:**

In pharmacokinetic study, it is well known that absence of a metabolic enzyme will result in constraints in drug metabolism and drug elimination. While this was our speculation, comparison to the horse and other mammalian species indicate that all the described CYP genes required for metabolism are present within the rhino genome. These results leave functional differences in enzyme activity and a lack of isoenzymes as the likely reason for the constraint in drug metabolism. Despite a more than 90% similarity of the equine and rhino gene sequences, seemingly small differences can have major effects on drug metabolism. Thus, in spite of the close anatomical relationship, the rhino should not simply be treated like a big horse.

## Introduction

The brutal poaching crisis which led to the killing of thousands of rhinos over the last few years (Poaching Statistics, 2018; Emslie et al., 2016) has drawn special attention to the plight of this threatened species. With the increasing number of rhinos requiring urgent medical help, the need to optimise the treatment of severely injured poaching victims is substantial. Unfortunately, with the need of appropriate drugs being a relatively recent problem, scientifically evaluated data for the treatment of rhino is lacking. Furthermore, research in wildlife medicine is often complicated by the animals’ size and untamed nature, by the lack of sufficient numbers of study animals, and is often challenging due to their status as endangered species.

As a result, wildlife veterinarians have resorted to using the horse (*Equus caballus*) as a model for the treatment of white rhino (*Ceratotherium simum*). While from a general phylogenetic point of view the horse is regarded as one of the closest related species to the rhinoceros (Tougard et al., 2001), we questioned whether it is correct to treat rhinos like large horses. Studies (Leiberich et al., 2018; M. Leiberich, 2018, unpublished data) have recently been undertaken in order to elucidate this issue. Enrofloxacin and carprofen, two potential drugs for the antimicrobial and analgesic treatment of poaching victims and other injured white rhinos, were evaluated in plasma pharmacokinetic studies. These studies revealed significant differences between drug pharmacokinetic parameters in the white rhino and the horse. Carprofen was characterised by a half-life of elimination of 105.71 ± 15.67 h, which is by far the longest recorded for any animal species (Leiberich et al., 2018). Furthermore, interspecies allometric scaling of enrofloxacin was able to show that the difference in the half-life of enrofloxacin in white rhino was not only due to the relative differences in their body size and that the half-life was not allometrically scalable (M. Leiberich, 2018, unpublished data). Overall, these studies suggest that the white rhino is a slow metaboliser of some drugs and that the rhino is not simply comparable with the horse. While speculative, it was believed that the interspecies differences result at least partially from differences in drug metabolising enzymes and possibly from a lack of an important drug metabolising enzyme family. However, very little is known about the metabolic capacity of different animal species and about the causes of variation in drug metabolism. Consequently, the assessment of the drug metabolising units is needed.

The most important drug metabolising enzymes are the cytochrome P450s (CYP450), a diverse family of heme containing monooxygenases (Ioannides, 2006; Toutain, Ferran & Bousquet-Mélou, 2010), which play a major role in phase I reactions (Smith, 2009). The discovery of CYP450 enzymes dates back to the 1950s, when Klingenberg first described the carbon monoxide binding pigment with its absorbance maximum around 450 nm (Estabrook, 2003; Klingenberg, 1958). Already in the 1960s, the CYP450s were known to be linked to the drug and steroid metabolism (Nebert & Russell, 2002). Nowadays, the diverse functions have been further elucidated and range from the synthesis of steroid hormones (Payne & Hales, 2004) and endogenous epithelial relaxation factor (Fisslthaler et al., 1999) to the metabolism of xenobiotics (Anzenbacher & Anzenbacherova, 2001). For classification purposes, the CYP450 enzymes have been divided into families sharing a primary structure, which is at least 40% identical. The classification into subfamilies, characterised by letters, is based on a more than 55% identical primary structure (Van Der Weide & Hinrichs, 2006). The individual isoenzymes differ by a minimum of 3% and are characterised by a second arabic number at the end (Anzenbacher & Anzenbacherova, 2001; Nelson, 2006). The major CYP450 families involved in drug metabolism are the CYP1, CYP2 and CYP3 families which account for more than 90% of the drug oxidation in humans (Ioannides, 2006; Zanger & Schwab, 2013). While cytochrome P450 enzymes have been widely studied in humans, information for animal species is scarce (Fink-Gremmels, 2008). Despite the interest in adequate animal models for human drug development and the interest in the prediction of drug residue levels in production animal species, the scientific knowledge in this field is still in its infancy (Fink-Gremmels, 2008; Ioannides, 2006; Martignoni, Groothuis & De Kanter, 2006).

The aim of this study was to assess the degree of in silico similarity between the CYP enzyme sequences published for the horse and other mammalian species with the rhino. The main objective was to ascertain if the rhino could be genetically deficient in any particular CYP enzyme families, which would provide insight for observed prominent differences in drug metabolism. The phylogenetic relationship of the CYP enzymes of the horse and the gene sequence of selected species including the white rhino, the cow (*Bos taurus*), the dog (*Canis lupus familiaris*), the pig (*Sus scrofa*), the elephant (*Loxodanta africana*), the sheep (*Ovis aries*), the mouse (*Mus musculus*) and the human (*Homo sapiens*) was assessed.

## Materials and Methods

A data mining strategy was applied to match the cytochrome P450 gene sequences of the horse to the gene sequences of selected species (Table 1) in order to determine the existence and the degree of homologous sequences amongst the different species. Fifteen identified CYP genes of the horse, namely CYP11A1, CYP17A1, CYP19A1, CYP27B1, CYP2A13, CYP2C113, CYP2C92, CYP2D50, CYP2E1, CYP3A89, CYP3A93, CYP3A94, CYP3A95, CYP3A96 and CYP3A97 (Wade et al., 2009) were used to perform a BLAT (BLAST like alignment tool) (Kent, 2002) search against the NCBI genome assemblies for the selected species (Archibald et al., 2010a, 2010b; Jiang et al., 2014; Lindblad-Toh et al., 2005; Uenishi et al., 2012; Zimin et al., 2009). Sequences with less than 15% alignment were taken out automatically. Using the Molecular Evolutionary Genetics Analysis (MEGA) (Kumar, Stecher & Tamura, 2016), the evolutionary relationship between the CYP450 genes of the horse and the corresponding gene sequences of the other species were inferred. Furthermore, a phylogenetic tree was constructed depicting the evolutionary relationship between the cytochrome P450 enzymes of the horse and the matching gene sequences of the selected species.

**Table 1:** Selected species, corresponding accession ID numbers and additional sample information included in the comparison to the gene sequences of CYP enzymes.

Species	Accession ID	Additional sample information
Horse (*Equus caballus*)	GCA_000002305.1	Female, thoroughbred, isolate Twilight
White rhinoceros (*Ceratotherium simum*)	GCA_000283155.1	Female
Cow (*Bos taurus*)	GCA_000003055.4	Pooled male and female samples, Hereford, tissue blood
Dog (*Canis lupus familiaris*)	GCA_000002285.2	Female Boxer
Pig (*Sus scrofa domesticus*)	GCA_000003025.4	Female, Duroc, isolate TL Tabasco
Elephant (*Loxodonta africana*)	GCA_000001905.1	Female
Sheep (*Ovis aries*)	GCA_000298735.1	Male and female, Texel
Mouse (*Mus musculus*)	GCA_000001305.2	Strain C57BL/6J
Human (*Homo sapiens*)	GCA_000001305.2	Genome Reference Consortium Human GRCh38

A detailed description on how to build a phylogenetic tree from molecular data with MEGA is given by Hall (2013). Briefly, the sequence alignment was performed using Multiple Sequence Comparison by Log Expectation (Edgar, 2004). Subsequently, different substitution models were assessed for the goodness of fit measured by the Bayesian information criterion (BIC) (Schwarz, 1978). Based on the lowest BIC value, the Kimura 2-parameter model (Kimura, 1980) (CYP2E1, CYP3A89, CYP3A96) and the Tamura 3-parameter model (Tamura, 1992) (CYP11A1, CYP17A1, CYP19A1, CYP27B1, CYP2A13, CYP2C113, CYP2C92, CYP2D50, CYP3A93, CYP3A94, CYP3A95, CYP3A97) were chosen to assess the evolutionary distance based on the Maximum Likelihood method. The initial trees for the heuristic search were constructed automatically. Therefore, the Neighbor-Join and BioNJ algorithms were applied to a matrix of pairwise distances, which were estimated using a maximum composite likelihood approach. The topology with the best log likelihood value was chosen. Additionally, in order to model the evolutionary rate differences amongst sites (five categories), a discrete gamma distribution was applied (Yang, 1994). Codon positions included were 1st + 2nd + 3rd + Noncoding. Positions with less than 95% site coverage were eliminated.

The bootstrap consensus tree based on 1,000 replicates (Felsenstein, 1985) was built in order to assess the reliability of a phylogenetic tree. The percentage of the recovery of the same nodes throughout the bootstrap analysis is indicated next to the branches. The analysis was based on nine nucleotide sequences from the different species. Another bootstrap consensus tree (1,000 replicates) of all equine CYP450s and the matching gene sequences of the chosen species was computed and displayed in the circular view. The estimation of the evolutionary tree was based on the Maximum Likelihood method. It includes 135 nucleotide sequences from nine different species and was constructed using the Tamura-3-parameter model. Branches corresponding to partitions reproduced in less than 50% bootstrap replicates were collapsed. The initial trees for the heuristic search were constructed automatically. Therefore, the Neighbor-Join and BioNJ algorithms were applied to a matrix of pairwise distances, which were estimated using a maximum composite likelihood approach. The topology with the best log likelihood value was chosen. In order to model the evolutionary rate differences amongst sites (five categories, parameter = 1.8250), a discrete gamma distribution was applied (Yang, 1994). The rate variation model allowed for some sites to be evolutionary invariable (2.6181% sites). Codon positions included were 1st + 2nd + 3rd + Noncoding. All positions with less than 95% site coverage were eliminated.

Furthermore, the evolutionary distance between the nucleotide sequences of the equine CYP3A enzymes and the matching gene sequences of the white rhino and of the other species were computed using the pairwise distance function in MEGA (Kumar, Stecher & Tamura, 2016). The nucleotide differences between each pair of sequences were calculated to facilitate the assessment of the degree of similarity among the sequences.

## Results

**Figure 1:**
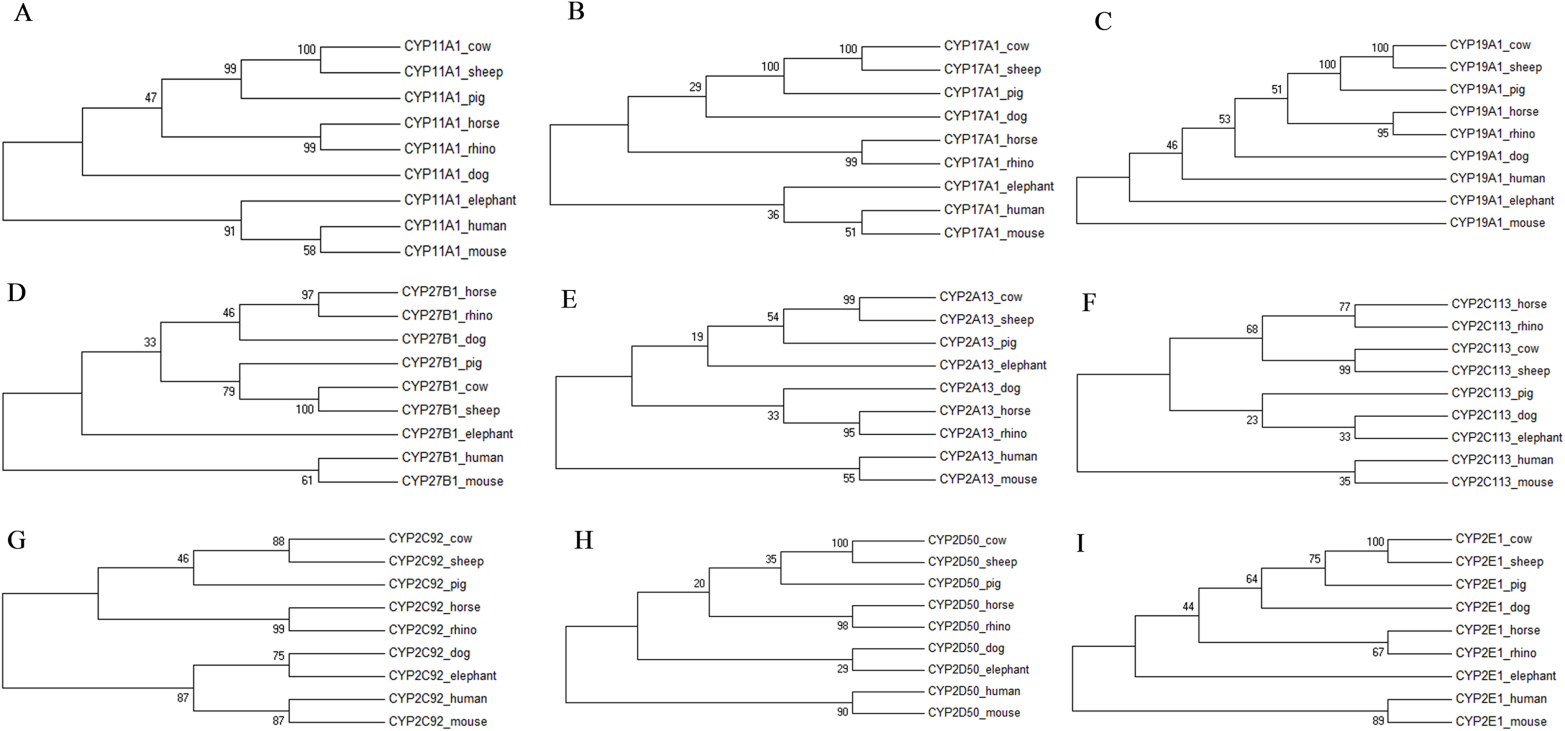
Molecular phylogenetic relationship of CYP11A1 (A), CYP17A1 (B), CYP19A1 (C), CYP27B1 (D), CYP2A13 (E), CYP2C113 (F), CYP2C92 (G), CYP2D50 (H) and CYP2E1 (I) across nine different species.

**Figure 2:**
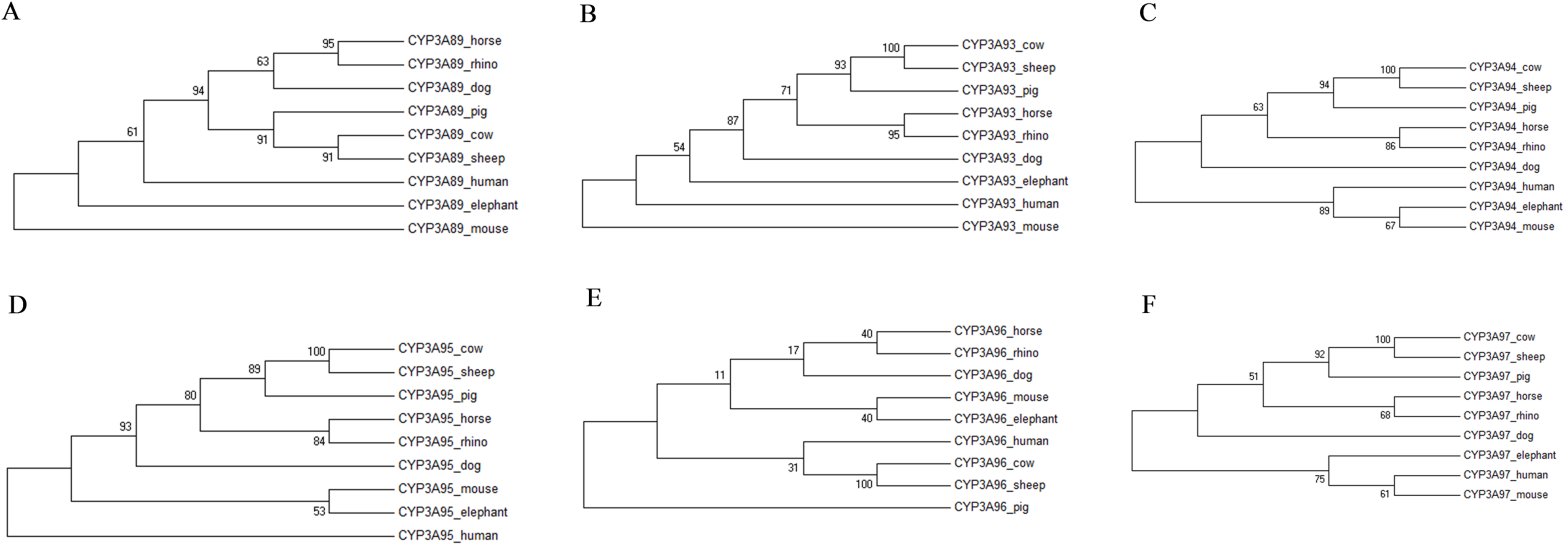
Molecular phylogenetic relationship of the CYP3A89 (A), CYP3A93 (B), CYP3A94 (C), CYP3A95 (D), CYP3A96 (E) and CYP3A97 (F) across nine different species by Maximum Likelihood (ML) method.

**Figure 3:**
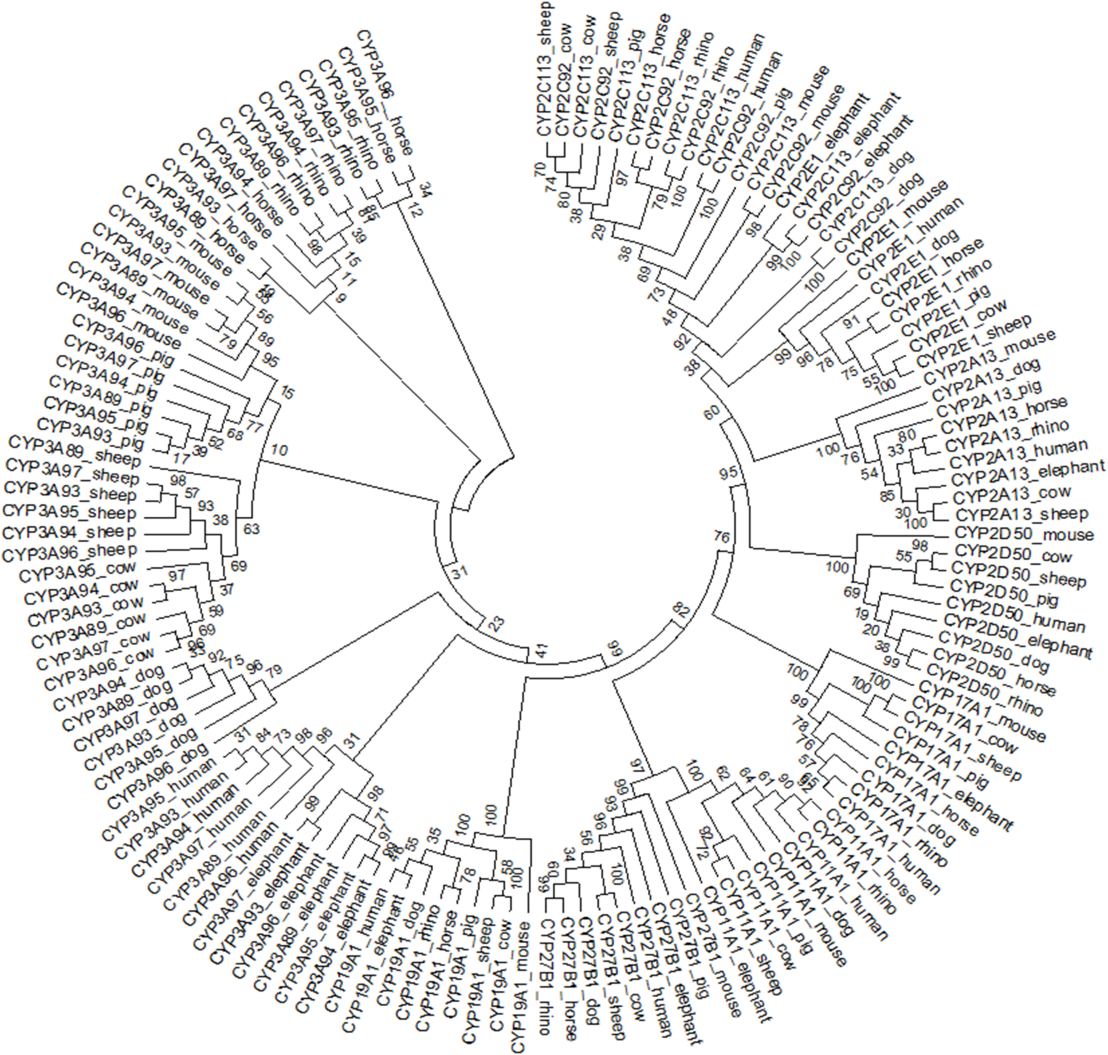
Circular phylogenetic tree depicting the relationship of 135 nucleotide sequences of all CYP450 genes of the horse and the matched nucleotide sequences of eight other species (also named CYP450).

The individual phylogenetic trees for each P450 enzyme detected in the horse (CYP11A1, CYP17A1, CYP19A1, CYP27B1, CYP2A13, CYP2C113, CYP2C92, CYP2D50, CYP2E1, CYP3A89, CYP3A93, CYP3A94, CYP3A95, CYP3A96 and CYP3A97) are depicted in Figs. 1 and 2. The phylogenetic trees highlight the evolutionary relationship of the equine gene sequences and the gene sequences of the white rhino, the cow, the dog, the pig, the elephant, the sheep, the mouse and the human. In all cases, the alignment of the CYP genes of the horse with the gene sequences of the other species showed that the horse CYP enzymes are most closely related to the sequences of the white rhino. The degree of similarity between the known equine CYP450 genes and the sequences identified in the genome of the white rhino ranged from 87.8% to 94.1% (Table 2). On average, the white rhino nucleotide sequences were 90.74% identical to the equine CYP450 gene sequences. Figure 3 illustrates the relationship between all CYP450 genes of the horse and the matching nucleotide sequences of the other species (also named CYP450s in the phylogenetic trees). Gene sequences of all CYP enzyme families identified in the horse seem to be present in the white rhino.

**Table 2:** Degree of similarity (%) between the equine CYP450 genes and the genome sequences of the white rhinoceros.

CYP450 of the horse	Similarity (%) to the genome sequences of the white rhinoceros and the horse
CYP11A1 (NM_001082521)	89.8
CYP17A1 (NM_001082523)	91
CYP19A1 (NM_001081805)	89
CYP27B1 (NM_001163957)	94.1
CYP2A13 (NM_001111337)	91.3
CYP2C113 (NM_001291302)	90.7
CYP2C92 (NM_001101652)	87.8
CYP2D50 (NM_001111306)	92.4
CYP2E1 (NM_001111303)	91.2
CYP3A89 (NM_001101651)	90.6
CYP3A93 (NM_001190938)	91.7
CYP3A94 (NM_001190939)	93
CYP3A95 (NM_001190940)	90.5
CYP3A96 (NM_001146163)	89.2
CYP3A97 (NM_001146164)	88.8

The calculation of the pairwise distance between the nucleotide sequences of the rhino (Table 3) revealed that the CYP3A gene sequences were identical or highly similar with distance indexes of as little as 0.023 between the CYP3A95 and the CYP3A93 nucleotide sequence and 0 between the CYP3A96 and CYP3A89 nucleotide sequences. Further investigations showed that the sequences matching all the equine CYP3A genes were found at the same location in the genome of the rhino and overlapped each other in most cases. Additionally, the calculation of the pairwise distance of the CYP3A nucleotide sequences of the cow, dog, pig, human and sheep matched to the equine CYP3A sequences demonstrate a very high degree of similarity of up to 100% identity amongst each other (S1).

**Table 3:** Estimates of evolutionary divergence between the nucleotide sequences of the white rhino, which matched the equine CYP3A sequences.

	CYP3A89_rhino	CYP3A93_rhino	CYP3A94_rhino	CYP3A95_rhino	CYP3A96_rhino	CYP3A97_rhino
CYP3A89_rhino						
CYP3A93_rhino	0.066					
CYP3A94_rhino	0.056	0.059				
CYP3A95_rhino	0.059	0.023	0.036			
CYP3A96_rhino	0.000	0.066	0.056	0.059		
CYP3A97_rhino	0.056	0.056	0.043	0.046	0.056	

**Notes:**

Presented as numbers of base differences per site between each pair of sequences. Codon positions included were 1st + 2nd + 3rd + Noncoding. Evolutionary analyses were conducted in MEGA (Kumar, Stecher & Tamura, 2016).

## Discussion

While large numbers of different species, diseases and conditions require medical attention and treatment, wildlife veterinarians are often faced with the lack of approved and scientifically evaluated drugs for zoo and wildlife species. A study published by Tana et al. (2010) stated that only 8–10 compounds are approved for the use in zoo and wildlife in the USA in contrast to close to 300 in cattle. The white rhino represents one of the species where basic medical knowledge is not yet readily available. However, the poaching crisis and the increasing numbers of injured individuals requiring urgent medical help has been on the rise, especially for the treatment of wounds. To overcome limitations in species specific information, the medical management of the rhino tends to be based on information available for the horse. However, the findings of a recent study we undertook in rhinos showed that the half-life of elimination for carprofen was more than threefold longer than that in the horse (Leiberich et al., 2018). This made us question the validity of the horse as a model.

The differences in drug metabolism could generally arise from distinctions in anatomy, physiology, behaviour, biotransformation and metabolism by CYP450 enzymes. While these potential influencing factors can all be considered, we believe that with the horse and the rhino showing a closely related digestive physiology and anatomy of the gastrointestinal tract and similar feeding habits, the differences in drug metabolism would most likely be ascribed to distinctions in their CYP450 enzymes. To assess this assumption, the gene sequences of the CYP450s of the horse, the white rhino and other selected species were compared as a first step in ascertaining if any of the genes coding for the major drug metabolising families were absent in the rhino genome. From the gene analysis it would appear that the complete genetic deletion of a major CYP enzyme family was not the cause of the evident limitations in drug metabolism, leaving functional differences in enzymes activity as the likely reason. The latter was evident in the subsequent relatedness analysis. While showing the horse to be the most closely related species, only 90.74% similarity were evident across all the equine CYP enzymes. This finding would indicate that the genetic differences between the horse and the rhino are sufficient to result in major differences in drug metabolism. This would render drug prediction between the species unreliable at the clinical level.

Similar conclusions have been previously drawn by Fink-Gremmels (2008), Martignoni, Groothuis & De Kanter (2006), Nelson, Goldstone & Stegeman (2013) and Toutain, Ferran & Bousquet-Mélou (2010) who declared that small differences in the amino acid sequence of the CYP enzyme can lead to marked changes in substrate specificity and catalytic activity. Thus, not even closely related species with similar physiological characteristics exhibit similar cytochrome P450 enzyme activity. Even a single change in amino acid sequences is sufficient to possibly alter substrate specificity (Lindberg & Negishi, 1989) and different CYP450 enzymes may metabolise the same substrate (Guengerich, 1997). In more serious cases, the genetic difference could result in stop codons being present in the incorrect area within the mRNA sequence with abnormal termination of the enzyme translation (McAdam, Goundis & Reid, 1988). Contrary to general expectations, the enzyme liver pattern of herbivore and carnivore species, of monogastric species and ruminants and even within a species such as cattle, differs markedly (Fink-Gremmels, 2008). Guengerich (1997) suggested a provisional classification according to ‘catalytic preservation’ of the CYP450 enzymes. Accordingly, the only CYP450 enzyme, which can be accurately extrapolated across species is the CYP2E1. The extrapolation of the CYP1A1, 1A2 and 17A enzymes needs to be conducted carefully. Even more caution is required for the extrapolation of CYP2D and 3A, whereas the extrapolation of CYP2A, 2B and 2C enzymes across species shows no catalytic preservation.

Another important finding in this study relates to the CYP3A enzyme family. In humans, it represents about 30% of the total liver CYP content and metabolises around 50% of all marketed drugs. Its most important drug metaboling isoenzyme is the CYP3A4 (Furge & Guengerich, 2006). While detailed information on the importance of the different isoenzymes and their contribution to drug metabolism in animals is not yet available, one would assume that the important drug metabolising CYP families are the same as in humans. The importance of these enzymes is further evident by the number of isoenzymes within the group. Four have been identified in man (CYP3A4, 3A5, 3A7 and 3A43) and six in the horse (CYP3A89, CYP3A93, CYP3A94, CYP3A95, CYP3A96, CYP3A97), with the equine CYP3A89 exhibiting the highest similarity to the human CYP3A4 (Schmitz et al., 2010).

The pairwise distance analysis between the CYP3A nucleotide sequences of the white rhino revealed high levels of similarity (Table 3). Additionally, the nucleotide sequences were all limited to the same location (JH767858: 353657–1001765) in the genome of the rhino. This finding suggests that the CYP3A family in the rhino genome has no isoenzymes, and consequently, that the white rhino has fewer isoenzymes than the horse. Overall, with the CYP3A subfamily being of major importance for the drug metabolism, a lack of isoenzymes may explain the observed constraint in white rhino. However, similar observations were made based on the pairwise comparison of the CYP3A gene sequences in the other species. In most cases, the sequences of the pig, cow, dog, human and sheep, which were matched to the different CYP3A gene sequences of the horse did not reveal any sequence difference and thus seemed to represent one single nucleotide sequence as in the rhino (S1). Alternatively, those findings may imply that the white rhino, like other species may have its own, species specific set of drug metabolising CYP3A isoenzymes, which differ from those in the horse. Rather than reflecting a true non-existence of CYP3A isoenzymes, those may have just not yet been identified in the rhino.

Similar to the rhino, drug doses for elephants, another species belonging to the hindgut fermenters, are often extrapolated from pharmacokinetic data available for horses (Hunter, Isaza & Koch, 2003; Mortenson, 2001). However, the comparison of the CYP3A gene sequences also showed that unlike in the rhino, the elephants’ CYP3A gene sequences seem to be the closest to those of the mouse and not closest related to the horse nor to the white rhino (Fig. 2). This finding may further indicate that drug dose extrapolation from horses to other hindgut fermenters and mega-herbivores such as the elephant needs to be conducted with caution and cannot be based solely on the fact that they share similar physiological characteristics.

## Conclusion

In conclusion, the rhino as a species was not overly deficient for any of the genes coding for the major metabolizing enzymes. While the white rhino CYP450 gene sequences were most similar to those of the horse, this was overall only at the 90% level. Despite appearing to be a minor distinction, even smaller differences are known to have a major effect on drug metabolism. As a result, despite the close anatomical relationship, the rhino should not simply be treated like a big horse.

## Supplemental Information

10.7717/peerj.5718/supp-1 Supplemental Information 1 Estimates of evolutionary divergence between the CYP3A nucleotide sequences of the cow (A), dog (B), pig (C), human (D), sheep (E), which were matched to the equine CYP3A sequences. Presented as numbers of base differences per site between each pair of sequences. Codon positions included were 1st+2nd+3rd+Noncoding. Evolutionary analyses were conducted in MEGA (Kumar, Stecher & Tamura, 2016).

10.7717/peerj.5718/supp-2 Supplemental Information 2 Nucleotide sequence of all CYP enzymes of the horse (*Equus caballus*) and the matching nucleotide sequences from the genomes of 8 other species.

## Additional Information and Declarations

### Data Availability

The following information was supplied regarding data availability: The raw data are provided in a Supplemental File.
