# Supplementary material for: Phylogenetic analysis of the cytochrome P450 (CYP450) nucleotide sequences of the horse and predicted CYP450s of the white rhinoceros (Ceratotherium simum) and other mammalian species
Source: PeerJ. 2018 Oct 9;6:e5718. doi: 10.7717/peerj.5718 (PMC6183514; doi:10.7717/peerj.5718)
Supplement: Supplemental Information 1 — Presented as numbers of base differences per site between each pair of sequences. Codon positions included were 1st+2nd+3rd+Noncoding. Evolutionary analyses were conducted in MEGA (Kumar, Stecher & Tamura, 2016). [file peerj-06-5718-s001.docx]

**Supplementary information**

**Estimates of evolutionary divergence between the CYP3A nucleotide sequences of the cow (A), dog (B), pig (C), human (D), sheep (E), which were matched to the equine CYP3A sequences.** Presented as numbers of base differences per site between each pair of sequences. Codon positions included were 1st+2nd+3rd+Noncoding. Evolutionary analyses were conducted in MEGA (Kumar, Stecher & Tamura, 2016).

A

|  | CYP3A89 | CYP3A93 | CYP3A94 | CYP3A95 | CYP3A96 | CYP3A97 |
| --- | --- | --- | --- | --- | --- | --- |
| CYP3A89 |  |  |  |  |  |  |
| CYP3A93 | 0.000 |  |  |  |  |  |
| CYP3A94 | 0.000 | 0.000 |  |  |  |  |
| CYP3A95 | 0.000 | 0.000 | 0.000 |  |  |  |
| CYP3A96 | 0.000 | 0.000 | 0.000 | 0.000 |  |  |
| CYP3A97 | 0.000 | 0.000 | 0.000 | 0.000 | 0.000 |  |

B

|  | CYP3A89 | CYP3A93 | CYP3A94 | CYP3A95 | CYP3A96 | CYP3A97 |
| --- | --- | --- | --- | --- | --- | --- |
| CYP3A89 |  |  |  |  |  |  |
| CYP3A93 | 0.000 |  |  |  |  |  |
| CYP3A94 | 0.000 | 0.000 |  |  |  |  |
| CYP3A95 | 0.007 | 0.007 | 0.007 |  |  |  |
| CYP3A96 | 0.000 | 0.000 | 0.000 | 0.007 |  |  |
| CYP3A97 | 0.000 | 0.000 | 0.000 | 0.007 | 0.000 |  |

|  | CYP3A89 | CYP3A93 | CYP3A94 | CYP3A95 | CYP3A96 | CYP3A97 |
| --- | --- | --- | --- | --- | --- | --- |
| CYP3A89 |  |  |  |  |  |  |
| CYP3A93 | 0.000 |  |  |  |  |  |
| CYP3A94 | 0.000 | 0.000 |  |  |  |  |
| CYP3A95 | 0.000 | 0.000 | 0.000 |  |  |  |
| CYP3A96 | 0.000 | 0.000 | 0.000 | 0.000 |  |  |
| CYP3A97 | 0.000 | 0.000 | 0.000 | 0.000 | 0.000 |  |

C

D

|  | CYP3A89 | CYP3A93 | CYP3A94 | CYP3A95 | CYP3A96 | CYP3A97 |
| --- | --- | --- | --- | --- | --- | --- |
| CYP3A89 |  |  |  |  |  |  |
| CYP3A93 | 0.102 |  |  |  |  |  |
| CYP3A94 | 0.102 | 0.000 |  |  |  |  |
| CYP3A95 | 0.102 | 0.000 | 0.000 |  |  |  |
| CYP3A96 | 0.099 | 0.05 | 0.05 | 0.05 |  |  |
| CYP3A97 | 0.102 | 0.000 | 0.000 | 0.000 | 0.05 |  |

E

|  | CYP3A89 | CYP3A93 | CYP3A94 | CYP3A95 | CYP3A96 | CYP3A97 |
| --- | --- | --- | --- | --- | --- | --- |
| CYP3A89 |  |  |  |  |  |  |
| CYP3A93 | 0.000 |  |  |  |  |  |
| CYP3A94 | 0.000 | 0.000 |  |  |  |  |
| CYP3A95 | 0.000 | 0.000 | 0.000 |  |  |  |
| CYP3A96 | 0.053 | 0.053 | 0.053 | 0.053 |  |  |
| CYP3A97 | 0.000 | 0.000 | 0.000 | 0.000 | 0.053 |  |

**Reference**

Kumar S, Stecher G, Tamura K. 2016. MEGA7: Molecular Evolutionary Genetics Analysis Version 7.0 for Bigger Datasets*. Molecular biology and evolution*, 33:1870-1874.
