# Supplementary material for: Phylogenetic analysis of the cytochrome P450 (CYP450) nucleotide sequences of the horse and predicted CYP450s of the white rhinoceros (Ceratotherium simum) and other mammalian species
Source: PeerJ. 2018 Oct 9;6:e5718. doi: 10.7717/peerj.5718 (PMC6183514; doi:10.7717/peerj.5718)
Supplement: Supplemental Information 2 [file peerj-06-5718-s002.docx]

Data sourced from the UCSC website (<https://genome.ucsc.edu/cgi-bin/hgGateway?hgsid=656118339_cTo7HC6CtymuWd58Rf7aPyxySf5B>)

The file contains the nucleotide sequence of all CYP enzymes (CYP11A1, CYP17A1, CYP19A1, CYP27B1, CYP2A13, CYP2C113, CYP2C92, CYP2D50, CYP2E1, CYP3A89, CYP3A93, CYP3A94, CYP3A95, CYP3A96 and CYP3A97) of the horse (*Equus caballus*) and the matching nucleotide sequences from the genomes of the white rhinoceros (*Ceratotherium simum*), the cow (*Bos taurus*), the dog (*Canis lupus familiaris*), the pig (*Sus scrofa*), the elephant (*Loxodanta africana*), the sheep (*Ovis aries*), the mouse (*Mus musculus*) and the human (*Homo sapiens*). A BLAT (BLAST-like Alignment Tool) search was performed to match and align the nucleotide sequence of the horse to that of the selected species.

>CYP11A1_horse

ATGCTGGTCAGGGGGCTTCCTCTGCGCTCAGTCTTGGTCAAAGGCTGCCAGCCCCTCCTGAGTGCTCCTCGGGAGGGCCCGGGGCACCCCAGGGTGCCCACTGGAGAGGGAGCCGGCATGTCCAGTCACAGCCCTCGCCCCTTCAAGGAGATCCCCTCCCCTGGTGACAATGGCTGGATAAACCTCTACCATTTCTGGAGGGAGAAGGGCCCAAAGAAATTACACTATCACCACTTCCAGAATTTCCAGAAGTATGGCCCCATTTACAGGGAGAAGCTTGGCAACGTGGAGTCAGTTTATATCGTGGACCCTGAAGATGTGGCTCTTCTCTTTAAGTTCGAGGGTCCCCATCCGGAACGATTTCTCATCCCACCCTGGACCGCCTATCACCAGTATTTTCAGAAACCTGTTGGGGTCCTGTTTAAGAGCTCAGACGCCTGGAAGAAAGACCGGCTAGCTCTGAACCCGGAGGTGATGGCTCTAGAGTCCATAAAGAACTTCATTCCCCTGCTGGACCCGGTGTCGCAGGACTTCGTCAGCCTCCTGCACAGGCGCATGGAGCAGCAGGGCTCCGGAAAGTTCTCTGGTCCCATCATTGAAGACCTGTTTCGCTTCGCCTTCGAGTCCATCACCAACGTCATATTTGGGGAGCGCCaGGGGATGCTGGACGAGATAGTGGACCCTGAGGCCCAGCGCTTCATTGATGCCGTCTACAAGATGTTCCACACCAGCGTCCCCATGCTCAGCCTCCCCCCAGACCTGTTCCGTCTGTTCAGGACCAAGACCTGGAGGGACCATGTGGCCGCATGGGACACAGTTTTTAGTAAAGCTGAACAATACACCGAGAAATTCTACCAGGACCTGAAACAGAAAAGACACTTCGACAGTTATCCAGGCATCTTCTACCGCCTCCTAGCAAGCAACAAGCTGCCCTTTAAGGACATCCAGGCCAACGTTACCGAGATGCTGGCGGGGGGCGTGGACACGACGTCCATGAgcCTGCAGTGGCACCTGTACGAGATAGCACGCAACCTAAGGGTACAGGAGATGCTGCGGGAGGAAGTCCTGGCTGCCCGGCGTCAGGCCCAGGGAGACACGAGCACGATGGTGCAGATGGTCCCACTGCTCAAAGCCAGCATCAAGGAGACCCTGAGACTCCACCCCATCGCCGTGACTTTGCAGAGATACCCCCAAAACGACTTGGTTATTCGAGATTACATGATTCCTGCCAAGACACTGGTGCAGGTGTCCATCTATACCATGGGCCAAGACCCCACCTTCTTCTCCAATCCGCGCCGTTTTGACCCGACCCGATGGCTGGATAAAAACAAGGACCTCACCCACTTCCGGAACCTGGGCTTTGGCTGGGGTGTGCGGCAGTGTTTGGGCCGGCGGATCGCCGAACTGGAGATGACCCTCTTCCTCATCCATATTCTGGAGAACTTCAGAGTTGAAATCCAACATCTCAATGACGTGGACAGCACATTCGGCCTCATCCTGATACCTGAAAAGCCCATCTCCTTCACCTTCTGGCCCATCACCCGGGCCCCACCCCAGGCGTGA

>CYP11A1_rhino

ATGCTGGTCAGGGGGCTTCCcCTGCGCTCAGTCcTGGTCAAAGGCTGCCAGCCCCTCCTGtGTGCcCCTCGGGAGGGCCtGGGGCAtCCCAGGGTGCCCACTGGAGAcGGAGCCGGCATcTCCAGTCACAGtCCTCGCCCCTTCAAtGAGATCCCtTCCCCTGGTGACAATGGCTGGcTAAACCTgTACCATTTCTGGAGGGAGAAGGGCtCAAAGAAAaTgCACTATCACCAacTCCAGAATTTCCAGAAGTATGGCCCCATTTACAGGGAGAAGCTcGGCAAtGTGGAGTCAGTgTATATCaTtGACCCTGAAGATGTGGCTCTTCTCTTTAAGTTCGAGGGTCCCaAcCCaGAACGATacCTCATCCCgCCCTGGgtCGCCTAcCACgAGTATTacCAGAAACCcaTTGGaGTCCTGTTTAAGaGaCTGGAAGAAAGACCGGtTAGCcCTGAACCaGGAGGTGATGGCTCcAGAGTCCATAAAGAACTTCATTCCCCTGCTGGAtgCtGTGTCtCgGGACTTCGTCAGCaTCCTGCACAGGCGCATcGAGCAGCAGGGCTCCGGAgAGTTgTCaGGggaCATCtgTGAAGACCTGTTTCGCTTCGCCTTCGAGTCCATCACCAACGTCATATTTGGaGAGCGCCtGGGGAcaCTGGAgGAGAcAGTGaACCCcGAGGCCCAGCagTTCATTGAcGCtGTCTACAAGATGTTCCACACCAGCGTCCCCATGCTCAaCtTCCCCCCgGgCCTGTTCCGTCTGcTgAGGACCAAGACCTGGAGGGACCATGTGGCatCATGGGACAtAaTTTTcAGTAAAGCTGAAaAATACACCcAGAAcTTCTACCAGGACCTGAAACAGAAAAGAgACTTCagCAaTTATCCgGGCATCcTCTACCGCCTCCTgGgAAGtAACAAGCTGCCgTTcgAGGACATCaAGGCCAACGTTACtGAGATGtTGGCaGGGGGCGTaGACACGACaTCCATGAcgCTGCAGTGGCACCTGTACGAGATgGgACGCAgCCTgAGGGTgCAGGAGATGCTGCGGaAGGAgGTCCTGaCTGCaCGGCGcCAGGCCCAGGGAGACAtGAaCAtGATGGTGCAGcTGGTCCCACTGCTCAAAGCCAGCATCAAGGAGACCCTGAGACTCCACCCtATCtCCGTGACccTaCAGAGATACatCacAAAtGACTTGGTTcTTCGAGATTACATGATTCCTGCCAAGACAtTGGTGCAGGcGTCCATCTATgCCATGGGCCAAGACCCCAaCTTCTTCTtaAATCCGCGCtGTTTTGACCCGACCCGATGGCTGGATAAAAACAAGGACCTCgtCCACTTCCGGAACCTGGGCTTTGGCTGGGGTGTGCGGCAGTGTTTGGGCCGGCGGATCGCCGAgCTtGAGATGACCCTCTTCCTCATCCAgATTCTGGAGAACTTCAGAGTTGAAATCCAACAcCTCAATGACGTGGACAcCACATTCaaCCTCATCCTGAcgCCTGAcAAGCCCATCTaCcTgACCTTCcGGCCCtTaACCCaGGaCCCACCCCAGGCaTGA

>CYP11A1_cow

cTcCAGGGAGAAGCTTGGCAAttTGGAGTCAGTTTATATCaTtcACCCTGAAGAcGTGGCcCaTCTCTTcAAGTTCGAGGGatCCtAcCCaGAgaGATaTgaCATCCCgCCCTGGctgGCCTATCACCgaTATTaTCAGAAACCcaTTGGaGTCCTGTTTAAGAagTCAGgaaCCTGGAAGAAAGACCGGgTgGtcCTGAACaCGGAGGTGATGGCTCcAGAGgCaATAAAGAACTTCATcCCaCTGCTGaAtCCaGTGTCtCAGGACTTCGTCAGCCTCCTGCACAaGCGCATcaAGCAGCAGGGCTCCGGAAAGTTtgtaGGggaCATCAagGAAGACCTGTTTCaCTTtGCCTTtGAGTCCATCACCAAtGTCATgTTTGGGGAGCGCCtGGGGATGCTGGAgGAGAcAGTGaACCCcGAGGCCCAGaagTTCATTGATGCCGTCTACAAGATGTTCCACACCAGtGTCCCtcTGCTCAaCgTCCCtCCAGAaCTGTaCCGTCTaTTCAGaACCAAGACtTGGAGGGACCATGTaGCCGCATGGGACACAaTTTTcAaTAAAGCTGAAaAATACACtGAGAtcTTCTACCAGGACCTGAgACgGAAAAcAgAaTTtaggAaTTAcCCAGGCATCcTCTACtGCCTCCTgaaAAGtgAgAAGaTGCtCTTggAGGAtgTCaAGGCCAAtaTTACgGAGATGCTGGCaGGGGGtGTGGACACGACaTCCATGAcatTGCAaTGGCACtTGTACGAGATgGCACGCAgCCTgAatGTgCAGGAGATGCTGCGGGAGGAgGTtCTGaaTGCCCGaCGcCAGGCagAGGGAGACAtaAGCAaGATGcTGCAaATGGTCCCACTtCTCAAAGCtAGCATCAAGGAGACgCTGAGACTCCACCCCATCtCCGTGACccTGCAGAGATACCCtgAAAgtGACTTGGTTcTTCaAGATTACcTGATTCCTGCCAAGACACTGGTGCAaGTGgCCATCTATgCCATGGGCCgAGACCCtgCCTTCTTCTCCAgTCCGgaCaagTTTGACCCaACCaGgTGGCTGagTAAAgACAAaGACCTCAtCCACTTCCGGAACCTGGGCTTTGGCTGGGGaGTGCGGCAGTGcgTGGGCCGGCGGATCGCCGAgCTGGAGATGACCCTCTTCCTCATCCAc

>CYP11A1_dog

ATGCTGGcCAaGGGGCTTCCTCTtCGCTCAGTCcTGGTCAAAGGCTGCCAGCCCtTCtTGAGcaCcgtgtGGGAGGGtCCaGGGCAtCCCAGGGTGCCCACcGGgGAtGGAGCCaGtATcTCCAcTCAgAttCCTCGCCCCTTCAgtGAGATCCCCaCCCCaGGaaACAATGGCTGGcTAAACCTgTACaATTTCTGGAGGGAaAtGGGCtCAcAGAAAaTcCACTATCACCAagTCCAGAATTTCCAGAAGTATGGCCCCATTTACAGGGAGAAGCTgGGCAgCGTGGAGTCgGTTTATATCaTcGACCCgGAAGATGTGGCcCTTCTgTTTAAGTTtGAGGGTCCCacTCCaGAACGATTTtgCATCCCgCCCTGGgtgGCCTATCACCAGTATcacCAGAgACCcGTcGGcGTCCTGTTgAAGAagTCAGgaGCgTGGAAGAAAGACCGGCTgGCTtTGAACCaGGAGGTGATGGCTCcAGAGgCCATAAAGAACTTCATTCCCCTGCTGGACCCaGTGTCtCAGGACTTtGTCAaagTCCTGCACAGGCGCATcaAGCAGCAGGGCTCCGGAAAGTTCTCaGGagaCATCAgTGAtGAtCTGTTTCGCTTCGCCTTtGAGTCCATCACCAAtGTCATgTTcGGGGAGCGCCtGGGGATGCTGGAgGAGAgAGTGGACCCgGAGGCCCAGCGgTTCATcGATGCtGTCTACcAGATGTTtCACACCAGtGTCCCCATGCTCAcCtTCCCCCCAGACCTGTTCCGTCTGTTCAaGACCAAGACCTGGAGGGACCATGTGGCtGCATGGGAtgtgaTTTTcAacAAAGCcGAgatcTACACCcAGAAcTTCTACtgGGAaCTGAggCAGAAgcGAgACgTCGACAaTTAcCCgGGCATCcTgcACCGCCTCCTAaaAAGtAACAAGCTGCttTTcgAGGACgTCaAGGCCAAtaTcACaGAGATGCTGGCGGGGGGtGTGGACACGACGTCCATGAcaCTGCAGTGGCACCTcTACGAGATgGCACGCAgCCTggaGGTgCAGGAGgTGCTGCGGGAGGAgGTCCTGGCcGCgCGGCGcCAGGCCCAGGGAaACgtGAGCACGATGcTGCAGcTGGTCCCgCTcCTCAAAGCCAGCATCAAGGAGACaCTGAGACTCCACCCCATCtCCGTGACccTGCAGAGATACCttgAAAAtGACTTGGTTcTTCGAaATTACATGATTCCcGCCAAGACgtTGGTGCAGGTGTCCAcCTATgCCATGGGCCAgGACCCCACCTTCTTCctCAAcCCGaGCaagTTTGACCCaACCCGcTGGCTGGggAAggACAAGGAgCTCAtCCACTTCCGGAACCTGGGCTTTGGCTGGGGcGTGCGGCAGTGcgTGGGCCGGCGtATCGCtGAgCTcGAGATGACCCTCTTCCTCATCCAc

>CYP11A1_pig

TCAGgaGCCTGGAAGAAAGACCGGtTgGtcCTGAACaCGGAGGTaATGGCTCcAGAGgCCATAAAGAACTTCATcCCCCTaCTGGACaCaGTGTCtCAGGACTTCGTCgGCgTCCTGCACAGGCGCATcaAGCAGCAGGGtTCtGGAAAGTTCTCaGGTgaCATCAggGAAGACCTGTTTCGCTTCGCCTTtGAGTCCATCACCAAtGTCATATTTGGaGAGCGCCtGGGGATGCTGGAgGAaATAGTGGACCCTGAGGCtCAGaagTTCATTGATGCtGTCTACcAGATGTTCCACACtAGtGTCCCCATGCTCAatCTCCCCCCtGACCTGTTCCGTCTGTTCAGGACCAAGACCTGGAGGGACCATGTaGCCGCATGGGACACtaTTTTcAaTAAAGCTGAAaAATACACCcAGAAcTTCTACtgGGACCTGAgACgGAAAAGAgAaTTtaACAaTTAcCCAGGCATCcTCTACCGCCTCCTgGgAAatgACAAGCTGCtCTcagAaGAtgTtaAGGCCAAtGTTACCGAGATGCTGGCaGGGGGtGTaGACACGACaTCCATGActCTGCAaTGGCACtTGTAtGAGATgGCACGtAgCCTgAatGTgCAGGAGATGCTGCGGGAGGAgGTCCTcaaTGCCCGGCGcCAGGCCCAaGGAGACACaAGCAaGATGcTGCAatTGGTCCCACTcCTCAAAGCtAGCATtAAGGAGACaCTGAGACTCCACCCCATCtCCGTGACccTGCAGAGATACCtCgtgAAtGACTTGGTTcTTCGAGATTACATGATTCCTGCCAAGACAtTGGTaCAaGTGgCCgTCTATgCCATGGGCCgAGACCCCgCCTTCTTCTCaAATCCagGCCagTTTGACCCaACCCGgTGGtTGGgTAAAgAaAgGGACCTCAtCCACTTCCGGAACtTGGGCTTTGGCTGGGGcGTGCGGCAGTGTgTGGGCCGGCGGATCGCtGAgCTcGAGATGACtCTCTTCCTCATCCAtATTCTGGAGAACTTCAagGTTGAAtTgCAgCActTCAgTGAtGTGGACAcCAtATTCaaCCTCATCCTGATgCCgGAcAAGCCCATCTt

>CYP11A1_elephant

ATGCTGGctAGaGGaCTcCCcCcaCGCTCAGTCcgGcTCAgAGGCTtCCgGtCCCTCCTGAGTGCcCtgtGGGAGGGCCCGGGGCAtCCCAGGGTGCCgACTGGAGAaGGAGCCAGCCCTCGCCCCTaCAgtGAagTCCCtTCCCCTGGTGACAATGGCTGGcTgAACCTgTACCAcTTCTGGAGGGAGAAGGGtttgcAccAAcTcCACTAcCgCCAggTCCAGAATTTCCAGAAGTtTGGCCCCATTTACAgGGAGAAGCTaGGCAcCaTGGAcTCgGTTTtTATtGTcagCCCTGAAGATGcGGCcaTTCTCTaTAAGTctGAGGGcCCCCtcCCaGAACGcTacCaCgTCCCACCCTGGACtGCtTATCACCAGcATTacaAGAAACCcaTcGGGGTCCTGTTcAAGAagTCAGAaGaCTGGAAGAAAGACCGGtTAGCcCTGAACCaGGAGcTGATGGCTCTgGAGagCATAAAaAACTTCgTgCCCCTGCTGGACCCaGTGTCtCAGGACTTCGTCAGCCTCtTGCACAGGCGCATcaAtCAGCAaGGCTttGGgAAGTaCTCaGGggaCATCAgTGAtGACCTGTTcCGCTTtGCCTTtGAGTgTCACCAACGTtgTATTTGGGGAGCGgCtGGGGATaCTGcAgGAGAcgGTGGAtCCTGAGGCCCAGCagTTCATTGATGCgGTaTACcAGATGTTCCACACtAGCGTCCCCATGCTCAGCaTCCCCCCAGAtCTGTTCCGcCTGcTCAGGACCAAGACCTGGAGGGACCAcGTGGCtGCATGGGAggtgaTTTTcAGTAAAGCTGAACAATAtACCcAGAggTTCTAtggGGACCTGAgACAaAAAAaAgttTTtGACAaTTAcCCtGGCATCcTCTACCGCCTCCTgGacAGtAgCAAGCTGCCCTTTgAcaACATCaAGGCCAAtGcTAttGAacTGCTGGCtGGtGGCGTGGACACGACaTCCATGAcaCTGCAGTGGaACaTGTAtGAGATgGCcCGCAgCCTgAGGGTACAGGAGgTGCTGaGGGcaGAgGTCCTGGCTGCCCGGCGcCAGGCCaAGGGAGACAtGAaCAtGATGcTcaAGtTGGTtCCgCTcCTCAAAGCCAGCATCAAaGAGACgCTGAGgCaagtCCaCATAGACAtTGGTaCAGGTGgCCgTCTtcgCCATGGGtCggGAtCCCgCCTTCTTCTCCAAcCCagagtGTTTTGACCCagCCCGATGGCTaaAcAAAgACAAGGACaTCACCtACTTCCGGAACCTGGGCTTTGGCTGGGGTagcCGGCAGTGTgTGGGgCGGCGcATCGCCGAggTtGAGATGACaaTCTTCCTtATCCAT

>CYP11A1_human

ATGCTGGcCAaGGGtCTTCCcCcaCGCTCAGTCcTGGTCAAAGGCTGCCAGaCCtTtCTGAGTGCcCCcaGGGAGGGgCtGGGGCgtCtCAGGGTGCCCACTGGcGAGGGAGCtGGCATcTCCAccCgCAGtCCTCGCCCCTTCAAtGAGATCCCCTCtCCTGGTGACAATGGCTGGcTAAACCTgTACCATTTCTGGAGGGAGAcGGGCaCAcAcAAAgTcCACctTCACCAtgTCCAGAATTTCCAGAAGTATGGCCCgATTTACAGGGAGAAGCTcGGCAACGTGGAGTCgGTTTATgTCaTcGACCCTGAAGATGTGGCcCTTCTCTTTAAGTcCGAGGGcCCCaAcCCaGAACGATTcCTCATCCCgCCCTGGgtCGCCTATCACCAGTATTacCAGAgACCcaTaGGaGTCCTGTTgAAGAGCTCgGcaGCCTGGAAGAAAGACCGGgTgGCcCTGAACCaGGAGGTGATGGCTCcAGAGgCCAccAAGAACTTttTgCCCCTGtTGGAtgCaGTGTCtCgGGACTTCGTCAGtgTCCTGCACAGGCGCATcaAGaAGgcGGGCTCCGGAAAtTaCTCgGGggaCATCAgTGAtGACCTGTTcCGCTTtGCCTTtGAGTCCATCACtAACGTCATtTTTGGGGAGCGCCAGGGGATGCTGGAgGAagTAGTGaACCCcGAGGCCCAGCGaTTCATTGATGCCaTCTACcAGATGTTCCACACCAGCGTCCCCATGCTCAaCCTtCCCCCAGACCTGTTCCGTCTGTTCAGGACCAAGACCTGGAaGGACCATGTGGCtGCATGGGACgtgaTTTTcAGTAAAGgTGAgTAcCgtGGCATCcTCTACaGaCTCCTgGgAgaCAgCAAGaTGtCCTTcgAGGACATCaAGGCCAACGTcACaGAGATGCTGGCaGGaGGgGTGGACACGACGTCCATGAcCCTGCAGTGGCACtTGTAtGAGATgGCACGCAACCTgAaGGTgCAGGAtATGCTGCGGGcaGAgGTCtTGGCTGCgCGGCacCAGGCCCAGGGAGACAtGgcCACGATGcTaCAGcTGGTCCCcCTcCTCAAAGCCAGCATCAAGGAGACCCgcAGACTtCACCCCATCtCCGTGACccTGCAGAGATAtCttgtAAAtGACTTGGTTcTTCGAGATTACATGATTCCTGCCAAGACACTGGTGCAaGTGgCCATCTATgCtcTGGGCCgAGAgCCCACCTTCTTCTtCgAcCCGgaaaaTTTTGACCCaACCCGATGGCTGagcAAAgACAAGaACaTCACCtACTTCCGGAACtTGGGCTTTGGCTGGGGTGTGCGGCAGTGTcTGGGaCGGCGGATCGCtGAgCTaGAGATGACCaTCTTCCTCATCaCTGGAGAACTTCAGAGTTGAAATCCAACAcCTCAgcGAtGTGGgCAcCACATTCaaCCTCATtCTGATgCCTGAAAAGCCCATCTCCTTCACCTTCTGGCCCtTtAaCCaGGaagCAaCCCAGcaGTG

>CYP11A1_sheep

ATGCTGGcCAGGGGGCTTCCctTcCGCTCAGcCcTGGTCAAAGcCTGCCcaCCCCTCCTGAacaCgggcaGGGAGGGCtgGGGcCACCaCAGGGTGggCACTGGAGAGGGAGCtGGCATcTCCAcgaggAcCCCTCGCCCCTaCAgtGAGATCCCCTCCCCTGGTGACAAcGGCTGGATtAACCTgTACCATTTCTGGAGGaAGAAGGGCtCAcAGAgAaTcCACTtTCACCACaTCgAGAAcTTCCAGAAGTATGGtCCCATTTACAGGGAGAAGCTcGGCAAttTGGAGTCAGTTTATATCaTtcACCCTGAAGAcGTGGCcCaTCTCTTTAAGTTCGAGGGatCCtAcCCacAgaGATaTgaCATCCCgCCCTGGctgGCCTATCACCAGTATTaTCAGAAACCcaTcGGaGTCCTGTTTAAGAagTCAGgaGCCTGGAAGAAAGACCGGgTgGtcCTGAACaCGGAGGTGATGGCTCcAGAGgCaATAAAGAACTTCATcCCaCTGCTGaAtCCaGTGTCtCAGGACTTCGTCAGCCTCCTGCACAaGCGCATcaAGCAGCAGGGCTCCGGAAAGTTtgtaGGggaCATCAagGAAGACCTGTTTCGCTTtGCCTTtGAGTCCATCACtAAtGTCATgTTTGGGGAGCGCCtGGGGATGCTGGAgGAcAcAGTGGACaCcGAGGCCCAGaagTTCATTGATGCCGTCTACAAGATGTTCCAtACCAGtGTCCCtcTGCTCAaCCTCCCtCCAGAaCTGTaCCGTCTGTTCAGaACCAAGACtTGGAGGGACCATGTaGCCGCATGGGAtACAaTTTTcAaTAAAGgTGAgGCCAAtaTTACgGAGATGCTGGCcGGGGGtGTGGACACGACaTCCATGAcgCTGCAGTGGCACtTGTACGAGATgGCACGCAgCCTgAatGTgCAGGAGATGCTGCGGaAGGAgGTtCTGaaTGCCCGaCGcCAGGCagAGGGcGACAtaAGCAaGATGcTGCAaATGGTCCCACTtCTCAAAGCtAGCATCAAGGAGACgCTcAGACTCCACCCtATCtCCGTGACccTGCAGAGATACCCtgAAAgtGACTTGGTTcTTCaAGATTACcTGATTCCTGCCAAGACACTaGTGCAaGTGgCCATCTATgCCATGGGCCgAGACCCtgCCTTCTTCTCCAATCCGgaCaagTTTGACCCaACCaGgTGGCTGGgTAAAgACAAGGACCTCAtCCACTTCCGGAACCTGGGCTTcGGCTGGGGaGTGCGGCAGTGcgTGGGCCGGCGGATCGCCGAgCTGGAGATGACCCTCTTCCTCATCCAcATTCTGGAGAACTTCAGgGTTGAAATgCAgCAgaTCggTGAtGTGaACAcCAtcTTCaaCCTCATCCTGActCCgGAcAAGCCCATC

>CYP11A1_mouse

ATGCTGGctAaaGGaCTTtCcCTGCGCTCAGTgcTGGTCAAAGGCTGCCAaCCttTCCTGAGcACTGGAaAGGGAGCtGGtAccTCtAcTagCAGtCCTaGgtCCTTCAAtGAGATCCCtTCCCCTGGcGACAATGGtTGGcTAAACCTgTACCAcTTCTGGAGGGAGAgtGGCaCAcAGAAAaTcCAtTAcCAtCAgaTgCAGAgTTTCCAaAAGTATGGCCCCATTTACAGGGAGAAGCTgGGCActtTGGAGTCAGTTTAcATCGTGGACCCcaAgGATGcGtCgaTaCTCTTctcaTgCGAGGGTCCCaAcCCGGAgCGgTTcCTtgTgCCcCCCTGGgtgGCCTATCACCAGTATTaTCAGAggCCcaTTGGGGTCCTGTTTAAGAGtTCAGAtGCCTGGAAGAAAGACCGaaTcGtcCTaAACCaaGAGGTGATGGCgCctGgagCCATcAAGAACTTCgTgCCCCTGCTGGAaggtGTagCtCAGGACTTCaTCAaagTCtTaCACAGaCGCATcaAGCAGCAaaatTCtGGAAAtTTCTCaGGggtCATCAgTGAtGACCTaTTcCGCTTttCCTTtGAGTCCATCAgCAgtGTtATATTTGGGGAGCGCatGGGGATGCTGGAgGAGATcGTGGAtCCcGAGGCCCAGCGgTTCATcaATGCtGTCTACcAGATGTTCCACACCAGtGTCCCCATGCTCAaCCTgCCtCCAGACtTcTTtCGaCTccTCAGaACtAAGACCTGGAaGGACCATGcaGCtGCcTGGGAtgtgaTTTTcAaTAAAGCTGAtgAgTACACCcAGAAcTTCTACtgGGACtTaAggCAGAAgcGAgACTTCagCcagTAcCCtGGtgTCcTtTAtaGCCTCCTgGgggGCAACAAGCTGCCCTTcAAGaACATCCAGGCCAACaTTACCGAGATGCTGGCaGGaGGgGTGGACACGACcTCCATGAcCCTGCAGTGGaACCTtTAtGAGATgGCACaCAACtTgAaGGTACAGGAGATGCTGCGGGctGAAGTCCTGGCTGCCCGGCGcCAGGCCCAGGGAGACAtGgcCAaGATGGTaCAGtTGGTtCCACTcCTCAAAGCCAGCATCAAGGAGACaCTGAGACTCCACCCCATCtCCGTGACcTTGCAGAGgTACaCtgtgAAtGACcTGGTgcTTCGtaATTACAaGATTCCaGCCAAGACttTGGTaCAGGTGgCtAgCTtTgCCATGGGtCgAGAtCCgggCTTCTTtcCCAATCCaaaCaagTTTGACCCaACtCGtTGGCTGGAaAAAAgCcAaaAtacCACCCACTTCCGGtACtTGGGCTTTGGCTGGGGTGTtCGGCAGTGTcTGGGCCGGCGGATtGCgGAgCTGGAGATGACCaTCcTCCTtATCaCTGGAGAACTTCAGAaTTGAAgTtCAAaATCTCcgTGAtGTGGggAcCAagTTCaGCCTCATCCTGATgCCTGAgAAcCCCATCctCTTCAaCTTCcaGCCt

>CYP17A1_horse

CCACAGCTGTCTGTGCTGCCTGCTGAGACTGGCCACAATGTGGGAGCTCTTGGCTTTCCTGCTGTTGGCCATAGCCTATTTCTTTCGGCCCAAGGTTAAGTGCCCTGGTGCCAAGTACCCCAAGAGCCTCCCATACCTGCCCTTGGTGGGCAGCCTGCCGTTCCTTCCCAGACATGGCCATCCGCATGTGAACTTCTTCAAGCTGCAGAAAAAATACGGCCCCATCTATTCCTTGCGCATGGGTACCAAGACTACGGTGATGGTCGGCCACTACCAACTGGCCAAGGAGGTGCTTATCAAGAAGGGCAAGGAATTCTCCGGGCGGCCCCAAGTGGCAACTCTAAACATCCTGTCAGACAATCAAAAGGGTGTCGCCTTCGCCGACCATGGTGCCCCCTGGCAGCTGCATCGGAAGCTGGTGCGGGCTGCTTTTGCCCTGTTCAAGGACGGCAACCAGAAGCTGGAGAAGATTATATGTCATGAAACCAGTTTATTGTGTGACTTACTGGCCACTCAGAATGGACAGACCATAGACTTGTCCTCGCCTCTCTTCCTGGCGGTGACCAACGTAATCTGCTGGATCTGCTTCAACTCCTCCTACATGAAAGGGGATCCTGCGCTGGAGACCATGCAGAATTACCACAAAGGCATCCTGGAaACcCTGGAAAAAGACAATGTGGTAGACATATTCCCCGCATTGAAGATTTTCCCCAACAAATCCCTGGAAAAAATGAGGCATTGTGTTAACATACGAAATGAACTGCTGAGTAAAATCTTTGAAAAACATAAGGAGAACTTCAACAGTGACTCCATCACTAGCATGCTGGACTTACTGATCCAAGCCAAGAAGAACTCAGACAATAACAACACTGGCCCAGACCAGGATTCAAAGCTGCTTTCGGATAAACACATTCTTGCCACCATAGGGGACATCTTCGGGGCCGGTGTGGAGACCACCACCTCTGTGGTGAAGTGGATTGTGGCCTTCCTGCTGCACGACCCTCAGCTGAAGAAGAAGATCCAGGAGGAGATTGACCAGAATGTGGGTTTCAGCCGCACACCAACACTCAGTGACCGGAACCGCCTCCTTCTGCTGGAGGCCACCATCCGAGAAGTGCTTCGTATCCGGCCTGTGGCCCCTATGCTCATCCCCCATAAGGCTCTTGTTGACTCCAGCATCGGCGAGTTTGCTGTTGACGAGGGCACAAACGTCATCATCAATTTGTGGGCACTCCATCACAATGAGAAGGAGTGGCACCAGCCTGACCGGTTCATGCCTGAGCGCTTCTTGGACCCAACGGGGAGCCAGCTCATCTCACCGTCGTTAAGCTACTTGCCCTTTGGAGCAGGTCCCCGCTCTTGCATAGGTGAGCTCCTGGCCCGCCAGGAGCTCTTCCTCTTCACGGCCTGGTTGCTGCAGAGGTTTAACCTGGAGGTCCCGGATGATGGCCAGCTGCCCTCCCTGGAGGGCCACCCCACAGCGGTCTTTCTGATCGACTCTTTCAAAGTGAAGATTAATGTGCGCCAGGCCTGGAGGGAGGCCCAGGCTGAGGGCAGCACCTAGAGGCTGTACCTAGCACCCCTGACTCCACCCTGTGTGACCCACAGCACAGAGTCAGAGGTACCACTCCATCCTCCCCCACCCTTTCTCCCTTCTCTCGGGGGCCCACTCTGCCTTCTTTTCCAGCCTGCAGCCCTGGCAGTGATGTGTATAAACCAAATGGTTTTTCTTCAGAAGGCCCCTGAGCAGCTCATCATTTATTCATTTGGTCTCTCTTTCAACAATTATTTTATTGAGCATCTACTACGTGCTGCTCATTGTCCCTGGACACTTGAGCTCCCTGTTCTCATGaAAGCTTACATTCTAGTGAGGGCTGACAAATAAAGAAGGATTTCAGAGACTTGT

>CYP17A1_rhino

CCACAGCTGTCTGTcCTGCCTGCcagGACcGGCCACcATGTGGGAGtTCTTGGCTTTCtTGCTGTTcGCCATAGCCTATTTCTTTtGGCCCAAGGTTAAGTGCCCTGGcGCCAAGTACCCCAAGAGCCTCCCgTgCCTGCCCTTGGTGGGCAGCCTGCCGTTCCTTCCCAGACATGGCCATCtGCAcGTGAAaTTCTTCAAGCTtCAGAAAAAATACGGCCCCATCTtTTCCTTaCGtATGGGTgCCAAGACTACaGTGATtGTCGGCCACcACCAgCTaGCaAAGGAGGTGCTTATCAAGAAGGGCAAGGAgTTCTCCGGGCGGCCCCAAGTGGaAACTCTAAACATCtTGTCAGACAAcCAAAAGGGTaTCGCCTTCGCCGACtAcGGTGCCCCCTGGCAGCTGCATCGGAAGCTGGTGCtGGCcaCcTTTGCCCTGTTCAAGGACGGCAACCAGAAGCTGGAGAAGATTATATGTaAgGAAgtCAGTTTATTGTGTGACTTcCTGGCCACcCAGAATGGACAGAgCATAGACTTGaCCTtGCCTCTCTcCCTGGCGGTGACCAACGTcATCTGCTtGtTCTGCTTCAACTCCTCCTACAaGAAtGGGGATCCcGaGCTGaAGACCATGCAGAAcTACaAtgAcGGCATCCTGGAcAgtCTGaAAcAgGACtgTaTGGTAGACATATTCCCtGCgTTGAAGATTTTCCCCAACAAAaCCCTGGAAAAgATGAGGCATTGcGTTAAaATACGAAATGAAaTGCTGAGTcAAATCcTTGAAAAACATAAGGAGAACTTCAACAGTGACTCtATCACTAcCATGCTGGACgTACTGATCCAAGCCAAGAAGAACTCAGACAATAACAAtgCTGGCCCAGACCAGGATTCAAAGCTGtTTTCGGATAAACACATTCTcaCCACCgTAGGGGACgTCTTtGGGGCtGGTGTGGAGACCACCACCTCTGTGGTGAAGTGGATTGTGGCCTTCCTGCTGCACcACCCTCAGCTGAAGAAGAAGATCCAGGAGGAGATTGACCAGAATGTGGGTTTCAGCCGCACACCAACtaTCAGTGACCGGAACCGCCTCCTTCTGCTGGAGGCCACCATCCGAGAgGTGCTTCGcATCCGGCCTGTGGCCCCTATGCTCATCaCCCATAAGGCTaTcGTTGACTCCAGCATCGGCGAGTTTGCTGTTGACaAGGGCACAAAtGTCATCATCAATTTGTGGGCACTgCATCACAAcGAGAAGGAGTGGCACCAGCCTGACCaGTTCATGCCcGAGCGtTTCTTGGACCCAACaGGGAGCCAGCTCATCTCgCCGTCcTTAAGCTACTTGCCCTTTGGAGCgGGaCCCCGCTCcTGCATAGGTGAGaTCCTGGCCCGCCAGGAGCTCTTCCTCTTCAtGGCCTGGTTGCTGCAGAGGTTcgACCTGGAGcTCCCGGATGATGGgCAGCTGCCCTCCCTGGAGGGCaACCCCACgatGGTCTTTCTGATtGAaTCTTTCAAgGTGAAGATcAccGTGCGCCAGGCCTGGAGGGAaGCCCAGGCTGAGaGtAGCACCTAGAGGtTGTACCTAGCcCCgACTCCACCCTGTGTGgCCCACAGCACAGAaTCAGAGGTgCtACTCCAcCCTCCCCCACCacTCCtccCTtcCcccaGCCCACTCTGCCTTCTTTcCCAGCCTGCAGCCCTGGCAGTGAcATAAACtAAATtGTTTTTCTTCAGAAGGCCCCTGAGCAGCTCAc

>CYP17A1_cow

ACAATGTGGctGCTCcTGGCTgTCtTtCTGcTcaCCcTcGCCTATTTaTTTtGGCCCAAGaccAAGcaCtCTGGTGCCAAGTACCCCAgGAGCCTCCCATcCCTGCCCcTGGTGGGCAGCCTGCCGTTCCTcCCCAGACgTGGCCAgCaGCAcaaGAACTTCTTCAAGCTGCAGgAAAAATAtGGCCCCATCTATTCCTTtCGttTGGGTtCCAAGACgACtGTGATGaTtGGaCACcACCAgtTGGCCAgGGAGGTGCTTcTCAAGAAGGGCAAGGAATTCTCtGGGCGtCCCaAAGTGGCcACTCTAgACATCCTGTCAGACAAcCAAAAGGGcaTtGCCTTtGCCGACCATGGTGCCCaCTGGCAGCTGCATCGGAAGCTGGcaCtGaaTGCcTTTGCCCTGTTCAAGGAtGGCAACCtGAAGtTaGAGAAGATcATCTGCTGGATCTGCTTCAACTtCTCCTtCAaGAAtGaGGATCCTGCcCTGaAGgCCATaCAaAATgtCaAtgAtGGCATCCTGGAggttCTGagcAAgGAagtTcTGtTAGACATATTCCCTTGcAGATTTTCCCCAgCAAAgCCaTGGAAAAgATGAaGggTTGTGTTcAaATgCGAAATGAAtTGCTGAaTgAAATCcTTGAAAAAAGGAGAACTTCAgCAGTGAtTCCATCACTAaCtTGCTGcACaTACTGATCCAAGCCAAGgtGAAtgCAGACAATAACAAtgCTGGCCCAGACCAGGATTCAAAGCTGCTTTCaaATAgACACATgCTTGCtACCATAGGGGACATCTTCGGGGCtGGTGTaGAGACCACCACgTCTGTGaTaAAGTGGATcGTGGCCTaCCTGCTaCACcAtCCTCAGtTGAAGAAGAgGATCCAGGAtGAcATTGACCAGAtTaTaGGTTTCAatCGCACcCCAACcaTCAGTGACCGGAACCGCCTtgTcCTGCTGGAGGCgACCATCaGAGAAGTGCTcCGaATCCGGCCTGTGGCCCCTAcGCTgATCCCCCAcAAGGCTgTcaTTGACTCCAGCATtGGCGAccTTaCcaTTGACaAaGGCACAgACGTtgTggTCAAccTGTGGGCACTgCATCACAgTGAGAAGGAGTGGCAgCAtCCcGACCtGTTCATGCCcGAGCGCTTCTTGGACCCcACGGGGgcgCAaCTCATCTCgCCaTCGTTAAGCTACTTGCCCTTTGGAGCAGGaCCCCGCTCcTGCgTAGGTGAGaTgCTaGCCCGCCAGGAGCTCTTCCTCTTCAtGtCCTGGcTGCTGCAGAGGTTcAACCTGGAGaTCCCGGATGATGGgaAGCTaCCCTCtCTGGAGGGCCAtgCCAgtctcGTCTTgCaGATCaAacCTTTCAAgGTGAAGATcgAgGTGCGCCAGGCCTGGAaGGAaGCCCAGGCTGAGGGtAGCACCTcGACTCCACCCTaTGTGACCCACcGCACAGAaTtAGAGGagCtcCcCCATCCTCtCCCACCCCtTctTCctcccctCCCACTCTGCCTTCTTTcCCAGCCTGCAGCCCTGGCAGTGATGTacATAAAaCAgtTtcTTTcTCc

>CYP17A1_dog

CTGGCtACcATGTGGGAaCTCTTGGtTTTCtTGCTGTTcaCCcTgGtCTATTTCTTatGGCCCAAGtcaAAGTGCCCTaaTGCCAAGTAtCCCAgGAGCCTCCCATcCCTGCCCTTGGTaGGCAGCCTGCCaTTCCTcCCCAGAgATGGCCAcCaGCAcGTGAgCTTCTTCAAGCTaCAGAAAAAATACGGCCCCATCTATTCCTTtCGtATGGGTACCAAGACTACaGTGATGGTCGGCCACcACCAgCTGGCCAAGGAGGTGCTTATCAAGAAGGGCAAGGAATTCTCtGGGCGGCCCCgAGTGGtATCCTGTCtGACAAcCAAAAGGGTaTCGCtTTCGCaGACCATGGTGCCagCTGGCAGCTGCAcaGGAAaCTGGcaCtGGCcaCcTTTGCCCTGTTCAAGGAtGGCAACCAGAgGCTaGAGAAGATcATATGTCAgGAAAaCAGTTTATTaTGTGAtTTcCTGGCCACcCAGAATGGAaAGtCCATAGAtTTGTCCTtGCCTCTCTTCCTGGCGGTGACCAAtaTAATCTGCTtGATCTGCTTCAACaCtTCCTACAaGAAtGGaGATCCaGCtCTGGAGAtCATaaAGAATTACaACgAtGGCATCtTGGAtACttTGGgAAggGAtcATaTGaTAGACATATTCCCCGggTTGAAGATcTTCCCCAACAAAaCCCTGGAAAAAATGAGGaATTGTGTTAAaATgCGAgATGActTGCTGAaTgAAATCcTTGAAAAAtATAAGGAGAAaTTCAgCAGcGACTCtATCACcAaCATGCTaGACaTACTGATCCAAGCCAgGAtGAACTCcGACgATAACAAtgCTGGCtCAGACCgGGATTCAAAGCTGCTTTCaGATAAACAtATTCTcatCAgCATAGGGGACATtTTtGGGGCCGGTGTGGAGACCACCACgTCTGTGGTGAAGTGGAcTGTGGCCTTCCTGCTaCACaACCCTCAGtTGcAGAAGAAGATCCAGGAGGAGATTGAtCAGAATGTaGGTTTtgGCCGCAtACCAACtaTgAGTGACCGGAgCaaaCTCaTctTGCTaGAGGCCACCATCaGAGAgGTGCTTCGcATtCGGCCTGcGGCCCCTATGCTCATCCCCCAcAAGGCTaTTGTcGAtTCCAGCATtGGtGAGTTTGCTGTTGACaAGGGCACAAgtGTtATCATCAATcTGTGGGCgCTgCATCACAATGAGAAGGAGTGGtACCgGCCTGACCaGTTCATGCCaGAGCGCTTCcTGGACgCcACaaaGAGtCAGCTCATCTCtCCaTCaTTAAGtTACTTGCCCTTTGGAGCAGGaCCtCGCTCcTGtgTAGGTGAGaTCCTGGCCCGCCAGGAaCTCTTCCTCgTCAtGGCCTGGTTGCTGCAGAGGTTcgACCTGGAGGcCCCaGATGATGGgCAGCTGCCtTCCCTGGAGGGCatCCCCAgAGtGGTCTTTtTGATCGAgTCTTTCAAAGTtAAGATcAAgGTGCGCCAGGCCTGGAGGGAaGCCCAGGCTGAGGGtAGCACCTAGAGGCTGacCtagcatCCCCTGACTCCACCCTGTGTGAtCCcACaCTGCCTTCTTTTCCAGCCTGCAGtCCc

>CYP17A1_pig

CCAgAGCTGTCTGTcCTGCCTGCcGccACTGGCCACcATGTGGGtGCTCTTGGtTTTCtTctTGcTcaCCcTcaCCTATTTaTTTtGGCCtAAGaccAAGgGCtCTGGTGCCAAGTACCCCAgGAGtCTCCCAgtCCTGCCCgTGGTGGGCAGCCTGCCaTTCCTaCCCAGACgTGGCCAcCaGCAcaTGAACTTCTTCAAGtTGCAGgAcAAATAtGGCCCCATCTtcTCCTTtCGtcTGGGTtCCAAGACTACcGTGgTaaTtGGtgACcACCAgCTGGCCAAGGAGGTGCTTcTCAAGAAGGGCAAGGAATTCTCCGGGCGGCCCagAGTGGtAATCTAgACATCCTGTCAGACAAcCAAAAGGGgaTtGCCTTCGCCGACCATGGTaCCtCCTGGCAGCTGCATCGGAAGCTGGcaCtGagcaCcTTTtCCCTGTTCAAGGgtGGCAACCtGAAGCTGGAGAAcATcTCAaGAAAtCAaagTAcTGTGcGAtTTcCTGGCCACaCgGAATGGAgAGtCCATtGAtTTGgCCcaGCCTCTCTctCTGGCGaTGACCAACaTAgTCaGCTttATCTGCTTCAACTtCTCCTtCAaGAAgGGGGATCCcGCGCTGcAGgCCATagtGAATTtCaAtgAtGGCATCCTGGATCCATCACTAaCtTGtTGGACaTAaTGATCCAAGCCAAGAcGAACgCAGAaAgTAACActggTGGCCCAGACCAcaATTtAAAGCTGCTTTCaGAcAgACACATgCTcGCCACtgTtGcGGACATCTTtGGGGCCGGTGTGGAGACttCtgCCTCTGTGGTaAAGTGGATcGTGGCCTTCCTGCTaCACtACCCTCAGCTGAgGAAGAAGATCCAGGAtGctATcGACCAGAATaTtGGTTTCAatCGtgCcCCAtCtaTCAGcGACCGGAACCaaCTtgTcCTcCTGGAGGCCACCATCCGAGAgGTGCTTCGatTCCGGCCTGTGtCCCCTAcGCTCATCCCCCAcAgGGCTaTcaTTGACTCCAGCATtGGCGAaTTTaCcaTTGACaAGGaCACAgAtGTCgTCgTCAATcTGTGGGCACTgCATCACAATGAGAAGGAGTGGCACCgGCCcGACCtGTTCATGCCTGAGCGCTTCcTGGACCCcACGGGaAcCCAGCTCATCTCACCaTCaTTgAGCTACTTGCCCTTcGGAGCAGGaCCCCGCTCTTGCgTAGGgGAGaTgCTaGCCCGCCAGGAGCTCTTCCTCTTCACGGCtgGaTTGCTGCAGAGGTTcgACCTGGAGcTCCCaGATGATGGgCAGCTaCCCTgtCTcGtGGGCaACCCCAgtttGGTCcTgCaGATaGAtcCTTTCAAAGTGAAGATcAAgGaGCGCCAGGCCTGGAaGGAaGCCCAcaCTGAGGGgAGtACCTTTCTcCAGAAGGCCCCTGAGCAGCTCATt

>CYP17A1_elephant

GCCAtcATGTGGGAGCTCTTGGCTgTCtTGCTGcTcaCCcTAGttTATTaCTTTtGGCCCAAGaTaAgGcGCCCTGGTGCCAAGTACCCCAAGAGCCTCCCgTtCCTGCCCcTGGTGGGCAGCCTGCCtTTCCTcCCtcGACATGGaCATCCtCAcaTcAAtTTCTTCAAGCTGCAGAAAAAATAtGGCCCCATCTATTCtTTtCGtcTGGGTACCgcaACTtatGTGgTtGTCGGCCAgTACCAgCTGGCCAgGGAGGTGCTcATCAAGAAaGGCAgaGAATTCTCCGGGCGaCCCaAtGTGAACTCTggcaATggTGTCAaACAAcCAAAAaGGcGTCGCCTTtGCCGACagTGGTcCCCaCTGGCAGCTaCATaGaAAGCTGGTGCaGGCTGCgTTTaCCCTGTTCAAGGACGGCcACCAGAgGCTaGAGAAGATcATCCTGGAgAatCTGGAcAcccACAgTcTGGTgGAtATcTTCCCCtgcTTGAAGAGGAGAAaTTCAgCAGTGACTCtATCACTAaCcTGCTaGACaTACTGATCCAAGCCAAGAtGAgCTCAGACAATAACAAtgaTGGCCCAGACCAGGATTCAAAGaTGCTTTCtcAagAACACATTaTTGCCACCATgatGGACATCTTCGGGGCtGGTGTGGAGACCACtACCTCTGTGaTGAAGTGGAcTGTGGCCTTCCTGCTGCACaACCCTCAGgTGAAGAAaAAGATCCAGGAGGAGATTGACCAGAAcaTaGGTTTtAGtCGCACACCAACtCTgAGTGACCGGAACCaCCTCgTTCTGCTGGAGGCCACCATCCGAGAgGTGCTaCGcATCCGaCCTaTaGCtCCccTcCTCAcCCCCCAcAtGGCTtccacTGACTCCAGCATtGGtGAGTTTaCcaTTGACaAaGGCACAAAtGTtATCATCAATTTGTGGGCACTgCATCACAgTGAGAAGGAGTGGCACCAGCCgGACCaGTTCATGCCTGAGCGCTTCTTGGACCCAACcGGGtGCCAGCTCATCTCACCaaCaTTgAGCTACTTGCCCTTTGGgGCgGGaCCCCGCTCcTGCcTAGGTGAGaTCCTGGCCCGCCAGGAGCTCTTCCTCTTCctGtCCTGGgTGCTcCAGAGGTTTgACCTGGAGGTgCCGGATGATGGgCAGCTaCCCTCCtTGGAGGGCaAtCCCAagGCGGTCTTTCTGATCagCaacTTCAAAGTGAAGgTcAAgGTGCGCgAGGCCTGGAGGGAaGCCCAtGCTGAGGGtAGtgCCTAGAGGCTGTgCCaAGCcCCa

>CYP17A1_human

GCCACcATGTGGGAGCTCgTGGCTcTCtTGCTGcTtaCCcTAGCtTATTTgTTTtGGCCCAAGagaAgGTGCCCTGGTGCCAAGTACCCCAAGAGCCTCCtgTcCCTGCCCcTGGTGGGCAGCCTGCCaTTCCTcCCCAGACAcGGCCATatGCATaacAACTTCTTCAAGCTGCAGAAAAAATAtGGCCCCATCTATTCggTtCGtATGGGcACCAAGACTACaGTGATtGTCGGCCACcACCAgCTGGCCAAGGAGGTGCTTATtAAGAAGGGCAAGGAcTTCTCtGGGCGGCCtCAAaTGGCAACTCTAgACATCgcGTCcaACAAcCgtAAGGGTaTCGCCTTCGCtGACtcTGGcGCaCaCTGGCAGCTGCATCGaAgGCTGGcGatGGCcaCcTTTGCCCTGTTCAAGGAtGGCgAtCAGAAGCTGGAGAAGATcAGGAGAAaTTCcggAGTGACTCtATCACcAaCATGCTGGACacACTGATgCAAGCCAAGAtGAACTCAGAtAATggCAAtgCTGGCCCAGAtCAaGAcTCAgAGCTGCTTTCaGATAAcCACATTCTcaCCACCATAGGGGACATCTTtGGGGCtGGcGTGGAGACCACCACCTCTGTGGTtAAaTGGAcccTGGCCTTCCTGCTGCACaAtCCTCAGgTGAAGAAGAAGcTCtAcGAGGAGATTGACCAGAATGTGGGTTTCAGCCGCACACCAACtaTCAGTGACCGtAACCGtCTCCTcCTGCTGGAGGCCACCATCCGAGAgGTGCTTCGccTCaGGCCcGTGGCCCCTATGCTCATCCCCCAcAAGGCcaacGTTGACTCCAGCATCGGtGAGTTTGCTGTgGACaAGGGCACAgAaGTtATCATCAATcTGTGGGCgCTgCATCACAATGAGAAGGAGTGGCACCAGCCgGAtCaGTTCATGCCTGAGCGtTTCTTGaAtCCAgCGGGGAcCCAGCTCATCTCACCGTCagTAAGCTAtTTGCCCTTcGGAGCAGGaCCtCGCTCcTGtATAGGTGAGaTCCTGGCCCGCCAGGAGCTCTTCCTCaTCAtGGCCTGGcTGCTGCAGAGGTTcgACCTGGAGGTgCCaGATGATGGgCAGCTGCCCTCCCTGGAaGGCatCCCCAagGtGGTCTTTCTGATCGACTCTTTCAAAGTGAAGATcAAgGTGCGCCAGGCCTGGAGGGAaGCCCAGGCTGAGGGtAGCACCTAaAGGCTGTAaCTcCCCACTCTGCCTTCTTTTtCAGCtTGtgGCaaTGcCAGTGATGTGcATAAAC

>CYP17A1_sheep

GGaCACAATGTGGGtGCTCTTGGCTgTCtTtCTGcTcaCCcTcGCCTATTTaTTTtGGCCCAAGaccAAGcaCtCTGGTGCCAAGTACCCCAgGAGCCTCCCATcCCTGCCCcTGGTGGGCAGCCTGCCGTTCCTcCCCAGACgTGGCCAgCaaCAcGaGAACTTCTTCAAGCTGCAGgAAAAATAtGGCCCCATCTATTCCTTtCGttTGGGTtCCAAGACTACtGTGATGaTtGGaCACcACCAgtTGGCCAgGGAGGTGCTTcTCAAGAAGGGCAAGGAATTCTCtGGGCGtCCCaAAGTGGCcACTCTAgACATCCTGTCAGACAAcCAAAAGGGcaTtGCCTTtGCCGACCATGGTGCCCaCTGGCAGCTGCATCGGAAGCTGGTaCtGaaTGCcTTTGCCCTGTTCAAGGAtGGCAACCtGAAGtTaGAGAAGATcTTGcAGATTTTCCCCAgCAAAgCCaTGGAAAAgATGAaGggTTGTGTTgAaAcgCGAAATGAAtTGCTGAGTgAAATCcTTGAAAAAtgTcAGGAGAACTTCAcCAGcGACTCCATCACTAaCtTGCTGcACaTACTGATgCAAGCCAAGgtGAAtgCAGACAATAACAACACTGGCCCAGAgCAGGATTCAAAGCTGCTTTCaaAcAgACACATgCTcGCtACCATAGcGGACATCTTCGGGGCtGGTGTGGAGACCACCACCTCTGTGaTaAAGTGGATcGTGGCCTaCCTGCTaCACcACCCTCAGtTGAAGAAGAgGATCCAGGAtagcATTGACCAGAATaTaGGTTTCAatCGCACcCCAACcaTCAGTGACCGGAACCGCCTtgTcCTGCTGGAGGCgACCATCCGAGAgGTGCTcCGaATCCGGCCTGTGGCCCCTATGCTgATCCCCCAcAAGGCTaTcaTTGACTCCAGCATtGGCGAccTTaCcaTTGACaAGGGCACAgACGTtgTggTCAAccTGTGGGCACTgCATCACAATGAGAAGGAGTGGCAgCAGCCcGACCtGTTCATGCCcGAGCGCTTCTTGGACCCcACGGGGAcaCAaCTCATCTCgCCaTCGTTAAGCTACTTGCCCTTTGGAGCcGGaCCCCGCTCcTGtgTAGGTGAGaTgCTaGCCCGCCAGGAGCTCTTCCTCTTCAtGtCCcGGcTGCTGCAGAGGTTcAACCTGGAGaTCCCGGATGATGGgaAGCTaCCCTCtCTGGAGGGCaAtCCCAgtctcGTCTTgCaGATCaAacCTTTCAAgGTGAAGATcgAgGTGCGCCAGGCCTGGAaGGAaGCCCAGGCTGAGGGtAGCACCTcGACTCCACCCTaTGTGACCCtTctTCctccccGCCCtgTCTGCCTTCTTTcCCAGCCTGtAGCCCTGGtAGTGATGTGcATAAA

>CYP17A1_mouse

CTacacCTGGCtgCcATGTGGGAaCTtgTGGgTcTCtTGCTGcTcatCcTgGCCTATTTCTTTtGGCCCAAGtcaAAGacaCCTaaTGCCAAGTtCCCCAgGAGCCTtCCATtCCTGCCCcTGGTGGGtAGtCTaCCGTTtCTcCCCAGACgTGGtCATatGCATGccAACTTCTTCAAGCTGCAGgAAAAgTAtGGtCCCATCTATTCtcTtCGCcTGGGTACCAcaACTgCaGTGATtGTCGGtCACTAtCAgCTGGCCAgaGAaGTGCTcgTgAAGAAGGGgAAaGAATTCTCtGGtCGGCCCCAgACTCTAggCcTCtTGTCgGACcAaggAAAaGGcGTCGCCTTtGCgGAtagTaGTagCtCCTGGCAGCTGCAcCGGAAGCTGGTattcagcaCcTTTtCCCTGTTCAgGGAtGAGAAaTTCAAtAGTGAaTCtcTCtCcAGCcTGacaGACaTtCTGATaCAAGCCAAGAtGAAtgCAGAaAATAAtAACACTGGggaAGgCCAGGAccCAAgtgTGtTcTCaGATAAgCAtATcCTTGtCACggTgGGaGACATCTTtGGGGCaGGcaTaGAGACaACtAgCTCTGTGcTGAAcTGGATccTGGCtTTCCTGgTGCACaACCCTCAGgTGAAGAgGAAGATCCAaaAGGAGATTGACCAGtATGTaGGcTTCAGtCGaACACCgtCttTCAaTGACCGGActCaCCTCCTcaTGCTGGAGGCCACtATCCGAGAAGTGCTTCGTATCaGGCCgGTGGCCCCctTGCTCATCCCaCAcAAGGCTaacaTTGACTCCAGCATtGGaGAGTTTGCcaTcccgaAGGaCACAcAtGTgATCATCAATcTcTGGGCACTgCATCACgATaAaAAtGAaTGGgACCAGCCaGAtCGGTTtATGCCTGAGCGCTTCTTaGAtCCAACaGGaAGCCAtCTCATtaCACCcaCacccAGtTAtTTGCCCTTcGGAGCtGGTCCCCGaTCgTGCATtGGaGAGgctCTGGCCCGgCAGGAGCTCTTtaTCTTCAtGGCCTtGcTGCTGCAGAGGTTTgACtTtGAtGTgtCaGATGAcaaaCAGCTGCCCTgtCTGGtGGGtgACCCCAagGtGGTCTTTCTGATCGACcCTTTCAAAGTGAAaATcAcaGTGCGaCAaGCaTGGAaGGAtGCaCAGGtTGAGGttAGCACCTAGAGG

>CYP19A1_horse

AAAGCCACCCGGTTCCTAACAGCCGTGCATCATTAGCAAAACTCATCATCTTCAAGAGTCCGGAAACTAGAAGTGACCAGCAGACTCAGGCCTTTACATTGCTTCGCCTGAGATCAAGGAGCACAAGATGATTTTGGAAATGCTAAACCCGATGCATTATAACCTCACCAGCATGGTGCCCGAAGTCATGCCTGTCGCCACCTTGCCCATTCTGCTGCTCACTGGCTTTCTTTTCTTTGTTTGGAATCATGAAGAAACATCCTCAATACCAGGCCCTGGCTATTGCATGGGAATCGGGCCCCTCATTTCCCACCTCCGGTTCCTGTGGATGGGGCTTGGCAGTGCCTGCAACTACTACAACAAGATGTATGGAGAATTCGTGAGAGTCTGGATCAGTGGAGAGGAAACGCTCGTTATTAGCAAGTCCTCAAGTACCTTCCACATCATGAAACACGATCACTACTCCTCCCGATTTGGCAGCACATTTGGGTTGCAGTATATGGGCATGCATGAGAATGGCGTCATATTTAACAATAACCCAGCCGTCTGGAAAGCTTTGCGACCTTTCTTTGTAAAAGCTTTGTCTGGCCCCAGCCTTGCGCGCATGGTGACAGTTTGTGTTGAATCCGTCAACAACCATCTGGACAGGTTGGACGAGGTCACCAATGCGTTGGGCCATGTCAACGTGTTGACCCTCATGCGACGTACCATGCTGGACGCTTCCAACACCCTCTTCCTGAGGATCCCCTTGGACGAGAAAAACATCGTGCTTAAAATCCAGGGTTATTTTGATGCATGGCAGGCTCTCCTTATCAAACCAAACATCTTCTTTAAGATTTCTTGGCTATCCAGAAAGCATCAAAAGTCCATCAAAGAATTGAGAGATGCCGTGGGAATTCTAGCAGAAGAAAAAAGACACAGGATTTTCACAGCAGAGAAACTGGAAGACCATGTGGATTTTGCCACTGATCTAATTTTGGCTGAGAAACGTGGTGAGCTGACCAAAGAGAATGTGAACCAGTGCATATTGGAAATGATGATTGCAGCGCCAGACACCTTGTCTGTCACTGTGTTCTTCATGCTATGTCTCATTGCGCAGCACCCTAAAGTTGAAGAGGCACTCATGAAGGAAATCCAGACTGTTCTTGGTGAAAGAGACTTAAAGAATGATGATATGCAAAAATTAAAAGTGATGGAAAATTTTATTAATGAGAGCATGCGGTACCAGCCTGTCGTGGACATTGTCATGCGCAAAGCCTTAGAGGATGATGTCATCGATGGCTATCCAGTGAAAAAGGGGACTAACATTATTCTGAATATTGGAAGAATGCATAAACTCGAGTTTTTCCCCAAGCCTAATGAATTTACTCTTGAAAACTTTGAGAAGAATGTTCCTTACAGGTATTTTCAGCCATTTGGTTTTGGGCCCCGTAGCTGCGCTGGAAAGTTCATCGCCATGGTGATGATGAAGGTGATGCTGGTTTCACTTCTGAGACGATTCCATGTGAAGACATTACAAGGAAACTGTCTTGAAAATATGCAGAAAACAAATGACTTGGCCCTCCACCCGGATGAGTCTAGAAGCTTACCGGCAATGATTTTTACTCCAAGAAATTCAGAAAAGTGCCTCGAACACTAAAAAAGTTTGGTCAGTACCTATTCCAGAGCATTTCTCATCAGTTATTCATAAGGAAACCATCCATCTTTGCCAGGTAGTGTCATCCTCATAGTAAACATTCGGTGGCCTGTGGCATTTTATAGGCATGCATCCTATGGGTTGTATGCAAGCCAGGAAACATTGGTCATCTGATCTTGTCCAAACCAGAGAACCAAACTGCAAGAGAAAATGCAGAGGCCAAGAGTTTGTGGGGGAAATGGTCAGTGAAGAGAATGCAGCCCTAAAGGCCCCATTCCACAAAATGTGCTTTGGCAAAGATAGGCCATCAGCAAAATTTATGTCCCATTTGCCACAGGATGTTCACTGCTCTGCCCCCAGAGCATTTTTATGTCTGGGGCAGAAACACTTCTAAAGAGTGCTCCTCCCATCCCATTGTCCATCTTCCTTGACATTTTCCCCCTCTCTTTCCTTCCATGACACCAAAAGCCAAGTTGATTAGAAAGACCAGGCCGATATCTGGGTACCTAGAGCCAAACAAACATGTTAGTGTCAATAAAGGTGCTTTGATTTGGTTTTTGGTGGGGTTGGCCACTGCAGCATTCATAGTCTTTGGAGAAATGCTTACAGATTCAGCATTCGACTTTTCCTGTGAATTATAATCCATTAACTCTTGTTTATTATGTGATTTGTCTGTGGCAAAAGTAAACTGGAGACTATCCTTTCCCAGTCTCTCAGTTCATGCCTCAGCCACTTACCTCTGATTCAGGCATGATTCAGATAATCAAGGTAACTTAGCAATAGCTTGAGTAAATAGAGTTAGGCCCACATGTCTGCTGTAGGAAAAAAACTCACACAATGCATTTCAAATTCAAATGAAAATTCGTAGGGGGAGGGGGATGGGAGGGGGGAGGGGTACAGGGGCACACATGCATGGTGACGGATGGAAACTAGACTTTTAGTGGTGAACGCGATGTAGTAGTCTATACAGAAGGCAAAATATAATGATGTACACCTGAAATTTACACAATCTTATGAACCAATGTTACCTAAATAAATAAAGTGATTTAATAAaaaaaaaaaaaaaaaa

>CYP19A1_rhino

AAAGCCACCCGGTTCCTAACAGCCGcGCATCATTAGCAAAACTCAcCATCTTCAAGAGTCCaaAAAgTAGAAGTGACCAGCAGACcCAGGaCTTTAaAcTGCTctGCCTGAGATCAAGGAGaACAAGATGgTTTTGGAAATGCTgAACCCaAcGCgTTATAACCTCACCAGCATGGTGCCCGAAGTCATGCCTGTtGCCACCTTGCCaATcCTGCTGCTCACTGGCTTTCTTcTCTTgGTTTGGAATCATGAgGAAACATCCTCAATACCAGGtCCTGGCTAcTGtATGGGgATtGGGCCCCTCATcTCCCACtTCaGGTTCCTGTGGATGGGGaTTGGCAGcGCCTGCAAtTACTACAACAAGATGTATGGAGAATTCaTGAGAGTCTGGATaAGTGGAGAGGAAACaCTCaTTATTAGCAAGTCaTCAAGTACgTTCCACgTCATGAAACACGATCACTACgttgCCCGATTcGGCAGCAaAcTcGGGTTGCAGTgcgTGGGCATGCATGAGAATGGCaTCATATTTAACAgTAACaCAGCCcTCTGGAAAGCTgTtCGACCTTTCTTTtTAAAAGCTTTGagcGGCCCtgGCCTTGCatGCATGGTGACAGTTTGTGTTGAggCCaTgAcgAgaCATCTGGACAGGTTGGAgGAGGTCACCAATGaGTTGGGCtATGTCgAtGTGTTGACCCTCATGCGACGTAtCATGCTGGACGCcTCCAACtCaCTCTTCCTGAGGATCCCCTTGGACGAaAAAAACcTCGTGgTTAAAATCCAGGGTTATTTTGATGCgTGGCAGGCTCTCCTTcTCAAACCAgACATCTTCTTTAAGATTTCTTGGCTgTaCAGAAAGtATgAAAAGTCCAgCAAgGAtTTGAaAGATtCtcTGGaAgTTCTgatAGAAGAAAAAAGAtACAGGATTTctACAGCAGAGAAACTGGAAGACCATaTGGATTTTGCCACTGAgCTgATTTTGGCcGAGAAACGTGGcGAcCTGACaAgAGAGAATGTGAACCAGTGCATATTGGAAATGcTGATcGCAGCGCCAGACACCaTGTCTGTCtCTGTcTTCTTCATGCTATtTCTCgTTGCcCAGCACCCTAAgGTTGAAGAGGCACTCATGAAGGAAATCCAGACTGTTgTTGGTGAAAGAGACaTAAgGAtTGATGATATGCAAAAATTAAAAGTGgTGGAAAAcTgTATTtATGAGAGCATGCGGTACCAGCCTGTCGTGGACATTGTCATGCGCAAAGCCTTgGAGGATGATGTCATtGAcGGCTAcCCAGTGAAAAAGGGGACTAACATTATcCTGAATATTGGAAGAATGCATAAACTCGAGTTTTTCCCCAAGCCcAATGAATTTACTCTTGAAAACTTTGAGAAGAATGTTCCTTACAGGTATTTTCAGCCATTTGGTTTTGGGCCCCGTgcCTGtGCaGGAAAGTaCATCGCCATGGTGATGATGAAGGTGATcCTGGTTaCACTTCTGAGACGATTCCgTGTGcAGACgTTACAAGGAgggaGTaTTGAAAATATGCAGAAgAaAAAccACTTGtCCtTgCACCCaGATGAGaCTAGcAGCTTgCtGGaAATGATTTTcAtcCCAAGAAATTCAGAcAAGaGCCTCaAACACTAAAtAAGTTTGGTCAGTACCTAcTCtgGAGCATTTCTCATCAGTagTTCAcAtGcAAACtATCCATCTTTGCCAGGTAGTGTCATtCTCAcAGTgAACATTCaGTGGCCTGTGGCATTTTATAGGCATaCcTCCTATGGGcTGTcaGCAAGCCAGGAgACATcaGTCATCTGATCTTGTCCAAACCAGAGAACCAgACTGCAAaAGAAAATGCAGAGGCCAAGAGTTTGTGGGGGAAATGGTCAGTGAAGAaAATGCAGCCgTAAAGGCCCaATTCCACAAAATaTGCTTTGGaAAAGATAGGCCATCgGCAAAAcTTATGTCCtgcTTGCCACAtGATcTTCACTGCaCTGCCCCaAGAGtATTTTaATGTCTGGGGCAGAAgCACTcAAGTTtATTAGAAAGgCCAGGCtGATgTCTGGGTACCTAGAGCCAAACAcACcTGcTAGTGcaAATAAAGGTGCTTTcATTTGGTTTTcaGTGGGcTTGGCCACTGCAaCtTTCgTAGTCTTTGGAGAAATGCTTACAaATTCAGCATTtGACTTTTCCTaTGAATTATAATCatTTAtCTCTTGTTTATGGGGTAaAGGGGCAaAtATGCATGGTGACaGATGGAAACTAGACTTTTAGTGGTGAACaCGATGTAGTAaTCTgTgCAGAAatagAAgTAcAATGATGTACACCTGAAATTTAtACAATgcTATaAACCAAaGTTACtgcAATAAAc

>CYP19A1_cow

ATCATTAGCAAAACTCAcCATCTTCAAGAGTCCAAACTAGAAGTGACCAGCAGACcCAGGaCTTTAaATTaCTTCcCCTGAGATCAAGtAaaACAAaATGcTTTTGGAAgTGCTgAACCCaAgGCATTAcAACgTCACCAGCATGGTGtCCGAAGTtgTGCCTaTtGCCAgCaTtgCaATcCTGCTGCTCACTGGaTTTCTTcTCTTgGTTTGGAATtATGAgGAcACATCCTCAATACCAGGtCCcaGCTAcTttcTGGGAATtGGGCCCCTCATTTCCCACtgCaGGTTCCTcTGGATGGGGaTcGGCAGTGCCTGCAAtTACTACAACAAGATGTATGGAGAATTCaTGAGAGTCTGGgTatGTGGAGAGGAAACcCTtaTTATTAGCAAGTCCTCAAGTAtgTTCCAtgTaATGAAgCACagTCACTACataTCCCGATTTGGCAGtAaAcTTGGGTTGCAGTtcATcGGCATGCAcGAGAAaGGCaTCATATTTAACAATAAtCCAGCacTCTGGAAAGCTgTtCGACCTTTCTTTacAAAAGCTTTGTCcGGCCCtgGCCTgGtGCGCATGGTGACcaTcTGTGcTGAtTCCaTCAcCAAgCATCTGGACAGGcTGGAgGAGGTCtgCAATGacTTGGGCtATGTggACGTGTTGACCCTCATGCGgCGcAtCATGCTGGACaCcTCtAACAtgCTCTTCCTGgGGATCCCCTTGGAtATCGTGgTTAAAATCCAGGGgTATTTTGATGCATGGCAaGCTCTCCTTcTCAAACCAgACATCTTCTTTAAGATTTCTTGGCTgTgCAGAAAGtATgAAAAGTCtATCAAgGAcTTGAaAGATGCCaTGGaAATTCTcatAGAAGAAAAAAGACACAGGATTTcaACAGCAGAGAAgCTGGAAGACagcaTaGATTTcGCCACTGAgtTgATTTTtGCTGAGAAACGTGGTGAaCTtACaAgAGAGAATGTaAACCAGTGCATATTGGAAATGcTGATcGCAGCGCCAGACACCaTGTCTGTttCTGTGTTCTTCATGCTgTtTCTtATTGCaaAGCAtCCccAgGTTGAAGAGGCAaTaATaAgGGAAATCCAGACTGTTgTTGGTGAAAGAGACaTAAgGAtTGATGATATGCAAAAgcTAAAAGTGgTGGAAAAcTTTATTAATGAGAGCATGCGGTACCAGCCcGTtGTGGACcTgGTCATGCGCAAAGCCTTAGAGGATGATGTCATCGATGGCTAcCCgGTGAAAAAGGGGACTAACATTATcCTGAATcTTGGAAGAATGCATAgACTCGAGTTTTTCCCaAAGCCTAATGAgTTTACTCTTGAAAACTTTGccAAGAATGTTCCTTACAGGTAcTTTCAGCCATTTGGcTTTGGGCCCCGggcCTGtGCgGGAAAGTaCATCGCCATGGTGATGATGAAGGTcgTcCTGGTcaCcCTTCTGAGACGcTTCCAcGTGcAGACtTTgCAAGGtcggTGcgTTGAgAAgATGCAGAAgAaAAATGACTTatCCtTgCAtCCaGAcGAGaCcAGAgaCcggCtaGaAATGATTTTcACcCCAAGAAATTCAGAcAAGTGCCTCGAgCgCTAAAgAAGTTTGGTCAGTcCCTgccCCAGAGCAcTgCTCAaCAGaacTcCAcAtGGgAACCAcCCATCTTTGCCAGGTAGTTGGCCTGTGcCATTTTATAGGCtTaCcTCCTgTGGGTTGTCAgACTGCAAGAGAAAATAGAGGCCAAGAGTTTGTTGCAGCCCTAAAGaCCCaATTCCACAAAAcaTGCTTGCtTTTTaAcaTCTGGGGCAGAAgCACTTcTtCCCtctTCTTTCtTTCCATGACcCtAAgTTTcATTTGcTTTTTGGTGGGGTgGGgTTGGAGAAATGCTTACAaATTCAGCATTtGACTTTTCCTacaAAcTAcAtTCaATTAACTCTTGTTTAT

>CYP19A1_dog

AAAGCCACCCGGTTCtTAACAGCaGcGCATCATTAGCAAAACTCAcCATCTTCAAGAGTCCaaAAACTAGAAGTGACCAGCAGACcCAGGtATGgTgTTGGAAATGCTgAACCCaATGCATTAcAACaTCACCAGCATGaTGCCCGAAGTCATGCCTGTgGCCACCaTGCCaATcCTGCTGCTCACaGGtTTTCTTcTtTTgGTTTGGAATtATGAAGAcACATCCTCAATACCAGGtCCTGGCTATTGtATGGGAATtGGGCCCCTaATTTCCCACtgCaGGTTCCTaTGGATGGGGaTTGGCAGTGCtTGCAACTACTACAACAAGATGTAcGGAGAATTCaTGAGAGTCTGGATatGTGGAGAGGAAACaCTCaTTATTAGCAAGTCCTCtAGcAtgTTCCACATaATGAAgCACagTCACTACagCTCCCGATTTGGCAGCAaAcTTGGaTTGCAGTgcATtGGCATGCATGAaAAcGGCaTCATATTTAACAAcAAtCCAaCacTCTGGAAAGCTaTtCGACCcTTCTTTacAAAAGCTTTGTCTGGCCCCgGCCTcGtGCGtATGGTaACAGTTTGTGTTGgATCCaTCAtaAcgCATCTGGACAGGTTGGAgGAaGTCAgCAAcGaacTGGGttATGTCgACGTGTTGACtCTCATGCGgCGcAtCATGCTGGACaCcTCtAACAttCTCTTCCTGgGGATCCCCTTGGACATtGTGgTTAAAATCCAGGGTTATTTTGATGCgTGGCAaGCTCTCCTTcTCAAACCAgACATCTTCTTTAAGATTTCTTGGCTATaCAaAAAGtATgAAAAGTCtATCAAgGAtTTaAaAGATGCCaTGGaAATTCTgatAGAAGAAAAAAGACACAGaATTTcCACAGCAGAGAAACTGGAAGACCATaTGGATTTTGCCACTGAatTgATTTTtGCTGAGAAACGTGGTGActTaACaAgAGAGAATGTGAACCAGTGCATAcTGGAAATGcTGATcGCAGCaCCAGACACCaTGTCTGTCtCTGTGTTCTTtATGCTATtTCTCATTGCaaAGCACCCTAAgGTTGAAGAatCAaTaATGAAGGAgATCCAGgCTGTTgTaGGTGAAAGAGAtaTAAgGAtTGATGATATGCAAAAATTAAAAGTGgTGGAAAAcTTTATctAcGAGAGCATGCGGTACCAGCCTGTtGTGaACtTgGTCATGCGCAAAGCCTTAcAGGATGATaTCATtGATGGCTAcCtAGTGAAAAAGGGGACTAACATTATcCTGAATATTGGAAGAATGCATAgACTgGAGTTTTTCCCCAAGCCcAATGAATTTACTCTTGAAAACTTTGcaAAGAATGTTCCTTACAGGTATTTTCAGCCATTTGGcTTTGGaCCtCGTAGCTGtGCaGGAAAGTaCATtGCCATGGTGATGATGAAaGTtgTcCTGGTTaCACTTCTGAGACGtTTCCAcGTGcAGACATTgCAAGGAgAgTGTaTTGAAAATATGCAGAAAAaAtATGgCTTGtCCtTaCACCCaGATGAGaCTAacAaCTTgCtGGaAATGgTTTTTgtcCCAAGAAATTCAGAAAAGTGCCTCaAACACTAAAgAAGgTTGaTCAGTAtCTAcTCtATCAGTagTTCAcAtGGAAACtgcCtgTCTTTaCCAaGTAGcGTCATCCTCgcAGTgAACATTtGGTGGCCTaTGcCATTTTATAGcCATaCcTCaTATGGacTGTcaGCAAGtCAGGAgACATTGGTCATCTGtTtTTGTCCAAACCAGAGAACttgtCTttcAGAGAAAATATTCCACAAAAcaTGCTTTGGaAAAGtTAGGCCATCAGCAAAATGTCTGGGCTaCAaCATTCATAtTCTTTGGAGAAATGCTTAtAaATTCAGCATTtGACTTTTtCTa

>CYP19A1_pig

AGGtCCTGGCTATTttcTGGGAATtGGGCCCCTCATTTCCCACtTCaGGTTCCTcTGGATGGGGaTTGGCAGTGCCTGCAACTACTACAACAAaATGTATGGAGAATTCaTGAGgGTCTGGATagGTGGAGAGGAAACaCTCaTTATTAGCAAGTCCTCAAGTgtgTTCCAtgTaATGAAgCACagTCACTACaCaTCCCGATTcGGCAGCAaAccTGGGTTGgAGTgcATcGGCATGtATGAGAAaGGCaTCATATTTAAtAATgAtCCAGCCcTCTGGAAAGCcgTtaGAaCTTaCTTTaTgAAAGCTcTGTCcGGCCCtgGCCTgGtGCGCATGGTGACcGTcTGTGccGAtTCCaTCAcCAAgCAcCTGGACAaGcTGGAgGAGGTCcgCAATGacTTGGGCtAcGTggACGTGTTGACCCTCATGCGgCGcAtCATGCTGGACaCcTCtAACAaCCTCTTCCTGgGGATCCCgTTGGAtGAaAAggcCATtGTGtgTAAAATCCAGGGTTATTTTGATGCATGGCAaGCTCTCCTTcTCAAACCAgAatTCTTtTTTAAGtTTTCaTGGCTgTaCAaAAAGCATaAAgAGTGAaAGAgaaCaTGGaAATTCTgatAGAgaAAAAAAGAtgCAGcATTaTCACAGCAGAGAAACTGGAAGACtgcaTGGATTTcGCCACTGAgtTgATTTTGGCcGAGAAACGTGGcGAatTaACaAAgGAGAATGTGAACCAGTGCATATTGGAAATGcTGATTGCAGCaCCAGACACCcTGTCTGTCACTGTGTTtTTCATGCTgTtTCTCATTGCaaAGCACCCccAgGTTGAAGAGGCAaTagTGAAGGAAATCCAGACTGTTaTTGGTGAAAGAGACaTAAgGAATGATGAcATGCAAAAAcTcAAAGTGgTGGAAAAcTTTATTtATGAGAGCATGaGGTACCAGCCTGTCGTGGACcTcGTCATGCGaAAAGCCTTAGAGGATGATGTCATCGATGGCTAcCCgGTGAAAAAGGGaACcAACATTATcCTGAATATTGGAAGAATGCATAgACTCGAGTTTTTCCCCAAGCCcAATGAATTTACTCTTGAgAACTTTGccAAGAATGTTCCTTACAGGTAcTTcCAGCCATTTGGcTTTGGGCCCCGggcCTGtGCgGGAAAGTatATCGCCATGGTcATGATGAAGGTcActCTGGTcatACTTCTGAGACGcTTCCAaGTGcAGACAccgCAAGaccggTGTgTTGAAAAgATGCAGAAgAaAAATGAtTTatCCtTgCACCCGGATGAGaCcAGcgGCcTgCtGGaAATGATTTTcAtcCCAAGAAATTCAGAcAAGaGCCTCGAgCACTAAAgAgGTTTGGTCAGTTCAcAtGGAAACCAcCCATCTTTGGCCCTAAAGttCCaATTaCAtAAAAcaTGtTTTGGaAAAGAcAaGCCAcCAGCATTTTaATGTCTGGGGCAGAAgCACGCtAAACAAACccacTcGTGTgAATAAAaGaGtTTTGATTTGGTgTTTaGTGGGAAgtTAAAtgaaAGACTATCCTTTCCCAGTCTCTCAaTTtATGCCTCAGCCA

>CYP19A1_elephant

AAACTCAcCATCTTCAAGAGTCCaaAAACTAGAAGTGACCAGCAGACcCAGGaCTTTAagTTGCTTCcCgTGAGgTCAAaGAaagCgAGATGATTTTGGAAATGCTgAACCCaATGCAcTATAACaTCACCAGtgTGGTGCCtGAgaTCATGCCTGTtGCCACCaTGCCaATcCTGCTGCTCACTGGCTTTCTTcTCcTgGTcTGGAATtATGAgGgcACATCtTCAATtCCAGGtCCTGGCTATTGtATGGGAATtGGcCCCCTCATTTCaCAtggCaGaTTCCTGTGGATGGGGaTTGGCAGTGCCTGCAACTACTACAACAAGATGTATGGAGAATTCaTGAGAGTCTGGATatGTGGAGAGGAgACaCTCaTcATTAGCAAGTCCTCAAGTAtgTTCCAtgTaATGAAgCACagTCACTACatCTCCCGATTTGGCAGCAaAcTTGGGTTGCAGTgTATtGGCATGCATGAaAAcGGCaTCATATTTAACAATAAtCCAGCCcTCTGGAAAGtgaTtCGACCTTTCTTTaTgAAAGCTTTGTCTGGCCCtgGCCTTatGCGgATGGTGgCgaTTTGTGTTGAATCCGTCAAgAcgCATCTGGACAGGcTGGAgGAGGTCACCAATGCaTcaGGCtATGTagACGTGcTGACtCTCATGCGACGcAtCATGCTGGACaCcTCCAACAtgCTCTTttTGgGtATCCCCTTGGACGATtGTGgTTAAAATCCAGGGTTATTTTGATGCgTGGCAaGCTCTCCTTcTCAAACCAgACATCTTCTTTAAGATTTCTTGGCTcTaCAaAAAGtATgAAAAGTCCAgCAAgGAtTTGAaAGATaCtaTaGagATTCTAattGAAGAAAAgAGACACAGGATTTctgagGCAGAGAAACTGGAAGACaATaTGGATTTTGCCACTcAgtTgATTTTtGCTGAGAAACGTGGTGAcCTGACgAgAGAGAATGTGAACCAGTGCATATTGGAAATGcTaATTGCAGCaCCtGACACCaTGTCcGTCtCTGTGTTCTTCATGCTATtTCTCATaGCaaAGCACCCTAAgGTTGAAGAGGCAtTaATGAAaGAAATtCAGACTtTTtTTGGTGAAAGAGACaTAAgGAtTGATGATgTaCcAAAATTAAAAGTGgTGGAAAAcTTcATTtATGAGAGCATGaGGTACCAGCCaGTtGTGGACtTgGTCATGaGaAAAGCtTTAcAaGATGAcaTCATtGATGGaTAcCCAGTGAAAAAGGGGACcAAtATTATTCTGAATATTGGAAGAATGCATAgACTCGAGTTTTTCCCgAAGCCcAATGAATTTACTCTTGAAAACTTTGAGAAGAATGTTCCTTACAGGTATTTTCAGCCATTTGGTTTTGGGCCCCGTAGtTGtGCaGGcAAGTaCATCGCCATGGTGATGATGAAaGccATcCTGGTTaCACTTCTGAGACGgTTCCAcGTGAAGACAcTgCAAGGAgggTGTaTTGAAAAcATGCAGAAAAaAAATGACTTGtCCtTgCACCCaGATGAGaCTgGtAaaTTtCtGGaAATGATTTTTACcCCAAGAAATTttGAcAAGTaCCTgaAACACTAAAgAAGgcTGGTCAGTACCTAcTCtgaAGCATTTCTCAaCAGTagTTCAcAcGGtAgCCATCCATCTTgcCCAGGTAGTGTCAaCtTtgcAGTgAACATTCGGTGGCATTTTATAGGCATaCtTCCTActaGcTGTcaGCAAaCCAGGAActATcGGTCATCcaATCTTGcCtAAACaAGgGAAtCAggCTGCgAGAGAAAAgaCAGAGGCCAAaAGTTTGccGGGGAAATGGTCAGTGgAcAaAccGtAaCCCTAAAGaCCCaATTCCACAAAAATGTCTGGGGCCAAACAcACcTGcTAGTGTggATAAAaGTGCTTTGATTTGGTTTTATTCATAGTCTTTGGAGAAATGCgTACAaAaatAGCATTtGACTTTTCAACTCTTGTTTATTCTCAGTTCATtCCTCAGCCACTTACCTCTGTAGGCCCAtATGTCTGCTGTAGGgAAgAAAaTCACAtgATGCATTTtAAATTCAAATaAAAAT

>CYP19A1_human

AACAGCCGcGCATCATTAGCAAAACTCAcCATCTTCAAGAGTCCaaAAACTAGAAGTGACCAGCAGACcCAGGaCTcTAaATTGCccCctCTGAGgTCAAGGAaCACAAGATGgTTTTGGAAATGCTgAACCCGATaCATTATAACaTCACCAGCATcGTGCCtGAAGcCATGCCTGctGCCACCaTGCCagTcCTGCTcCTCACTGGCcTTtTTcTCTTgGTgTGGAATtATGAgGgcACATCCTCAATACCAGGtCCTGGCTAcTGCATGGGAATtGGaCCCCTCATcTCCCACggCaGaTTCCTGTGGATGGGGaTcGGCAGTGCCTGCAACTACTACAACcgGgTaTATGGAGAATTCaTGcGAGTCTGGATCtcTGGAGAGGAAACaCTCaTTATcAGCAAGTCCTCAAGTAtgTTCCACATaATGAAgCACaATCAtTACagCTCtCGATTcGGCAGCAaAcTTGGGcTGCAGTgcATcGGtATGCATGAGAAaGGCaTCATATTTAACAAcAAtCCAGagcTCTGGAAAaCaactCGACCcTTCTTTaTgAAAGCTcTGTCaGGCCCCgGCCTTGttCGtATGGTcACAGTcTGTGcTGAATCCcTCAAaAcaCATCTGGACAGGTTGGAgGAGGTgACCAATGaaTcGGGCtATGTggACGTGTTGACCCTtcTGCGtCGTgtCATGCTGGACaCcTCtAACACgCTCTTCtTGAGGATCCCtTTGGACATCGTGgTTAAAATCCAGGTTATTTTGATGCATGGCAaGCTCTCCTcATCAAACCAgACATCTTCTTTAAGATTTCTTGGCTATaCAaAAAGtATgAgAAGTCtGAaAGATGCCaTaGaAgTTCTgatAGcAGAAAAAAGACgCAGGATTTcCACAGaAGAGAAACTGGAAGAatgTaTGGAcTTTGCCACTGAAGAAACGTGGTGAcCTGACaAgAGAGAATGTGAACCAGTGCATATTGGAAATGcTGATcGCAGCtCCtGACACCaTGTCTGTCtCTtTGTTCTTCATGCTATtTCTCATTGCaaAGCACCCTAAtGTTGAAGAGGCAaTaATaAAGGAAATCCAGACTGTTaTTGGTGAgAGAGACaTAAAGAtTGATGATATaCAAAAATTAAAAGTGATGGAAAAcTTcATTtATGAGAGCATGCGGTACCAGCCTGTCGTGGACtTgGTCATGCGCAAAGCCTTAGAaGATGATGTaATCGATGGCTAcCCAGTGAAAAAGGGGACaAACATTATcCTGAATATTGGAAGgATGCAcAgACTCGAGTTTTTCCCCAAaCCcAATGAATTTACTCTTGAAAAtTTTGcaAAGAATGTTCCTTAtAGGTAcTTTCAGCCATTTGGcTTTGGGCCCCGTgGCTGtGCaGGAAAGTaCATCGCCATGGTGATGATGAAaGccATcCTcGTTaCACTTCTGAGACGATTCCAcGTGAAGACATTgCAAGGAcAgTGTgTTGAgAgcATaCAGAAgAtAcAcGACTTGtCCtTgCACCCaGATGAGaCTAaAAaCaTgCtGGaAATGATcTTTACcCCAAGAAAcTCAGAcAgGTGtCTgGAACACTAgAgAAGgcTGGTCAGTACCcAcTCtgGAGCATTTCTCATCAGTagTTCAcAtacAAAtCATCCATCcTTGCCATAGTGTCATCCTCAcAGTgAACAcTCaGTGGCCcaTGGCATTTTATAGGCATaCcTCCTATGGGTTGTcacCAAGCtAGGtgctATTtGTCATCTGcTCcTGTtCAcACCAGAGAACCAggCTaCAAGAGAAAAaGCAGAGGCCAAGAGTTTGaGGGaGAAATaGTCgGTGAAGAaAccGtAtCCaTAAAGaCCCgATTCCACcAAATGTGCTTTGagAAgGATAGGCCtTCACCaAGgGgATTTTTATGTCTGGGGCAGAAACACTcAAGTTGATTAGAAAGACCAGGCCaATgTCaGGGTACCTgGgGCCAAACccACcTGcTAGTGTgAATtAAaGTaCTTTaATTTtGTTTTctGTGGCAaCATTCATAGTCTTTGGAGAAATGCTTAgAaATTCAGCATTtGACccTTgCTGTGAATTAagccCaATTAAtTCcTGTTTgGATcTGTCTGTGGCAAAAGTtGAGgaaATtCTTTCCCAGTCTgTCgaTTtATGCCTCAGCCACTTgCCT

>CYP19A1_sheep

TAACAGCCGcGCATCATTAGCAAAACTCAcCATCTTCAAGAGTCCaAAACTAGAAGTGACCAGCAGACcCAGGaCTTTAaATTaCTTCcCCTGAGATCAAGtAaCACAAaATGcTTTTGGAAgTGCTgAACCCaAgGCATTAcAAtgTCACCAGCATGGTGtCCGAAGTtgTGCCTaTtGCCAgCaTtgCaATcCTGCTGCTCACTGGaTTTCTTcTCTTgGTTTGGAATtATGAgGAcACATCCTCAATACCAGGtCCcaGCTAcTttcTGGGAATtGGGCCCCTCATTTCCtACggCaGGTTtCTcTGGATGGGGaTTGGCAGTGCCTGaAAtTACTACAACAAGATGTATGGAGAATTCaTGAGAGTCTGGgTatGTGGAGAGGAAACcCTtaTTATTAGCAAGTCCTCAAGTAtgTTCCAtgTaATGAAgCACagTCACTACataTCCCGATTTGGCAGtAaAcTTGGGTTGCAGTtcATcGGCATGCATGAGAAaGGCaTCATATTTAACAATAAtCCAGCacTCTGGAAAGCTgTtCGACCTTTCTTTacAAAAGCTTTGTCcGGCCCtgGCCTgGtGCGCATGGTGACcaTcTGTGcTGAtTCCaTCAcCAAgCATCTGGACAGGTTGGAgGAGGTCtgCAATGacTTGGGCtATGTggACGTGTTGACCCTCATGCGgCGcAtCATGCTGGACaCcTCtAACAtCCTCTTCCTGgGGATCCCCTTGGAtATCGTGgTTAAAATCCAGGGgTATTTTGATGCATGGCAaGCTCTCCTTcTCAAACCAgACATCTTCTTTAAGATTTCTTGGCTgTgCAGAAAGtATgAAAAGTCCAgCAAgGAcTTGAaAGATGCCaTGGaAATTCTcatAGAAGAAAAAAGACACAGGATTTcaACAGCAGAGAAgCTGGAAGACtgcaTaGATTTcGCCACTGAgtTgATTTTtGCcGAGAAACGTGGTGAaCTtACaAAAGAGAATGTaAACCAGTGCATATTGGAAATGcTGATcGCAGCGCCAGACACCaTGTCTGTttCTGTGTTCTTCATGCTATtTCTCATTGCaaAGCAtCCccAgGTTGAAGAGGCAaTgATGAgGGAAATCCAGACTGTTgTTGGTGAAAGAGACaTAAgGAtTGATGATATGCAAAAgcTAAAAGTGgTGGAAAAcTTTATTAATGAGAGCATGCGGTACCAGCCcGTtGTGGACcTgGTCATGCGCAAAGCCTTAGAGGATGATGTCATCGATGGCTAcCCgGTGAAAAAGGGGACTAACATTATcCTGAATcTTGGAAGAATGCAcAgACTtGAGTTTTTCCCaAAGCCTAATGAATTTACTCTTGAAAAtTTTGccAAGAATGTTCCTTACAGGTAcTTTCAGCCATTTGGcTTTGGGCCCCGggcCTGtGCgGGAAAGTaCATCGCCATGGTGATGATGAAGGTcATcCTGGTcaCcCTTCTGAGACGcTTCCAcGTGcAGACtTTgCAAGGtcggTGTgTTGAgAAgATGCAGAAgAaAAATGACTTatCCtTgCAtCCaGAcGAGaCcAGcgaCcggCtGGaAATGATTTTcAtcCCgAGgAATTCAGAcAAGTGCCTCGAgtgCTAAAgAAGTTTGGTCAGTcCCTgccCCAGAGCAcTgCTCAaCAGaatTcCAcAtGGgAACCAcCCATCTTTGCCAGGTgGTcTGGCCTGTGcCATTTTATAGGCtTaCcTCCTgTGGGTTGTcaGCtAGCCAGGAgACAcgGtTCATCTGAcCagaTCCAAACCAGAACTGCAAGAGAAAATAGAGGCCAAGAGTTTGTGCAGCCCTAAAGaCCCaATTCCACAAAAGCtTTTTaAcaTCTGGGGCAGAAgCACCAAtTTGATTAcAAAGgCCAGGCCaAcATCTGcGTACCTcGgatCAAACAtAtcTccTAGTGTgAATAAAacaGtTTTcATTTGcTTTTTGGTGGGGTaGGgTTGGAGAAATGCTTACAaATTCAGCATTtGACTTTTCCTacGAAcTAcAtTCaATTAACTCTTGTTTATGACTgTCCTTTtCtgaTtgCTCAGTTtATGCCTCAGCCA

>CYP19A1_mouse

TTttaAACgGCtGcGCATCATTAGCAAAACTCAcCATCTTCAAGAGTCCaaAAACTAGAAGTGACCAGCAGAtcCAGGtGGtCCTGGCTAcTGtcTGGGAATtGGGCCCCTCATTTCCCAtggCaGaTTCtTGTGGATGGGGaTTGGaAGTGCCTGCAACTACTACAAtAAGATGTATGGAGAgTTCaTGAGAGTCTGGATCAGTGGAGAGGAgACaCTCaTTATcAGCAAGTGGCATGCATGAGAAcGGCaTCATATTTAACAAcAACCCAGCCATtGTGaagAAAATCCAGGGTTAcTTTaAcGCcTGGCAaGCTCTCCTcATCAAACCAAACATCTTCTTTAAGATTTCTTGGCTcTaCAGAAAGttTgGtgGAAaAgAAgAGACACAaagTTTcCACAGCtGAGAAACTGGAAGACtgTaTGGATTTTGCaACTGATtTgATTTTcGCTGAGAAACGTGGaGAcCTGACgAAAGAGAAcGTGAAtCAGTGCATAcTGGAgATGcTGATTGCAGCcCCtGAC

ACCaTGTCgGTCACTcTGTaCTTCATGtTActTCTCgTcGCagAGtAtCCagAgGTcGAAGcaGCAaTCcTGAAGGAgATCCAcACTGTTgTgGGTGAcAGAGACaTAAAGAtcGAgGATATcCAAAAtTTAAAAGTGgTGGAAAAcTTcATTAATGAaAGCATGCGGTACCAGCCTGTtGTGGACtTgGTCATGCGCAgAGCtcTgGAGGATGAcGTaATtGAcGGCTAcCCgGTtAAAAAGGGaACTAACATcATTCTGAAcATcGGAAGAATGCAcAggCTCGAGTacTTCCCtAAGCCcAATGAATTTACcCTTGAAAACTTTGAGAAGAATGTTCCcTACAGGTAcTTTCAGCCtTTTGGcTTTGGGCCCCGcgGCTGtGCTGGgAAGTaCATCGCCATGGTGATGATGAAaGTtgTctTGGTTaCACTTtTGAGACGATTCCAgGTGAAGACAcTgCAAaaAAggTGTaTTGAAAAcATaCcGAAAAagAATGACcTGtCCtTgCACCCaaATGAGgacAGgcaCcTtgtGGaAATaATTTTctCTCCAAGgAATTCAGAcAAGTaCCTCcAACAgTgAAgAAGTGGgAAAGAcAGaCCATCAGtAAAA

>CYP27B1_horse

ATGACCCAGACCCTCAAGCTCGCCTCCAGAGTGTTCCATCGCCTCCGCTGTGCTCCCGAGCTGGGCACCTCACTGGGTTCCAGAGGCTCCGACTCAGCGCCCCGGAGCTTGGCGGACATCCCAGGCCCCTCCACACCCGGCTTCCTTGCCGAACTTTTCTGCAAGGGGGGGCTGTCGCGGCTACACGAGCTGCAGGTGCAGGGAGCCGCGCGCTTCGGACCGGTGTGGTTGGCCAGCTTCGGGACGGTGCGCACGGTGTACCTGGCGGCCCCTACGCTCATCGAGCAGCTCCTACGACAGGAGGGTCCCCGGCCCGAGCGCTGCAGCTTCTCCTCCTGGGCTGAGCACCGTCGCTGCAGCCAGCGGGCTTGTGGACTACTCACCGCGGAAGGCGAAGAATGGCAGAGGCTCCGCAGCCTGCTGGCCCCGCTCCTCCTCCGGCCTCAAGCGGCCGCCCGCTACGCCGGGACCCTGGACGACGTGGTCCGTGACCTCGTGAGACGACTGCGGCGCCAGCGGGGACGGGGCGCTGGGCCGCCTGCTCTGGTTCGGGACGTGGCAGGGGAGTTTTACAAGTTTGGACTAGAAGGCATAGCCGCTGTGCTGCTGGGTTCGCGCCTGGGCTGCCTGGAGGCCGAAGTGCCCCCAGACACTGAGGCCTTCATCCGCGCGGTGGGTTCCGTGTTTGTGTCCACGCTGCTGACCATGGCGATGCCCAACTGGCTGCATCGCCTCGTGCCCGGACCCTGGGGACGCCTCTGCCGAGACTGGGACCAGATGTTCGCATTTGCCCAGCAGCACGTGGAGCGGCGCGAGGCCGAGGTCGCCGGGAGGAGCCCGGGAAAGCCTGAGGAGGACATGGGATCTGGGGCGCACCTTACCTACTTCCTGTTCCGGGAAGAGTTGTCAGCCTCGTCCATCCTGGGGAATGTGACGGAGCTGCTACTGGCTGGAGTGGACACGGTGTCCAACACGCTCTCTTGGGCTCTGTATGAACTCTCTCGGCACCCCGAAGTCCAGACGGCCCTGCACTCCGAGATCACAGCTGCCCTGGGCCCTGGCTCCAATGGCCACCATTCAGCCACTGCTCTGTCCCGGCTGCCCCTGCTTAAGGCCGTGGTCAAGGAAGTGCTGAGACTGTACCCCGTGGTACCTGGAAATTCCCGTGTCCCAGACAGAGACATTCATGTGGGTGACTATATTATCCCAAAAAATACGCTGGTCACACTGTGTCATTATGCCACATCAAGGGACCCTGCTCAGTTTCCAGAGCCAAATTCTTTTCGTCCAGCTCGCTGGTTGGGGGAGGGTCCAGCGCCCCATGCGTTTGCATCTCTCCCTTTTGGCTTTGGCAAGCGCAGCTGTATGGGGAGACGCCTGGCAGAGCTTGAGCTGCAAATGGCTTTGGCCCAGATCTTGATCCACTTTGAGGTGCAGCCTGAGCCAGGTGCTACTCCAATCAGACCCATGACCCGGACTGTCCTGGTACCCGAGAGGAGCATCAACCTACAGTTTGTGGACAGATAGTCCCATGGAAGGAGGCTGTCATCATCACC

>CYP27B1_rhino

ATGACCCAGACCCTCAAGCTCGCCTCtAGAGTGTTgCATCGCaTggGCTGTGCTCCCGAGCTGGGCACCTCgCTGGGcTCCAGAGGCTCCtACTCAGCGCCCCGGAGCTTGGCGGACATCCCAGGCCCCTCtACgCCCGGCTTCCTTGCCGAACTTTTCTGCAAGGGcGGGCTGTCGCGGCTACACGAGCTGCAGGTGCAcGGcGCtGCaCGCTTCGGgCCGGTGTGGTTGGCCAaCTTCGGGACGGTGCGCACGGTGTACCTGGCGGCCCCTACaCTCATCGAGCAGCTCCTACGACAGGAGGGgCCCCaGCCCGAGCGCTGCAGCTTCTCacCCTGGGCcGAGCACCGcCGCcGCcGCCAGCGaGCTTGTGGACTACTCACCGCGGAAGGCGAAGAATGGCAGAGGCTCCGCAGCCTcCTGGCCCCGCTCCTCCTCCGGCCTCAAGCGGCCGCCCGCTACGCCGGGACCCTGGACGACGTGGTCCGTGACCTtGTGcGACGACTGCGGCGCCAGCGGGGACGGGGCGCTGGGCCGCCcGCgCTGGTTCGGGACGTGGCAGGaGAGTTTTACAAGTTTGGACTAGAAGGCATAGCCGCcGTGCTGCTGGGTTCGCGCCTGGGCTGCCTGGAGGCCGAAGTGCCCCCAGACACaGAGaCCTTCATCCaCGCGGTGGGaTCgGTGTTTGTGTCCACGCTGCTGACCATGGCGATGCCCcACTGGCTGCAcCGCCTCGTGCCCGGACCCTGGGGcCGCCTCTGCCGgGACTGGGACCAGATGTTtGCATTTGCCCAGCAGCACGTGGAGCaGCGCGAGGCCGAGaTaGCtGtGAGGAGCCaGGaAAAGCCTGAGGAGGACATGGGATCTGGGGCGCACCTaACCTACTTCCTGcTCCGGGAAGAGcTGcCtGCCTCGTCCATCCTGGGGAAcGTGACGGAGCTGCTACTGGCcGGgGTGGACACGGTGTCCAACACGCTCTCcTGGGCTCTaTATGAACTCTCTCGGCACCCCGAAGTCCAGgtGGCCCTcCACTCtGAGATCACAGCTGCCCTGGGtCCTGGCTCCAATGGCCACCAcTCAGCCACTGCTCTGTCtCGGCTGCCCCTGCTgAAGGCCGTcGTCAAGGAAGTGCTGAGACTGTACCCCGTGGTACCTGGAAATTCCCGTGTCCCAGACAaAGAtgTTCgTGTGGGTGACTATATTATCCCAAAAAATACGCTGGTCACACTGTGTCATTATGCCACtTCAAGGGACCCaGCcCAGTTcCCAGAGCCAAATTCTTTTCGTCCAGCTCGCTGGcTGGGGGAGaGTCCAGCcCCaCAcGCaTTTGCATCTCTtCCcTTTGGCTTTGGCAAGCGCAGCTGcATGGGGAGACGCCTGGCAGAGCTTGAGCTGCAAATGGCTTTGGCCCAGATCTTGAcCCACTTTGAGGTGCAGCCTGAGCCAGGTGCTgCcCCAATCAGACCCATGACCCGGACTGTCCTGGTACCtGAGAGGAGCATCAACCTACAGTTTGTGGACAGATAGTCCCgTGGAAGGAGGCTGTCATCATt

>CYP27B1_cow

ATGACCCAGACCCTCAAatTtGCaTCCAGAGTGTTCCATCGCgTCCGCTGTCCtGAGCTGGGCgCCTCACTGGGcTCCAGAGGCTCCGAgTCAGCGCCCCGGgttTTGGCGGACATCCCAGGCCCCTCCACgCCCGGCTTCCTTGCgGAgCTTTTCTGCAAaGGGGGGCTGTCaCGGCTACACGAGCTGCAGGTGCAGGGcGCCGCGCGCTTCGGgCCGGTGTGGTTGGCCAGCTTCGGGACGGTGCGCACcGTGTACCTGGCGGCCCCTACGCTCgTCGAGCAGCTgCTACGACAGGAGGGaCCCCGGCCCGAaCGCTGCAGCTTCTCacCCTGGaCTGAGCACCGTCGCcGCcGCCAGCGGGCTTGcGGAtTgCTCACCGCGGAAGGgGAAGAATGGCAGAGGCTtCGCAGCCTcCTGGCCCCGCTgCTCCTCCGGCCTCAAGCGGCCGCCCGCTAtGCCGGGACCCTacACGgCGTGGTgCGTGACCTtGTGAGgCGACTGCGGCGCCAGCGaGGACtGGGCGCTGGGCCGCCctCcCTGGTTCGaGACGTGGCgGGaGAGTTTTACAAGTTTGGACTgGAAGGCATtGCtGCgGTGCTGCTGGGcTCcCGCCTcGGCTGCCTGGAGGCaGAgGTGCCCCCAGACACaGAGaCCTTCATCCGCGCGGTcGGcTCgGTGTTcGTGTCCACGCTctTGACCATGGCGATGCCCAgCTGGCTGCAcCGCgTCGTGCCCGGACCCTGGGacCGCCTCTGCCGAGACTGGGACCAGATGTTtGCATTcGCCCAGCAGCAtGTaGAGCaGCGaGAGGCtGAGGTaGCCatGAGGAAGtCTGAGGAGGACATGGGAcCTGGGGCaCACCTgACCTACTTCtTGcTCCaGaAAGAGTTGcCtGCCgCGTCCATCCTGGGGAATGTGACGGAGCTGCTtCTcGCTGGgGTGGACACGGTGTCCAACACtCTCTCcTGGGCTCTGTATGAACTCTCcCGGCACCCCGAAaTCCAGACaGCaCTcCAtgCtGAGATCACAGCTGCCtTGGGCCCcGGCTCCAgTacCCAaCccTCAGCCACTGCTCTGTCCCaGCTGCCCCTGCTgAAGGCCGTGGTCAAGGAAGTGCTaAGACTGTACCCtGTGGTACCTGGAAATTCCCGTGTCCCAGACAGAGACATTtgTGTGGGTGAaTATATcATCCCcAAAAATACGCTGGTCACtCTGTGTCAcTATGCCACtTCAAGGGACCCTGCcCAGTTcCCAGAaCCAAATTCTTTTCGTCCAGCTCGCTGGcTaGGGGAaGGTCCAGCtCCCCAccCaTTTGCATCTCTCCCcTTcGGCTTTGGCAAGCGCAGCTGcgTGGGGAGACGCCTGGCAGAGCTTGAGCTGCAAATGGCcTTGGCCCAGATCTTGATCCACTTTGAGGTaCAaCCTGAGCCAGGTtCTgCcCCtgTCAGACCaATGACCCGGACTGTCCTGGTACCtGAGAGaAGCATCAACCTACAGTTTGTGGACAGATAaTatggTGGAAGcAGaCTGTCATt

>CYP27B1_dog

ATGACCCAGgCCCTCAAGCTCGCCTCCAGAGTGTTCCATCGCaTCCaCTGgGCTCCCaAGCTGGGCtCCTCACTGGGcTCCAGAGGCTCCGACTCAGCaCCCCGcAGCTTGGCGGACATCCCAGGCCCCTCCACACCCGtCTTCCTTGCtGAACTTTTCTGCAAGGGcGGtCTGTCGCGGCTACACGAGCTGCAGGTGCAGGGcGttGCGCGCTTCGGgCCtGTGTGGTTGGCCAGCTTCGGGAaGGTGCGCACcGTGTACgTGGCGGCCCCTgCcCTCgTCGAaCAGCTCtTgCGACAGGAGGGgCCCCGGCCgGAGCGCTGCAGCTTCTCgcCCTGGGCaGAGCACCGaCGCcaCcGCCAGCGGGCTTGcGGgCTgCTCACCGCGGAAGGCGAgGAAgGGCAGAGGCTCCGCAGCCTcCTGGCCCCGCTgCTCCTCCGGCCTCgAGCcGCCGCCCGCTACGCCGcGcCCCTGGcCGACGTGGTCCGcGACCTtGTGcGgCGcCTGCGGGGTTCGGGACGTGGCcGcaGAGTTcTACAAGTTTGGACTAGAAGGCAcAGCCGCcGTGCTcCTGGGTTCcCGCCTGGGCTGCCTGGAGGCCcAAGTGCCCCCcGACACgGAcGCCTTCATCCGCGCcGTGGGgTCCGTGTTTGTGTCCACGCTGCTGACCATGGCGATGCCCggCTGGCTtCAcCGCCTgGTGCCCGGgCCCTGGGGcCGCCTCTGtCGAGACTGGGACCAGATGTTtGCATTTGCCCAGCAGCACGTGGAGaGGCGgGAGGCCGAGGTaGCCttGAGGAGCgaGGGgAAGgCcGcGGAGGAtgTGGGATCTGGGGCaCACCTgACCTACTTCCTGcTCCGGGAAGAGcTGcCtGCCcCGTCCATCCTGGGcAAcGTGACGGAGCTGCTACTGGCTGGcGTGGACACGGTgTCCAACACGCTCTCcTGGGCgCTGTATGAACTCgCTCGGCACCCgGAcGTgCAGACtGCgCTcCACTCtGAGATCACAGCTGCCCTGGGCCCTGGCTCCAgTGcCCACCccTCAGCtgCgGCTCTaTCCCaGCTGCCtCTGCTgAAGGCaGTGGTCAAGGAgGTGCTGAGACTGTACCCtGTGGTACCTGGAAATTCCCGTGTCCCAGACAaAGACATTCgTGTGGGTGACTATATTATCCCcAAAAATACaCTGGTCACACTGTGTCATTATGCCACtTCAAGGGAtCCTGCcCAGTTcCCAGAGCCAAATTCTTTTCGTCCAGCTCGaTGGcTGGGGGAaGGTCCAGCcCCCCAccCaTTTGCcTCTCTtCCcTTTGGCTTTGGCAAGCGCAGCTGcATGGGGAGACGCCTGGCAGAGCTTGAGCTGCAAATGGCTTTGGcCCAGATCcTGAcCCACTTTGAGGTGCAGCCTGAGCCAGGTGCTgCcCCgATCAGgCCaATGACCCGGACTGTCtTGGTACCCGAGAGGAGCATCAACCTACAGTTTGTGGACAGATAGTCCCATaGgAaGAaGCT

>CYP27B1_pig

ATGACCCAaACCCTCAAGCTCGCtTCCAGAGTGTTCCATCGCgTCtGCcGTGCTCCtGAGCTGGGTTCCAGAGGCTCCGACTCAGCGCCtCGGgGaTTGCCTCCACgCCtGGtTTCCTTGCCGAACTTTTCTGCAAGGGGGGtCTGTCaCGGCTgCACGAGCTcCAGGTGCAGGGtGCCGCGCGCTTtGGcCCaGTGTGGTTGGCCAGtTTCGGGAaGGTGCGCACGGTGTACgTGGCGGCCCCTACGCTCgTCGAGCAGCTgCTACGACAGGAGGGaCCCttGCCCGAGCGCTGCAGCTTCTCacCCgGGaCgGAGCACCGTCGCcGacGCCAGCGGtCTTGcGGACTgCTCACCGCGGAAGGtGAAGAATGGCAGAGGCTCCGCAGtCTcCTGGCCCCGtTgCTCCTCCGGCCTCAAGCGGCaGCCCGCTAtGCCGGGACCCTGcAtGACGTGGTCCagGACCTtGTGcGgCGACTGCGGaGCCAGCGGGGACtGGGCGCTGGGCCtCCcGCcCTGGTTCGGGCGGTGGGaTCgGTGTTTGTGTCCACGCTcCTGACCATGGCGATGCCCAgCTGGCTGCAcCGCCTCCGCCTCTGCCGcGACTGGGACCAGATGTTtGCATTTGCCCAGgAGCACGTGGAGCGGCGaGAGGCCGAGGctGCCatGAaGAGCCaGGGAAAGCCTGAGGAGGACtTGGGATCTGGGGCGCACCTgACCTACTTCCTcTTCCGGGAAGAGcTGcCAGCCcCGTCCATCCTGGGGAATGTGACaGAGtTGCTACTGGCTGGAGTGGACACGGTGTCCAACACaCTCTCcTGGGCTCTcTATGAACTCTCTCGGCACCCtGAAGTCCAGAtGGCaCTcCAtTCtGAGATCAaAaCTGCttTGGGCCCcaGCTCCAgTGcCCACCcaTCAGCCACTGtTCTaTCCCaGCTGCCCCTGCTTAAGGCaGTGGTCAAGGAAGTGCTaAGACTGTACCCtGTGGTACCTGGAAAcTCCCGTGTgCCAGACAaAGACATTtgTGTGGGTGACTAcATTATCCCcAAAAATACaCTGGTCACtCTGTGTCAcTATGCCACtTCAAGGGACCCTGCcCAGTTcCCAGAGCCAAATTCTTTTCGTCCAGCTCGCTGGcTGGGGGAatGTCCAGCcCCCCAccCaTTTGCATCTCTCCCcTTTGGCTTTGGCAAGCGCAGCTGcATGGGGAGACGCCTGGCAGAGCTTGAGCTGCAAATtGCTTTGGCCCAGATCTTGATCCACTTTGAGGTGCAGCCTGAGCCAGGTtCTgCcCCAATCAGACCCATGACCCGGACTGTtCTGGTACCtGAGAGaAGCATCAAtCTACAGTTTGTGGACAGATAGTCCtgTGGAAGGAGGCTGTCATCATCACC

>CYP27B1_elephant

ATGACCCAGACCCTtAAGCTtaCtTaCAGgGTGTgCCATCGCgTCCGCTGgGCTCCCGAGCTGGGCgCCTCACTGGGcTCCAGAGGCgtCtgCTCAGCGCCCCGGAGCTTGGgGGAtATCCCAGGCCCgTCCACgCCCGGCTTtCTaGCCGAACTcTTCTGCAAGGGGGGaCTGTCtCGGCTACACGAGCTGCAGGTGCAGGGcGCCGCGCGCTTCGGtCCGGTGTGGTTGGCCAGtTTCGGGACcGTaCGCACaGTGTACCTGGCGGCtCCTgCGCTCATtGAGCAGCTgCTgCGACAGGAGGGgCCCCtGCCaGAGCGCTGCAGCTTCTCgcCCTGGaCcGAGCACCGgCGCcagcGgCAGCGGGCTTGTGGACTgCTCACCGCGGAAGGtGAgGAgTGGCAGAGGCTCCGCAGCCTcCTGGCCCCGCTCCTCCTtCGGCCTCAAGCaGCCGCggGaTACGCCGGaACCCTGGACagCGTGGTCCGTGACCTtGTGcGgCGAtTGCGGCGCCAGCGGGaACaGGcCGCTGGGCCGCCcGCcCTGGTTCGGGACGTGGCgGGaGAGTTTTACAAGTTcGGgtTAGAAGGCATcGCCGCgGTaCTGCTGGGTTCGaGgCTGGGCTGtCTGGAGGCCGAAGTGCCgCCgGACACaGAGaCaTTCATCCGCGCaGTGGGcTCtGTGTTTGTGTCCACGCTGCTaACCATGGCGATGCCgAACTGGCTGCAcCGtCTaGTGCCtGGACCtTGGGGcCGCCTCTGCCGtGACTGGGACCAGATGTTtGCATTTGCCCAGCAGCACGTGGAGCGaCGaGAGGCtGAaGTgGCCatGAGGAcCCatGGAAAGCCTGAGGgGGAtgTGGcAcCTGGGGtGCAtCTgACCTACTTCCTGTTCCGaGAAaAacTGTCtGCCcCGTCCATCCTGGGGAATGTGACaGAaCTGCTgCTGGCcGGAGTGGACACGGTGTCCAACACtCTCTCcTGGGCTCTGTATGAgCTCTCTCGGCACCCCGAcaTCCAGAtGGCaCTcCACTCgGAGATCACAGCTGCCCTGGGCCCcaGCTCCtgTGcCCACCtTcCAGCCACTGCTCTGTCCCaGCTGCCCCTaCTaAAGGCtGTGGTCAAGGAgGTGCTGAGACTaTACCCtGTGGTACCTGGAAATTCCCGTGTCCCAGACAaAGACATTCATGTGGGTGACTAcATcATCCCcAAAAgACaCTGGTCACtCTGTGTCAcTATGCCACtTCtcGtGAtCCTGCcCAGTTcCCAGAGCCAAATTCTTTTCGTCCAGCTCGCTGGcTGGGGGAGGGTCCAGCcCCCCAccCaTTTGCATCTCTCCCcTTTGGCTTTGGCAAGCGCAGCTGcATGGGGAaACGCCTGGCAGAGCTccAGCTaCAAATGGCTTTaGCCCAGATCTTGAcCCACTTTGAGGTGCgGCCTGAGCCAGGTGCTgCcCCAATCAGACCCATGACCCGGACcGTCCTGGTACCCGAGcGGAGCATCAACCTACAGTTTGTGGACAGATAGcCCtcTGGAAGcAGGCTGTCATCATCACg

>CYP27B1_human

ATGACCCAGACCCTCAAGtaCGCCTCCAGAGTGTTCCATCGCgTCCGCTGgGCgCCCGAGtTGGGCgCCTCcCTaGGcTaCcGAGagTaCcACTCAGCaCgCCGGAGCTTGGCaGACATCCCAGGCCCCTCtACgCCCaGCTTtCTgGCCGAACTTTTCTGCAAGGGGGGGCTGTCGaGGCTACACGAGCTGCAGGTGCAGGGcGCCGCGCaCTTCGGgCCGGTGTGGcTaGCCAGCTTtGGGACaGTGCGCACcGTGTACgTGGCtGCCCCTgCaCTCgTCGAGgAGCTgCTgCGACAGGAGGGaCCCCGGCCCGAGCGCTGCAGCTTCTCgcCCTGCtCCtTCaCTGCAGCCAGGAAGGCGAAGAATGGCAaAGGCTCCGCAGtCTcCTGGCCCCGCTCCTCCTCCGGCCTCAAGCGGCCGCCCGCTACGCCGGaACCCTGaACaACGTaGTCtGcGACCTtGTGcGgCGtCTGaGGCGCCAGCGGGGACGtGGCaCgGGGCCGCCcGCcCTGGTTCGGGACGTGGCgGGGGAaTTTTACAAGTTcGGACTgGAAGGCATcGCCGCgGTtCTGCTcGGcTCGCGCtTGGGCTGCCTGGAGGCtcAAGTGCCaCCcGACACgGAGaCCTTCATCCGCGCtGTGGGcTCgGTGTTTGTGTCCACGCTGtTGACCATGGCGATGCCCcACTGGCTGCgcCaCCTtGTGCCtGGgCCCTGGGGcCGCCTCTGCCGAGACTGGGACCAGATGTTtGCATTTGGGAcAGCCcGAGaAGGACcTGGagTCTGGGGCGCACCTgACCcACTTCCTGTTCCGGGAAGAGTTGcCtGCCcaGTCCATCCTGGGaAATGTGACaGAGtTGCTAtTGGCgGGAGTGGACACGGTGTCCAACACGCTCTCTTGGGCTCTGTATGAgCTCTCcCGGCACCCCGAAGTCCAGACaGCaCTcCACTCaGAGATCACAGCTGCCCTGaGCCCTGGCTCCAgTGcCtACCccTCAGCCACTGtTCTGTCCCaGCTGCCCCTGCTgAAGGCgGTGGTCAAGGAAGTGCTaAGACTGTACCCtGTGGTACCTGGAAATTCtCGTGTCCCAGACAaAGACATTCATGTGGGTGACTATATTATCCCcAAAAATACGCTGGTCACtCTGTGTCAcTATGCCACtTCAAGGGACCCTGCcCAGTTcCCAGAGCCAAATTCTTTTCGTCCAGCTCGCTGGcTGGGGGAGGGTCCcaCcCCCCAccCaTTTGCATCTCTtCCcTTTGGCTTTGGCAAGCGCAGCTGTATGGGGAGACGCCTGGCAGAGCTTGAatTGCAAATGGCTTTGGCCCAGATCcTaAcaCAtTTTGAGGTGCAGCCTGAGCCAGGTGCggCcCCAgTtAGACCCAaGACCCGGACTGTCCTGGTACCtGAaAGGAGCATCAACCTACAGTTTtTGGACAGATAGTCCCATGGAAaGAGaCTGTCATCATCACC

>CYP27B1_sheep

ATGACCCAGACCCTCAAatTCGCaTCCAGAGTGTTCCATCGCgTCCGCTGTCCtGAGCTGGGCgCCTCACTGGGcTCCAGAGGCTCCGACTCAGCaCCCCGGgttTTGGCGGACATCCCAGGCCCCTCCACgCCCGGCTTCCTTGCgGAgCTTTTCTGCAAaGGGGGGCTGTCaCGGCTACACGAGCTGCAGGTGCAGGGcGCCGCGCGCTTCGGgCCGGTGTGGTTGGCCAGCTTtGGGACGGTGCGCACcGTGTACCTGGCGGCCCCTACGCTggTCGAGCAGCTgCTACGACAGGAGGGaCCCCGGCCCGAaCGCTGCAGCTTCTCacCCTGGaCgGAGCACCGTCGCcGCcGCCAGCGGGCTTGcGGACTgCTtACCGCGGAAGGgGAAGAATGGCAGAGGCTtCGCAGCCccCcGGCCCCGCTgCTCCcCCcGCCcCAAGCGGCCGCCGCgcGcCCGCCcGCcCTGGTTCGaGACGTGGCgGGaGAGTTTTACAAGTTTGGACTgGAAGGCATtGCtGCgGTGCTGCTGGGcTCcCGCCTcGGCTGCCTGGAGGCgGAgGTaCCCCCAGACACaGAGaCCTTCATCCGCGCGGTcGGcTCgGTGTTcGTGTCCACGCTGtTGACCATGGCGATGCCCAACTGGCTGCAcCGCgTCGTGCCCGGACCCTGGGacCGCCTCTGCCGAGACTGGGACCAGATGTTtGCATTcGCCCAGCAGCACGTaGAGCaGaGaGAGGCtGAGGTaGCCatGAGGGGgAAGtCTGAGGAGGACAcGGGAcCTGcGGCaCACCTgACCTACTTCtTGcTCCGGaAAGAGcTGcCtGCCgCGTCCATCCTGGGGAATGTGACGGAGCTGCTtCTaGCTGGgGTGGACACGGTGTCCAACACGCTCTCcTGGGCTCTaTATGAACTCTCcCGGCACCCtGAAaTCCAGACaGCaCTcCAtgCtGAGATCACAGCTGCCtTGGGCCCTGGCTCCAgTacCCAaCccTCAGCCACTGCcCTaTCCCaGCTGCCCCTGCTgAAGGCCGTGGTCAAGGAAGTGCTaAGAtTGTACCCCGTGGTACCTGGAAATTCCCGTGTCCCAGACAGAGACATTtgTGTGGGTGAaTATATcATCCCcAAAAATACaCTGGTCACtCTGTGTCAcTATGCtACtTCAAGGGACCCTGCcCAGTTcCCAGAaCCAAAcTCTTTTCGTCCAGCTCGCTGGcTaGGGGAaGGTCCAGCtCCCCAccCaTTcGCATCTCTCCCcTTTGGCTTTGGCAAGCGCAGCTGcATGGGaAGACGCCTGGCAGAGCTTGAGtTGCAcATGGCcTTGGCCCAGATCTTGATCCACTTTGAGGTGCAaCCTGAGCCAGGTtCTgCcCCcgTCAGACCgATGACCCGGACTGTCCTGGTgCCtGAGAGaAGCATCAACCTACAGTTTGTGGACAGATAGTatggTGGAAatAGGCTGTCAcCATCt

>CYP27B1_mouse

ATGACCCAGgCagTCAAGCTCGCCTCCAGAGTtTTtCAcCGaTCgCTGGGcTCCAGAGGCagtGAgTCgGttCtCCGGAGCTTGtCtGACATCCCtGGgCCCTCtACACtCaGCTTCCTgGCtGAACTcTTCTGCAAaGGGGGGCTGTCcaGGCTgCAtGAaCTGCAGGTGCAtGGcGCtGCGCGgTaCGGgCCaaTaTGGTctGgCAGCTTtGGGACacTtCGCACaGTtTACgTtGCcGaCCCTACaCTtgTgGAGCAGCTCCTgCGACAaGAaaGTCaCtGtCCaGAGCGCTGtAGtTTCTCaTCaTGGGCaGAGCACCGTCGCcGCcaCCAGCGtGCTTGcGGAtTgCTaACgGCGGAAGGtGAAGAATGGCAGAGGCTCCGaAGtCTtCTGGCCCCGCTCCTCCTCCGGCCaCAAGCaGCCGCggGCTAtGCtGGaACtCTGGACaACGTGGTCCGTGACCTtGTGcGACGACTaaGGCGCCAGCGGGGACGtGGCtCTGGGCtaCCcGgcCTaGTTCtGGACGTGGCAGGaGAGTTTTACAAaTTTGGcCTAGAAGGtATAGgCGCgGTGCTGCTGGGaTCGCGCCTGGGCTGCCTaGAGGCtGAAGTcCCtCCtGACACaGAaaCCTTCATaCatGCaGTGGGcTCaGTGTTTGTGTCtACaCTctTGACCATGGCGATGCCCAACTGGtTGCAcCaCCTtaTaCCtGGACCCTGGGccCGCCTCTGCCGAGACTGGGAtCAGATGTTtGCcTTTGCCCAGagGCACGTGGAGCtGCGaGAaGgtGAaGctGCgatGAGGAaCCaGGGAAAGCCTGAGGAGGAtATGccgTCTGGGcatCACtTaACCcACTTCCTtTTtCGGGAAaAGgTGTCtGtCcaGTCCATagTGGGGAATGTGACaGAGCTaCTACTGGCTGGAGTGGACACGGTGTCCAAtACGCTCTCcTGGaCaCTcTATGAgCTtTCcCGGCACCCCGAtGTCCAGACtGCaCTcCACTCtGAGATCACAGCTCCgTGGCTCCtgTGcCCACCcccatGgCACTGCTCTGTCCCaGCTGCCCCTGtTaAAGGCtGTGaTCAAaGAAGTGtTGAGAtTGTACCCtGTGGTACCTGGgAATTCCCGTGTCCCAGACAGAGACATcCgTGTaGGaaACTATgTaATtCCcACGCTaGTCtCcCTaTGTCAcTATGCCACtTCAAGGGACCCcaCaCAGTTTCCAGAcCCcAAcTCTTTTaaTCCAGCTCGCTGGcTGGGGGAGGGTCCgaCcCCCCAccCaTTTGCATCTCTtCCcTTcGGCTTTGGCAAaCGgAGCTGcATcGGGAGACGCtTGGCAGAGCTTGAGCTaCAAATGGCTTTGtCCCAGATCTTGAcCCAtTTTGAaGTGCtaCCTGAGCCAGGTGCTctTCCtATCAagCCCATGACCCGGACTGTCCTGGTcCCtGAGAGGAGCATCAAtCTACAGTTTGTaGAtAGATAacCattcGGAAGacaGCcaaCATCgTCt

>CYP2A13_horse

ATGCTGGCCTCAGGGCTGCTTCTGGTGGCTTTGCTGTCCTGCCTGACTTTAATGGTATTGATGTCTGTCTGGCGGCAGAGAAAGCTCTGGGGAAGGCTGCCTCCTGGACCCACCCCATTGCCCTTCATCGGGAACTACCTGCAGCTGGACACAGAGCGGATATGTGACTCCCTCATGAAGATAGGCAAGCGCTATGGGCCAGTGTTCACAGTTCACCTGGGGCCCCGCCGGGTCGTGGTGCTATGTGGATACGATGCTGTGAAGGAGGCTCTGTTGGACCAGGCTGAGGAATTCAGTGGACGAGGAGAGCAGGCCACCTTCAACTGGATCTTCAAAGGCTATGGTGTGGCATTCAGCAATGGGGAGCGGGCCAAGCAGCTCAGGCGCTTCTCCATCACCACACTGAGGGACTTCGGAGTGGGTAAGCGTGGCATTGAGGAGCGCATCCAGGAGGAGGCGGGCTTCCTCATCGAGGCCTTCCGGAGCACGTGTGGCAACTTCATTGATCCCACCTTCTTCCTGAGCCAAGCTGTCTCCAATGTCATCAGCTCCATTGTCTTTGGGAACCGCTTTGACTATGAGGACAAAGAGTTCCTGTCACTGCTGCGTATGATGCTGGGAAACTTCCAGTTCACAGCTACATCTACGGGGCAGCTCTATGAGATGTTCTACTCAGTGATGAAACACCTGCCAGGGCCACAGCAACAGGCCTTTAAGGAGCTGCAGGGCCTGGAGGACTTCATAGCCAAGAAGGTGGAGCAGAACCAACGCACCCTGGATCCCAACTCCCCGCGGAACTTCATCGACTCCTTCCTCATCCGCATGCAGCAGGAACAGACGAACCCTAACACGGAGTTCTACTTGAAGAACCTGGTGATAACCACGTTGAACCTCTTCATTGCTGGCACAGAAACGATCAGCACAACCCTGCGTTATGGCTTCCTGCTGCTGATGAAGCACCCAGATATAGAAGCCAAGGTTCATGAGGAGATTGATCGAGTGATTGGCAAGAACCGTCAGCCCAAGTTTGAGGACCGGGCCAAGATGCCCTACACAGAGGCAGTGATCCACGAAATCCAAAGATTTGGAGATGTGATCCCCATGAGCCTGGCCCGTAGAGTTACCAAGGATACCAAGTTTCGGGGCTTCCTCATCCCCAAGGGCACTGAAGTGTTCCCTATGCTGGGCTCCGTCCTGAGAGACCCCAAGTTCTTCTCCAATCCCCACGATTTCAACCCCCAGCACTTCCTGGATGAGAAGGGGCAGTTTAAGAAGAGTGATGCTTTTGTGCCTTTCTCCATTGGTAAGCGGTACTGCTTTGGAGAAGGCCTGGCTAGAATGGAGCTCTTTCTCTTCCTCACCACCATCATGCAGAATTTCTGCTTCAGGTACCCTCAGTCGCCCCAGGATATCGACGTGTCCCCGAAACACGTGGGCTTTGCCAGCATTCCAAGAAACTACACCATGAGCCTCCAGCCCCGCTAA

>CYP2A13_rhino

ATGCTGGCCTCAGGGCTGCTTCTGGTGGCTTTGtTGgCCTGCCTGACTgTAAcGGTcTTGATGTCTGTaTGGaGGCAGAagAAGCTCTGGGGgAaGCTGCCTCCTGGACCCACCCCgTTGCCCTTCATCGGGAACTACtTGCAGCTGaACACgGAGCaGATgTacaACTCCCTCATGAAGATcaGCAAaCGCTATGGGCCAGTGTTCACgGTcCACCTGGGGCCCCGgCGGaTCGTGGTaCTgcGTGGcTAtGAaGCTGTGAAGGAGGCTCTGgTGGACCAGGCTGAGGAATTCAGTGGACGAGGAGAGCAGGCCACCTTCgACTGGcTCTTCAAAGGCTATGGTGTtGCgTTCAGCAATGGGGAGCGcGCCAAGCAaCTCAGGCGCTTCTCCATCACCACcCTGAGGGACTTtGGcGTGGGcAAGCGTGGCATcGAGGAGCGCATCCAGGAGGAGGCGGGtTTCCTCATtGAGGCCTTCCGGAGtACtcaTGGCAcCTTCATcGATCCCACCTTCTTCCTGAGCCgAaCaGTCTCCAATGTCATCAGtTCCATTGTCTTTGGGgACCGCTTTGACTATGAGGACAAAGAGTTCCTGTCACTGCTGCGTATGATGCTGGGAAgCTTCCAGTTCACAGCTACATCTACaGGGCAGCTCTATGAGATGTTCTACTCAGTGATGAAACACCTGCCAGGGCCACAGCAgCAGGCCTTTAAGGAGCTGCAGGGgCTGGAGGACTTCATAGCCAAGAAGGTGGAGCAGAACCAACGCACCCTGGATCCCAACTCCCCGCGGgACTTCATCGACTCtTTCCTCATCCGCATGCAGCAGGAgCAGAaGAACCCTAAtACaGAGTTCTgCTTcAAGAACCTGGTacTgACCACGcTGAACCTCTTCtTTGCgGGCACAGAgACGgTCAGCACgACCCTGCGTTATGGCTTCCTGCTGCTcATGAAGCACCCAGATgTgGAAGCCAAGGTcCATGgGGAGgTTGAcCGgGTGATTGGCAAGAACCGTCAGCCCAAGTTTGAGGACCGaGCCAAGATGCCCTACACAGAGGCgGTGATCCACGAgATCCAAAGATTTGGAGAcaTGATCCCCATGgGCCTGGCCCGcAGAGTcACCAAGGAcACCAAaTTTCGGGaCTTCaTCcTCCCCAAGGGCACTGAAGTGTTCCCTATGCTcGGCTCtGTgCTGAGAGAaCCCAAGTTCTTCTCCAATCCCCAaGATTTCAACCCtgAGCACTTCCTGGATGAcAAGGGGCAGTTTAAGAAGAGTGATGCTTTTGTGCCcTTCTCCcTcGGTAAGCGGaACTGtTTTGGAGAAGGCtTGGCTAGAATGGAGCTCTTcCTCTTCCTCACCACCATCATGCAGAAcTTtTGCTTCAaGTctCCgCAGgCaCCCCAGGAcATCGACGTGTCCCCcAAACACGTGGGCTTTGCCAcCATTCCAcGAAACTACACCATGAGCtTCCtGCCCCGCTgA

>CYP2A13_cow

ATGCTGGCCTCAGGGCTGCTcCTcGTGGCTTTGCTGgCCTGtCTGACTaTcATGGTcTTGATGTCTGTCTGGCGGCAaAGgAAcCTCaaaGGgAaatTGCCTCCaGGgCCCACtCCtcTGCCCTTCATCGGGAACTACCTGCAGCTGaACACcGAGCaGATgTGcaACTCCCTCATGAAGATcaGCgAGCaCTAcGGcCCtGTGTTCACgGTcCACCTGGGGaCCCGgCaGaTtGTGGTGCTgTGTGGcTAtGATGCTGTGAAGGAGGCTCTGgTGGACCAGGCTGAaGAATTCAGTGGgCGAGGcaAGCAGGCtACCTTtgACTGGcTCTTCAAAGGCTATGGTGTGGCgTTCAGCAAcGGGGAGCGcGCCAAGCAGCTCcGGCGCTTCTCgATtACaACtCTGcGGGACTTCGGcGTGGGcAAGCGcGGCATcGAaGAGCGCATCCAGGAGGAGGCGGGCTTCCTCATCGAGGCCTTCCGGgGCACtcGcGGCgcCTTCATcGATCCCACCTTCTTCCTGAGCCgAaCgGTCTCCAATGTCATCAGCTCCATTGTCTTcGGGgACCGCTTTGACTAcGAGGACAAAGAGTTCtTGTCACTaCTGCGaATGATGCTGGGAAgCTTCCAGTTCACcGCTACgTCTACcGGGCAGCTCTAcGAGATGTTCTACTCAGTGATGAAAtAtCTGCCAGGGCCACAGCAACAGGCCTTTAAGGAGCTGCAGGGaCTGGAGGACTTCATAGCCAAGAAGGTGGAaCAaAACCAgCGCACgCTGGAcCCCAACTCCCCaCGGgACTTCATCGACTCCTTCCTCATCCGCATGCAGCAGGAgaAGgaGAAtCCcAACACGGAGTTCTACagGAAGAACCTGGTGATgACaACacTGAACCTCTTCtTTGCgGGCACcGAgACGgTCAGCACgACCaTGCGgTATGGCTTCCTGCTGCTcATGAAGCACCCAGATgTgGAAGCCAAaaTcCATGAGGAGATTGAcCGcGTGATcGGCAAGAACCGTCAGCCCAAGTTTGAGGACCGaGCCAAGATGCCCTACACAGAGGCtGTGATCCACGAgATCCAgAGATTcGGAGAcaTGATCCCCATGgGCtTGGCtCGcAGAGTcACCAAGGATACCAAGTTTCGaGaCTTCCTgcTCCCCAAGGGCACcGAAGTGTTCCCTATGCTGGGCTCtGTgCTGAGAGACCCCAAGTTCTTCTCCAAcCCCCgaGATTTCAAtCCCCAGCACTTCCTGGAcGAGAAGGGGCAaTTTAAGAAGAGTGATGCTTTTGTGCCcTTCTCCATcGGTAAGCGGctgTGCcTgGGcGAgGcgCTGGCgcGcATGGAGCTCTTcCTCTaCCTCACCgCCATCcTGCAGAgcTTC

>CYP2A13_dog

ATGtTGGCCTCAGGGCTtCTTCTGGTGGCTTTGCTGgCCTGCCTGACaaTAATtGTcTTGATGTCTGTCTGGaaGCAGAGgAAaCTggGGGGgAaGCTcCCTCCaGGACCCACCCCAcTGCCCTTCATCGGaAAtTACCTGCAGCTGaACACAGAGCaGATgTacaACTCtCTCATGAAGATcaGCgAGCGCTATGGcCCgGTGTTCACcaTcCAtCTGGGGCCCCGgCctGTCGTGGTGCTgTGTGGAcACGAgGCgGTGAAGGAGGCTCTGgTGGACCAGGCgGAGGAATTCAGcGGgCGAGGcGAGCAGGCCACaTTtgACTGGcTCTTCAAAGGCTATGGTGTGGCgTTCAGCAATGGGGAGCGGGCCAAGCAGCTCAGGCGCTTCTCCATCACCACACTGcGGGACTTtGGAGTGGGaAAGCGcGGCATTGAaGAGCGCATtCAGGAGGAGGCaGGCTTCCTCATtGAGGCCcTCCGGgGCACacGaGGtgcCTTCATcGATCCCACCTTCTTCCTGAGCCgAaCaGTgTCCAATGTCATCAGCTCCATTGTtTTTGGGgAtCGCTTTGACTATGAGGACAAAGAGTTCCTGTCACTGCTGCGTATGATGCTGGGgAgCTTCCAGTTCACAGCTACATCTAtGGGGCAGCTCTATGAGATGTTtTACTCAGTGATGAAACACCTGCCAGGGCCACAGCAACAGGCaTTTAAGGAGCTGCAGGGtCTGGAaGACTTCATAaCCAAGAAaGTGGAGCAGAACCAACGCACaCTGGATCCCAACTCCCCtCGagACTTCATtGACTCCTTCCTtATCCGCATGCAGCAGGAgCAGAacAACCCcAACACGGAGTTtTACTTGAAGAACCTGGTGtTgACCACacTGAACCTCTTCtTTGCgGGCACtGAgACagTCAGtACAACCCTGCGgTATGGCTTCCTGCTGCTcATGAAGCACCCAGATgTgGAAGCCAAaGTcCATGAGGAGATTGATCGgGTGATTGGCAAGAACCGTCAGCCCAAGTTTGAGGACaGGGCCAAGATGCCCTACACAGAGGCAGTGATCCAtGAgATCCAAAGATTTGGAGAcaTGATCCCCATGgGCgTGGCCCGcAGAGTcAtCAAGGAcACCAAGTTTCGGGagTTtCTCcTCCCCAAGGGCACTGAAGTGTTCCCTATGCTGGGCTCtGTgCTGAGAGAtgCCAAGTTCTTCTCCAAcCCCCAaGAcTTCcACCCtCAGCACTTCCTGGATGAGAAGGGGCAGTTTAAGAAGAGTGATGCTTTTGTGCCcTTCTCCATTGGTAAGCGaTACTGtTTTGGAGAAGGCCTGGCTAGgATGGAGCTCTTTCTCTTCCTCACCACCATCtTGCAGAAcTTCcaCTTCAaGTcCCCgCAGctGCCtCAaGAcATCGAtGTGTCCCCcAAACAtGTGGGtTTTGCgAcCATcCCAcGAAAtTACACCATGAGCtTCCAGCCCCGCTgA

>CYP2A13_pig

ATGCTGGCCTCAGGctTGCTTCTcGTGGCTcTGCTGaCCTGCCTGACcaTAATGGTcTTGATGTCcGTCTGGCGcCAGAGgAAGCTCcaGGGgAaaCTGCCcCCcGGACCCACCCCgcTGCCCTTCATCGGGAACTACCTGCAGCTGaACACgGAGCaGATgTacaACTCCCTCATGAAGATcaGCcAGCGCTATGGcCCtGTGTTCACcGTcCACCTGGGGCCCCGgCGGaTaGTGGTGCTgTGTGGATACGAcGCgGTGAAGGAGGCcCTGgTGGACCAGGCTGAGGAATTCAGcGGgCGAGGcGAGCAGGCCACtTTCgACTGGcTCTTCAAAGGCTATGGTGCAGCTCTATGAGATGTTCTACTCgGTGATGAAACACCTGCCAGGGCCgCAGCAACAGGCaTTTAAGGAcCTGCAGGGgCTGGAGGACTTCATAGCCAgGAAGGTGGAaCAcAACCAgCGCACgCTGGATCCCAACTCCCCGCGagACTTCATCGACTCCTTCCTCATCCGCATGCAGCAGGAgaAGAaGAAtCCTgACACcGAGTTCTAtTgGAAGAACCTGGTtcTgACCACacTGAACCTCTTCtTcGCgGGCACcGAgACGgTCAGCACAACgaTGCGcTAcGGCTTCCTGCTGCTcATGAAGCACCCgGATgTgGAAGCCAAaGTcCAcGAGGAGATTGAcCGcGTGATcGGCAgGAACCGcCAGgCCAAGTTcGAGGACCGGGCCAAGATGCCCTACACgGAGGCcGTGATCCACGAgATCCAgAGATTcGGAGAcaTGATCCCCATGgGCCTGGCCCGaAGAGTcACCAAGGATACCAAGTTTCGGGaCTTCCTCcTCCCCAAGGGCACaGAgGTcTTCCCccTcCTtGGCTCCaTCCTGcatGACCCCgAGaTCTTCgaacAgCCCgAgGAgTTCAACCCaggcCgaTTCCTGGATGcagAcGGaaAGTTccAGAAGcagGcgGCcTTccTGCCcTTCTCCAcaGGcAAGCGcattTGtcTTGGgGAAGGCCTGGCccGcAccGAatTaTTcCTCTTCtTCACCACCATCcTcCAGAAcTTCTcCgTggccagCCCcgtGgCtCCggAGGAcATCGACcTGaCaCCccAggctGTtGGg

>CYP2A13_elephant

ATGCTGGCCTCAGGGaTGCTTCTGGTGGCTTTGCTGgCCTGCCTGtCTgTAATGGTcTTGATGTCTGTCTGGCGaCAaAGgAAGCTCTGGGGgAaGCTtCCcCCTGGgCCCACtCCcTTGCCtTTCATCGGGAACTACCTGCAGCTGaACACAcAGCaGATgTacaACTCCCTCATGAAGAGCGCTATGGcttgGTGTTCACgGTcCACtTGGGGtCCCGgCGGGTtGTGGTaCTgTGcGGATACGAcGCTGTGAAGGAGGCTCTGgTGGACCAGGCTGAGGAATTCAGTGGgCGAGGcGAGCAGGCCACCTTCgACTGGcTCTTCAAAGGCTAcGGTGTGGCgTTCAGCAAcGGGGAGCGaGCCAAGCAGCTCcGGCGCTTCTCCATCACCACgCTGcGGGACTTCGGcGTGGGaAAGCGTGGCATTGAGGAGCGtATCCAGGAGGAGGCcGGCTTCCTCATCGAGaCCTTCCGGgGCACgcGcGGCAcCTTCATTGATCCCACCTaCTTCCTGAGCCgAaCaGTCTCCAATGTCATCAGCTCCATTGTCTTcGGagACCGgTTTGACTATGAaGAtAAAGAGTTCCTtTCACTGCTGCGTATGATGCTGGGAAgCTTCCAGTTCACAGCTACcgCTACcGGaCAGCTCTATGAcATGTTCTACTCgGTaATGAAAtACCTaCCAGGGCCACAGCAACAGGCCTTTAAGGAGCTGCAGGGgCTGGAGGACTTCATAaCCAAGAAGGTGGAGCAGAACCAgCGCACaCTGGAcCCCAACTCCCCaCGGgACTTCATtGACTCCTTCCTCATCCGCATGCgGCAGGAgaAGAaGAACCCcAACACaGAGTTCTACaTGAAGAACCTGGTGcTgACCACGcTaAACCTCTTCtTTGCgGGCACAGAgACcgTCAGCACAACCCTGCGTTAcGGCTTCCTGCTGCTcATGAAGCACCCAGATAGCCAAGtTgCAcGAGGAGATTGAcCagGTGATTGGCAAGAACCGTCAGCCCAAGTTTGAGGACCGGGCCAAGATGCCCTACACcGAGGCtGTGATCCAtGAgATCCAgAGATTTGcAGAcaTGATCCCCATGgGggTGGCCCGcAGgGTcACCAAGGAcACCAAGTTTCGGaaCTTCtTCATCCCCAAGGGCACaGAAGTGTTCCCTATGCTGGGCTCtGTgCTGAGAGACaCCAAGTTCTTCTCCAAcCCCCAaGATTTCAACCCCCgGCACTTCCTGGATGAGAAaGGGCAaTTTAAGAAGAacGAcGCcTTTGTGCCcTTCTCCATTGGTAAGCGGTACTGtTTcGGgGAAGGtCTGGCcAGAATGGAGCTCTTTgTCTTCCTtACCACCATCtTGCAGAAcTTCTGCTTCAaGTcCCCgCAGTCGCCCaAGGAcATCGAtGTGTCtCCcAAACACGTGGGCTTTGCCActATcCCAcGAAcCTACACCATGAGCtTCttGCCCCGCTgA

>CYP2A13_human

ATGCTGGCCTCAGGGCTGCTTCTGGTGaCcTTGCTGgCCTGCCTGACTgTgATGGTcTTGATGTCaGTCTGGCGGCAGAGgAAGagCaGGGGgAaGCTGCCTCCgGGACCCACCCCATTGCCCTTCATtGGaAACTACCTGCAGCTGaACACAGAGCaGATgTacaACTCCCTCATGAAGATcaGtgAGCGCTATGGcCCtGTGTTCACcaTTCACtTGGGGCCCCGgCGGGTCGTGGTGCTgTGcGGAcAtGATGCcGTcAAGGAGGCTCTGgTGGACCAGGCTGAGGAgTTCAGcGGgCGAGGcGAGCAGGCCACCTTCgACTGGcTCTTCAAAGGCTATGGTGTGGCgTTCAGCAAcGGGGAGCGcGCCAAGCAGCTCcGGCGCTTCTCCATCgCCACcCTaAGGGgtTTtGGcGTGGGcAAGCGcGGCATcGAGGAaCGCATCCAGGAGGAGGCGGGCTTCCTCATCGAcGCCcTCCGGgGCACGcacGGtATcGATCCCACCTTCTTCCTGAGCCgcaCaGTCTCCAATGTCATCAGCTCCATTGTCTTTGGGgACCGCTTTGACTATGAGGACAAAGAGTTCCTGTCACTGtTGCGcATGATGCTGGGAAgCTTCCAGTTCACgGCaACcTCcACGGGGCAGCTCTATGAGATGTTCTctTCgGTGATGAAACACCTGCCAGGaCCACAGCAACAGGCCTTTAAGGAGCTGCAaGGgCTGGAGGACTTCATcGCCAAGAAGGTGGAGCAcAACCAgCGCACgCTGGATCCCAAtTCCCCaCGGgACTTCATCGACTCCTTtCTCATCCGCATGCAGCAGGAggAGAaGAACCCcAACACaGAGTTCTACTTGAAGAACCTGGTGATgACCACccTGAACCTCTTCtTTGCgGGCACtGAgACcgTgAGCACcACCCTGCGcTAcGGtTTCCTGCTGCTcATGAAGCACCCAGAggTgGAAGCCAAGGTcCATGAGGAGATTGAcaGAGTGATcGGCAAGAACCGgCAGCCCAAGTTTGAGGACCGGGCCAAGATGCCCTACACAGAGGCAGTGATCCACGAgATCCAAAGATTTGGAGAcaTGcTCCCCATGgGttTGGCCCacAGgGTcAaCAAGGAcACCAAGTTTCGGGatTTCtTCcTCCCCcAGGGCACTGAAGTGTTCCCTATGCTGGGCTCCGTgCTGAGAGACCCCAgGTTCTTCTCCAAcCCCCggGAcTTCAAtCCCCAGCACTTCCTGGATaAGAAGGGGCAGTTTAAGAAGAGTGATGCTTTTGTGCCcTTtTCCATcGGTAAGCGGTACTGtTTTGGAGAAGGCCTGGCcAGAATGGAGCTCTTTCTCTTCtTCACCACCATCATGCAGAAcTTtcGCTTCAaGTcCCCTCAGTCGCCtaAGGATATCGACGTGTCCCCcAAACACGTGGGCTTTGCCAcgATcCCAcGAAACTACACCATGAGCtTCCtGCCCCGCTgA

>CYP2A13_sheep

ATGCTGGCCTCAGGGCTGCTcCTcGTGGCTTTGCTGgCCTGtCTGACcaTcATGGTcTTGATGTCTGTCTGGCGGCAaAGgAAcCTCaaaGGgAaatTGCCTCCaGGgCCCACtCCtcTGCCCTTCATCGGGAACTACCTGCAGCTGaACACcGAGCaGATgTacaACTCCCTCATGAAGATcaGCgAGCGCTAcGGcCCcGTGTTCACgaTcCgCCTGGGGaCCCGgCaGaTCGTGGTGCTgTGcGGcTAtGATGCTGTGAAGGAGGCTCTGgTGGACCAGGCTGAaGAATTCAGTGGgCGAGGAGtGCAGGCtACCTTtgACTGGcTCTTCAAAGGCTATGGTGTGGCtTTCAGCAAcGGGGAGCGcGCCAAGCAGCTCcGGCGCTTCTCaATtACgACgCTGcGGGACTTCGGcGTGGGcAAGCGcGGCATcGAaGAGCGCATCCAGGAGGAGGCGGGCTTCCTCATCGAGGCCTTCCGGgGCACtcGcGGCAcCTTCATcGATCCCACCTTCTTCCTGAGCCgAaCgGTCTCCAATGTCATCAGCTCCATTGTCTTcGGGgACCGCTTTGACTAcGAGGACAAAGAGTTCCTGTCACTaCTGCGaATGATGCTGGGAAgCTTCCAGTTCACcGCTACgTCTACcGGGCAGCTCTAcGAGATGTTCTACTCAGTGATGAAAtAtCTGCCAGGGCCACAGCAgCAGGCCTTTAAGGAGCTGCAGGGaCTGGAGGACTTCATAGCCAAGAAGGTGGAaCAaAACCAgCGCACaCTGGAcCCCAACTCCCCaCGGgACTTCATCGACTCCTTCCTCATCCGCATGCAGCAGGAgaAGgaGAAtCCcAACACGGAGTTCTACaTGAAGAACCTGGTGcTAACaACccTGAACCTCTTCtTTGCgGGCACtGAgACGgTCAGCACgACCaTGCGgTATGGCTTCCTGCTGCTcATGAAGCACCCAGATgTgGAAGCCAAaaTcCATGAGGAGATTGAcCGcGTGATcGGCAAGAACCGTCAGCCCAAGTTTGAGGACCGGGCCAAGATGCCCTACACAGAGGCtGTGATCCACGAgATCCAgAGATTcGGAGAcaTGATCCCCATGgGCCTGGCCCGcAGAGTcACCAAGGATACCAAGTTTCGaGaCTTCCTCcTCCCCAAGGGCACcGAAGTGTTCCCTATGCTGGGtTCCGTgCTGAGAGACCCCAAGTTCTTCTCCAAcCCCCgaGATTTCAAtCCCCAGCACTTCCTGGAcGAGAAGGGGCAaTTTAAGAAGAGTGATGCTTTTGTGCCcTTCTCCATcGGTAAGCGGTACTGtTTcGGAGAAGGCCTcGCcAGAATGGAGCTCTTcCTCTTCtTCACCACCATCATGCAGAAcTTCcGCTTCAaGTcCCCaCAGTCGCCCCAGGAcATCaAtGTGTCCCCcAAACtCGTGGGCTTTGCCActATcCCtccAAACTACACCATGAGCtTCCtGCCCCGtTgA

>CYP2A13_mouse

ATGCTGaCCTCAGGaCTcCTcCTGGTGGCTgcagTGgCCTtCCTcAgcgTccTGGTcTTGATGTCTGTCTGGaaGCAGAGAAAGCTCTcaGGAAaGCTGCCTCCaGGACCCACCCCAcTGCCCTTCATtGGGAACTtCCTtCAGCTGaACACAGAGCaaATgTacaACTCtCTCATGAAGCGtTATGGtCCtGTaTTCACcaTctACCTGGGaCCtCGCCGaaTtGTGGTGCTgTGcGGAcAgGAgGCaGTcAAGGAaGCTCTGgTGGACCAaGCTGAGGAATTCAGcGGgCGgGGcGAGCAaGCtACCTTCgACTGGcTtTTCAAAGGCTATGGTGTaGCcTTCAGCAgcGGGGAGCGaGCCAAaCAGCTaAGGCGCTTCTCCATCgCCACgCTGcGGGACTTCGGcGTGGGgAAGCGTGGCATcGAGGAGCGCATCCAaGAGGAGGCGGGCTTtCTCATCGAttCaTTtCGGAagACGaacGGtgctTTtATTGAcCCCACCTTCTaCCTtAGCCgAaCaGTCTCCAATGTCATtAGCTCaATTGTCTTcGGGgACCGCTTTGACTATGAGGACAAAGAGTTCCTGTCACTGCTtCGaATGATGCTGGGAAgCTTCCAGTTCACtGCTACcTCcAtGGGGCAGCTCTATGAGATGTTCTctTCtGTGATGAAACACCTGCCAGGGCCcCAGCAACAGGCCTTTAAGGAGCTGCAGGGCCTGGAGGACTTCATAaCCAAGAAaGTGGAaCAcAAtCAgCGCACgCTGGATCCCAAtTCCCCaaGGgACTTCATCGACTCCTTCCTCATCCGaATGCtGCAGGAgaAGAaGAACCCcAAtACtGAGTTCTACaTGAAGAACtTGGTGcTgACtACacTaAAtCTCTTCtTTGCTGGCACAGAgACcgTCAGCACcACCCTGCGcTATGGCTTtCTGtTGCTcATGAAGCACCCAGATATtGAAGCCAAGGTcCATGAGGAGATTGATCGgGTGATTGGCAgGAACCGgCAGCCCAAGTaTGAGGACCGaatgAAGATGCCCTAtACgGAGGCtGTaATCCAtGAgATCCAgAGATTTGcAGAcaTGATCCCCATGgGCCTGGCtCGaAGgGTcACCAAGGAcACCAAGTTTCGaGatTTCCTCcTCCCCAAGGGtACTGAAGTGTTtCCTATGCTGGGCTCtGTgCTGAaAGACCCCAAGTTCTTCTCCAAcCCCaAaGAcTTCAACCCaaAGCACTTCCTaGATGAcAAGGGaCAGTTTAAGAAGAaTGATGCcTTTGTGCCcTTtTCCATTGGTAAGCGGTAtTGtTTcGGAGAAGGaCTGGCTAGgATGGAaCTCTTcCTCTTCCTCACaAaCATCATGCAGAAcTTCcaCTTCAaaTcCaCaCAGgCaCCCCAGGAcATCGAtGTGTCtCCtAgACtCGTGGGCTTTGCCAcgATcCCAccAAcCTACACtATGAGttTCttGtCCCGtTgA

>CYP2C113_horse

ATGGATCTGGTTGTGGTCCTGGGGCTCTGTCTCTCCTGTTGGCTTCTCCTTTCACTCTGGAAACAGAGCTCCAGGAAAGGGAAGCTCCCACCTGGCCCCACTCCTCTCCCTATTATTGGAAATATCCTGCAGTTAGATGTTAAGAACATCAGCAAATCCTTAACCAATCTCTCAAAAGTCTATGGCCCTGTGTTTACTCTGTATATTGGCATGAAGCCCGCTGTGGTGTTGCATGGATATGAAGCAGTGAAGGAATCCCTGATTGATCTGGGGGAGGAGTTTTCTGGAAGAGGCAATTCCCCAGGGTCTGAAAGAGCTAATAAAGGACTTGGAATCGTTTTCAGCAATGGAAAGATATGGAGGGAGATGCGGCGTTTCTCCCTCATGACTCTGCGGAATTTTGGGATGGGGAAGAGGAGCATTGAGGACCGAGTTCAAGAGGAAGCCCGCTGCCTTGTGGAGGAGTTGAGAAAAACCAATGCTTCACCCTGTGATCCCACTTTTATCCTGGGCTGTGCTCCTTGCAATGTGATCTGCTCCATCATTTTCCAGAATCGTTTTGATTATACAGATCAGAATTTTCTAAATATAATGAAAAAATTTGATGAAAATGTCAGGATCGGTAGCTCTCCATGGATGCAGCTCTGCAATCATTTCCCTGTTCTCTTAGATTATTTCCCAGGGAGTCATAACAAATTGCTTAAAAATATTGCATATATAAACAATTATGTTTTGGAGAAAACAAAGGAACACCAAGAATCCCTGGACATTAACAACCCTCGGGACTTTATTGATTGTTTCCTGATCAAAATGGAGCAGGAAAAAGAAAATAAACAGTCAGAGTTTACTTTTGAAAACTTGACAATCACTGTGTCTGACTTGTTTGGAGCTGGGACAGAGACAACGAGCACCACCCTGAGATATGGACTTCTCCTCCTGCTGAAGCACCCAGAGGTCACAGCTAAAGTCCAGGAAGAGATTGATCGTGTGATTGGCAGACACCGAAGCCCCTGCATGCAGGACAGGAGCCACATGCCCTATATGGATGCCGTGATACATGAGGTCCAGAGATACATTGACCTCATCCCCACCAACCTGCCCCATGCAGTGACCCGTGACGTTAAATTCAGAAACTACCTCATCCCCAAGGGCACAAACATATTAATATCACTGACTTCTGTGTTGCATGATGATAAAGAATTCCCTAACCCAGAGGTGTTTGACCCTGGCCACTTCCTGGACAAGAGTGGCAACTTTAAGAAGAGTGACTATTTCATGGCTTTCTCAGCAGGAAAACGGATTTGTGTGGGAGAGGGCCTGGCCCGCATGGAGCTGTTTTTATTCCTGACAACCATTTTACAGAAATTTAACTTGAAGTCTCTGGTTGACCCAAAAGACATCGACGTCACCCCAGTTGTCAATGGATTTGCTTCTGTGCCACCCTTTTACAAGATCTGTTTCATTCCTGTGTGA

>CYP2C113_rhino

ATGGATCcaGcTGTGGTCCTGGtGCTCTGTCTCTCCTcTTtcCTTCTCCTTTCcCTCTGGAAACAGAGCTCCAGGAAAGGGAAGCTCCCgCCTGGCCCCACTCCTCTCCCTATTATTGGAAATATCCTGCAGTTAGATaTTAAGAACATCAGCAAATCCTTAACCcAgCTCTCAAAAGTCTATGGCCCTGTGTTcACTCTGTATtTgGGCtTGAAGCCCaCTGTGGTGcTGCATGGATATGAAGCAGTGAAGGAAgCCCTGATTGATtTGGGGGAGGAGTTTTCTGGAAGAGGCAgTTtCCCAGcGgCTGAAAGAGCTAATAAAGGACTTGGAATCaTTTTCAGCAATGGAAAGAggTGGAaGGAaATcCGGCGcTTCTCCCTCATGACcCTGCGGAATTTcGGGATGGGGAAGAGGAGCATTGAGGACCGAGTTCAAGAGGAAGCCCGCTGCCTTGTGGAGGAGTTGAGAAAAACCAATGCTaCACCCTGTGATCCCACTTTTATCCTGGGCTGTGCTCCcTGCAATGTGATCTGCTCCATtATTTTCCAcAAcCGTTTTGATTATACAGATgAGAATTTTCTAAAcATAgTGgAAAtATTTGAcGAAAATGTCAaGATtatcAGtTCcCCATGGATGCAGCTCTGCAATagTTTCCCTaTggTCcTtGATTATTTCCCAGGGAGTCAcAAaAAATTatTTAAAAATgTTGCtTATgTAAAaAgTTATGTTTTaGAGAAAgCAAAGGAACACCAAGcATCtCTGGACATTAACAAtCCTCGaGACTTTATTGATTGTTTCCTGATCAAAATGGAGCAGGAAAAgcAAAATcAgCAGTCAGAGTTTACcTTgGAAAAtTTGACAgTCACTGcaTtTGAtTTGTTTGGAGCTGGGACAGAGACAACaAGCACtACCCTGAGATATGctCTcCTCCTCCTGCTGAAGCACCCAGAGGTCACAGCTAAAGTCCAGaAAGAGATTGAcCaTGTGATTGGCAGACACCGgAGCCCCTGCATGCAGGAtAaGAGCCAtATGCCCTAcATGGATGCtGTGgTACATGAGaTCCAaAGATACATTGACCTCATCCCtACCAACCTGCCCCATGCAGTGACtCGTGACaTTAAATTCAGAAACTACCTCATCCCCAAGGGCACgAcCATATTAAcATCtCTGACTTCTGTGcTGCATGATGATAAAGAATTCCCcAACCCAGgGGTGTTTGACCCTGGCCACTTCCTGGACAAGAGTGGCAACTTTAAGAAGAGTGACTATTTCATGcCTTTtTCAGCAGGAAAACGGATaTGTGcGGGAGAGGGaCTGGCCCGCATGGAGCTGTTTTTATTCCTGACcACCATTTTACAGAAATTTAcCcTGAAaTCTgTGGTTGAtCCAAAgGACATCGACacCACtCCAGcTGcCAATGGgTTTGCTTCTGTGCCACCtTcTTAtcAGcTCTGcTTCATTCCTGTcTGA

>CYP2C113_cow

ATGGATCTGGcTGTGGTCCTGGtGCTCTGTCTCTCCTGTctGCTTCTCCTcTCACTCTGGAAACAGAGCTCtgGGAAAGGGAAGCTaCCgCCgGGCCCCACTCCTCTCCCgATTcTTGGAAATATCCTaCAGTTAGATGTTAAGAACATtgGCAAATCCTTAAgCAATCTCTCAAAcacCTATGGCCCTGTGTTcACagTGTATtTTGGCtTGAgGCCCaCTGTtGTGTTGCATGGgTATGAAGCAGTGAAaGAAgCCCTaATTGATCaGGGaGAaGAaTTTTCTGGAAGAGGCAATatCCCAatGTCTcAAAGAGtTAATAAAGGAtaTGGAATCaTTTTCAGCAAcGGgAAGAgATGGAaGGAGATcCGaCGcTTCTCCCTCATGACcCTGCGGAATTTTGGGATGGGGAAGAGGAGCATcGAGGACCGcGTTCAAGAaGAAGCgCaCTGCCTgGTGGAGGAGTTGAGAAAAACCAATGCTTCACCCTGTGATCCCACTTTcATCCTGGGCTGcGCTCCcTGCAATGTGATCTGCTCCATtATTTTCCAaAATCGgTTTGATTATACAGATCAGAATTTTCTtAActTgcTagAcAAATTTaATGAAAATcTCcaGgTtGtgAGCTCcCCATGGATGCAGTCTGCAAcacTTTCCCTaTTCTCaTcGAcTATTTCCCAGGGAGTCATAACAAAcTatTTAAAAATtTTGCtTATATAAAaAATTATGTTTTGGAGAAAACAAgGGAACACCAAGcATCCCTGGAtATTAACAAtCCTCGaGACTTTATTGATTGcTTCCTGATCAAgATGGAGCAGGAAAAAcAcAATcAtCAGTCAGAaTaTACaTTTGAAAACcTGACAATCACTGTaTCTGAtTTGTTTGGAGCTGGGACAGAGACAACGAGCACCACaCTGAGATAcGGACTcCTCCTCCTGCTGAAGCACCCcGAaGTCACAGCTAAgGTCCAGGAAGAGATTGAcCaTGTcATTGGCAGACACCGgAGCCCCTGCATGCAGGACAaGAGCCACATGCCCTAcAcaGATGCtGTGgTcCAcGAGaTCCAGAGATACATTGACCTggTCCCCACCAACCTGCCCCATGCgGTGACCtGTGACaTTAAATTCAGAAACTACCTCATCCCaAAGGGCACAggtATATTAAcATCACTGACTTCTGTGcTatATGATGAcAAAGAATTtCCcAACCCAGAGGTGTTTGACCCTGGCCACTTtCTGGAtgAGAGTGGCAACTTTAgGAAGAGTGACcATTTCATGGCTTTCTCAcCAGGgAAACGGATTTGTGTGGGAGAGGGCCTGGCCCGCATGGAGCTGTTTTTATTCCTGACcACaATTTTACAaAAcTTTAcCTTGAAaTCTgTGGTTGACCCAAAgGACcTCGACacCACCCCAGTTGTCAATGGgTTactTTCTGTGCCACCtTTTTACcAGcTCTGTTTCATTCCTGTGTGA

>CYP2C113_dog

ATGGATCTctTcaTaGTtCTGGtGaTaTGTCTtTCtTGTTtGaTTtcttTcTttCTgTGGAAtCAaAatcgtgccAAAGGGAAGCTgCCACCTGGCCCCACTCCTCTCCCaATcATTGGAAATATtCTaCAGaTAaATacTAAGAAtgTCAGCAAATCCcTAtTATGGCCCTGTGTTcACTgTGTATtTTGGCATGAAGCCtaCcGTGGTGTTGtAcGGgTATGAAGCgGTGAAGGAAgCtCTaATTGATCgGaGtGAaGAGTTTTCaGGcAGAGGCcATTtCCCAttGTtGGAATtGTTTTCAGCAAcGGAgAaAaATGGAaGcAaAccCGGCGTTTtTCCCTgAcagtTtTGCGGAATaTgGGGATGGGaAAGAaGActgTTGAaGACaGAaTTCAAGAaGAgGCCttgTatCTaGTGGAaGcaTTaAaAAAAACCAAcGCaTCtCCCTGTGATCCtACTTTccTtCTGGGCTGTGCTCCcTGCAATGTGATtTGCTCCATtATTTTCCAGAATCGTTTTGAgTATgatGATaAagATTTTtTACAGCTCTaCAATgcTTTCCCTcTTtTaaTAcATTAccTtCCAGGaAGTCATcAtgtgTTatTTAAAAAcATTGCtaAccaAtttAAgTtTaTTTcGGAaAAAAtAAAaGAACACgAAGAATCtCTaaACtTTAgtAACCCTCGGGACTTTATTGAcTacTTCCTGATCAAAATaGAaCAGGAAAAAcAcAAcAAACAGTCtGAaTTTACcaTgGAcAACTTGAtcATtACcaTaTggGAtgTGTTTaGtGCgGGaACAGAGACAACGAGCACCACCCTGAGATAcGGACTatTggTgCTatTaAAGCACCCAGAtGTCACAGCTAAAGTCCAGGAAGAGATTcATCGTGTagTTGGCAGACAtCGgAGCCCCTGCATGCAaGAtAGGAGCtgCATGCCCTAcAcaGATGCtGTGgTACATGAGaTCCAGAGATACATTGAtCTtgTCCCCAaCAAtCTGCCCCATtCAGTGACtCagGACaTcAAgTTtAGAgAaTACCTtATtCCCAAGGGCACAAcCATATTAAcATCtCTGACTTCTGTccTGCATGATGAgAAAGgATTCCCcAACCCAGAtcaGTTTGAtCCTGGCCACTTCCTGGAtgAaAaTGGCAgCTTTAAGAAGAGTGACTAcTTCATGGCcTTCTCAGCAGGgAAgaGagTTTGTGTtGGAGAaGGCCTGGCCCGCATGGAGCTGTTTTTgcTaCTGACcAatATTTTACAGcAtTTcAcCTTGAAacCTCTGGTTGAtCCAAAgGACATtGACacCACCCCAaTTGcCAATGGgTTgGgTgCTacaCCACCtTccTAtAAGcTCTGTTTtgTTCCaGTcTGA

>CYP2C113_pig

ATGGATCTGGTTGTGGTCCTGGtGCTtaGTCTCTCCTGTctGCTTCTCCTTTCACTCTGGAAAgAGAGCTCtgGGAAAGGGgAGCTCCCACCTGGCCCCACTCCTCTCCCaATTcTTGGAAATATCCTaCAGTTgGATGTTAAGAACATCAGCAAATCCTTAACCcAgCTCgCAgAAGTCTATGGCCCTGTGTTcACcgTGTATtTTGGCATcAAGCCCaCTGTGGTGTTatATGGATATGAAGCAGTGAAGGAAgCtCTGgTTGATCTGGGaGAGGAGTTTTCTGGAAGAGGCcATTtCCCAGtGgCcGAAAaAaCTcAcAAAGGACTTGGAATCaTcTTCAGCAATGGAAAGAaATGGAaGGAaATcCGGCGcTTCTCCCTCATGACctTGCGGAATTTaGGGATGGGGAAaAGGAGCATTGAGGAtCGcaTTCAgGAGGAAGCCtGCTGCCTTGTGGAGGAGTTaAGAAAAACCAATGCcTCACCCTGTGATCCCACTTTTATCCTGGaCTGTGCTCCcTGCAATGTGATCTGCTCCATtATTTTCCAaAATCGTTTTGATTATACAGATCAGgATTTTCTtAcctTgtTGgAAAAATTccATGAAAAccTCAatATtctgAGCTCTCCATGGATcCAGCTCTGtAATaAcTTCCCTGcTCTCaTtGATTATTctCCAGGaAtTCATAACAAATTaCTTAAAAATATTGCtTATtTgAAaAgTTATGTTTTGaAGAAAgtAAAaGAACACCAAGAATCaCTGGACATTAACAAtCCTCGGGACTTcATTGAcTGTTTCCTGATCAAAATGGAaCAGGAAAAgcAcAATcAACAGTCtGAaTaTACaaTTGAAAACTTGAtAgcCACTGTaTCTGAtaTGTTTtctGCTGGtACAGAGACAACaAGCACCACCaTGAGATATGGACTcCTgCTtCTaCTGAAGCACCCAGAGGTCACAGCTAAgGTCCAGGAAGAGATTGAcCGTGTGATTGGCAGgCACCGgAGtgCCTGCATGCAGGACAGGAGCCACATGCCCTAcAcaGATGCtGTGgTACAcGAGaTCCAGAGATACATTGACCTggTCCCCACCAACCTGCCCCATGCtGTGACttGTGACaTTAAATTCAGAAACTACCTCATCCCCAAGGGCACAAcaATATTAAcATCttTGACTTCTGTGcTGtATGATtgcAAAGcATTCCCcAACCCAGAaGTGTTTGACCCTGGCCAtTTCCTaGAtgAGAGTGGtAACTTTAAGAAGAGTGACTAcTTCATGcCTTTCTCAaCAGGAAAACGGATTTGTGTGGGAGAGGGCCTGGCCCGCATGGAGCTaTTTTTATTCCTGACcACaATTTTACAGAAATTTAACcTGAAaTCTgTGGTTGAtCCAAAgGACATCGACacCACtCCAGTTGcCAATGGATTTGCTTCTGTGCCtCCCTTcTACcAGATCTGcTTCATTCCacTGTGA

>CYP2C113_elephant

ATGGATCTaGccGTGGTCCTcGtGCTCTGTCTCTCCaGTTtGCTTCTCtTTTCACTCTGGAAACAGAGCTaCgGGAAAGGGAAGCTCCCgCCTGGCCCCACTCCTCTCCCaATTATTGGAAATAgCCTGCAGTTAaATtTTAAGAAtATCAGCAAATCtTTaCTCTCAAAAacCTATGGCCCTGTGTTcACcCTGTATtTgGGCATGAAGCCCaCaGTGGTGTTGCATGGgTATGAAGCAGTGAAGGAAgCCCTGATTGATCaGGGaGAGGAGTTTTCTGGAAGAGGCAgTTtCCCAGtGTCTGAAAaAGtTcATAAAGGACTTGGAgTCGTTTTCAGCAATGGAAAGATgTGGAaGGAGAccaGGCGcTTCTCCCTCATGACcCTGCGGAATTTTGGGATGGGGAAGAGGAGCATTGAGGACCGtGTTCAgGAGGAAGCCtGCTGCCTaGTGGAGGAGTTGAGAAAAACCAAGCcTCACCCTaTGATCCtACTTTcATCCTGGGCTGTGCTCCcTGCAATGTGATCTGCTCCATtATTTTCCAGAATCGTTTTGATTATACAGATCAGgATTTTCTgAActTgATaggAAAATTgaATGAAAAcaTacaGATtctgAGCTCcCCATGGgTcCAGCTCTGCAATagTTTtCCctTatTCaTtGATTATTTgCCAGGGAGTCATAACAAAcTctTTAAtAATATTGCtTATtcAAAaAgTTAcaTTTTGGAGAAAAtgAAaGAACACCAgGAAaCCtTGGACATTAACAAtCCTCGGGACTTcATTGATTGTTTCCTGATCAAAATGGAaCAGGAAAAgGAcAATcAAgAGTCtGtaTTTACTaTTGAAAACTTGAtAATCACTaTaagTGAtgTGTTTtctGCTGGGACAGAGACAACaAGtACCACaCTGAGgTATGGACTcCTgCTtCTGCTaAAGCACCCAGAGGTCACAGCTAAAGTCCgGGAAGAGATTGAcCGTGTGgTTGGCcGACACCGgAGCCCCTGCATGCAGGACAGGAGCagCATGCCCTAcAcGGATGCtGTGgTgCAcGAGaTCCAGAGATACATTGACCTtATCCCCACCAACCTGCCCCATGCgGcGACCCaaGACaTTAAATTCAGgAACTACCTCATCCCCAAGGtATATTAAcATCACTGACTTCTGTGcTtCAcGATaAcAAAGAATTCCCcAACCCAGAGacaTTTGACCCTGctCACTTtCTGGAtgAGAGTGGCAACTTTAAGAAGAGcGACTAcTTCATGGCTTTtTCAGCAGGAAAACGtATTTGTcTGGGAGAGGGCCTGGCCCGCATGGAGCTGTTTTTATTCtTcACtgCCATTTTACAGAAcTTTAcCTTGAAGcCTCTGGTTGACCCAAAgGACATCGAtacCAaCCCAGTTGaaAAaGGgTTTGCcTCTGTGCCACCCaagTACcAGcTCTGcTTtATTCCTGTGTGA

>CYP2C113_human

ATGGATCcaGcTGTGGctCTGGtGCTCTGTCTCTCCTGTTtGtTTCTCCTTTCACTCTGGAggCAGAGCTCtgGaAgAGGGAgGCTCCCgtCTGGCCCCACTCCTCTCCCgATTATTGGAAATATCCTGCAGTTAGATGTTAAGgACATgAGCAAATCCTTAACCAATgTCTCAAAAGTCTATGGCCCTGTGTTcACTgTGTATtTTGGCcTGAAGCCCatTGTGGTGTTGCATGGATATGAAGCAGTGAAGGAggCCCTGATTGATCatGGaGAGGAGTTTTCTGGAAGAGGaAgTTttCCAGtGgCTGAAAaAGtTAAcAAAGGACTTGGAATCcTTTTCAGCAATGGAAAGAgATGGAaGGAGATcCGGCGTTTCTgCCTCATGACTCTGCGGAATTTTGGGATGGGGAAGAGGAGCATcGAGGACCGtGTTCAAGAGGAAGCCCGCTGCCTTGTGGAGGAGTTGAGAAAAACCAATGCcTCACCCTGTGATCCCACTTTcATCCTGGGCTGTGCTCCcTGCAATGTGATCTGCTCtgTtATTTTCCAtgATCGaTTTGATTATAaAGATCAGAggTTTCTtAActTgATGgAAAAATTcaATGAAAAccTCAGGATtctgAGCTCTCCATGGATcCAGTCTGCAATaATTTCCCTGcTCTCaTcGATTATcTCCCAGGaAGTCATAAtAAAaTagcTgAAAATtTTGCtTAcATtAAaAgTTATGTaTTGGAGAgAAtAAAaGAACAtCAAGAATCCCTGGACATgAACAgtgCTCGGGACTTTATTGATTGTTTCCTGATCAAAATGGAaCAGGAAAAgcAcAATcAACAGTCtGAaTTTACTgTTGAAAgCTTGAtAgcCACTGTaaCTGAtaTGTTTGGgGCTGGaACAGAGACAACGAGCACCACtCTGAGATATGGACTcCTgCTCCTGCTGAAGtACCCAGAGGTCACAGCTAAAGTCCAGGAAGAGATTGAatGTGTagTTGGCAGAaACCGgAGCCCCTGtATGCAGGACAGGAGtCACATGCCCTAcAcaGATGCtGTGgTgCAcGAGaTCCAGAGATACATTGACCTCcTCCCCACCAACCTGCCCCATGCAGTGACCtGTGAtGTTAAATTCAaAAACTACCTCATCCCCAAGGGCACgAcCATAaTAAcATCcCTGACTTCTGTGcTGCAcaATGAcAAAGAATTCCCcAACCCAGAGaTGTTTGACCCTGGCCACTTtCTGGAtAAGAGTGGCAACTTTAAGAAaAGTGACTAcTTCATGcCTTTCTCAGCAGGAAAACGGATgTGTaTGGGAGAGGGCCTGGCCCGCATGGAGCTGTTTTTATTCCTGACcACCATTTTgCAGAAcTTTAACcTGAAaTCTCaGGTTGACCCAAAgGAtATtGACaTCACCCCcaTTGcCAATGcATTTGgTcgTGTGCCACCCTTgTACcAGcTCTGcTTCATTCCTGTcTGA

>CYP2C113_sheep

ATGGATCTGGcTGTGGTCCTGGtGCTCTGTCTCTCCTGcctGCTTCTCCTcTCgCTaTGGAAACAGAGCTCtgGGAAAGGGAAGCTCCCcCCaGGCCCCACTCCTCTCCCaATTcTTGGAAATATCtTtCAGcTAGATGTTAAGAAtATCAGCAAATCtTTAACCAgCTCTCAAAAGTCTAcGGaCCTGTGTTTACTgTGTATtTTGGaATGAAGCCCaCgGTaGTtTTGCATGGATATGAAGCAGTGAAGGAggCtCTGATTGATtTGGGaGAGGAGTTTTCTGGAAGAGGCAgTTtCCCAGtGatTcAAAGAaCTgtTAAgGGAtaTGGAATCGTaTTCAGCAAcGGgAAaAcATGGAaGGAGAccCGGCGcTTCTCgCTCATGACgCTGCGGAATTTcGGGATGGGGAAGAGGAGCATcGAGGACCGcGTTCAAGAGGAAGCgCGCTGCCTgGTGGAGGAGcTGcGgAAAACCAAcCCCTGTGATCCCACTTTcATCCTGGGCTGTGCTCCcTGCAATGTGATCTGCTCCATtATTTTtCAGAAcCGTTTTGATTATAaAGATCAGAgTTTTCTAAATcTAATGAAAAcAgTaaATGAAAATaTCAaGATtttggGCTCTCCATGGATGCAGCAATatTTTCCCTGTgCTCcTtGATTtTTTCCCAtGGAGTtATAAaAAATTttcTAcAAATATTGCtTATgTAAAaAATTATGTTTTGGAGAAAACAAgGGAACACCAAGcATCCCTGGAtATTAACAAtCCTCGaGACTTTATTGATTGcTTCCTGATCAAgATGGAGCAGGAAAAAcAcAATcAtCAGTCAGAaTaTACaTTTGAAAACcTGACAATCgCTGTaTCTGAtTTGTTTGGAGCTGGGACAGAGACAACGAGCACCACgCTGAGATATGGACTcCTCCTCCTGCTGAAGCACCCAGAGGTCACAGCTAAgGTCCAGGgAGAGATTGAcCGaGTcATTGGCAGACACCGgAGCCCCTGCATGCAGGACAGGAaCCACATGCCCTAcAcGGATGCgGTGATcCATGAGaTCCAGcGATACATTGAtCTgATCCCCACCAgtCTtCCCCATGCAGTGACCCaTGACaTTAAATTCAGAAACTACCTCATCCCCAAGGGCACAAAtATATTAAcATCACTGACTTCTGTGcTaCATGATGgcAAAGAATTCCCcAACCCtGAGGTaTTTGAtCCTGGCCACTTCCTGGAtgAGAGTGGCAACTTTAgaAAGAGTGACTATTTCATGGCTTTCTCtaCAGGAAAACGtgTTTGTtTGGGAaAGGGCCTGGCCCGCgTGGAGCTcTTTTTATTCCTGACcACaATTTTACAaAAATTTAACTTGAAaTCTgTGGTTGACCCAAAgGACcTCGACaTCACCCCAGTTGTCAATGGgTTaGtTTCTtTGCCACCgTTTTACcAGcTtTGTTTaATTCCTGTGTGA

>CYP2C113_mouse

ATGGATCcaGTccTGGTCCTGGtGCTCacTCTgTCCTGTctGCTTCTCCTcTCAtgaTGGAgACAaAaCTCtgGGAgAGGGAAGCTCCCtCCTGGCCCCACcCCTCTCCCaATTATTGGAAAcATCCTcCAGaTAGATGTgAAGAACTCAAAAGTCTAcGGCCCcGTaTTTACTCTGTActTTGGCATGAAGCCCtCcGTGGTGTTGCATGGgTATGAAGCcGTGAAGGAAgCtCTtgTTGATCTaGGaGAGGgaTTTTCTGGgAGAGGCAgTTttCCAGtagCTGAAAaAGCTAgcAAAGGcCTTGGggTtGTTTTCAGCAgTGGgAgGAccTGGAaaGAGATGaGaCGcTTCTCaCTCATGACTCTGCGaAATTTTGGGATGGGGAAGAGGAGCATTGAGGACCGAGTTCAAGAGGAgGCtCGCTGtCTTGTGGAtGAaTTGAGgAAAACCAACCCTGTGATCCCACcTTcATtCTcGGCTGTGCTCCTTGCAATGTcATCTGCTCCATtgTTTTCCAGAATCGTTTTGATTATAaAGATCAGgAaTTTCTtAcctTtATagAcAtcTTaaATGAAAATGTGCAATaATTTtCCcGcTaTCaTtGATTATcTaCCAGGaAGaCAcAgaAAATTaCTTAAAAATtTTGatTtTgcAAAacATTActTTTTGGcaAAAgtAAtacAgCAtaAAGAgTCaCTGGAtATTAACAAtCCTCGGGACTTTATTGATTGTTTCCTGATCAAAATGGAGCAGGtAAAAGAAAATAAACAaTCAGAGTTTtCacTTGAAAAtcTGgCAAgCACTaTcaaTGACcTcTTTGGtGCaGGGACAGAGACcACaAGCACaACaCTGAGATATGctCTcCTaCTCCTGCTGAAGtACCCAGAtGTCACAGCTAAAGTCCAGGAAGAGATTGAcCGTGTGgTTGGCAGgCACCGcAGCCCCTGCATGCAaGACAGGAGCCACATGCCCTATAcaGAcGCCaTGATtCATGAGGTCCAGAGATtCATcGACCTCcTCCCCACtAgCCTGCCtCATGCAGTGACCtGTGACaTTAAATTCAGgAAaTACCTCATCCCCAAGGGaACAAcagTAgTAAcgTCACTGACaTCaGTtcTGCATGAcagcAAgGAgTTCCCaAACCCAGAGtTGTTTGACCCTGGCCACTTtCTaGAtgctAaTGGaAACTTTAAGAAaAGTGACcAcTTCATGcCTTTCTCAGCAGGAAAACGtgTTTGTGctGGAGAGGGCCTGGCCCGCATGGAGCTGTTTcTATTCCTGACcACCATTTTACAGAAcTTTAAacTGAAaTCTCTGGTTcACCCAAAAGACATtGAtaTgAtCCCAtTTGTgAATGGATTaatagCTcTGCCACCCcaTTACcAGgTCTGcaTCATTCCTcgcTaA

>CYP2C92_horse

AGAGAATGGATCTGGTTGTGGTTCTGGGGCTCTGTCTCTCCTGTTTACTTCTCCTTTTACTCTGGAAAGAGAGCTCCAGGAAAGGGAAGCTCCCACCTGGCCCCACTCCTCTCCCTATTATTGGAAATATCCTACAGTTAGATGTTAAGAACATCAGCAAATCCTTAAGTAATCTTTCAAAAGTCTATGGCCCTGTGTTTACTCTGTATTTTGGCATGAAGCCCACTGTGGTGCTGCATGGATATGAAGCAGTGAAGGAAGCCCTGATTGATCTGGGGGAGGAGTTTTCCGGGAGAGGCCGTTTCCCAGTGACTGAAAGAGTTAATAAAGGACATGGAATCATTTCCAGCAATGGAAAGAGGTGGAAAGAGATCCGGCGCTTCTCCCTCATGACTCTGCGAAATTTCGGGATGGGGAAGAGGAGTATTGAGGACCGAGTTCAAGAGGAAGCCCGTTGCCTTGTGGAGGAGTTGAGAAAAACCAATGCTTCACCCTGTGACCCCACTTTTATCCTGGGCTGTGCTCCCTGCAATGTGATCTGCTCCATCATATTCCAGAATCGTTTTGATTATAAAGATCAGAATTTTCTAAACATAATGAAAGTCTTTGATGAAAATTTCAAGATTCTGAGCTCTCCATGGATGCAGATCTGCAATGCTTTTCCTGCTCTCCTTGAGTATTTCCCAGGGAGTACTGACAAATTATTTAAAAATGTTGCTTATGTAAGAAGTTATATTTTGGAGAAAGTAAAGGAACACCAGGCATCTCTGGACATTAACAATCCTCGGGACTTTATTGATTGTTTCCTGATTAAAATGGAGCAGGAAAAGCAAAATCAACAGTCGGAGTTTACTTTTGAAAACTTAAAAATCACTGTATCTGATTTGTTTGGAGCTGGGACAGAGACAACGAGCACCACCCTGAGATATGGACTTCTCCTCCTGCTGAAGCACCCAGAGGTCATAGCTAAAGTCCAGGAAGAGATTGATCGTGTGATTGGCAGACACCGGAGCCCGTGCATGCAGGACAAGAGCCACATGCCCTATACAGATGCTGTGGTGCATGAGATCCAGAGATACATTGACCTCCTCCCTACCAATGTGCCCCATGCAGTGACTCGTGACGTTAAATTCAGAAACTACTTTATCCCTAAGGGCACAACCATATTAATATCACTGACTTCTGTGCTGCACGATGACAGAGAATTCCCCAATCCAGAAGTATTTGACCCTGGTCACTTCCTGGATGAGAGTGGCAACTTTAAGAAGAGCGACTACTTCATGGCTTTCTCAGCAGGAAAACGGGTGTGTGCAGGAGAGGGCCTGGCCCGCATGGAGCTGTTTTTATTCCTGACCACCATTTTACAAAAATTTAACTTGAAATCTGTGGTTGATCCAAAGGACATTGACACCACACCTGTTGCAAATGGATTTGCTTtTGTGCCACCTTCTTATCAGCTTTACTTCATTCCTGTGTGAGACTGTtTGAACACCTGACTTTTCCTATGATGATGTtTGCAACTCCCTCTCATTTGGGACATGGTCCACCTCCTTTtTTtGTCACCTTCTTCCTGGGAATACCTTCGCTGACCTtTTCTtTCCTCATCATCCTATTtTtTCAAGATCCAGGAATATCCAATtTCCATTAAAGGAGAATTTCCTGAGTTTCACTGCATATACTCCATACTCTATtTGCTATTCTtTATACTtTGCAACGTTTGTTTTGGCCATCATATATGCTAATACCTATCTAATATTGAGTAGTGgTATGTCATCAATATGAAATAAAGCAAGATGATTAGTGTATACCAATGCAAAGCCATTTtTCCTtTGCATTTTtTAAATAAAATATATAATTTGATGAaaaaaaaaaaaaaaaaaaaaaaaaaaa

>CYP2C92_rhino

AATGGATCcaGcTGTGGTcCTGGtGCTCTGTCTCTCCTcTTTcCTTCTCCTTTccCTCTGGAAAcAGAGCTCCAGGAAAGGGAAGCTCCCgCCTGGCCCCACTCCTCTCCCTATTATTGGAAATATCCTgCAGTTAGATaTTAAGAACATCAGCAAATCCTTAAGTAATCTcTCAAAAGTCTATGGCCCTGTGTTcACTCTGTATTTgGGCtTGAAGCCCACTGTGGTGCTGCATGGATATGAAGCAGTGAAGGAAGCCCTGATTGATtTGGGGGAGGAGTTTTCtGGaAGAGGCaGTTTCCCAGcGgCTGAAAGAGcTAATAAAGGACtTGGAATCATTTtCAGCAATGGAAAGAGGTGGAAgGAaATCCGGCGCTTCTCCCTCATGACcCTGCGgAATTTCGGGATGGGGAAGAGGAGcATTGAGGACCGAGTTCAAGAGGAAGCCCGcTGCCTTGTGGAGGAGTTGAGAAAAACCAATGCTaCACCCTGTGAtCCCACTTTTATCCTGGGCTGTGCTCCCTGCAATGTGATCTGCTCCATtATtTTCCAcAAcCGTTTTGATTATAcAGATgAGAATTTTCTAAACATAgTGgAAaTaTTTGAcGAAAATgTCAAGATTaTcAGtTCcCCATGGATGCAGcTCTGCAATagTTTcCCTatggTCCTTGAtTATTTCCCAGGGAGTcacaAaAAATTATTTAAAAATGTTGCTTATGTAAaAAGTTATgTTTTaGAGAAAGcAAAGGAACACCAaGCATCTCTGGACATTAACAATCCTCGaGACTTTATTGATTGTTTCCTGATcAAAATGGAGCAGGAAAAGCAAAATCAgCAGTCaGAGTTTACcTTgGAAAAtTTgAcAgTCACTGcATtTGATTTGTTTGGAGCTGGGACAGAGACAACaAGCACtACCCTGAGATATGctCTcCTCCTCCTGCTGAAGCACCCAGAGGTCAAGCTAAAGTCCAGaAAGAGATTGAcCaTGTGATTGGCAGACACCGGAGCCCcTGCATGCAGGAtAAGAGCCAtATGCCCTAcAtgGATGCTGTGGTaCATGAGATCCAaAGATACATTGACCTCaTCCCTACCAAccTGCCCCATGCAGTGACTCGTGACaTTAAATTCAGAAACTACcTcATCCCcAAGGGCAtgACCATATTAAcATCACTGACTTCTGTGCTGCACGATGACAaAGAATTCCCCAAcCCAGAgGTgTTTGACCCTcGcCACTTCCTGGATGAGAGaGGCAACTTTAAGAAGAGCGAtTAtTTCATGGCTTTCTCAGCAGGAAAACGGaTGTGTGCAGGAGAaGGCCTGGCCCGCATGGAGCTGTTTTTATTCCTGACCgCCATTTTACAgAAATTTAcCcTGAAATCTGTGGTTGATCCAAAGGACATTGACACCACcCCgaTTaCcAATGGgTTTGCTTcTGTGCCgCCgTCTTAcCAGCTcTgCTTCATTCCcaTGTGAGAaccTcaGAACAtCTGACTTTTCCTgTGATGtTGTcTcCAACTCCCTCTCAcTTGGGgCtTcaTtCACaTaCTTccTTctTCACCcTCTTCCTGGtAATgCCTTCtCTGACCTcTTCTcTCCTCATCAcCtcATTcTcTCAAGtTCCAGGAAcATgCAAccTCCATgAAAGGgaAtTTcCtgagtTTcactgcatatTAtTCaActCTgTATcTGCTATTtTccATACTcTGtAACaTTcaTaTTGGCCATCATATATGCTAATACtTATaTAATATTGAGTAGTGtTATaTagTCAATATGAAATAAAGCAAGATGATTAGTGTATACCAATGCAgAtCCAcTTccCCTcTGCATTTTcTAAATAAAAagTATgATTTGATGAg

>CYP2C92_cow

cGtcAATGGATCTGGcTGTGGTcCTGGtGCTCTGTCTCTCCTGTcTgCTTCTCCTcTcACTCTGGAAAcAGAGCTCtgGGAAAGGGAAGCTCCCgCCaGGCCCCACTCCTCTCCCaATTcTTGGAAATATCtTtCAGTTAGATGTTAAGAAtATCAGCAAATCtTTAAcccAgCTcTCAAAAGTCTAcGGCCCTGTGTTcACTgTGTATTTTGGaATGAAGCCCACaGTaGTttTGCATGGATATGAAGCAGTGAAGGAAGCtCTGATTGATtTGGGaGAGGAGTTTTCtaGaAGAGGCaGTTTCCCAGTGAtTGAAAGAaaTgtTAAgGGACAcGGAgTCATTTtCAGCAATGGgAAGAtaTGGAAgGAGAcCCGGCGCTTCTCCCTCATGACcCTGCGggATTTCGGGATGGGGAAGAGGAGcATgGAGGACCGtGTTCAAcAGGAAGCgtGcTGCCTgGTGGAGGAGTTGAGAAAAACCgATGCCCTGTGAtCCtACTTTcATCCTGGGCTGTGCTCCCTGCAATGTGATCTGCTCCATtATtTTCCAGAATCaTTTTGATTATAAAGATCAGAtTTTTCTAgAtcTAATGgAAagacTgaATGAAAAcgcCAgaATTCTGgGCTCTCCATGGATtCAGgTCTGCAgTtCTTTcCCTGCTCTCaTTGAtTATgTCCCAGGGAaacaTaAaAAATTcTTTgAAAATtaTGCTTgTaTgAaAAGTTATgTTTTGGAGAAAacAAgGGAACACCAaGCATCcCTGGACATgAACAAcCCTCGGGACTTTATTGATTGTTTCCTGAccAAgATGGAaCAGGAAAAGCAcAATCAAgAactGGAGTaTACagTTGAAAACTTggcAcaCACTGTATtgGATTTGTTTGtgGCTGGGACAGAGACgACGAGCACCACaCTGAGATATGGgCTcCTCCTCCTGCTGAAaCACCCAGAGGTCAGCTAAgGTCCAGGAAGAGATTGAcCaTGTcATTGGCAGACACCGGAGCCCcTGCATGCAGGACAAGAGCCACATGCCCTAcACAGATGCTGTGGTcCAcGAGATCCAGAGATACATTGACCTggTCCCcACCAAccTGCCCCATGCgGTGACctGTGACaTTAAATTCAGAAACTACcTcATCCCaAAGGGCACAggtATATTAAcATCACTGACTTCTGTGCTatAtGATGACAaAGAATTtCCCAAcCCAGAgGTgTTTGACCCTGGcCACTTtCTGGATGAGAGTGGCAACTTTAgGAAGAGtGACcAtTTCATGGCTTTCTCAcCAGGgAAACGGaTtTGTGtgGGAGAGGGCCTGGCCCGCATGGAGCTGTTTTTATTCCTGACCACaATTTTACAAAAcTTTAcCTTGAAATCTGTGGTTGAcCCAAAGGACcTcGACACCACcCCaGTTGtcAATGGgTTactTTcTGTGCCACCTTtTTAcCAGCTcTgtTTCATTCCTGTGTGAGgACTCCCTCTCATcTGcGgCATTATcTGCTgTTCTTcATACTcTGtAACaTcTaTaTTGGCCtcCAgATATGCaAATAatTAcaTAATATTGAGTAG

>CYP2C92_dog

AAAGGGAAGCTgCCACCTGGCCCCACTCCTCTCCCaATcATTGGAAATATtCTACAGaTAaATacTAAGAAtgTCAGCAAATCCcTAAGccAgCTagCAgAgaatTATGGCCCTGTGTTcACTgTGTATTTTGGCATGAAGCCtACcGTGGTGtTGtAcGGgTATGAAGCgGTGAAGGAAGCtCTaATTGATCgGaGtGAaGAGTTTTCaGGcAGAGGCCaTTTCCCAGGAATtgTTTtCAGCAAcGGAgAaAaaTGGAAgcAaAcCCGGCGtTTtTCCCTgAcagtTtTGCGgAATaTgGGGATGGGaAAGAaGAcTgTTGAaGACaGAaTTCAAGAaGAgGCCttgTatCTaGTGGAaGcaTTaAaAAAAACCAAcGCaTCtCCCTGTGAtCCtACTTTccTtCTGGGCTGTGCTCCCTGCAATGTGATtTGCTCCATtATtTTCCAGAATCGTTTTGAgTATgAtGATaAagATTTTtTAAcCtTgtTagAgtatTTTcATGAAAAccTtctaATTtcaAGCaCctCcTGGATaCAGgAATGCTTTcCCTctTtTaaTacAtTAccTtCCAGGaAGTcaTcAtgtgTTATTTAAAAAcaTTGCTaAccaAtttAagTtTATTTcGGAaAAAaTAAAaGAACACgAaGaATCTCTaaACtTTAgtAAcCCTCGGGACTTTATTGAcTacTTCCTGATcAAAATaGAaAGGAAAAaCAcAAcaAACAGTCtGAaTTTACcaTgGAcAACTTgAtcATtACcaTATggGATgTGTTTaGtGCgGGaACAGAGACAACGAGCACCACCCTGAGATAcGGACTatTggTgCTatTaAAGCACCCAGAtGTCAcAGCTAAAGTCCAGGAAGAGATTcATCGTGTagTTGGCAGACAtCGGAGCCCcTGCATGCAaGAtAgGAGCtgCATGCCCTAcACAGATGCTGTGGTaCATGAGATCCAGAGATACATTGAtCTtgTCCCcAaCAATcTGCCCCATtCAGTGACTCagGACaTcAAgTTtAGAgAaTACcTTATtCCcAAGGGCACAACCATATTAAcATCtCTGACTTCTGTcCTGCAtGATGAgAaAGgATTCCCCAAcCCAGAtcagTTTGAtCCTGGcCACTTCCTGGATGAaAaTGGCAgCTTTAAGAAGAGtGACTACTTCATGGCcTTCTCAGCAGGcAAgCGaGTtTGTGttGGAGAaGGCCTGGCCCGCATGGAGCTGTTTTTgcTaCTGACCAatATTTTACAgcAtTTcAcCTTGAAAcCTcTGGTTGATCCAAAGGACATTGACACCACcCCaaTTGCcAATGGgTTgGgTgcTacaCCACCTTCcTATaAGCTcTgtTTtgTTCCaGTcTGA

>CYP2C92_pig

ATGGATggGGcTGTGGTcCTGGtGCTCTGTCTCTCCTGTcTgCTTCTCCTcTcACTCTGGAAAcAGAaCTCtgGGAAgGGGAgGCTCCCgCCTGGCCCgACTCCTCTCtCaATTcTTGGAAATATCCTACAGTTAGATGTTAAGgACATCAGCAAATCCTTAAGcAAgCTcTCAAAAGTCTATGGCCCTGTaTTcACcgTGTATTTTGGCtTGAAGCCtgCTGTtGTGtTGCATGGcTATGAAGCAGTaAAaGAAGCCCTaATTGATggGGGaGAGGAaTTTTCtGGaAGgGGCCaTTTCCCAaTGgCTGAgAGAGTTAATAAAGGgCATGGAATCATcTtCAGCAgTGGAAAGAGaTGGAAgGAGAcCCGGCGCTTCTCCCTCATGACcCTGCGgAATTTtGGGATGGGGAAGAGaAGcATTGAGGAaCGgGTTCAAGAGGAAGCCCGcTGCCTTGTGGAGGAGTTGAGAAAAACCAATGCTTCACCCTGTGAtCCCACTTTccTCCTGGGCTGTGCTCCCTGCAATGTGATCTGCTCCATCATtTTCCAcAATCGTTTTGATTATAcAGATCcaAATTTTCTtAcCtTgtTGgAAaaacTaaATGAAAAcTTCAgGATTCTGAGCTCcCCgTGGATcCAGgTCTGCAATaaTTTcCCTGCTCTgaTTGAcTATcTCCCAGGGAGTcaTaACAAAgTgcTTAAAAATtcTGCTTATGTAAaAAGcTATATTTTGGAGAAAGTAAAaGAACAtCAaGCATCcCTGGAtgTTAAtgAcCCTCGGGACTTcATTGATTGTTTCCTGATcAAgATGGAaCAgGAAAAaCAcAATCAcCAGgtGGcaTTTACTTTTGAAAACTTgAtAgcCACTaTAaCTGAcTTGTTTGGAGCTGGGACAGAGACAACaAGtACCACCCTGAGATATGGgCTcCTCCTgCTGCTGAAGCACCCAGAtGTCAAGCcAAAGTgCAGaAAGAGATTGActccGTGATTGagAGACACCGaAGtCCtTGCATGCAGGACAgaAGCCgCATGCCCTATACAGATGCcGTGGTGCATGAGATCCAGAGATACATTGACCTgaTCCCcAtCAgcGTGCCtCAcGCAGTGACcaGgGACaTTAAATTCAGAAACTACcTcATCCCcAAgGGCACAACtgTgTTAAcATCACTGACgTCTGTGCTGCgtGATGACcaAGAATTCCCCAAcCCAGAgGTgTTTGACCCTGGcCACTTCCTGGATGAGAGTGGCAACTTTAAGAAGAGtGACTgCTTCATGcCTTTCTCAaCAGGtAAACGGGgtTGTGtAGGAGAaGGCCTGaCCtGCATGGAGCTGTTTTTATTCCTGACCACaATTTTACAAAAATTTAcCTTGcAATCTGTGGTTGAcCCcAAGGACATTGACACCAgcaCTaTTtttggTGGtTTaGCTTccaTGCCACCTTCTTAcCAGCTcTgCTTCATTCCTtTGTCCCTCTCATcTGGGgCAT

>CYP2C92_elephant

ATGGATCTaGccGTGGTcCTcGtGCTCTGTCTCTCCaGTTTgCTTCTCtTTTcACTCTGGAAAcAGAGCTaCgGGAAAGGGAAGCTCCCgCCTGGCCCCACTCCTCTCCCaATTATTGGAAATAgCCTgCAGTTAaATtTTAAGAAtATCAGCAAATCtTTAtcccAgCTcTCAAAAacCTATGGCCCTGTGTTcACcCTGTATTTgGGCATGAAGCCCACaGTGGTGtTGCATGGgTATGAAGCAGTGAAGGAAGCCCTGATTGATCaGGGaGAGGAGTTTTCtGGaAGAGGCaGTTTCCCAGTGtCTGAAAaAGTTcATAAAGGACtTGGAgTCgTTTtCAGCAATGGAAAGAtGTGGAAgGAGAcCaGGCGCTTCTCCCTCATGACcCTGCGgAATTTtGGGATGGGGAAGAGGAGcATTGAGGACCGtGTTCAgGAGGAAGCCtGcTGCCTaGTGGAGGAGTTGAGAAAAACCAAGCcTCACCCTaTGAtCCtACTTTcATCCTGGGCTGTGCTCCCTGCAATGTGATCTGCTCCATtATtTTCCAGAATCGTTTTGATTATAcAGATCAGgATTTTCTgAACtTgATaggAaaaTTgaATGAAAAcaTacAGATTCTGAGCTCcCCATGGgTcCAGgTCTGCAATagTTTTCCcttatTCaTTGAtTATTTgCCAGGGAGTcaTaACAAAcTcTTTAAtAATaTTGCTTATtcAAaAAGTTAcATTTTGGAGAAAaTgAAaGAACACCAGGaAaCctTGGACATTAACAATCCTCGGGACTTcATTGATTGTTTCCTGATcAAAATGGAaCAGGAAAAGgAcAATCAAgAGTCtGtaTTTACTaTTGAAAACTTgAtAATCACTaTAagTGATgTGTTTtctGCTGGGACAGAGACAACaAGtACCACaCTGAGgTATGGACTcCTgCTtCTGCTaAAGCACCCAGAGGTCAcAGCTAAAGTCCgGGAAGAGATTGAcCGTGTGgTTGGCcGACACCGGAGCCCcTGCATGCAGGACAgGAGCagCATGCCCTAcACgGATGCTGTGGTGCAcGAGATCCAGAGATACATTGACCTtaTCCCcACCAAccTGCCCCATGCgGcGACcCaaGACaTTAAATTCAGgAACTACcTcATCCCcAAGGGtAtgACCATATTAAcATCACTGACTTCTGTGCTtCACGATaACAaAGAATTCCCCAAcCCAGAgacATTTGACCCTGcTCACTTtCTGGATGAGAGTGGCAACTTTAAGAAGAGCGACTACTTCATGGCTTTtTCAGCAGGAAAACGtaTtTGTctgGGAGAGGGCCTGGCCCGCATGGAGCTGTTTTTATTCtTcACtgCCATTTTACAgAAcTTTAcCTTGAAgcCTcTGGTTGAcCCAAAGGACATcGAtACCAacCCaGTTGaAAAaGGgTTTGCcTcTGTGCCACCcaagTAcCAGCTcTgCTTtATTCCTGTGTGA

>CYP2C92_human

AATGGATCcaGcTGTGGcTCTGGtGCTCTGTCTCTCCTGTTTgtTTCTCCTTTcACTCTGGAggcAGAGCTCtgGaAgAGGGAgGCTCCCgtCTGGCCCCACTCCTCTCCCgATTATTGGAAATATCCTgCAGTTAGATGTTAAGgACATgAGCAAATCCTTAAccAATgTaTCAAAAGTCTATGGCCCTGTGTTcACTgTGTATTTTGGCcTGAAGCCCAtTGTGGTGtTGCATGGATATGAAGCAGTGAAGGAgGCCCTGATTGATCatGGaGAGGAGTTTTCtGGaAGAGGaaGTTTtCCAGTGgCTGAAAaAGTTAAcAAAGGACtTGGAATCcTTTtCAGCAATGGAAAGAGaTGGAAgGAGATCCGGCGtTTCTgCCTCATGACTCTGCGgAATTTtGGGATGGGGAAGAGGAGcATcGAGGACCGtGTTCAAGAGGAAGCCCGcTGCCTTGTGGAGGAGTTGAGAAAAACCAATGCcTCACCCTGTGAtCCCACTTTcATCCTGGGCTGTGCTCCCTGCAATGTGATCTGCTCtgTtATtTTCCAtgATCGaTTTGATTATAAAGATCAGAggTTTCTtAACtTgATGgAAaaaTTcaATGAAAAccTCAgGATTCTGAGCTCTCCATGGATcCAGgTCTGCAATaaTTTcCCTGCTCTCaTcGAtTATcTCCCAGGaAGTcaTaAtAAAaTAgcTgAAAATtTTGCTTAcaTtAaAAGTTATgTaTTGGAGAgAaTAAAaGAACAtCAaGaATCcCTGGACATgAACAgTgCTCGGGACTTTATTGATTGTTTCCTGATcAAAATGGAaCAGGAAAAGCAcAATCAACAGTCtGAaTTTACTgTTGAAAgCTTgAtAgcCACTGTAaCTGATaTGTTTGGgGCTGGaACAGAGACAACGAGCACCACtCTGAGATATGGACTcCTgCTCCTGCTGAAGtACCCAGAGGTCAcAGCTAAAGTCCAGGAAGAGATTGAatGTGTagTTGGCAGAaACCGGAGCCCcTGtATGCAGGACAgGAGtCACATGCCCTAcACAGATGCTGTGGTGCAcGAGATCCAGAGATACATTGACCTCCTCCCcACCAAccTGCCCCATGCAGTGACctGTGAtGTTAAATTCAaAAACTACcTcATCCCcAAGGGCACgACCATAaTAAcATCcCTGACTTCTGTGCTGCACaATGACAaAGAATTCCCCAAcCCAGAgaTgTTTGACCCTGGcCACTTtCTGGATaAGAGTGGCAACTTTAAGAAaAGtGACTACTTCATGcCTTTCTCAGCAGGAAAACGGaTGTGTatgGGAGAGGGCCTGGCCCGCATGGAGCTGTTTTTATTCCTGACCACCATTTTgCAgAAcTTTAACcTGAAATCTcaGGTTGAcCCAAAGGAtATTGACAtCACcCCcaTTGCcAATGcATTTGgTcgTGTGCCACCcTtgTAcCAGCTcTgCTTCATTCCTGTcTGAaATcTGCTATTCTccATACTcTGtAtCacTTGTaTTGaCCAcCAcATATGCTAATACCTATCTAcTgcTGAGTtGTc

>CYP2C92_sheep

AATGGATCTGGcTGTGGTcCTGGtGCTCTGTCTCTCCTGTcTgCTTCTCaTcTcACTCTGGAAAcAGAGCTCtgGGAAAaGGAAGCTCCCgCCgGGCCCCACTCCTCTCCCaATTcTTGGAAATATCCTACAGTTAGATGTTAAGAACATCAGCAAATCtTTAAccAATCTcTCAAAAGTCTATGGCCCTGTGTTcACTgTGTATTTTGGCATGAAGCCCAtcGTGGTctTGCATGGATATGAAGCAGTGAAaGAAGCCCTGATTGATCTGGGaGAaGAGTTTTCtGGaAGAGGaacaTTtCCAtTGgCTGAAAGAGcTAATGGAATCcTTTtCAGCAATGGgAAGAcaTGGAAgGAGATgCGGCGCTTCTCCCTCATGACcCTGaGAAATTTCGGGATGGGGAAGAGGAGcATTGAGGACCGAGTTCAAGAaGAAGCCCacTGCCTgGTGGAGGAGTTGAGAAAAACCAATGCTTCACCCTGTGACCCCACTTTcATCCTGGGCTGTGCTCCCTGCAATGTGATCTGCTCCATtATtTTCCAGAAcCGTTTTGATTATAAAGATCcGAtTTTTCTAgAtATAATGgAAaaaTTgaAcGAAAtccTCAgGATTCTGAGCTCTCCATGGgTtCAGgTGCAATaaTTTcCCTGCaCTCaTTGAtTATTTaCCAGGaAGTcaTaACAAAgTAaTTAAAAATGTTGCTaATtTAAaAAGTTATgTgTTGGAGAAAGcAAtGGAACACaAaGCATCcCTGGAtATTAACAAcCCTCGGGACTaTATTGATTGTTTCCTGATcAAAATGGAGCAGGAAAAaCAAAATCAACAaTtGGAGTTTACTTTgGAAAACTTgAcAAcCACTGTATtTGATTTGTTTGGAGCTGGGACAGAGACgACaAGCACCACtCTGAGATATGGgCTcCTCCTgCTGtTGAAGCACCCAGAGGTCAcAGCTAAgGTCCAGGAAGAGATTGAcCaTGTGATTGGaAGACACCGGAGCCCcTGCATGCAGGACAgaAGCCACATGCCCTAcACgGATGCTGTGGTcCATGAGATCCAGAGATACATTGACCTggTCCCTtCCAgTcTGCCCCATatgGTGACcCaTGACaTTgAATTCAGAAACTACaTcATCCCcAAGGGCACAggtgTATTAgTATCACTGAgTTCcGTGCTGtAtGATGACAaAGtATTtCCtAAcCCAGAAaTgTTTGACCCTGGcCACTTCCTGGATGAGAGTGGtAACTTTAAaAAGAGtGACTACTTCATGcCTTTCTCAGCAGGAAAACGGaTtTGTGCAGGAGAGGGCCTGGCCCGCATGGAGgTGTTTTTATTCCTGACatCgATTTTACAAAAATTTActTTGAAATCTGTGGTTGAcCCAAAGGACATcGACACCACcCCaacTGCcAATGGgTTcGCTTcTGTGCCACCTcCTTAcaAGCTcTgCTTCATTCCTcTGTaAGgCTGACTTTGATGTTTtCAACTCCCTCTCATTTTCACTGCATATcTGCTgTTCTTcATACTcTGtAACaTcTGTaTTGGC

>CYP2C92_mouse

ATGGATCcaGTccTGGTcCTGGtGCTCacTCTgTCCTGTcTgCTTCTCCTcTcAtgaTGGAgAcAaAaCTCtgGGAgAGGGAAGCTCCCtCCTGGCCCCACcCCTCTCCCaATTATTGGAAAcATCCTcCAGaTAGATGTgAAGAACTCAAAAGTCTAcGGCCCcGTaTTTACTCTGTAcTTTGGCATGAAGCCCtCcGTGGTGtTGCATGGgTATGAAGCcGTGAAGGAAGCtCTtgTTGATCTaGGaGAGGgaTTTTCtGGGAGAGGCaGTTTtCCAGTagCTGAAAaAGcTAgcAAAGGcCtTGGAATCATTTttAGCAATGGgAtGAaaTGGAAAGAGATCCGGCGtTTCTCCgTCATGACTGaGgAATTTtGGGATGGGcAAGAGGAGTgTTGAGGAtCGtGTTCAAGAGGAgGCtCGgTGtCTTGTGGAGGAacTGAGgCCCTGTGAtCCCACcTTcATCCTGGGCTGTGCTCCCTGCAATGTGATCTGCTCCATtATtTTCCAGAAaCGcTTTGATTATAAAGATCAGAcTTTTCTtAACtTgATGgAtaaaTTTaATGAAAAcTTCAgGATctTGAGCaCaCCATGGATaCAGgTCTaCAATGCTcTgCCTaCcCTCaTcaAtTATcTCCCAGGGAGTcaTaACAAAgTAaTTAAAAATtTTaCTgAaaTAAaAAGTTAcATTTTGGgGcgAGTgAAaGAACACCAGGaAaCaCTGGACATggACAATCCTCGGGACTTcATTGAcTGcTTCCTGATcAAAATGGAaCAGGAAAAaCAcAATCccCAtTCGGAGTTTACTaTTGAAAgCTTgAtggcCACTGTAaCcGAcaTaTTTGtAGCTGGaACAGAaACcACaAaCAttACtCTaAGATATGGACTctTgCTCCTaCTGAAaCACaCAGAGGTCATAGCTAAAGTCCAGGcAGAGATcGATCacGTGATTGGCAGACACaGGAGCCCcTGCATGCAGGACAgaAcCCgCATGCCCTAcACAGATGCcaTGGTaCATGAGATtCAGAGATACATTGACCTCaTCCCcAaCAATGTGCCCCATGCAGctACctGTaAtGTTAgATTCAGAAgtTACTTcATCCCcAAGGGCACAgagtTAgTAAcATCACTGACTTCTGTGCTGCAtGATGACAaAGAATTtCCCAAcCCAGAAGTATTTGACCCTGGcCAtTTtCTGGATGAGAaTGGgAACTTTAAGAAGAGtGACTACTTtATGcCTTTCTCAaCAGGAAAgCGaaTGTGcGtgGGAGAGGcCCTGGCtCGCAcGGAGCTGTTTTTgaTtCTGACCACCATTTTACAgAAtTTTAACcTGAAATCTtTGGTTGATaCAAAGGACATTGACACtACtCCaGTgGCcAATactTTTGgccgTGTaCCACCTTCaTAcCAGCTgTACTTCATTCCTcgtTaA

>CYP2D50_horse

ATGGGGCTGCTGACCTGGGATAAACTGGGGCCTGTGGCTGTGGCCGTGGCCATCTTCCTGCTCCTGGTGGACCTGATGCACCGGCGCCAACGCTGGGCCCCACGCTACCCCCCAGGCCCCATGCCCCTGCCTGGGCTGGGCAACCTGCTGCAGGTGGACTTCCAGGACACGGTTTCCAGCTTTACTCGGCTGCGGCGCCGCTTCGGGGACGTGTTCAGCCTGCAGCTGGCCTGGACGCCCGTGGTCGTGCTCAACGGGCTGGCGGCCATTCGCGAGGCGCTGGTGCACCGCGGCGAGGACACCTCCGACCGCCCACGTGTGCCGGTGATGGAGCACCTGGGTTTCGGGCCGCACGCGGAAGGGGTGGTCTTTGCCCGCTATGGGCACACCTGGCGGGAGCAGCGGCGCTTCTCCGTGTCCACCCTGCGCAACTTCGGCCTGGGCAAGAAGTCCCTGGAGCAGTGGGTGACCCAGGAGGCCTCGTACCTCTGTGCCGTCTTCGCCGACCAGGGCGGAAGGCCCTTTAGCCCCGACGCCCTCCTGAATAAAGCGGTGAGCAACGTGATCGCCTCCCTGACCTTtGGGgGCCGCTTTGACTACAACGACCCGCACTTCCTTGAGATATTGGACCTAACAGAGGACATACTGAAAGAGCAGTCAGGCTTCCTGCCCCAGGTGCTGAACGCGATCCCCATGCTCCTGCACATCCCGGGGCTGGTCGCCAAGGTCTTTCCTGGGCAGAGGGCCTTCATGGCCCAGTTGGATGAGCTGGTTGCTGAGCGCAGGATGACCCGGGACCCGGCCCAGCCTCCTCGAGACCTGACTGATGCCTTCCTGGACGAGGTGCAGAAGGCCAAGGGGAACCCGGAGAGCAGCTTCAATGATGACAACCTGCGCCTGGTGGTGTCTGACCTGTTCGCCGCTGGGATGGTGACCACCTCAACAGCGCTGGCCTGGGCCCTCCTGCTCATGATCCTGCACCgGGATGTGCAGCGCCGTGTCCAACAGGAGATCGATGAGGTGATAGGGCAGGCGCGGCGACCAGAGATGGGGGACCAGGCCCGtATGCCCTTCACCATGGCgGTGGTCCACGAGGTTCAGCGCTTTGGGGACATCGCCCCAGTGGGCGCGCCCCACATGACGTCCCGTGACATTGAAGTGCAGGGCTTCCTCATTCCCAAGGGgACCACGCTCATCaCCAACCTGTCATCGGTGCTGAAGGACGAGACCGTCTGGAAGAAGCCCTTCCGCTTCCACCCCGAGCACTTCCTGGACGCCCAGGGCCGCTTCGTCAAGCAGGAGGCCTTCATGCCCTTCTCAGCAGGCCGCCGCTCGTGCCTCGGGGAGCCCCTGGCCCGCATGGAGCTCTTCCTCTTCTTCACCTGCCTCCTGCAGCGCTTCAGCTTCTCGGTGCCCGCTGGGCAGCCCCGCCCCAGCGACCACGGTGTCTTTGGCACCCTGGTCTCCCCGGCCCCCTACCAGCTCTGTGCTGAGCCCCGCTAG

>CYP2D50_rhino

ATGGGGCTGCTGACCgGGGAgAcACTGGGGCCccTGGCTGTGGCCGTGGCCATCTTCCTGCTCCTGGTGGACCTGATGCACCGGCGCCAgCGCTGGGCCCCACGCTACCCaCCAGGCCCCAcGCCCCTGCCTGGGCTGGGCAACCTGCTGCAGGTGGACTTCCAGGACACacTTTgCAGCTTTACTCGGCTGCGGCGCCGCTTCGGGGACGTGTTCAGCCTGCAGCTGaCCTGGACGCCCaTGGTCGTGCTCcACGGGCTGGCGGCCATgCGCGAGGCGCTGGTGgACCaCGGCGAGGACACCgCgGACCGCCCACcTaTGCCcGTGtTcGAGCACCTGGGcTTCtGaCCaCACGCGcAAGGGGTGGTCTTTGCCCGCTATGGGCgCgCCTGGCGGGAGCAGCGGCGCTTCTgCGTGTCCACCCTGCGCAACTTCGGCCTGGGCAAGAAGTCtCTGGAGCAGTGGGTGACCgAGGAGGCCTCGTgCCTCTGcGCCGcCTTCGCaGACCAGGcCGGAcGcCCCTTTAGCCCCaACGCCCTCCTGAATAAAGCGGTGgGCAACGTGATCGCCTCCCTcACCTTcGGGtGCCGCTTcGAgTACAAgGACCtGCgCTTCCTcaAGcTATTGGACCTcACAGAGtACtTACTGAAAGAGgAGaCtGGtTTCCTGCCCCAGGTGtTGAACGCGATCCCCATGCTCCTGtgCATCCCGGGGCTGGTCGCCAAGGTCTTTCCaGGtCAtAaGGCCTTCATGGaCCtGaTGGATGAGCTGaTTGCTGAGCaCAGGATGACCCGGGACCCGGCgCAGCCTCCcCGAGACCTGACTGATGCCTTCCTGGACGAGGTGgAGAAGGCCAAGGGGAACCCcGAGAGCAGCTTCAgTGATGAgAACCTGCGCCTGGTGGTGTCTGACCTGTTCaCCGCTGGGATGGTGACCACCTCgACcaCGCTGGCCTGGGCCCTCCTGCTCATGATCCTGCACCcGGATGTGCAGCGCCGTGTCCAACAGGAGATCGATGAGGTGATtGGcCAGGCaCGGCGACCAGAGATGaGGGACCAGGCCCacATGCCCTTCACCATGGCcGTGGTCCAtGAGGTgCAGCGCTTTGGGGACATtGtCCCAcTGGGCttGCCCCACATGACaTCtCGTGACATTGAAGTGCAGGGCTTCCTCATcCCCAAGGGGACaAtGCTCATCACCAACCTGTCATCGGTGCTGAAGGACGAGACCGTCTGGAAaAAGCCCTTCCGCTTCCACCCtGAGCACTTCCTGGACGCCCAGGGCCGCTTCGTCAAGCAGGAGGCCTTCATGCCCTTCTCAGCAGGCCGCCGCTCGTGCCTCGGGGAGCCCCTGGCCCGCATGGAGCTCTTCCTCTTCTTCACCTGCCTCCTGCAGCGCTTCAGCTTCTCGGTGCCCGCTGGGCAGCCCCGCCCCAGtGACtAtGGTGTCTTTGGCtCtCTGGTgaCCCCGtCaCCCTACCAGCTCTGTGCTGtGCCCCGCTAG

>CYP2D50_cow

ATGGGGCTGCTGtCtgGGGAcAcgCTGGGGCCccTGGCcGTGGCCcTGctCATCTTCtTGCTCtTGcTGGACCTGATGCACCGGCGCtcACGtTGGGCCCCACGCTACCCaCCAGGCCCCAcGCCgCTGCCgGtGCTGGGCAAtCTGCTGCAGGTGGACTTCgAGGACcCacgTcCCAGCTTTACTCGGCTGCGGCGCCGCTTCGGGaACGTGTTCAGCCTGCAGCaGGtCTGGACGCCgGTaGTCGTGCTCAACGGGCTGGCGGCtgTgCGCGAGGCaCTGGTGtACCGCaGCcAGGACACtgCCGACCGtCCACcTccGgCcGTctacGAGCACCTGGGTTaCGGGCCGCgCGCcGAAGGaGTGaTCcTgGCgCGaTATGGGgACgCCTGGCGcGAGCAGCGGCGCTTCTCCcTGaCCACCCTGCGCAACTTCGGCCTGGGgAAGAAGTCaCTGGAGCAGTGGGTGACCgAGGAGGCCTCGTgCCTCTGTGCCGcCTTCGCCGACCAGGcCGGAcGtCCCTTTAGCCCCatgGaCCTCCTGAATAAAGCaGTGAGCAACGTGATCGCCTCCCTGACCTTcGGGtGCCGCTTcGAgTACAACGAtCCtCgCaTCaTcaAGcTgTTGGACtTgACgGAGGAtgggCTGAAgGAaCAGGTGgTGgAaGCtgTgCCagTGCTCCTGagCATCCCaGGGCTGGctGCCAgGGTCTTcCCgGcGCAGAaGGCCTTCATGGCCCtGaTtGATGAGCTGaTcGCTGAGCagAaGATGACCCGGGACCCGaCCCAGCCaCCcCGAcACCTGACTGAcGCCTTCCTGGAtGAGGTGacccAGGCCAAGGGGAACCCcGAGAGCAGCTTCAATGATGAgAACCTGCGCCTGGTGGTGgCcGACCTGTTCtCCGCcGGGATGGTcACCACCTCgACcaCaCTGGCCTGGGCCCTCCTcCTCATGATCCTGCACCctGAcGTGCAGCGtCGgGTCCAgCAGGAaATCGATGAGGTGATAGGGCAGGtGaGGCGACCAGAGATGGGGGAtCAGGCCCtcATGCCCTTCACCgTGGCcGTGGTCCAtGtGGaggAaCGCTTTGcGGACATCGtCCCccTGGGattGCCCCAaAaGgCaTCCCGTGACATcGAgGTGCAGGGCTTCCaCATcCCCAAGGGGACgACaCTCATCACCAACCTGTCgTCaGTGCTGAAGGACGAGACCGTCTGGgAGAAGCCCTTCCGCTTCCACCCgGAGCACTTCCTGGAtGCCCAGGGCCGCTTCGTCAAGCAGGAGGCCTTCATaCCCTTCTCtGCAGGCCGCCGCgCGTGCCTCGGGGAGCCCCTGGCCCGCATGGAGCTCTTCCTCTTCTTCACCaGCCTCtTGCAGCatTTCAGCTTCTCGGTGCCtGCcGGGCAGCCCCGCCCCAGCGAgCACGGTGTCTTTGcCttCCTGGTgaCCCCaGCCCCCTACCAGCTCTGTGCgGtGCCCCGCTAG

>CYP2D50_dog

ATGGGGCTGCTGACCgGGGAcAcgCTGGGGCCccTGGCcGTaGCCGTGGCCATCTTCCTGCTCCTGGTaGACCTGATGCACCGGCGCaggCGCTGGGCCaCACGCTACCCgCCAGGCCCCAcGCCagTGCCcatGgTGGGCAACCTGCTGCAGaTGGACTTCCAGGAGCTGCaagGCCGCTTCGGaaACGTGTTCAGCCTGgAGCTGGCCTGGACGCCgGTGGTCGTGCTCAACGGcCTGGaGGCggTgCGCGAGGCcCTGGTGCACCGCaGCGAGGACACtgCCGACCGCCCACcTaTGCCGaTctatGAcCACCTGGGTTTgGGGCCaGGtTGtTCcTgGCgCGCTAcGGGCgtgCgTGGCGcGAGCAGCGGCGCTTCTCgcTGTCCACCCTGCGCAACTTCGGCCTGGGCAAGAAGTCCCTGGAGCAGTGGGTGACCgAGGAGGCCTCGTgCCgCAGGccCTGAACtCcATCCCCgTGCTCCTGCACATCCCGGGaCTGGcCagCAAGGTCTTctCcGcGCAGAaGGCCTTCATaaCCCtGacaaATGAaaTGaTccagGAGCaCAGGAaGACCCGGGACCCcaCCCAGCCaCCcCGAcACCTGAtcGAcGCCTTCgTGGAGGCCAAaGGGAACCCcaAGAcCAGCTTCAATGAgGAgAACCTGtGCaTGGTGaccTCTGACCTGTTCGtCGCTGGGATGGTGtCCACCTCAAtcaCGCTGaCCTGGGCCCTCCTGCTCATGATCCTGCACCcGGAcGTGCAGCgtCGTGTCCAgCAGGAGATaGATGAGGTGATAGGGCgGGaGCaGCtACCAGAGATGGGaGACCAGaCCCGTATGCCCTTCACCgTGGCcGTGaTCCAtGAGGTgCAGCGtTTTGGGGACATCGtCCCAcTcGGtGtGCCCCACATGACGTCCCGaGACAcTGAAGTGCAGGGCTTCCTCATcCCCAAGGt

>CYP2D50_elephant

ATGGGGCTaCTGAttgGGGAaggcCTGGtGCtTcTGGCaGTGGCCGTGGCCgcCTTCCTGCTCaTGGTGGACtTGATGCACCGGCGtgcACGCTGGGCtgCAaaCTACCCtCCAGGCCCtATGCCaaTGCCTGGGCTGGGCAACCTGCTGCAGcTGGACTTCgAGGACAtGccaTaCAGCTtTACTCGGCTGCGGCGCCGCTTtGGGGACGTGTTCAGCaTcCAGCTGGCCTGGACaCCCGTGGTtGTGCTCAACGGGCTGaaaGCCgTgCGtGAGGCcCTGGTGCtCCGCGGCGAGGACACggCCGACCGCCCACcTGTaCCcGTctatGAGCACCTGGGcTTCGGGCCGCggGCGcAAGGcGCgCGCTATGGGCACgCgTGGCGcGAGCAGCGaCGCTTCTCCtTGTCCACaCTGCGCAACTTCGGCCTGGGCAAGAAGTCgCTGGAGCAGTGGGTGACCgAGGAGGCCgCcTgtCTCTacGCCGgCTTCGCtGcCCAGGctGGAcGcCCCTTcAGgCCCacCGCgCTgCTGAAcAAAGCGGTGAGCAACGTGATCGCCTCCCTcgtCcaTGGGcGCCGCTTcGACTAtgAtGACCCGacCTTgCTcaAacTccTGGAgCTggCgGAcGcaggACTGAAgcAGgAtTCAGcCTTCCAGaTGCTGAACGCGgTCCCggTGCTCCTGaACgTCCCGGGGCTGGcCagCAAGGTCTTctCgGcaCAGAaGGCCTTCgTGGtCCtaaTGGATaAaCTGGTcGgcGAGCaCcGGgaGACgCGGGACCCGGCCCAGCCgCCcCGcGACCTGACcGATGCCTTCCTGGAtGAGGTGgAGAAGGCCAAGGaGAAaCCGGAGAGCAGCTTCAcTaATGAgAACCTGCGCtTGGTGGTGgCcGACCTGTTCaaCGCTGGGATGGTGACCACCTCgACcaCaCTGGaCTGGGCaCTtCTGCTtATGcTCCTGCACCcGGAcGTGCAGCGCCGTGTCCAgCAGGAGATCGAcGAGGTGATAGGGCAGGttCGGCaACCtcAGATGGaGGACCAGGCCCGcATGCCCTTCACCAcaGCaGTGGTCCAtGAGGTgCAaCGCTTTGGGGACATaGtgCCAcTGGGtGtGCCtCACATGACGTCCCGTGACAcTGAAGTGCAGGGaTTtCTCATcCCCAAGGGcACgACGCTCATCtgCAACCTGTCATCGGTGCTGAAGGAtGAGgCtGTCTGGAAGcAGCCaTTCCGtTTtCACCCCGAGCACTTCCTGGAtGCCCAGGGCCGCTTCGTgAAGCAGGAGGCtTTCATGCCtTTCTCAGCAGGCCGtCGCTtaTGCCTtGGGGAGCCCCTGGCaCGCATGGAGCTCTTtCTCTTCTTCACCTGCCTCCTGCAGCGCTTCAGCTTCTtGGTGCCtGCcGGaCAGCCCCGgCCCAGtGACCAtGGTGTCTaTGcCttCCTGtTCaCCCCattCCCCTACCAGCTCTGTGCTGtGCCCCGCTAa

>CYP2D50_human

ACTGGtGCCccTGGCcGTGataGTGGCCATCTTCCTGCTCCTGGTGGACCTGATGCACCGGCGCCAACGCTGGGCtgCACGCTACCCaCCAGGCCCCcTGCCaCTGCCcGGGCTGGGCAACCTGCTGCAtGTGGACTTCCAGaACACGGTTTCCctCccTctgCaGtTGCGGCGCCGCTTCGGGGACGTGTTCAGCCTGCAGCTGGCCTGGACGCCgGTGGTCGTGCTCAAtGGGCTGGCGGCCgTgCGCGAGGCGCTGGTGacCCaCGGCGAGGACACCgCCGACCGCCCgCcTGTG

CCcaTcAcccAGatCCTGGGTTTCGGGCCGCgGGGGTGtTCcTgGCgCGCTATGGGCcCgCgTGGCGcGAGCAGaGGCGCTTCTCCGTGTCCACCtTGCGCAACTTgGGCCTGGGCAAGAAGTCgCTGGAGCAGTGGGTGACCgAGGAGGCCgCcTgCCTtTGTGCCGcCTTCGCCaACCActcCGGAcGcCCCTTTcGCCCCaACGgtCTCtTGgAcAAAGCcGTGAGCAACGTGATCGCCTCCCTcACCTcGGGcGCCGCTTcGAgTACgACGACCCtCgCTTCCTcagGcTgcTGGACCTAgCtcAGGAgggACTGAAgGAGgAGTCgGGCTTtCTGCgCgAGGTGCTGAAtGCtgTCCCCgTcCTCCTGCAtATCCCaGcGCTGGctGgCAAGGTCcTaCgcttcCAaAaGGCtTTCcTGaCCCAGcTGGATGAGCTGcTaaCTGAGCaCAGGATGACCtGGGACCCaGCCCAGCCcCCcCGAGACCTGACTGAgGCCTTCCTGGcaGAGGTGCAGAAGGCCAAGGGGAACCCtGAGAGCAGCTTCAATGATGAgAACCTGCGCaTaGTGGTGgCTGACCTGTTCtCtGCcGGGATGGTGACCACCTCgACcaCGCTGGCCTGGGgCCTCCTGCTCATGATCCTaCAtCcGGATGTGCAGCGCCGTGTCCAACAGGAGATCGAcGAcGTGATAGGGCAGGtGCGGCGACCAGAGATGGGtGACCAGGCtCacATGCCCTaCACCActGCcGTGaTtCAtGAGGTgCAGCGCTTTGGGGACATCGtCCCccTGGGtGtGaCCCAtATGACaTCCCGTGACATcGAAGTaCAGGGCTTCCgCATcCCCcAGGGaACgACaCTCATCACCAACCTGTCATCGGTGCTGAAGGAtGAGgCCGTCTGGgAGAAGCCCTTCCGCTTCCACCCCGAaCACTTCCTGGAtGCCCAGGGCCaCTTtGTgAAGCcGGAGGCCTTCcTGCCtTTCTCAGCAGGCCGCCGtgCaTGCCTCGGGGAGCCCCTGGCCCGCATGGAGCTCTTCCTCTTCTTCACCTcCCTgCTGCAGCaCTTCAGCTTCTCGGTGCCCaCTGGaCAGCCCCGgCCCAGCcACCAtGGTGTCTTTGctttCCTGGTgagCCCatCCCCCTAtgAGCTtTGTGCTGtGCCCCGCTAG

>CYP2D50_sheep

ATGGGGCTGCTGtCtgGGGAcAtgCTGGGGCCccTGGCcGTGGCCGTGctCATCTTCtTGCTCtTGcTGGACCTGATGCACCGGCGCtcACGCTGGGCCCCACGCTACCCaCCAGGCCCCAcGCCgCTGCCgGtGCTGGGCAACCTGCTGCAGGTGGACTTtgAGGAgcCatgTctCAGCTTGGaGTGaTCcTgGCgCGaTATGGGaAggCCTGGCGaGAGCAGCGGCGtTTCTCCcTGTCCACCCTGCGCAACTTCGGCCTGGGgAAGAAGTCaCTGGAGCAGTGGGTGACtgAGGAGGCCTCGTgCCTCTGTGCCGcCTTCGCaGACCAGGcCGGAcatCCCTTTAGCCCCatgGatCTCCTGAATAAAGCaGTGAGCAACGTaATCGCCTCCCTGACCTTcGGGtGtCGaTTcGAgTACAAgGAtCCtCgCaTCgTcaAGcTgTTGGAtgTgAtgGAGGAtgggCTGAAgGAaCCTGCgCATCCCaGGGCTGGcCGCCcAGGgCTTcCCgGGGCtGAaGGCCTTCATGGCCCtACCgGGGACCCtcCCCAGCCaCCcCGAcACCTGACcGAcGCCTTCCTGGAtGAGGTGacccAGGCCAAGGGGAACCCcGAGAGCAGCTTCAATGATGAgAACCTGCGCaTGGTGGTGgtcGACCTGTTCtCCGCTGGGATGGTcACCACCTCgACcaCaCTGGCCTGGGCCCTCCTcCTCATGATCCTGCACCcaGAcGTGCAGCgtCGgGTCCAgCAGGAaATCGATGAGGTGATAGGGaAGGtGaGGCGACCAGAGATGGGGGAtCAGGCCttcATGCCCTTCACCgTGGCcGTGGTCCAtGAGGTgCAaCGCTTTGcGGACATCatCCCccTGGGactGCCCCACATGACaTCCCGcGACATcGAgGTGCAGGGCTTCCaCATcCCaAAGGGGACgACaCTCATCACCAACCTGTCgTCaGTGCTGAAGGAtGAGACCGTCTGGgAGAAGCCCTTCCGCTTCCACCCgGAGCACTTCCTGGAtGCCCAGGGCCGCTTCGTCAAGCAGGAGGCCTTCAaaCCCTTCTCcGCAGGCCGCCGCgCaTGCCTCGGGGAGCCCCTcGCCCGCATGGAGCTCTTCCTCTTCTTCACCaGCCTCCTGCAGCaCTTCAGCTTCTCGGTGCCtGCcGGGCAGCCCCGCCCCAGCaACCACGGTGTCTTTGcCttCCTGGTgaCCCCaGCCCCCTACCAGCTCTGTGCgGtGCCCCGCTAG

>CYP2D50_mouse

ATGGaGCTGCTGACtgGGacTggcCTGtGGCCTGTGGCcaTattCacaGtCATCTTCaTatTaCTGGTGGACCTGATGCACCGGCGCCAgCGCTGGaCttCtCGCTACCCaCCgGGCCCtgTGCCatgGCCTGtGCTGGGtAACCTGCTGCAGGTGGACcTggAtaACAtGccaTaCAGCTtCTGCGGgGtCGCTatGGGGACcTGTTCAGCCTaCAGCTGGCCTctgaGtCaGTGGTtGTaCTaAAtGGGCTGaCGGCCcTgCGaGAGGCaCTGGTGaAaCaCaGCGAGGACACtgCtGACCGgCCACcgcTGCattTcAatGAcCtgCTGGGcTTtGgaCCaGGtaTaGTCcTaGCaCGgTATGGGCctgCCTGGCGtcAGCAGCGGCGCTTCTCtGTGTCtACCaTGCaCcACTTtGGCCTGGGCAAGAAGTCaCTGGAGCAGTGGGTGACtgAGGAGGCCagaTgCCTCTGTGCCGcCTTCGCtGACCAtactGGtAGGCCtTTcAGCCCtaACaCCCTatTGgAcAAAGCaGTGtGtAACGTGATCGCgTCCCTcctCTaTGcctGCCGCTTTGAgTACgAtGACCCaCgCTTCCTcaCaCAGtTcCTGAAtGtGtTCCCgATGCTCCTGCgCATCCCGGGGCTGGTtGgCAAGGTCTTcCCTGGGaAaAGGGCCTTtgTtaCCatGTTGGATGAGCTGcTgGCTGAaCaCAaGAcGACCtGGGACCCtaCCCAGCCaCCcCGAGAttTGACTGATGCCTTCCTGGctGAGGTGgAGAAGGCCAAGGGGAAtCCtGAGAGCAGCTTCAATGATGAgAACCTGCGCacGGTaGTGggTGACCTGTTCtCtGCaGGGATGGTGACCACCCGaGTaCAACAGGAaATCGATGAaGTcATAGGGCAGGtGCaGtGtCCAGAGATGGcaGACCAGGCtCGcATGCCCTaCACCAatGCtGTcaTtCAtGAGGTgCAGCGCTTTGcaGACATtctCCCtcTtGGtGtaCCtCACAaGACtTCtCGTGACATTGAAcTaCAGGGCTTCCTtATcCCtAAGGGGACgACcCTCATCACCAACCTGTCcTCcGcGCTaAAaGAtGAGACtGTCTGGgAGAAGCCCcTCtGCTTCCAtCCtGAaCACTTCCTGGAtGCCCAGGGCCaCTTtGTgAAGCctGAGGCCTTCATGCCaTTCTCAGCAgGCCGCaGaTCaTGCCTgGGGGAGCCCCTGGCCCGCATGGAGCTCTTCCTCTTCTTCACCTGCCTCCTGCAGCGCTTtAGCaTCTCaGTGCCCGaTGGaCAGCCCCagCCCAGCGACCAtGGcGTCTTTaGggCtCTGacaaCCCCatgCCCCTACCAGCTCTGTGCTttGCCCCGCTAa

>CYP2E1_horse

ATGGCTGCCCTGGGCATCACGGTGGCCCTGCTGGTGTGGGTGGCCACCCTGCTGCTCATCTCCATCTGGAAGCAGATCTACAGCAGCTGGAACCTGCCCCCTGGTCCTTTCCCACTGCCCATCATTGGGAATCTTTTCCACTTGGACCTCAAGAATATTCCCAAGTCCTTCACCAGGCTGGCAGAGCGGTACGGGCCGGTGTTCACCCTGTACCTGGGCTCGCAGCGCGTCGTGGTCATGCATGGCTACAAGGCCGTGAAGGAGGTCCTGCTCAACTACAAGAATGAGTTGTCCGGCAGGGGAGAAATCGCCGTGTTCCAGGCGCACAAGGACAATGGAGTTATTTTCAATAATGGACCAAGCTGGAAGGACACCCGGCGGCTCTCCCTGACTATCCTCCGAGACTATGGGATGGGGAAGCAGCGCAACGAGGAGCGGATCCAGAGGGAAACCCACTTCCTGCTGGAGGCCCTCAGGAAGACCCAGGGCCAGCCCTTCGACCCCACCTTTGTCCTTGGCGGCGGGCCCTTCAACGTCATCGCCGACATCCTCTTCCACAAGCACTTTGACTACGAGGACAAGACGTGTCAGAGGCTGATGCACTTGTTCAACGAGAACTTCTACTTGCTCAGCACCCCCTGGCTCCAGGCTTATAATTATTTTTCAACCTATCTGCGCTACCTGCCTGGAAGCCATAGAAAAGTAATGAAAAATGTGTCTGAAATTAAAGAGTTTACTTCAGAAAGAGTGAAGGAGCACCATAAGTCACTGGACCCCAACTGCCCCCGAGACTTCACCGACAACCTGCTCATGGAAATGGAGAAGGAGAAACACAGTGCGGAGCCCCTGTTTACGTTGGAAAACATCACTGTGACCACGGCTGACATGTTCTTTGCAGGGACAGAGACCACCAGCACCACGCTGAGATACGGGCTCCTGATTCTCTTGAAACACCCGGAGGTTGAAGAGAAACTTCATAAAGAGATTGACAGTGTGATTGGGCCAAGCCGAATCCCTGCCTTCAAGGACAGACTAGAGATGCCCTACATGGATGCTGTGGTGCATGAGATTCAGCGATTCATCAACCTCGTGCCCTCCAACCTGCCCCATGTAGCAACCCAGGACACAGCGTTCAGAGGATATGTCATCCCTAAGGgcacagtcgtaattccgacactggattcactcttgtatgacaaccaagagttccctgatgcAGAGAAGTTTAAGCCAGAGCACTTTCTGAACGAAAACGGAAAGTTCAAGTACAGCGACCATTTCAAGGCATTTTCCGCAGGAAAGCGCGTGTGCGTCGGAGAAGGCCTGGCTCGCATGGAGCTGTTTCTGTTCCTGACTGCCATTCTGCAGCACTTTAACTTGAAGTCTCTGGTTGACCCCAAGGATATTGACCTCAGCCCCGTCACGATTGGGTTTGGCAACATCCCACCCAATTACAAGCTCTGCATCATTCCCCGCTCGTGAGCGCGAGGGACAGGTGCTCAGAAGACCCCTGGGCCCCTTGATGCCCTAGGGTTCCTGCCAGCGCCCACTCAGTGCCACCAGGAAGACCCTCCCCTCACGGCTCAGTGAGTCAGGGCTCCACGGGGATCACTCGGCCGGAGAGGCTGCTTCCCAGAATCATTCTTCAGATAGAATTTGAAAGCAAAGTCCAAAAAAGATTGTGTAAAACCAATTAAACTAACTAAAGAAACTG

>CYP2E1_rhino

ATGGCgGCCCTGGGCATCACaGTcGCgCTGCTGGTGTGGGTGGCCACCCTGCTGCTCgTCTCCATCTGGAAGCAGATCTACAGCAGCTGGAAaCTGCCCCCTGGcCCTTTCCCACTGCCCATCcTTGGGAAcgTTTTCCAggTGGACtTaAAGAAcATTCCCAAGTCCTTtACCAGGCTGGCAGAGCGtTACGGGCCaGTGTTCACCtTGcACCTGGGCTCGCgGCaCGTCGTGGTCtTGCATGGCTACAAGGCCGTGAAGGAaGTCCTcCTCAcCTACAAGAAcGAGTTtTCtGGCAGaGGAGAAATCcCCGcaTTCCAGGaGttCAAGGACAAaGGAGTgATTTTCAATAAcGGgCCgAcCTGGAgGGACACgCGGCGctTCTCCCTGACcgTCCTCCGtGACTATGGGATGGGGAAaCAGCGCAACGAGGAGCGGATCCAGcGGGAgACCCACTTCCTGCTGGAGGCgCTCAGGAAGACCCAGGGCCAGCCCTTCGACCCCACCTTTGTtaTcGGCGGtGGGCCCTTCAACGTCATtGCCGACATCCTCTTCCACgAGCACTTTGACTAtGAGGACAAGAtGTGTCtGAGGCTGATGCAtTTGTTCAACGAGAACTTCTACcTGCTCAGCACtCCCTGGCTCCAGGtTATAATTATTTTTCAAgCTATCTGCGCTACCTGCCTGGAAGCCATAGAAAAGTAATGAAAAATGTGTCcGAAATTAAAGccTacACTTtAGAAAGAGTGAAGGAGCACCATAAGTCgCTGGACCCCAACTGCCCCCGAGACTTCACtGACAgCCTGCTCgTGGAAcTGGAGAAGGAaAAgCACAGTGCaGAGCCtgTGTTcACcTTGGAAAACATtgCgGTGACCACGGCTGACATGTTCTTTGCgGGGACAGAGACCACCAGCACCACtCTGAGATAtGGGCTCCTGATTCTCcTGAAAtACCCGGAGaTTGAAGAGAAACTTCATAAAGAGATTGACAGaGTGATTGGGCCAAGCCGAATCCCTGCCaTCAAGGACAGACTAGAGATGCCCTACATGGATGCcGTtGTGCATGAGATTCAGCGATTCATCAACCTtGTGCCCTCCAACCTGCCCCATGcAGCAACCCAGGACACAGaGTTCAGAGGATATGTCATCCCcAAGGGCACgGTCcTAATTCCGACACTGGAcTCACTCTTGTATGACgACCAAGAGTTCCCTGATcCAGAGgAGTTTAAGCCAGAGCACTTTCTtAACGAAAAtGGAAAGTTCAAGTACAGtGACCATTTCAAGGCATTTTCCGCAGGAAAaCGgGTGTGtGTCGGAGAAGGCCTGGCTCGCATGGAGtTGTTTCTGTTCCTGtCTGCCATTCTGCAGCACTTTAACTTGAAGTCTCTGGTTGACCCCAAGGATATTGACCTCAGCCCCaTCACGATTGGGTTTGGCAgCATCCCgCCCcATTACAAGCTCTGCgTCATTCCgCGCTCaTGAGtGtGAGGcACAcGcGCTCtGAAGACCCCTGGGgtCCTTGATGCCtCTCAGTGAGTCAGGGCTCCAtGGGGATCACTCaGCCaGtGAcaCTGCTTCCCAGAgTCATTCTTCAGAcAGAATTTGAAAaCAAAGTCCAAAAAAGATTa

>CYP2E1_cow

ATGGCcGCCCTGGGCATCACGGTcGCCCTGCTGGTGTGGaTGGCCACCCTGCTGtTCATCTCCATCTGGAAGCAcATCTACAGCAGCTGGAAaCTGCCCCCTGGcCCTTTCCCACTGCCCATCATcGGGAATCTTTTaCAgcTGGAtaTtAAGAAcATTCCCAAaTCCTTCACCAGGCTGGCgGAGaGGTACGGGCCGGTGTTCACCCTGTACCTGGGCTCtCAGCGtGcCGTGGTCgTGCATGGCTACAAGcCCGTGAAGGAGGTCCTGCTtgACTACAAGAAcGAGTTtTCtGGCAGaGGAGAAAaCcCtGgGTTCCAGatGCACAAGaACAATGGtGTcATTTTCAAcAATGGAtCAAcCTGGcgGGACACCCGaCGGtTCTCgtTGACcAcCCTCCGtGACTtaGGGATGGGaAAaCAGgGCAAtGAGcAGCGGATCCAGAGGGAggCCCACTTCCTGCTGGAGGtgCTCAGGAAGACCCAGGGCCAGCCCTTtGACCCCACgTTTGTCgTcGGCtttGcaCCtTaCAAtGTCATCtCtGACATCCTCTTCCACAAaCgCTTTGACTAtaAaGAtcAGACGaGcCtGAGGCTGATGagtcTGTTCAACGAGAACTTCTACcTGCTCAGCAgCCCCTGGaTCCAGGtTTATAATaATTTcTCAgaCTAcCTaCagTACCTGCCTGGAAGCCATAGAAAgcTAcTGAAAAATGTGTCTGAAgTaAAAagtTaTgCTTtAGAAAGAGTGAAGGAtCACCAgAAGTCcCTGGAaCCCAgCTGCCCCCGAGgCTTCttgGACAcCaTGCTgATaGAAATGGccAAgGAaAgACACAGTGtGGAcCCtaTGTacACcTTGGAAAACATCgCTGTGACtgtGGCcGACcTGcTCTTTGCgGGGACAGAGACCACCAGCACCACcCTGcGATACGGGCTCCTGATTCTCaTGAAAtACCCGGAGGTTGAAGAGAAACTTCATgAAGAaATTGACAGgGTGATTGGGCCAAGCCGAATCCCTGCCgTCAAGGACAGgCTgGAcATGCCCTACcTGGATGCTGTGGTaCATGAGATTCAGCGATTCATCgACCTCcTtCCCTCCAAtCTGCtCCAcGaAGCAACCCAGGACACAGtGTTCAGAGGATATGTCATCCCcAAGGGCACAGTCGTAATTCCGACgCTGGAcTCtgTCTTacAcGAtAggCAAGAGTTtCCTGAacCAGAGAAGTTTAAaCCAGAGCACTTTCTGAAtGAAAAtGGAAAGTTCAAGTACAGtGACCATTTtAAGGCgTTTTCtGCAGGAAAGCGgGTGTGtGTtGGAGAAGGCCTGGCTCGCATGGAaCTGTTcCTGcTCtTGgCcGCCATcCTGCAGCACTTTAACTTGAAGTCaCTtGTTGACCCCAAGGATATcGACCTCAGCCCCaTtgCaATTGGGTTTGGCAAgATCCCgCCCcgTTACAAaCTCTGtcTCATTCCCCGCTCaaaAGtGtGAGGCAGAcAGAAcTTGAAAGCAAAGTgCAAAAg

>CYP2E1_dog

ATGGCcGCCtTGGGCATCACaGTGGCCCTGCTGGTGTGGaTGGCCACCtTaaTGCTtATCTCtATCTGGAAGCAGATCTACAGCcGCTGGAAaCTGCCCCCTGGcCCTTTtCCACTGCCCATCATTGGGAATaTTcTgCAggTGGAtaTCAAGAATgTTCCCAAaTCtcTCgCCAGCTGGCAGAGCaGTACGGGCCaGTGTTCACCtTGTACCTGGGCTCcCAGCGCacaGTGGTCcTGCATGGCTACAAGGCgGTGAAGGAaGTtCTGCTtgACcACAAGAATGAccTtTCtGGCAGaGGAGAAgTCttCGcaTTCCAGtCGCACAAGGACAgaGGtTTCAAcAATGGgCCcgGCTGGAAGGACACgCGGCGaCTCTCCCTGAgcAcCCTCCGgGACTAcGGcATGGGGAAGCgcgGCAACGAGGAGCGGATCCAGAGGGAgAtCCcCTTCCTGCTGGAGGCgCTCAGGggcACCCgGGGCCAGCCCTTCGACCCCACCTTTcTCCTgGGCttCGccCCCTTCAACGTCATCGCtGACATCCTCTTCCACAAGCACTTTGACTACtcGGAtcAGACtgGgCtGcGGaTacaGaAgcTGTTCAACGAGAACTTCcACcTGCTCAGCACCggCTGGCTCCAGGTTATAAcatTTTccCAAgCTATCTGCaCTACCTGCCcGGAAGCCATAGAAAAGTctTaAgAAATGTGgCTGAAcTaAAgGAtTacAgcTtAGAAAGgGTGAAGGAGCACCAggAGTCgCTGGACCCCAcCTGCtCCCGgGACTTCACtGACtgCtTGCTCcaGGAgGAGCCttgGTaTACcTTGGAcAACATtgCcGTGACCgtGGCTGACcTGTTCTTTGCgGGcACgGAGACCACCAGCACCACcCTGAGATACGGGCTCCTGATTCTCaTGAAAtACCCaGAGGTcGAAGAGAAACTTCATgAAGAaATcGACAGgGTGATcGGcCCAAGCCGAgTCCCTGCCaTCAAGGACAGgCTgGAGATGCCCTACATGGATGCcGTGGTGCAcGAGATTCAGCGATTCATCgACCTgcTGCCCTCCAACCTGCCCCATGTAGCAAaCCAGGACACgatGTTCAGAGGATATGTCATCCCcAAGGGCACAGTgGTAATTCCcACACTGGAcTCcgTCTTGTtTGACAAaCAAGAaTTCCCTGATcCAGAGAAGTTcAAGCCAGAGCACTTTCTGAAtGAAAACGGAAAaTTCAAaTAtAGtGACtAcTTCAAGGCATTcTCCGCAGGAAAGCGgGTGTGtGTtGGAGAAGGCCTGGCTCGCATGGAGCTcTTcCTGTTCtTGtCcGCCATTtTGCAGCACTTTAACcTGAAGTCTCTcGTcGACCCCAAGGATATTGACCTCAGtCCCtgCACaATcGGGTTTGcCAAgATCCCcCCCcATTACAAaCTCTGtgTCgTTCCCCGCTCGgTGCCtCCAGGAAGACCCTCCCTTTGAcAGCAAcGTCCAAAAAAGATTGTGcAAAACtAATTAAACaAAtTgA

>CYP2E1_pig

ATGaCTGCCCTGGGCATCACGGTGGCCCTGCTGGTGTGGtTGGtCACCCTGCTGCTCATCTCCATCTGGAAGCAcATCcACAGtAGCTGGAAaCTtCCCCCTGGcCCTTTCCCACTGCCCATCgTTGGGAAcaTTTTCCAgTTGGACCTtAAGAATATTCCCAAaTCCTTCACCACTGGCAGAGCGtTACGGGCCGGTGTTCACtgTGTACCTGGGtTCGCgGCGCaTtGTGGTCcTGCAcGGCTACAAGGCCGTGAAGGAGGTCtTGCTCcACTACAAGAATGAGTTcTCtGGCAGaGGgGAAATCcCCacGTTCCAaGtGCACAAGGACAAaGGgGTcATTTTCAATAATGGACCAAcCTGGcgGGACACtCGGCGGtTCTCCCTcACcAcCCTCCGtGACTtcGGGATGGGGAAaCAGgGCAAtGAGcAGCGGATCCAGAGGGAggCCCACTTCCTGCTGGAGGCaCTCAGGAAGACCCatGGCCAGCCCTTtGAtCCCACCTTccTCaTcGGCtGCGcaCCCTgCAAtGTCATCtCCGACATCCTCTTCCgCcAGCACTTTGACTACaAtGACAAGACcTGTCtGAGGtTaATGagCaTGTTCAAtGAGAACTTCTACcTGCTCAGtACtggCTGGCTCtAGctTTATAATaATTTcTCAggCTATCTaCGCTACCTGCCTGGAAGCCATAGgAAAcTAATGAAAAATaTaTCTGAAATaAAAGAtTaTgCTTtAGAAAGAGTGAAGGAcCACCgggAtTCACTGGAgCCCAgCTGtCCtCGAGAtTTCACtGACAcCCTGCTgATGGAAATGGAGAAGGAaAAAtACAGTGCaGAaCCtaTaTacACcTTGGAcAACATtgCcGTGACCgtGGCcGACATGTTCTTTGCgGGGACAGAGACCACCAGCACCACcCTGAGATACGGGCTCCTaATTCTCaTGAAAtACCCaGAGGTTGAAGAGAAACTTCATgAAGAaATTGACAGgGTcATTGGtCCAAaCaGAATCCCTGCCaTCAAGGACAGgCTgGAcATGCCCTACcTGGATGCcGTGGTaCATGAGATTCAGCGATTCATCgACCTCaTtCCCTCCAACCTGCCaCATGaAGCAACCCgGGACACAGtaTTCAGAGacTAcaTCATCCCcAAGGGCACAGTgGTAATTCCGACACTGGAcTCcgTCTTaTATGACAgCCAAGAaTTCCCTGAgcCgGAGAAGTTTAAGCCAGAGCACTTTCTGAAtGAAAACGGAAAGTTCAAGTACAGtGAtCATTTCAAGGCATTTTCCGCAGGAAAGCGgGTGTGtGTCGGAGAgGGCCTGGCTCGCATGGAaCTGTTcCTGTTCaTGgCTGCCATctTGCAGCACTTTAACTTGAAaTCTCTtGTTGACCCCAAGGATATcGACCTCAGCCCCaTCACGATTGGGTTTGcCAAgATtCCcCCCcATTACAAaCTCTGtgTCATTCCCCGCTCacaAGtGtGAGGGAgAtGTGCTCgaAAGgCCCGCCaGtGAaGCTGCTTCCagcccaCATcCTTCAGATAGAATTTGAAAGCAAAGTCCAAAAAAGATTtTGTAcAAtCAATTAAAgTAAgTAAAG

>CYP2E1_elephant

tTGCTtCTCtTtTCacTCTGGAAaCAGAgCTACgGgAaagGGAAgCTcCCgCCTGGcCCcactCCtCTcCCaATtATTGGaAATagccTgCAgTTaaAttTtAAGAATATcagCAAaTCtTAtGGcCCtGTGTTCACCCTGTAttTGGGCatGaAGCcCacaGTGGTgtTGCATGGgTAtgAaGCaGTGAAGGAaGcCCTGaTtgAtcAgggagAgGAGTTtTCtGGaAGaGGcGAaAAggACAaTcaaGAGtCtgTaTTTACtaTtGAAAACtTgAtaaTcACtAtaagTGAtgTGTTtTcTGCtGGGACAGAGACaACaAGtACCACaCTGAGgTAtGGaCTCCTGcTTCTgcTaAAgCACCCaGAGGTcAGAGATTGACcGTGTGgTTGGcCgAcaCCGgAgCCCctgCaTgcAGGACAGgagcagcATGCCCTACAcGGATGCTGTGGTGCAcGAGATcCAGaGATaCATtgACCTtaTcCCCaCCAACCTGCCCCATGcgGCgACCCAaGACAttaaaTTCAGgaacTAccTCATCCCcAAGGtaAgAtTtGTttcTCCtACAGGAAAaCGtaTtTGtcTgGGAGAgGGCCTGGCcCGCATGGAGCTGTTTtTaTTCtTcACTGCCATTtTaCAGaACTTTAcCTTGAAGcCTCTGGTTGACCCaAAGGAcATcGAtacCAaCCCaGTtgaaAaaGGGTTTGcCtctgTgCCACCCAAgTACcAGCTCTGCtTtATTCCt

>CYP2E1_human

ATGtCTGCCCTcGGagTCACcGTGGCCCTGCTGGTGTGGGcGGCCttCCTcCTGCTggTgTCCATgTGGAgGCAGgTgcACAGCAGCTGGAAtCTGCCCCCaGGcCCTTTCCCgCTtCCCATCATcGGGAAcCTcTTCCAgTTGGAatTgAAGAATATTCCCAAGTCCTTCACCcGGtTGGCccAGCGcTtCGGGCCGGTGTTCACgCTGTACgTGGGCTCGCAGCGCaTgGTGGTgATGCAcGGCTACAAGGCgGTGAAGGAaGcgCTGCTggACTACAAGgAcGAGTTcTCgGGCAGaGGcGAccTCcCCGcGTTCCAtGCGCACAgGGACAggGGAaTcATTTTtAATAATGGACCtAcCTGGAAGGACAtCCGGCGGtTtTCCCTGACcAcCCTCCGgaACTATGGGATGGGGAAaCAGgGCAAtGAGagcCGGATCCAGAGGGAggCCCACTTCCTGCTGGAaGCaCTCAGGAAGACCCAaGGCCAGCCtTTCGACCCCACCTTccTCaTcGGCtGCGcGCCCTgCAACGTCATaGCCGACATCCTCTTCCgCAAGCAtTTTGACTACaAtGAtgAGAaGTtTCtaAGGCTGATGtAtTTGTTtAAtGAGAACTTCcACcTaCTCAGCACtCCCTGGCTCCAGGTTAcAATaATTTTcCcAgCTtTCTaCaCTACtTGCCTGGAAGCCAcAGAAAAGTcATaAAAAATGTGgCTGAAgTaAAAGAGTaTgtgTCtGAAAGgGTGAAGGAGCACCATcAaTCtCTGGACCCCAACTGtCCCCGgGACcTCACCGACtgCCTGCTCgTGGAAATGGAGAAGGAaAAgCACAGTGCaGAGCgCtTGTacACaaTGGAcggtATCACcGTGACtgtGGCcGACcTGTTCTTTGCgGGGACAGAGACCACCAGCACaACtCTGAGATAtGGGCTCCTGATTCTCaTGAAAtACCCtGAGaTcGAAgAGAAgCTcCATgAAGAaATTGACAGgGTGATTGGGCCAAGCCGAATCCCTGCCaTCAAGGAtAGgCaAGAGATGCCCTACATGGATGCTGTGGTGCATGAGATTCAGCGgTTCATCAcCCTCGTGCCCTCCAACCTGCCCCATGaAGCAACCCgaGACACcattTTCAGAGGATAccTCATCCCcAAGGGCACAGTCGTAgTgCCaACtCTGGAcTCtgTtTTGTATGACAACCAAGAaTTtCCTGATcCAGAaAAGTTTAAGCCAGAaCACTTcCTGAAtGAAAAtGGAAAGTTCAAGTACAGtGACtATTTCAAGcCATTTTCCaCAGGAAAaCGaGTGTGtGctGGAGAAGGCCTGGCTCGCATGGAGtTGTTTCTtTTgtTGtgTGCCATTtTGCAGCAtTTTAAtTTGAAGcCTCTcGTTGACCCaAAGGATATcGACCTCAGCCCtaTacatATTGGGTTTGGCtgtATCCCACCacgTTACAAaCTCTGtgTCATTCCCCGCTCaTGAG

>CYP2E1_sheep

ATGGCcGCCCTGGGCATCACGGTcGCCCTGCTGGTGTGGaTGGCCgCCCTGCTGCTCATCTCCATCTGGAAaCAcATCTACAGCAGCTGGAAaCTGCCCCCTGGcCCTTTCCCACTGCCCATCATTGGGAATCTTTTgCAacTGGAtaTtAAGAAcATTCCCAAGTCCTTCACCAGGCTGGCAGAGaGGTtCGGGCCGGTGTTCACCCTGTACCTGGGtTCtCgGCGCGTCGTGGTCgTGCAcGGCTACAAGcCCGTGAAGGAGGTCCTGCTtgACTACAAGAAcGAGTTtTCtGGCAGaGGAGAAAaCcCtGgGTTCCAGGtGCACAAaaACAAcGGtGTcATTTTCAAcAATGGACCAAcCTGGcgGGACACCCGGCGGtTCTCgtTGACcATCCTCCGtGACTtaGGGATGGGcAAaCAGgGCAACGAGcAGCGGATCCAGAGGGAggCCCACTTCCTGCTGGAcGtgCTCAGGAAGACCCAGGGCCAGCCCTTtGACCCCACaTTTGTCaTcGGCttCGctCCtTaCAAtGTCATCtCtGACATCCTCTTCCACAAGCgCTTTGACTAtaAaGACAAGACGgGgCtGAGGCTGATGagtcTGTTCAAtGAGAACTTCTACcTGCTCAGCACCCCCTGGaTCCAGGTACCTGCCTGGAAGCCATAGAAAgcTAcTGAAAAAcGTGTCTGAAAGGAaAAACACAGTGCGGAcCCCaTGTacACcTTaGAAAACATCgCTGTGACtgtGGCcGACcTGcTCTTTGCAGGGACgGAGACCACCAGCACCACcCTGcGATAtGGGCTCCTGATTCTCaTGAAAtACCCaGAGGTTGAAGAGAAACTTCATgAAGAaATTGACAGgGTaATTGGGCCAAGCCGAATCCCTGCCaTCAAGGACAGgCTgGAcATGCCCTACcTGGATGCTGTGGTGCATGAGATTCAGCGATTCATCgACCTCaTtCCCTCCAAtCTGCtCCATGaAGCAACCCAGGACACAGtGTTCAGAGGATATGTCATCCCcAAgGGCACAcTCaTAATTCCGACgCTGGAcTCtgTCTTGTATGAtAAgCAAGAGTTtCCTGAacCAGAGgAGTTTAAGCCAGAGCACTTTCTGAAtGAAAgtGGAAAGTTCAAGTACAGtGACCATTTCAAGGCgTTTTCtGCAGGAAAGCGgGTGTGtGTtGGAGAAGGCCTGGCTCGCATGGAaCTGTTcCTGTTCtTGgCcGCCATcCTGCAGCACTTTAACcTGAAGTCaCTtGTTGACCCgAAGGATATcGACCTCAGCCCCaTtgCaATTGGGTTTGGCAAgATCCCgCCCcATTACAAaCTCTGtcTCATTCCCCGCTCagaAGtGtGAGG

>CYP2E1_mouse

ATGGCgGttCTtGGCATCACcGTtGCCtTGCTtGTcTGGaTcGCCACCCTcCTcCTCgTaTCCATCTGGAAaCAGATCTAtAGaAGtTGGAACCTGCCCCCaGGaCCTTTCCCAaTtCCttTCtTTGGaAAcaTTTTtCAgcTGGAttTgAAGgATATTCCCAAGTCtTTaACCAGGtTGGCAaAGCGcTtCGGGCCaGTGTTCACaCTGcACCTGGGtcaGagGCGCaTCGTGGTCcTGCATGGCTACAAGGCtGTcAAGGAGGTgCTaCTgAACcACAAGAATGAGTTcTCtGGCcGaGGgGAcATtcCtGTGTTCCAGGaGtACAAGaACATTATTTTCAATAATGGACCcAcaTGGAAGGACGGCCAGCCtTTtGACCCtACCTTTcTgaTTGGCtGtGcaCCCTgCAAtGTCATtGCgGAtATtCTCTTCaACAAaCgCTTcGAtTACGAtGACAAGAaGTGTCtGgaGCTcATGagtTTGTTCAAtGAaAACTTCTACcTGCTgAGtACtCCCTGGaTCCAGGCTTAcAATTAcTTTTCggatTATCTaCaaTAtCTaCCTGGAAGCCAcAGAAAAGTcATGAAAAATGTGTCTGAAATaAgAcAGTacACacttGgAAaAGccAAGGAaCACCtTAAGTCACTGGACatCAACTGCCCCCGgGAtgTgACtGACtgtCTcCTCATaGAgATGGAGAAGGAaAAACACAGccaaGAaCCCaTGTacACaaTGGAAAAtATttCTGTGACtttGGCcGACcTGTTCTTTGCAGGaACAGAGACCACCAGCACaACtCTGAGATAtGGGCTCCTGATTCTCaTGAAAtACCCaGAaaTTGAAGAGAAACTTCATgAAGAaATTGACAGgGTtATTGGGCCAAGCCGtgcCCCTGCagTCcgaGACAGgaTgaAtATGCCCTACATGGAcGCTGTaGTGCATGAGATTCAGaGATTCATCAACCTCGTcCCtTCCAACCTGCCCCAcGaAGCAACCCgaGACACcGtGTTCcGAGGATATGTCATCCCcAAGGGtACAGTtGTAATTCCaACtCTGGAcTCcCTtTTaTtTGACAACtAtGAGTTtCCaGATcCAGAGAcaTTTAAaCCtGAGCAtTTTCTGAAtGAAAAtGGgAAGTTCAAGTACAGtGACtATTTCAAGGCgTTTTCtGCAGGAAAGCGCGTGTGtGTtGGAGAAGGCCTGGCcCGCATGGAaCTGTTTCTGcTttTGtCTGCtATTCTGCAGCAtTTTAAtcTGAAGTCTCTGGTTGACCCtAAGGATATcGACCTCAGCCCtGTtACaATTGGcTTTGGCAgtATCCCACgCgAaTttAAaCTCTGtgTCATTCCtCGtTCaTGA

>CYP3A89_horse

GGAAGGAAGCTCTGTGCCCACAGGCCTGCAAACAGTAGCACCGACTGCTGAAAGGAAAACTCAGAGGAGAGAATCTCAGAAGGAAAGTGGCCATGGACCTGATCCCAAGCTTTTCCATGGAAACCTGGGTTCTCCTGGCTACCAGCCTGGTGCTCCTCTATCTATACGGGACCTACACTCATGGACTTTTTAAGAAGCTAGGAATTCCTGGGCCAACACCTCTGCCTTTTTTTGGAAATGTTCTGAGTTACCATAAGGGTATTTGGGATTTTGATAAGAAATGTTTTGAAAAGTATGGAAAAATGTGGGGGACTTATCATGGCACAAAACCTGTGCTGGCTATCACAGATCCAGACATGATCAAAACAGTACTAGTGAAAGAATGCTATTCTGTCTTTACAAACCGGCGGCCTTTTGGTCCATTCGGATTTATGAAAAGTGCCATCTCTCTGTCTGAGGATGAACAATGGAAGAGAATACGAACATTGCTGTCTCCAACCTTCACGAGTGGAAAGCTCAAGGAGATGTTCCCCATCCTTGGCCAGTATGGAGACGTGTTGGTTAGGAACCTGAAGAAGGAAGCAGAGAAAGGCAAGCCCATCACCTTGAAAGACATCTTTGGGGCCTACAGCATGGATGTGATTACTAGCACATCATTTGGAGTGAACATCGACTCCCTCAACAATCCACAAGATCCCTTTGTGGAAAATACCAAGAAGCTCTTCAGTTTTGATTTCCTTGATCCATTACTTCTCTCAATAACACTCTTTCCATTTCTTAATGCAGTTTTTGAAGTATTAAATGTCTTTGTGTTTCCAAAAAGTGTTACTGATTTTTTCATAAAATCTGTAAAAAGGATGAAAGAAAGTCGCCTCAAAGATAAAGAAAAGCACCGAGTTGATTTTCTTCAGCTGATGATTAACTCTCAGAATTCAAAAGAACTGGACACCCATAAAGCTCTGTCTGATCTGGAGCTCGTGGCCCAATCTATTATCTTTATTTTTGCTGGCTATGAGACCACTAGCAGTTCTCTTTCCTTCCTTATGTATTTTTTGGCCACTCACCCTGATGTCCAGCAGAAGCTGCAGGAGGAGATTGATGcGACTTTCCCCAATAAGGCTCCTCCCACCTATGATGCCCTGGTACAGATGGAGTATCTTGACATGGTGTTGAATGAATCCCTCAGGTTATTCCCAATTGCTGTTAGACTTGAGCGGGTCTGTAAGAAAGATGTGGAAATCAATGGGGTGTTCATTCCTAAAGGGACAGTGGTGATGGTGCCAACCTTTGCTCTTCACAAACACCCAGAGTTCTGGCCAGAGCCTGAGGAGTTCCGTCCTGAAAGGTTCAGTAAGGAGAACAAGGACAGCATAAATCCTTATATATACCTGCCCTTTGGAGCTGGACCTCGAAACTGCATTGGCATGAGGTTCGCTCTGATGAACATGAAACTTGCTCTTGTCAGAATGCTGCAGAACTTCTCCTTCAAACCTTGTAAAGAAACACAGATCCCCCTGAAATTAGGCAATCAAGGACTTCTTCAACCACAAAAACCCATTGTTCTAAAGGTTGAGTCCAGAGATGGGACAGTGAGTGGAGCCTGACTTTCCACAAGGACTTCTGTTTTGTTCTTCAAGGAAGTTGTATCTCAGAACATCAGAGACCTCAATTTACTTTATGAATAAAACCCGGAATGAAGATGGGCTTAACCTACTGTATTTGATGGATGCCTAGAGATTCTTGCATTCTTTGAAATTTCTCAATGCCTATGTAGAGTATTATATGCTGTGTGATATAAAGGAGGGGGCTATATAAGTGTCAGATATATGGACTCAGCTTATCTGGTTCTCACAGGATGAGCTCCATCCACCCCCAGTTAGTACCATCTATTTTTCCTGAGCACTGATCAAGAATAAAACTTTCTCAACAATTTTATCAACAAACTTTAATGAAAAAAAATTATTGTGCTGACTGTAGTAGTGACATTTATATTTCATGTTCATTTTGAATCTACTATGAAGCATTATACTGAGCAAGCCAATAAATAGCTCTTTACAAaaaaaaaaaaaaaaaaaaaaaaaaaaaaaaaaaaaaaaaaaaaaaaaaaaaaaaaaaaaaaaaaaaaaaaaaaaaaaaaaaa

>CYP3A89_rhino

ACAaTAGCACtGACTtCTGAAAGGAAAACTCAGAcGAcAGAATaTCAGAAGGAAAGTGGCCATGGACCTGATCCCAAGCTTTTCCAcaGAAACCTGGGTTCTCCTGGCTACCAGCCTGGTGCTCCTCTATCtgTAtGGGACCTAtACTCATGGcCTTTTTAAGAAGCTgGGAATTCCTGGGCCgACACCTCTGCCTTTTTTgGGAcATaTTCTGAGcTACCATAAGGGTtTTTGGGATTTTGAcAAtAAATGTTTTaAAAAGTATGGAAAAATGTGGGGgTTATgATGGtAgAcggCCTGTGtTGGCTATCACAGATCCAGACATGATCAAAACAGTACTAGTGAAAGAATGtTATTtTGTtTTcACAAACCGGCaGCCTTTTGGTCCAcTgGGATTTATGAAAAcTGCCgTCTCTCTGTCTGAGGATGAAaAATGGAAGAGAATACGAAtgTTGCTGTCTCCAACCTTCACGAGTGGAAAGCTCAAGGAGATGTTCCCCATCaTTGGCCAGTATGcAGACaTGTTGGTgAGtAACCTGAgGAAGGcAGCAGAGAAAGGCAAGCCCATCACCTTGAAAGaCATCTTTGGGGCCTACAGCATGGATGTGATTACcAGCAtATCATTTGGAGTGAACATCGAtTtCCTCAACAAcCCACAAGATCCCTTTGTGGAAAATgCCAAGAAGCTCTTtAGaTTTGATTTCCTcaATCCATTcaTTCTCTCAATAACtCTCTTTCCAaTTgTTAATcCAGTTTTTGAAtTATTAAATaTCTcTcTaTTTCCAAAAAGTGTTACTGATTTTTTCAcAAAATCTGTAAAAAGGATGAAAGAAAGcCGCCTCAAAGATAAgGAAAAGCACCGAGTgGATTTTCTTCAGCTGATGATTAACTCcCAGAATTCcAAAGAACTGGACACCCATAAAGCTCTGTCTGATCTGGAGCTtGTGGCCCAATCTATTATCTTTATTaTTGCTGGCTATGAGACCACTAGCAGTTCTCTTTCCTTCCTTATGTATcTTTTGGCCACTCACCCTGATGTCCAGCAGAAGCTGCAGGAGGAGATTGATGaGACTTTtCCCAATAAGGCaCCTCCCACaTAcGATGCtCTGGTACAGATGGAGTATCTTGACATGGTGTTGAATGAATCtCTCAGaTTATTCCCAATTGtTGgTAGACTacAGaGGGTCTGTAAGAAAGATGTGGAAATCAATGGGGTGTTCATTCCcAAAGGGACAcTGGcGgTGGTGCCcACaTTTGCTCTTCACcgAgcCtCgGAGTTCTGGCCAGAGCCTGAGGAGTTCCGTCCTGAAAGGTTCAGTAAGaAGAACAAGGAtAGCATAAATCCTTActTATACCTGCCCTTTGGAaCTGGACCTCGcAACTGCATTGGCATGAGGTTCGCTgTtgTGAAtgTGAAACTTGCTgTcGTCAGAATGCTGCAGAACTTCTCCTTtAAACCTTGTAAAGAAACACAgATCCCCCTGAAAcTAGGCAggCgAGGAgTTCTTCAACtgCAAAAACCCATTGTTCTAAAGGTTGAGTCCAGAGATGGGACtGTGAGTGGAGCCTGACTcTCCctAAGGACTTCTGTTTTGTTgTTCAAGGAAGcTGTATCcCAGAACAcCAGAGAtCTCAATTTACTTTgTGAATAAAACCCaGAATGAAtATGaGCTTAACCcgCTGTgcTTGAcGGAaGCCcAGgGATTCTTaCATTCaTTGcAcTTTCTCAgTGtCTgTGTtGAGTATTATATGtTGTtcaATATAAAGGAaGGGcCTAAGTGcCAGATATgTGGACTCAGCTTATCTGtTTCTCACAGGAcaAtCTCCATCCACCCCAGcTAtTACCgTCTAcTcTTCCTGAGCACTGATCAAGAATgAAAgTTTCTCAACAATTgTATCAACAAACTTTAATGAAAccAAATTATTGTaCTGAtTGTAGTAGTGACATTgcTATcaCATGTTCATTTTGAATCTtCTATGAAatAgTATACTGAGCAAGtCAATAAATAtCTCTTTgCAA

>CYP3A89_cow

ACaGCTGAAAGGAAACTCAGAGGAGgGAATCgAAGaAAAGTGGCCATGGAgCTaATCCCAAaCTTTTCCgTGGAAACCTGGGTTCTCCTGGCTAtCAGCCTGGTGCTCCTCTATCTATAtGGaACtTAttCaCATGGACTgTTTAAGAAGCTgGGggTTCCTGGcCCAAgACCTCTGCCTcTTTTTGGAAAcGTTCTatccTACCgaAAGCCTcTGtTGGtTATCACgGATCCAGACATGATCAAAACAGTACTAGTGAAAGAATGtTATTCTGTCTTcACAAACCGGaGGTTTTGGTCCAaTgGGAgTTATGAAAAaTGCtgTtTCTgTGgCTGAGGATGAACAATGGAAGAGAATACGgACATTGCTGTCTCCAACCTTCACcAGTGGgAAGCTCAAGGAgATGTTCCCtATCaTTGGgaAGTATGGAGAtGTGTTGGTgAGGAACCTGAgGAAGGAAGCAGAGAAAGGCTCTTTGGGGCCTACAGCATGGATGTGATTACTAGCACATCATTTGGAGTGAAtATtGAtTCCCTtggCAAcCCACAAGATCCCTTTGTGGAgAATgCCAAGAAGCTCTTaAGaTTTGATaTCCTTGATCCATTtCTaCTtTCagTAgtACTCTTTCCATTcCTTAtccCAaTcTTTGAAGTATTAAATaTCagcaTaTTTCCAAAAAGTGcTgtgaATTTTTTgAcAAcATCcGTAAAAAaGATaAAAGAAAGTCGCCTCAAAGATActcAAACGtGTgGAcTTTCTTCAGCTGATGATTAACTCcCAGAATTCcAAAGAAacaGACAatCATAAAGCTCTcTCTGAcCaaGAaCTCaTGGCCCAgagTATTATCTTTATTTTTGgTGGCTATGAGACCACTAGCAcTTCTCTTTCCTTCaTTATaTATgaaTTGGCCACTCACCCTGATGTCCAGCAGAAGCTGCAGGAGGAGATTGATGCGACTTTCCCCAATAAGGCgCCTCCgACCTATGATGtCCTGGcACAGATGGAGTATCTTGACATGGTGgTGAATGAgaCtCTCAGaaTgTTtCCtATTGCTGTTAGACTTGAGaGGtTCTGTAAGAAgGATGTGGAAATCcATGGGGTGTcCATTCCcAAAGGGACAacGGTGAcGGTGCCAAtCTcTGtgCTTCACAgAgACCCAcAGcTCTGGCCAGAGCCTGAGGAGTTCCGTCCTGAAAGGTTCAGTAAGaAGAACAAGGACAGCATAAATCCTTAcgTcTACCTGCCtTTTGGAaCTGGACCcCGAAAtTGCATTGGCATGAGGTTtGCTaTcATGAACATGAAACTTGCTgTTGTCAGAgTcCTGCAGAACTTCTCCTTCAAACCTTGTAAAGAAACACAGATCCCCtTGAAAaTAaaaAgTCAAGGACTTtTaagACCggAAAAACCCATTGTTCTgAAGGTTGtGctCAGAGATGaGACcaTaAGTGGAGCtTGACTTcCCctAAGGACcTggGcTTTGTTCTTCAAGGAgCAGATATgTacACTCAGCTTAgCTGaTTCTCAaAGGAccAtCTCCAcCCACCCCCAGTTAGTAaCATCTAcTcaTtCaGAGCAaTGATCAAcAAagAAAgTgTCTCAACAgTTTTATCAgCAAAtTaatgaaAAcAAgAATTATTGTGgTGACTGaAGTtcTGACATTTAT

>CYP3A89_dog

CAGAAGaAAAGTGGCCATGGACCTaATCCCAAGCTTTTCCATGGAAACCTGGcTTCTCCTGGCTACCAGCCTGGTGCTCCTCTATCTgTAtGGGACCTACACaCATGGggTTTTTAAGAAGCTAGGAATTCCTGGaCCAACACCTCTGCCTTTTgTgGGAAcTGcTCTGgGcTACCgTAAGTATgATGGgcgAcAgCCTGTGCTGGCTATCACAGATCCgGACATGATCAAAACAGTgCTAGTGAAAGAATGtTATTCTGTCTTcACAAACCGGCGGCTTTTGGTCCAgTgGGATTTATGAAAAGTGCCATCTCTCTGTCTGAGGATGAAgAATGGAAGAGAATACGAACATTGCTGTCcCCAACCTTCACcAGTGGAAAGCTCAAGGAGATGTTCCCCATCaTTGGCCAGTATGGAGAtGTGTTGGTgAGGAACCTGAgGAAGGAgGCAGAGAAAGGCAAatCCATCAaCTTGAAAgCATCTTTGGaGCCTACAGCATGGATGTGATTACcAGCACATCATTTGGAGTGAACATtGAtTCCCTCAACAAcCCACAAGATCCCTTTGTGGAAAATAtCAAGAAGCTCTTaAaaTTTGATTTCCTTGATCCATTttTctTCTCAATAACTgTTTCCATTcCTTAcccCAGTTTTTGAAGTATTAAATaTCTggcTcTTTCCAAAAAGTGTTACTGATTTTTTCAcAAAATCTGTAAAAAGaATGAAgGAAAaTCGCCTCAAAGATAAAcAAAAGCAtCGAGTgGAcTTTCTTCAGCTGATGATTAACTCcCAGAATTCtAAAGAAacaGACACtCATAAAGCTCTaTCTGATtTGGAGCTgGTGGCCCAATCTATTATCTTTATTTTTGCTGGCTATGAGACCACTAGCAcTTCTCTTTCCTTCCTTATGTATgaaTTGGCCACTCACCCTGATGTCCAGCAGAAaCTGCAGGAGGAGATTGATGCGACTTTCCCCAATAAGGCattgCCCACtTAcGATGCCCTtGTgCAGATGGAaTATCTgGACATGGTGTTGAATGAAaCtCTCcGaTTATaCCCAATcGCTGgTAGACTTGAGaGGGTCTGTAAGAAAGATGTGGAAATCAgTGGtGTGTTCATTCCcAAAGGGACAGTGGTGATGGTGCCAACCTTTaCTCTTCAtcgAgACCagagtcTCTGGCCAGAGCCTGAGGAaTTCCGaCCTGAAAGGTTCAGTAgGaAGAACAAGGACAGCATAAATCCTTATAcATACCTGCCtTTTGGAaCTGGACCcCGAAACTGCATTGGaATGAGGTTtGCgaTcATGAACATGAAACTTGCcCTTGTtAGggTcCTGCAGAACTTCTCCTTCAAACCTTGTAAAGAAACACAGATCCCCCTGAAATTAaatgcTCAAGGgaTTaTTCAACCtgAAAAgCCCATTGTTCTcAAGGTTGAGcCaAGAGATGGGAgtGTaAaTGGAGCCTGACTTTCCctAAGGACTTCctCAGAGACCTCAATTTtCTTTATGAgTAAAACCCAATGAAGATGGGCTTATAAGTGTCAGAGTACCATCTATCCTGAGaACTGATCAAGAATAAAAaTTTCTCAACAATTTTgTCAACgAACTTTAAT

>CYP3A89_pig

AGGAcgGTGGCCATGGACCTGATCCCAAGCTTTTCtgTGGAAACCTGGcTgCTtCTGGCTACCAGCCTGGTGCTCCTCTATCTATAtGGGACCTAttCaCAcGGACTTTTTAAGAAGCTgGGgATTCCcGGGCCgAaACCTCTGCCTTaTTTTGGAAgTGTTCTagaaTACCgTAAGTATgAcGGtAgAcAgCCTGTGCTGGCTATgAgAGAcCCAGAaATGATCAAAgCAGTgCTtGTGAAAGAATGtTAcTCgGctTTcACAAAtCGGCGGTTTTGGTCCAgcgGGAgTTATGAAAAaTGCCATCTCTgTGgCTaAGGATGAgCAATGGAAGcGAgTACGAACATTGCTGTCTCCAACCTTCACcAGTGGAAAGCTCAAaGAgATGTTCCCCATCaTTGcCCAGTATGGAGAtaTGcTGGTgAGGAACCTGAgGAAGGAAGCAGAGAAAGGCAAGtCCATCAaCacGAAAGAATCTTTGGGGCCTACAGCATGGAcGTGATcACaAGCACAgCATTTGGAGTGAACTTTGATTTCCTcGATCCATTtaTTCTCTCAATAACAtTaTTTCCATTcCTcAgccCAtTcTTTGAAGTATTAgAcaTCacTtTGTTTCCAAgAAGTtcTgtgaAaTTTTTCAcAcAgTCTGTAAAAAGGATGAAAGAAAGTCGCCTCAAAGATcAAcAAACGAGTgGAccTTCTTCAGCTGATGATTAACTCcCAGAATTCcAAAGAAacGGAtgCCCATAAAGCTCTGTCTGATCaaGAaCTtGTGGCCCAAagTATTATCTTcATTTTTGCcGGCTATGAGACCACTAGCAGTTCTCTcTCCTTCCTggcGTATaTacTGGCCACTCACCCTGAcGTCCAGCAGAAGCTGCAGGAGGAGATTGATGCGACcTTCCCCAgcAAGGCg

>CYP3A89_elephant

AGCACacACaGCTtgAAGGAAAAgTCAGAGGAGActcTCcggcAAGGAAAGTGaCgATGGACCTGATCCCAAGCTTTTCCATGGAgACCTGGaTTCTtCTGGtcACCAtCCTGGTaCTCCTCTATagATAtGGaACCcAttCaCATaatgTTcTaAAGAAGCTtGGgATTCCTGGGCCcAaACCTtTGCCTTTTTTgGGAAcTtcTCTGgccTACCtcAAGTATgATGGtcgAcAgCCTGTGaTaGCcATCACAGATCCAGtCATGATCAAgACAaTACTgGTGAAAGAATGtTATTCcacCTTcACAAAtCGGaGGGGTCCAaTgGGATTTATGAAAtcTGCtATtTCctTGTCcGAGGATGAAgtgTGGAAGAGAgTACGAACATTGCTGTCTCCAACtTTCACcAGTGGAAAaCTCAAGGAGATGTTCCCCATCaTcaGCCAaTATGGtGAtGTGTTGGTgAaGcAtCTGAgagAGGAAGaAcAGAAAGGCAAGCCtgTtgCgTTGAAAGACATCTTTGGaGCtTACAGCATGGATGTGATaACTAGCACgTCATTTGGAGTGAACATCGAtTCCCTCcACAAcCCACAAGATCCCTTTGTGcAAAAcgCCAgGAAGCTCaTaAagTTTGATTTCtTgGAcCCAcTtCTTgTCcCtATAgCACTCTTTCCATTcCTTAcTcCAaTTTgTGAAGcATTAAAcaTCTcTcTGTTTCCAAgAgacGcTACTGATTTTTTCAcAAAATCTGTAcAAAGaATGAAAGAAAGcCGCCTtAAAGATAAtGAAAAGCACCaAGTgGATTTgCTTCAGCTGATGATcgACTCcCAGAATTCcAAAGAAaTtatgtCCCATAAAgCTCTGaCTGATacGGAGCTCGTGGCtCAATCaATTATgTTTATTTTTGCTGGCTATGAGACCACTAGCAcTgCTCTTTCCTTCgTTATGTATTTaTTGGCCACaCACCCTGATaTtCAGgAGAAaCTGCAGagGGAGATTGATGCagCTTTtCCCAATAAGGCaCCTCCgACgTATGAcGCCacGtTACAGATGGcGTATCTTGACATGGTGgTGAAcGAAaCaCTCAGaTTATTCCCAATcGCTGgcAGACTTGAGaGGGTCTGTAAGAAAGATaTtGAAATCAATGGaGTGacCATTCCcAAAGGGACAaTcGTGATGGTGCCAAtCTTTGtTCTTCACcgAgACgCAGAacaCTGGCCAGAGCCTGAGaAGTTCatgCCTGAAAGGTTCAGTAAGGAGAACAAGGACAaCgTAgATCCTTActTATAtCTGCCCTTTGGAaCTGGACCcCGAAACTGCATcGGCATGAGGTTtGCTCTcATGAACATGAAgCTTGCTaTcaTCAaAATtCTGCAagAgTTCTCCgTCAAACCTTGTAAAGAAACACAGATCCCCtTaAAATTAGGCAgggAAaGAaTcCTggcACCggAAggACCtATTGTTaTAAAGtTTGAGTCaAGAGATGGacatGcaAGTGGAGCCTaACTTTtCctAAGGAaTTCTGcTTTGTTCTTCAAaGAAGTTGTtTtcCAGAtaAcCAGAGACCTgtATTgACTcTtTGAATA

>CYP3A89_human

GCTGAAAGGAAgACTCAGAGGAGAGAgataagtAAGGAAAGTaGtgATGGctCTcATCCCAgaCTTggCCATGGAAACCTGGcTTCTCCTGGCTgtCAGCCTGGTGCTCCTCTATCTATAtGGaACCcAttCaCATGGACTTTTTAAGAAGCTtGGAATTCCaGGGCCcACACCTCTGCCTTTTTTgGGAAATaTTtTGtccTACCATAAGGtTATgATGGAcAgCCTGTGCTGGCTATCACAGATCCtGACATGATCAAAACAGTgCTAGTGAAAGAATGtTATTCTGTCTTcACAAACCGGaGGCCTTTTGGTCCAgTgGGATTTATGAAAAGTGCCATCTCTaTagCTGAGGATGAAgAATGGAAGAGAtTACGAtCATTGCTGTCTCCAACCTTCACcAGTGGAAAaCTCAAGGAGATGgTCCCtATCaTTGcCCAGTATGGAGAtGTGTTGGTgAGaAAtCTGAgGcgGGAAGCAGAGAcAGGCAAGCCtgTCACCTTGAAAGACATCTTTGGGGCCTACAGCATGGATGTGATcACTAGCACATCATTTGGAGTGAACATCGACTCtCTCAACAATCCACAAGAcCCCTTTGTGGAAAAcACCAAGAAGCTtTTaAGaTTTGATTTttTgGATCCATTctTTCTCTCAATAACACTCTTTCCATTcCTcAtccCAaTTcTTGAAGTATTAAATaTCTgTGTGTTTCCAAgAgaaGTTACaaATTTTTTaAgAAAATCTGTAAAAAGGATGAAAGAAAGTCGCCTCgAAGATAcAcAAAAGCACCGAGTgGATTTcCTTCAGCTGATGATTgACTCTCAGAATTCAAAAGAAactGAgtCCCAcAAAGCTCTGTCcGATCTGGAGCTCGTGGCCCAATCaATTATCTTTATTTTTGCTGGCTATGAaACCACgAGCAGTgtTCTcTCCTTCaTTATGTATgaacTGGCCACTCACCCTGATGTCCAGCAGAAaCTGCAGGAGGAaATTGATGCagtTTTaCCCAATAAGGCaCCaCCCACCTATGATaCtgTGcTACAGATGGAGTATCTTGACATGGTGgTGAATGAAaCgCTCAGaTTATTCCCAATTGCTaTgAGACTTGAGaGGGTCTGcAAaAAAGATGTtGAgATCAATGGGaTGTTCATTCCcAAAGGGgtgGTGGTGATGaTtCCAAgCTaTGCTCTTCACcgtgACCCAaAGTaCTGGaCAGAGCCTGAGaAGTTCCtcCCTGAAAGGTTCAGcAAGaAGAACAAGGACAaCATAgATCCTTAcATATACacaCCCTTTGGAagTGGACCcaGAAACTGCATTGGCATGAGGTTtGCTCTcATGAACATGAAACTTGCTCTaaTCAGAgTcCTtCAGAACTTCTCCTTCAAACCTTGTAAAGAAACACAGATCCCCCTGAAATTAaGCttaggAGGACTTCTTCAACCAgAAAAACCCgTTGTTCTAAAGGTTGAGTCaAGgGATGGcACcGTaAGTGGAGCCTGAaTTTtCctAAGGACTTCTGcTTTGcTCTTCAAGaAAtcTGTgcCTgAGAACAcCAGAGACCTCAAaTTACTTTgTGAATAgAACtCtGAATGAAGATGGGCTTGAgcTcTCTCAtTGtCTgTGTAGAGTgTTATAcATATAAAGGAGGTCCACCACCCCCAGTTAGcACCATtaAcTccTCCTGAGCtCTGATAAGATTTCTCAAtAATTTccTCcACAAAtTaTtAatgAAAAtAAgaATTaTttTG

>CYP3A89_sheep

AAGaAAAGTGGCCATGGAgCTaATCCCAAGCTTTTCCgTGGAAACCTGGGTTCTCCTGGCTACCgGCCTGGTGCTCCTCTATCTATAtGGGACtTAttCatATGGACTTTTTAAGAAGCTgGGgATTCCTGGGCCAAgACCTgTGCCcTaTTTTGGAAgTaTTCTGgccTACCgcAAGGGTATTccaGAgTTTGAcAAtcAgTGTTTTaAAAAGTATGGgAAAATGTGGGGGACTTATgAaGGtcgAcAACCTaTGtTGatTATCACAGATCCAGAtATaATCAAAACAGTACTAGTGAAAGAgTGtTATTCTGTCTTcACAAACCGGaGGTTTTGGTCCAaTgGGAaTcATGAAAAaTGCCATCTCTCTGgCTtgGGATGAACAATGGAAGAGAATACGgACATTGCTGTCTCCAgCCTTCACcAGTGGAAAGCTCAAGGAGATGcTCCCCATCgTTGGCCAGTATGcAGAtGTGTTGGTgAGGAACCTGAgGAAGGAAGCAGAGAAAGGCAAtCCtgTCAaCaTGAAAGCATgTTTGGGGCCTACAGCATGGATGTGATcACTgGCACAgCATTTGGAGTGAAtATtGAtTCCCTCAACAAcCCACAcGATCCCTTTGTGGAAtAcAgCAAGAAcCTCTTaAaaTTTagaccCtTcGATCCATTcaTTCTtTCAATAACAtTaTTTCCgTTTCTcAAccCAGTTTTTGAAcTATTAAAcaTtacTaTaTTTCCAAAAAGTacTgtgGAcTTTTTCAcAAAATCTGTAAAgAaGATtAAAGAAAGTCGCCTCAcAGATAAAcAAAAGCACCGAGTgGATTTaCTTCAGCTaATGATTAACTCTCAGAATTCcAAAGAAaTtGACACCCATAAAGCTCTGTCTGATgTaGAaCTCGTGGCCCAAaCTAcTATCTTTATTTTTGgTGGCTATGAGACCACTAGCAcTTCTCTTTCCTTCaTTATaTATgaaTTGGCCACTCACCCTGATGTCCAGCAGAAGCTGCAGGAGGAGATTGATGCGACTTTCCCCAATAAGGCcCCTCCCACCTATGATGCCCTGGTACAGATGGAGTATCTTGACATGGTGgTGAATGAAaCtCTgAGaaTgTTtCCAATTGCcGggAGACTTGAGaGGcTCTGTAAGAAgGATGTGGAAATCcgcGGGGTGTcCATTCCcAAAGGGACAacaGTGATGGTGCCgctCTTcGtgCTTCACAAcaACCCAGAGTTCTGGCCAGAGCCTGAGGAGTTCCGTCCcGAAAGGTTCAGTAAGaAGAACAAGGACAGCATAAATCCTTAcgTcTACCTGCCtTTTGGAaCTGGACCcCGAAACTGCcTTGGCATGAGGTTCGCTtTaATGAACATaAAACTcGCTCTcGTCAGAATcCTtCAGAACTTCTCCTTCAtACCTTGTAAAGAAACACAGgTCaTGGGACcacaAGTGGAGCCTGACTTTCtctAAGGAtTTtcacTTTGgTtTggAAGGAAGcTGcATCTCAGAgtAcCAGAGAATAAAACCAATGAAGATGGGTTCTCAtAGGAcacGCTCCATCCACCCCAGTTAGTACCATCTAcTccTCCTGgGCACTGgTCATTCTCAACAATcTTATCgACAgACTTTAATGAAAAtAAgTaAAaCATTATAtTGAGCAAGaCAATAAgcAtCTgTTTACAA

>CYP3A89_mouse

AAaGAAAacaGCaATGGACCTGATCCCAAaCTTTTCCATGGAAACCTGGaTgCTCCTGGCTACCAGCCTGGTcCTtCTCTATagATAtGGaACtcAttCaCATGGAaTTTTTAAaAAGtTgGGAATTCCTGGGCCAAaACCTCTGCCTTTcTTgGGgAcgaTTCTtgcTTACCAgAAGGGctTcTGGGAaTgTGAcAtacAATGTcaTaAAAAaTATGGgAAAATGTGGGGGACTTATgAcGGtcgAcAgCCTGTGCTGGCTATCACAGATCCAGACATaATCAAAACAGTgCTgGTGAAgGAATGtTAcTCTacCTTcACAAACCGGCGgCgcTTTGGTCCAgTgGGtaTTtTGAAAAagGCCATCTCTaTcTCTGAGaATGAAgAATGGAAGAGAATcCGAgCccTGCTGTCTCCAACCTTCACcAGTGGgAgGCTCAAGGAGATGTTCCCCATCCATCTTTGGGGCCTACAGCATGGATGTGATcACagcCACcTCATTTGGAGTGAAtATtGAtTCCCTCAACAAcCCACAgGATCCtTTTGTGGAgAAaAtCAAGAAGCTCTTaAaaTTTGATaTCtTTGATCCAcTgtTcCTCTCAgTgACACTCTTTCCATTcCTTaaGAGTgGATTTTCTTCAGCTGATGATaAACTCcCAGAATTacAAAGCatTaTCTGATgTGGAGaTtGTGGCtCAgTCagTTATCTTTATTTTTGCTGGCTATGAGACCACaAGCAGTgCTCTTTCCTTtgcatTGTATTTgcTGGCCAtTCACCCTGATGTaCAGaAGAAaCTtCAGGAtGAaATTGATGCGgCTcTgCCCAATAAGGCaCCTgCCACCTATGATaCCCTGcTACAGATGGAGTATCTaGACATGGTGgTGAATGAAaCtCTCAGaTTATatCCAATcGCTGgaAGACTTGAGaGGGTCTGTAAGAcAGATGTtGAAATCAATGGGcTaTTCATTCCcAAAGGGACtGTGGTGATGaTaCCAACCTTTGCTCTTCACAAAgACCCgaAGTaCTGGCCAGAGCCTGAGGAaTTCCGcCCTGAAAGGTTCAGcAAGaAGAAtcAGGACAGCATcAATCCTTAcATgTACCTGCCCTTTGGgagTGGACCaaGgAACTGCATTGGCATGAGGTTtGCTCTcATaAACATGAAAgTTGCTCTTGTCAGAgTcCTGCAGAACTTCaCtgTCcAgCCTTGTAAgGAAACtCAGATCCCttTaAAATTAaGCAAaCAAGGACTTCTTCAACCAgAAAAcCCacTccTTCTAAAaGTTGtGTCCAGAGATGaGACtGTaAGT

>CYP3A93_horse

ATGGACCTGATCCCAAcCTTTTCCAtGGAAACCTGGGTTCTCCTGGCTACCAGCCTAGTGCTCgTcTATCTATATGGGACCTATACaCATGGACTTTTTAAGAAGCTGGGAATTCCTGGGCCAACACCTCTGCCTTTTTTTGGAAATGTTCTGTCCTACCGTAAGGGTATTTcGAATTTTGACAGGAAATGTTTTAAAAAGTATGGGAAAATGTGGGGGGTTTATGATGGTAGAAGGCCTGTGTTGGCTATCACAGACCCAGACATGATCAAAACAGTACTAGTGAAAGAATGTTATTCTGTCTTCACAAACCGGCGGCCTTTTCGTCCAGTGGGATTTATGAAAAGCGCCATCTCTCTGTCTGAGGATGAGGAATGGAAGAGAATACGAACGTTGCTGTCTCCAACCTTCACCAGTGGAAAGCTCAAGGAGATGTTCCCAATCATTGGCCAGTATGGAGATGTGTTGGTGAGGAACCTGAGGAAGGAAGCAGAGAAAGGCAAACCCGTCACCTTGAAAGACATCTTTGGGGCCTACAGCATGGATGTGATTACTAGCACATCATTTGGAGTAAACATCGATTCCCTCAACAACCCGCAGGATCCCTTTGTGGAAAATAGCAACAAGCTCTTAAGATTTGATTTCCTCAATCCACTCATTCTTTTAATGGTACTCTTTCCGTTTCTTCAGCCAATTTTTGAAGTATTAAATATCTCTCTGTTTCCAAAAAGTGCTATTGATTTTTTCaCAAAATCTGTAAAAAGGATGAAAGAGAGTCGCCTCAAAGATAAAGACAAGCACCGAGTTGATTTTCTTCAGCTGATGATTAACTCTCAGAATTCCAAAGAACTGGACACCCATAAAGGTCTGTCTGATCTGGAGCTCGTGGCCCAATCTATTGTCTTTATTTTTGCTGGCTATGAGACCACAAGCACTTCTCTTTCCTTCCTTTTGTATCTTTTGGCCACTCACCCTGATGTCCAGCAGAAGCTGCAGGAGGAGATTGATGCAATTTTCCCGAATAAGGCACCTCCCACCTATGATGCCCTGGTACAGATGGACTATCTTGACATGGTGTTGAATGAATCTCTCAGATTATTCCCAGTTGCTGTTAGACTTGAGAGAGTCTGTAAGAAAGATGCGGAAATCAATGGAGTGTTCATTCCCAAAGGGACAGTGGTGATGGTGCCAACCTTTAGTCTTCACCGAGCCTCAGAGTTTTGGCCTGAGCCCGAGGAGTTCCGTCCTGAAAGATTCAGTAAGAAGAACAAGGACAACATAAATCCTTGTATATACATGCCTTTTGGAAACGGACCCCGAAATTGCATCGGCATGAGGTTTGCTATGGTGAATATGAAACTTGCTCTTGTCAGAGTGCTGCAGAACTTCTCCTTCAAACCTTGTAAAGAAACACAGATTCCCCTGAAATTAGGCAATCAAGGACTTCTTCAACCGCAAAAACCCATTGTTCTAAAGGTTGAGTCCAGAGATGGGACCGTCAATGGAGCCTGA

>CYP3A93_rhino

ATGGACCTGATCCCAAaCTTTTCCgTGGAAACaTGGGTTCTCCTGGCTACCAGCCTgGTGCTCcTCTATCTATATGGGACCTATACtCATGGACTTTTTAAGAAaCTaGGAATTCCTGGGCCcACACCTCTGCCTTTTTTTGGAAcTGTTCTGggCTACCGTAAGGGcATTTgGgAgTTTGAtAaGAAATGTcTgAAAAAGTATGGaAAAATGTGGGGGtTTTATGATGGTAaAcaGCCTGTGTTGGCTATCACAGACCCAGACATGATCAAAACAGTACTAGTGAAAGAATGcTATaaTGcCTTCACAAACCGGCGGgCTTTTgGTCCAaTGGGATTTATGAAAAaaGCCATCTCTCTGTCTGAGGATGAacAATGGAAGAGAATACGAAatTTGCTGTCTCCAACCTTCACCAGTGGAAAGCTCAAGGAGATGTTCCCcATCATTGGCCAGTATGGAaATaTGTTGGTGAaGAACCTGAGGAAGGAAGCAGAGAAAGGCAAgCCCGTtACCTTGAAAGACATCTTgGGGGCCTACAGCATGGATGTGATTACTAGCACATCATTTGGAGTgAACATCGATTCCCTCAACAACCCaCAaGATCCCTTTGTGGAAAActGCAAgAAGCTCTTAAGATTTGATTTCCTtgATCCAtTCATTCTcTcAATGTgCTtTTTCCaTTTCTTaAtCCAgTTTTTGAAGcATTAAATATCTtTCTGTTTCCAAAAAGTGtTAcTGATTTTTTtgCAAAATCTGTAcAAAGGATaAAAGAaAGTCGCCTCAAAGATAAAGAaAAGCAgCGAGTTGATTTTCTTCAGCTGATGATTAACTCcCAGAATTCCAAAGAACTGGACACCCAcAAAGGTCTGTCTGATCTGGAGCTCaTaGCCCAATCTATTaTCTTTATTTTTGCTGGCTATGAGcCCAttAGCACTTCTCTTTCCTTCCTTaTaTATtTTTTGGCCACTCACCCTGATGTCCAGCAGAAGCTGCAGGAGGAGATTGATGCAAcTTTCCCcgATAAGGCACCTgtCACtTATGATGCCCTGaTACAGATGGAgTATCTTGACATGGTGTTGAATGAATCTCTCAGATTATTCCCAaTTGCTGgTAGACTTGAGAGgGTCTGTAAGAAAGATttGGAAcTCggTGGgGTGTTCATTCCCAAAGGGACAGTaGTGATGGTGCCAACCTTTtcTCTTCACCGAGaacCAGAGTTcTGGCCTGAGCCtGAGGAGTTCCaTCCTGAAAGgTTCAGTAAGgAcAACAAGGACAgtATAAATCCTTaTATATACATGCCgTTTGGAAgtGGACCCCGAAAcTGCATtGGCATGAGaTTTGCTgTcGTGAAcATGAAACTTGCTgTTGTCAGAcTGCTGCAGAACTTCTCCTTCAAACCTTGcAAAGAAACtCAGATTCCCCTGAAATTAGGCAcTCAAGGACTcaTTCAACCGgAAAAACCaATTGTTCTAAAaGTTGAGTCgAGAGcTGGGACtGTgAATGGAGCCTGA

>CYP3A93_cow

ATGGAgCTaATCCCAAgCTTTTCCATGGAAACCTGGGTTCTCCTGtCTAtCAGCCTgGTGCTCcTtTATCTATATGGaACtTATtCACAcGGACTaTTTAAGAAGCTGGGAgTTCCTGGGCCAAgACCTCTGCCTTaTTTTGGAAATGTTCTGTCCTACCGaAAGGGTgTTTgtgAgTTTGAtgaagAATGTTTTAAAAAGTATGGGAAAATGTGGGGGaTTTtTGAaGGTAaAcatCCTcTGTTGGtTATCACgGAtCCAGACATGATCAAAACAGTACTAGTGAAAGAATGTTATTCTGTCTTCACAAACCGgaGGgTTTTgGTCCAaTGGGAgTTATGAAAAatGCtgTtTCTgTGgCTGAGGATGAacAATGGAAGAGAATACGgACaTTGCTGTCTCCAACCTTCACCAGTGGgAAGCTCAAGGAGATGTTCCCtATCATTGGgaAGTATGGAGATGTGTTGGTGAGGAACCTGAGGAAGGAAGCAGAGAAAGGCAcgtCCGTCgaCaTGAAAGATCTTTGGGGCCTACAGCATGGATGTGATTACTAGCACATCATTTGGAGTgAAtATtGATTCCCTtggCAACCCaCAaGATCCCTTTGTGGAgAATgcCAAgAAGCTCTTAAGATTTGATaTCCTtgATCCAtGTACTCTTTCCaTTcCTTatcCCAATcTTTGAAGTATTAAATATCagcaTaTTTCCAAAAAGTGCTgTgaATTTTTTgACAAcATCcGTAAAAAaGATaAAAGAaAGTCGCCTCAAAGATACGtGTgGAcTTTCTTCAGCTGATGATTAACTCcCAGAATTCCAAAGAAacaGACAatCATAAAGGTCTcTCTGAcCaaGAaCTCaTGGCCCAgagTATTaTCTTTATTTTTGgTGGCTATGAGACCACtAGCACTTCTCTTTCCTTCaTTaTaTATgaaTTGGCCACTCACCCTGATGTCCAGCAGAAGCTGCAGGAGGAGATTGATGCgAcTTTCCCcAATAAGGCgCCTCCgACCTATGATGtCCTGGcACAGATGGAgTATCTTGACATGGTGgTGAATGAgaCTCTCAGAaTgTTtCCtaTTGCTGTTAGACTTGAGAGgtTCTGTAAGAAgGATGtGGAAATCcATGGgGTGTcCATTCCCAAAGGGACAacGGTGAcGGTGCCAAtCTcTgtgCTTCACaGAGaCcCAcAGcTcTGGCCaGAGCCtGAGGAGTTCCGTCCTGAAAGgTTCAGTAAGAAGAACAAGGACAgCATAAATCCTTacgTcTACcTGCCTTTTGGAActGGACCCCGAAATTGCATtGGCATGAGGTTTGCTATcaTGAAcATGAAACTTGCTgTTGTCAGAGTcCTGCAGAACTTCTCCTTCAAACCTTGTAAAGAAACACAGATcCCCtTGAAAaTAaaaAgTCAAGGACTTtTaagACCGgAAAAACCCATTGTTCTgAAGGTTGtGctCAGAGATGaGACCaTaAgTGGAGCtTGA

>CYP3A93_dog

ATGGACCTaATCCCAAgCTTTTCCATGGAAACCTGGcTTCTCCTGGCTACCAGCCTgGTGCTCcTCTATCTgTATGGGACCTAcACACATGGggTTTTTAAGAAGCTaGGAATTCCTGGaCCAACACCTCTGCCTTTTgTgGGAAcTGcTCTGggCTACCGTAAgGGtTTTATGATGGgcGAcaGCCTGTGcTGGCTATCACAGAtCCgGACATGATCAAAACAGTgCTAGTGAAAGAATGTTATTCTGTCTTCACAAACCGGCGGgCTTTTgGTCCAGTGGGATTTATGAAAAGtGCCATCTCTCTGTCTGAGGATGAaGAATGGAAGAGAATACGAACaTTGCTGTCcCCAACCTTCACCAGTGGAAAGCTCAAGGAGATGTTCCCcATCATTGGCCAGTATGGAGATGTGTTGGTGAGGAACCTGAGGAAGGAgGCAGAGAAAGGCAAAtCCaTCAaCTTGAAAGACATCTTTGGaGCCTACAGCATGGATGTGATTACcAGCACATCATTTGGAGTgAACATtGATTCCCTCAACAACCCaCAaGATCCCTTTGTGGAAAATAtCAAgAAGCTCTTAAaATTTGATTTCCTtgATCCAtGTACTgTTTCCaTTcCTTaccCCAgTTTTTGAAGTATTAAATATCTggCTcTTTCCAAAAAGTGtTAcTGATTTTTTCACAAAATCTGTAAAAAGaATGAAgGAaAaTCGCCTCAAAGATAAAcAaAAGCAtCGAGTgGAcTTTCTTCAGCTGATGATTAACTCcCAGAATTCtAAAGAAacaGACACtCATAAAGGTCTaTCTGATtTGGAGCTgGTGGCCCAATCTATTaTCTTTATTTTTGCTGGCTATGAGACCACtAGCACTTCTCTTTCCTTCCTTaTGTATgaaTTGGCCACTCACCCTGATGTCCAGCAGAAaCTGCAGGAGGAGATTGATGCgAcTTTCCCcAATAAGGCAttgCCCACtTAcGATGCCCTtGTgCAGATGGAaTATCTgGACATGGTGTTGAATGAAaCTCTCcGATTATaCCCAaTcGCTGgTAGACTTGAGAGgGTCTGTAAGAAAGATGtGGAAATCAgTGGtGTGTTCATTCCCAAAGGGACAGTGGTGATGGTGCCAACCTTTAcTCTTCAtCGAGaCcagagtcTcTGGCCaGAGCCtGAGGAaTTCCGaCCTGAAAGgTTCAGTAgGAAGAACAAGGACAgCATAAATCCTTaTAcATACcTGCCTTTTGGAActGGACCCCGAAAcTGCATtGGaATGAGGTTTGCgATcaTGAAcATGAAACTTGCcCTTGTtAGgGTcCTGCAGAACTTCTCCTTCAAACCTTGTAAAGAAACACAGATcCCCCTGAAATTAaatgcTCAAGGgaTTaTTCAACCtgAAAAgCCCATTGTTCTcAAGGTTGAGcCaAGAGATGGGAgtGTaAATGGAGCCTGA

>CYP3A93_pig

ATGGACCTGATCCCAAgCTTTTCtgTGGAAACCTGGcTgCTtCTGGCTACCAGCCTgGTGCTCcTCTATCTATATGGGACCTATtCACAcGGACTTTTTAAGAAGCTGGGgATTCCcGGGCCgAaACCTCTGCCTTaTTTTGGAAgTGTTCTagaaTACCGTAAGGATTTTGACAatAAATGTTTTAAAAAGTATGGGAAAATGTGGGGTTTATGAcGGTAGAcaGCCTGTGcTGGCTATgAgAGACCCAGAaATGATCAAAgCAGTgCTtGTGAAAGAATGTTAcTCgGctTTCACAAAtCGGCGGTTTTgGTCCAGcGGGAgTTATGAAAAatGCCATCTCTgTGgCTaAGGATGAGcAATGGAAGcGAgTACGAACaTTGCTGTCTCCAACCTTCACCAGTGGAAAGCTCAAaGAGATGTTCCCcATCATTGcCCAGTATGGAGATaTGcTGGTGAGGAACCTGAGGAAGGAAGCAGAGAAAGGCAAgATCTTTGGGGCCTACAGCATGGAcGTGATcACaAGCACAgCATTTGGAGTgAACTTGATTTCCTCgATCCAtTtATTCGTAtTaTTTCCaTTcCTcagcCCAtTcTTTGAAGTATTAgAcATCaCTtTGTTTCCAAgAAGTtCTgTgaAaTTTTTCACAcAgTCTGTAAAAAGGATGAAAGAaAGTCGCCTCAAAGATcCGAGTgGAccTTCTTCAGCTGATGATTAACTCcCAGAATTCCAAAGAAacGGAtgCCCATAAAGGTCTGTCTGATCaaGAaCTtGTGGCCCAAagTATTaTCTTcATTTTTGCcGGCTATGAGACCACtAGCAgTTCTCTcTCCTTCCTggcGTATaTacTGGCCACTCACCCTGAcGTCCAGCAGAAGCTGCAGGAGGAGATTGATGCgAccTTCCCcAgcAAGGCg

>CYP3A93_elephant

ATGGACCTGATCCCAAgCcTTTCCAcaGAgACtTGGaTTCTCCTGGCcACCAGCCTgGTGCTCcTCTAcCTATATGGaACCTATtCACATaatgTTcTaAAGAAGCTcGGgATTCCTGGGCCcAagCCTCTGCCTTTTgTgGGAgcTcTTCTGgCCTACCGcAAGGGTtTgTgGgATTTTGACAtGAAATGTTcTAAAAAGTATGGaAAAATaTGGGGGtTTTATcATGGTctAcaGCCTGTGaTaGCcATCACAGAtCCtGgCATGATCAAgACAaTAaTgGTGAAAGAATGTTATTCcacCTTCACAAACCGGaGGgTTTTgtTCCAaTGGGATTTATGAAAtctGCCATtTCctTGTCTaAGGATGAaGAATGGAgGAGAgTACGAACGTTGCTGTCTCCAACCTTCACCAGTGGAAAaCTCAAGGAGATGcTCCCcATCATcGGCCAGTATGGAGAaGTaTTGtTGAaGcAtCTGAGagAGGAAGCAGAaAAAGGCAAgCCtGTCACaTTGAAgCATCTTcGGGGCtTACAGCATGGATGTGATcACaAGCACgTCATTTGGAGTgAACATCGATTCCCTCAACAACCCaCAaGATCCCTTTGTGcAAAAcAtCAggAAGCTCaTgAGATTTaATaTCtTCgAcCCAtTgATTtTTTCCaTTcCTTactCCAATTcTTGAAGcAcTAAgTATCTCTgTGTTTCCAAgAgcTGtTAcTGATTTTTTtACAAAATCTGTAAAAAcaATaAAAGAaAGcCGCCTtAAAGAcAAtaAaAAGCACCGAGTgGAcTTTCTTCAGCTGATGATcgAtTCcCAGAATTCCAAgGAAactatgtCCCATAAAGGTtTGaCTGATaTGGAGCTCGTaGCCCAATCaATTaTCTTTATTTTTGCTGGtTATGAGACCACtAGCACTaCTCTTTCCTTCCTTaTGTATtTaTTGGCCACcCACCCTGATaTtCAGCAGAAaCTGCAGaAGGAGATTGATGCggcTTTCCCcAATAAGGCAtCTCCCACaTATGATGtCaTGtTgCAGATGGAaTATCTTGACATGGTGgTGAATGAAaCaCTCAGATTATTCCCAaTTGtTGgcAGAaTTGAGAGgGTCTGcAAGAAAGATGttGAAATCAgTGGAGTGacCATTCCCAAAGGGgCAGTGGcaATGGTGCCAgCCTTTgcTCTTCACCGAGaCcCAGAacacTGGCCaGAGCCtGAGaAGTTCatTCCTGAgAGgTTCAGTAAGgAGAACAAGGACAgCATAgATCCTTactTATACcTGCCcTTTGGAAttGGACCCCGAAATTGCATCGGtATGAGGTTTGCTcTcaTGAAcATGAAACTTGCTaTcaTCAGAGTtCTGCAGgAgTTCTCCgTCAAACCTTGTAAAGAAACACAGg

>CYP3A93_human

TCCCAgaCTTggCCATGGAAACCTGGcTTCTCCTGGCTgtCAGCCTgGTGCTCcTCTATCTATATGGaACCcATtCACATGGACTTTTTAAGAAGCTtGGAATTCCaGGGCCcACACCTCTGCCTTTTTTgGGAAATaTTtTGTCCTACCaTAAGGtTTTATGATGGTcaAcaGCCTGTGcTGGCTATCACAGAtCCtGACATGATCAAAACAGTgCTAGTGAAAGAATGTTATTCTGTCTTCACAAACCGGaGGCCTTTTgGTCCAGTGGGATTTATGAAAAGtGCCATCTCTaTagCTGAGGATGAaGAATGGAAGAGAtTACGAtCaTTGCTGTCTCCAACCTTCACCAGTGGAAAaCTCAAGGAGATGgTCCCtATCATTGcCCAGTATGGAGATGTGTTGGTGAGaAAtCTGAGGcgGGAAGCAGAGAcAGGCAAgCCtGTCACCTTGAAAGATCTTTGGGGCCTACAGCATGGATGTGATcACTAGCACATCATTTGGAGTgAACATCGAcTCtCTCAACAAtCCaCAaGAcCCCTTTGTGGAAAAcAcCAAgAAGCTtTTAAGATTTGATTTttTggATCCAtTCtTTCTcTcAATaGcAgTCTTTCCaTTcCTcatcCCAATTcTTGAAGTATTAAATATCTgTgTGTTTCCAAgAgaaGtTAcaaATTTTTTaAgAAAATCTGTAAAAAGGATGAAAGAaAGTCGCCTCgAAGATACACCGAGTgGATTTcCTTCAGCTGATGATTgACTCTCAGAATTCaAAAGAAactGAgtCCCAcAAAGGTCTGTCcGATCTGGAGCTCGTGGCCCAATCaATTaTCTTTATTTTTGCTGGCTATGAaACCACgAGCAgTgtTCTcTCCTTCaTTaTGTATgaacTGGCCACTCACCCTGATGTCCAGCAGAAaCTGCAGGAGGAaATTGATGCAgTTTTaCCcAATAAGGCACCaCCCACCTATGATaCtgTGcTACAGATGGAgTATCTTGACATGGTGgTGAATGAAaCgCTCAGATTATTCCCAaTTGCTaTgAGACTTGAGAGgGTCTGcAAaAAAGATGttGAgATCAATGGgaTGTTCATTCCCAAAGGGgtgGTGGTGATGaTtCCAAgCTaTgcTCTTCACCGtGaCcCAaAGTacTGGaCaGAGCCtGAGaAGTTCCtcCCTGAAAGATTCAGcAAGAAGAACAAGGACAACATAgATCCTTacATATACAcaCCcTTTGGAAgtGGACCCaGAAAcTGCATtGGCATGAGGTTTGCTcTcaTGAAcATGAAACTTGCTCTaaTCAGAGTcCTtCAGAACTTCTCCTTCAAACCTTGTAAAGAAACACAGATcCCCCTGAAATTAaGCttaggAGGACTTCTTCAACCagAAAAACCCgTTGTTCTAAAGGTTGAGTCaAGgGATGGcACCGTaAgTGGAGCCTGA

>CYP3A93_sheep

ATGGAgCTaATCCCAAgCTTTTCCcTGGAAACCTGGGTTCTCCTGGCTACCgGCCTgGTGCTCcTCTATCTATATGGaACtTATtCACATGGACTgTTTAAGAAGCTGGGAgTTtCTGGGCCAAgACCTCTGCCTTaTTTTGGAAATGTTCTGTCCTACCGaAAGGGTgTTTgtgAaTTTGAtgaagAATGTTTTAAAAAGTATGGGAAAATGTGGGGGaTTTtTGAaGGTAaAcatCCTcTtTTGGtTATCACAGAtCCAGACATGATCAAAACAGTACTAGTGAAAGAATGTTAcTCTGTCTTCACAAACCGGaGGTTTTgGTCCAaTGGGAaTTATGAAAAatGCtgTtTCTgTGgCTGAGGATGAacAATGGAAGAGAATACGgACaTTGCTGTCTCCAACCTTCACCAGTGGgAAGCTCAAaGAGATGTTtCCtATCATTGGgaAGTATGGAGAcGTGTTGGTGAGGAACCTGAGGAAGGAAGCAGAGAAAGGCAcgtCCGTCAaCaTGAAAGACATCTTTGGGGCCTACAGCATGGATGTGATTACTAGCACATCATTTGGAGTgAAtATtGATTCCCTtggCAACCCaCAaGATCCCTTTGTGGAgAATgcCAAgAAGCTCTTAAGATTTaATaTCCTCgATCCAtTtcTTCGTACTCTTTCCaTTcCTTgtcCCAATcTTTGAAGTATTAAATATCaCcaTGTTTCCcAAAAGTGCTgTgGATTTTTTgACAAAATCTGTAAAAAGGATaAAAGAaAGTCGCCTCAAAGATAACGtGTgGAcTTTCTTCAGtTGATGATTAACTCTCAGAATTCCAAAGAAacaGACAaCCATAAAGGTCTcTCTGAcCaaGAaCTCaTGGCCCAAagTgTTaTCTTTATTTTTGCcGGCTATGAGACCACtAGCAaTaCgCTTTCCTTCCTTTTGTATaTTTTGGCCACTCACCCTGATGTCCAGCAGAAGCTGCAGGAGGAGATTGATGCgAcTTTCCCcAATAAGGCACCTCCgACCTAcGATGtCCTGGcACAGATGGAgTATCTTGACATGGTGgTGAATGAgaCTCTCAGAaTgTTtCCAaTTGCTGTTAGACTTGAtAGgcTCTGTAAGAAgGATGtGGAAATCcAcGGgGTGTcCATTCCCAAAGGGACAGcGGTGAcGGTGCCAAtCTTTgtgCTTCACaGAGaCcCAcAGcTcTGGCCaGAGCCtGAGGAGTTtCGTCCTGAAAGgTTCAGTAAGAAGAACAAGGACAgCATAAATCCTTaTgTATACcTGCCTTTTGGAActGGACCCCGAAATTGCATtGGCATGAGGTTTGCcATcaTGAAcATGAAACTTGCTaTTGTCAGAGTcCTGCAGAACTTCTCCTTCAAACCTTGTAAAGAAACACAGg

>CYP3A93_mouse

ATGGACCTGATCCCAAaCTTTTCCATGGAAACCTGGaTgCTCCTGGCTACCAGCCTgGTcCTtcTCTATagATATGGaACtcATtCACATGGAaTTTTTAAaAAGtTGGGAATTCCTGGGCCAAaACCTCTGCCTTTcTTgGGgAcgaTTCTtgCtTACCagAAGACAtacAATGTcaTAAAAAaTATGGGAAAATGTGGGGGtTATGAcGGTcGAcaGCCTGTGcTGGCTATCACAGAtCCAGACATaATCAAAACAGTgCTgGTGAAgGAATGTTAcTCTacCTTCACAAACCGGCGGCCtTTTgGTCCAGTGGGtaTTtTGAAAAagGCCATCTCTaTcTCTGAGaATGAaGAATGGAAGAGAATcCGAgCccTGCTGTCTCCAACCTTCACCAGTGGgAgGCTCAAGGAGgCATCTTTGGGGCCTACAGCATGGATGTGATcACagcCACcTCATTTGGAGTgAAtATtGATTCCCTCAACAACCCaCAGGATCCtTTTGTGGAgAAaAtCAAgAAGCTCTTAAaATTTGATaTCtTtgATCCACTgtTcCTcaGAGTgGATTTTCTTCAGCTGATGATaAACTCcCAGAATTaCAAAaccaaaGAatCtCATAAAGGTtTaTCTGATgTGGAGaTtGTGGCtCAgTCagTTaTCTTTATTTTTGCTGGCTATGAGACCACAAGCAgTgCTCTTTCCTTtgcaTTGTATtTgcTGGCCAtTCACCCTGATGTaCAGaAGAAaCTtCAGGAtGAaATTGATGCggcTcTgCCcAATAAGGCACCTgCCACCTATGATaCCCTGcTACAGATGGAgTATCTaGACATGGTGgTGAATGAAaCTCTCAGATTATatCCAaTcGCTGgaAGACTTGAGAGgGTCTGTAAGAcAGATGttGAAATCAATGGgcTaTTCATTCCCAAAGGGACtGTGGTGATGaTaCCAACCTTTgcTCTTCACaaAGaCcCgaAGTacTGGCCaGAGCCtGAGGAaTTCCGcCCTGAAAGgTTCAGcAAGAAGAAtcAGGACAgCATcAATCCTTacATgTACcTGCCcTTTGGgAgtGGACCaaGgAAcTGCATtGGCATGAGGTTTGCTcTcaTaAAcATGAAAgTTGCTCTTGTCAGAGTcCTGCAGAACTTCaCtgTCcAgCCTTGTAAgGAAACtCAGATcCCttTaAAATTAaGCAAaCAAGGACTTCTTCAACCagAAAAcCCacTccTTCTAAAaGTTGtGTCCAGAGATGaGACtGTa

>CYP3A94_horse

ATGGACCTAATCCCAAaCTTTTCCATGGAAACCTGGGTTCTCCTGGCTACCAGCCTGGTGCTCCTTTATCTATATGGGACCTATACACATGGACTTTTTAAGAAGCTGGGAATTCCTGGGCCGACACCTCTACCTTTTTTGGGAACTGTTCTGGGCTACCTTAAGGGTTTCTGGGATTTTGACAAGAAATGTTTTAAAAAGTATGGAAGTATGTGGGGGATTTATGATGGCCCACAGCCTGTGTTGGCCATTACAGATCCAGATATGATCAAAACAGTACTAGTGAAAGAATGTTATTCTGTCTTCACAAACCGGCGGTCTCTTGGTCCAGTGGGATTTATGAAGAATGCCATCTCTCTGTCTGAGGATGAGCAATGGAAGAGAATAAGAACATTGCTGTCTCCAACCTTCACCAGTGGAAAGCTCAAGGAGATGTTCCCCATCATTGGCCAGTATGGAGATGTGTTGGTGAGGAACCTGAGGAAGGAAACAGAGAAAGGAAAACCTGTCACCTTGAAAAACATCTTTGGGGCCTACAGCATGGATGTGATTACTAGcACATCATTTGGAGTGAATATCGATTCCCTCAACAACCCACAAGATCCCTTTGTGGAAAATACCAAGAAGCTCTTAAGATTTAATTTCCTCGATCCATTCTTTCTCTCAATAACACTCTTTCCATTTCTTAAAGCAGTTTTTGAAGTAATACACATATATATGTTTCCAAAACGTGTTACTGATTTTTTCACAAAATCTGTAAAAAGGATGAAAGAAAGTCGCCTCAAAGATAAACAAAAGCACCGAGTGGATTTTCTTCAGCTGATGATTAACTCTCAGAATTCCAAAGAAATGGACACCCATAAAGCTCTGTCTGATCTGGAGCTTGTGGCCCAATCTATTATCTTTATTTTTGCTGGGTACGAGACCACTAGCACTTCTCTTTCTTTCCTTATGTATCTTTTGGCCACTCATCCTGATGTCCAGCAGAAGCTTCAGGATGAGATTGATGTGACTTTCCCAAATAAGGCCCTTCCCACCTATGATACCCTGTTACAGATGGAGTATCTTGACATGGTGCTGAATGAATCTCTCAGATTGTTCCCAATTGCTGGTAGACTTGAGAGGGTCTGTAAGAAAGATGTGGAAATCAATGGAGTGCTAATTCCCAAAGGGACAGTGGTGATGGTGCCAACCTTTATTCTTCATCGAGCCTCAGAGTTCTGGCCTGAGCCTGAGGAGTTCTGTCCTGAAAGGTTCAGTAAGAATAACAAGGACAACATAAATCCTTATATATACCTGCCCTTTGGAACTGGACCTCGAAACTGCATTGGCATGAGATTTGCTATCATGAACATGAAACTTGCTATCGTCAGAGTGCTGCAGAACTTCTCCTTCAAACCTTGTAAAGAAACACAGATCCCCCTGAAAATAGGCAATCAAGGACTTATTCAACCACAGAAACCCATTGTTCTAAAGGTTGAGTCCAGAGATGGTACCTGGAATGGAGCCTGA

>CYP3A94_rhino

ATGGACCTgATCCCAAgCTTTTCCAcaGAAACCTGGGTTCTCCTGGCTAtCAGCCTGGTGCTCCTcTATCTATATGGGACCTATtCACATGGACTTTTTAAGAAGCTGGGAATTCCTGGGCCGACACCTCTgCCaTTTTTtGGAACaGTTCTGGGCTACCTTAAGGGTTTtTGGGATTTTGACAAGAAATGTTTTAAAAAaTATGGAAaTATGTGGGGGtTTTATGATGGCCCACAGCCTGTaTTGGCtATcACAGATCCAGAcATGATCAAAACAGTACTAGTGAAAGAATGTTAcTCTGTCTTCACAAACCGGCGGgCTCTTGGTCCAaTGGGATTTATGAAGAATGCCATCTCTaTGTCTGAGGATGAaCAATGGAAGAGAATAcGAAtgTTGCTGTCTCCAACCTTtACCAGTGGAAAGCTaAAGGAGATGTTCCCCATCATTGGCCgaTATGGAGATGTGTTGGTGAGGAACCTGAGaAAGGAAgCAGAGAAAGGcAggCCTGTCACtTTGAAACATCTTTGGGGCCTAtAGCATGGATGTGATTACTAGCACATCATTTGGAGTGAAcgTCGATTCCCTCAACAACCCACAAGATCCCTTTGTGGAAAATACCAAGAAGCTCTTAAaATTTgATTTCCTCGATCCATTCTTTCTCTCAATAACACTCTTTCCATTTCTTAgccCAGTTTTTGAAGTAtTAaAtATcTtTcTcTTTCCAAAAaGTGTTACTGATTTTTTCACAAAATCTGTAAAAAGGATGAAAGAAAGTCGCCTCAAAGATAAACAAAAGACCGAGTGGATTTTCTTCAGCTGATGATTAACTCcCAGAATTCCAAAGAAATGGACACCCATAAAGCTCTGTCTGATCTGGAGCTgGTGGCCCAATCTATTATCTTTATTTTTGCTGGcTACGAGACCACaAGCACTTCTCTTTCcTTCCTTATGTATCTcTTGGCtACTCAcCCTGATGTCCAaCAGAAGtTgCAGGAgGAGATTGATGctACTTTCCCcAATAAGGCCCcaCCCACaTATGATACCCTGTTACAGATGGAGTATCTTGACATGcTGtTGAATGAATCTCTCAGATTaTTCCCAATTGCTGGTAGACTTGAGAGGGTtTGTAAGAAcGATGTGGAAATCAATGGAGTGtTcATTCCtAAAGGcACAGTaGTGATGGTGCCAACaTTTATTCTTCAcCGAGCCTCAGAGTTCTGGCCaGAtCCTGAGGAGTTCcaTCCTGAAAGGTTCAGTAAGAAgAAtAAGGACAAtATAAATCCTTATATATACCTGCCCTTTGGAACTGGACCcCGAAACTGCATTGGCtTGAGgTTcGCTATCATGAACATGAAACTTGCTgTCGTCAaAaTGCTGCAGAACTTCTCCTTCAgACCTTGTAAAGAAACACAGATCCCCCTGAAAtTAGGCAcTCAAGGACTTATTCAACCACAGAAACCCATTGTTCTAAAGGTTGAGTCCAGAGATGGgACCgtGAgTGGAGCCTGA

>CYP3A94_cow

ATGGAgCTAATCCCAAgCTTTTCCATGGAAACCTGGGTTCTCCTGtCTAtCAGCCTGGTGCTCCTTTATCTATATGGaACtTATtCACAcGGACTaTTTAAGAAGCTGGGAgTTCCTGGGCCaAgACCTCTgCCTTaTTTtGGAAaTGTTCTGtcCTACCgaAAGGTTTGAtgAagAATGTTTTAAAAAGTATGGgAaaATGTGGGGGATTTtTGAaGGtaaACAtCCTcTGTTGGttATcACgGATCCAGAcATGATCAAAACAGTACTAGTGAAAGAATGTTATTCTGTCTTCACAAACCGgaGGgCTCTTGGTCCAaTGGGAgTTATGAAaAATGCtgTtTCTgTGgCTGAGGATGAaCAATGGAAGAGAATAcGgACATTGCTGTCTCCAACCTTCACCAGTGGgAAGCTCAAGGAGATGTTCCCtATCATTGGgaAGTATGGAGATGTGTTGGTGAGGAACCTGAGGAAGGAAgCAGAGAAAGGcTCTTTGGGGCCTACAGCATGGATGTGATTACTAGCACATCATTTGGAGTGAATATtGATTCCCTtggCAACCCACAAGATCCCTTTGTGGAgAATgCCAAGAAGCTCTTAAGATTTgATaTCCTtGATCCATTtcTaCTtTCAgTAggACTCTTTCCATTcCTTAtccCAaTcTTTGAAGTAtTAaAtATcagcATaTTTCCAAAAaGTGcTgtgaATTTTTTgACAAcATCcGTAAAAAaGATaAAAGAAAGTCGCCTCAAAGATActCAAAAGCACCGtGTGGAcTTTCTTCAGCTGATGATTAACTCcCAGAATTCCAAAGAAAcaGACAatCATAAAGCTCTcTCTGAcCaaGAaCTcaTGGCCCAgagTATTATCTTTATTTTTGgTGGcTAtGAGACCACTAGCACTTCTCTTTCcTTCaTTATaTATgaaTTGGCCACTCAcCCTGATGTCCAGCAGAAGCTgCAGGAgGAGATTGATGcGACTTTCCCcAATAAGGCgCcTCCgACCTATGATgtCCTGgcACAGATGGAGTATCTTGACATGGTGgTGAATGAgaCTCTCAGAaTGTTtCCtATTGCTGtTAGACTTGAGAGGtTCTGTAAGAAgGATGTGGAAATCcATGGgGTGtccATTCCCAAAGGGACAacGGTGAcGGTGCCAAtCTcTgTgCTTCAcaGAGaCcCAcAGcTCTGGCCaGAGCCTGAGGAGTTCcGTCCTGAAAGGTTCAGTAAGAAgAACAAGGACAgCATAAATCCTTAcgTcTACCTGCCtTTTGGAACTGGACCcCGAAAtTGCATTGGCATGAGgTTTGCTATCATGAACATGAAACTTGCTgTtGTCAGAGTcCTGCAGAACTTCTCCTTCAAACCTTGTAAAGAAACACAGATCCCCtTGAAAATAaaaAgTCAAGGACTTtTaagACCggAaAAACCCATTGTTCTgAAGGTTGtGctCAGAGATGagACCataAgTGGAGCtTGA

>CYP3A94_dog

ATGGACCTAATCCCAAgCTTTTCCATGGAAACCTGGcTTCTCCTGGCTACCAGCCTGGTGCTCCTcTATCTgTATGGGACCTAcACACATGGggTTTTTAAGAAGCTaGGAATTCCTGGaCCaACACCTCTgCCTTTTgTGGGAACTGcTCTGGGCTACCgTAAGGtGGtTTTATGATGGgCgACAGCCTGTGcTGGCtATcACAGATCCgGAcATGATCAAAACAGTgCTAGTGAAAGAATGTTATTCTGTCTTCACAAACCGGCGGTCTtTTGGTCCAGTGGGATTTATGAAaAgTGCCATCTCTCTGTCTGAGGATGAagAATGGAAGAGAATAcGAACATTGCTGTCcCCAACCTTCACCAGTGGAAAGCTCAAGGAGATGTTCCCCATCATTGGCCAGTATGGAGATGTGTTGGTGAGGAACCTGAGGAAGGAggCAGAGAAAGGcAAAtCcaTCAaCTTGAAAAgCATCTTTGGaGCCTACAGCATGGATGTGATTACcAGCACATCATTTGGAGTGAAcATtGATTCCCTCAACAACCCACAAGATCCCTTTGTGGAAAATAtCAAGAAGCTCTTAAaATTTgATTTCCTtGATCCATTtTTctTCTCAATAACTgTTTCCATTcCTTAcccCAGTTTTTGAAGTAtTAaAtATcTggcTcTTTCCAAAAaGTGTTACTGATTTTTTCACAAAATCTGTAAAAAGaATGAAgGAAAaTCGCCTCAAAGATAAACAAAAGCAtCGAGTGGAcTTTCTTCAGCTGATGATTAACTCcCAGAATTCtAAAGAAAcaGACACtCATAAAGCTCTaTCTGATtTGGAGCTgGTGGCCCAATCTATTATCTTTATTTTTGCTGGcTAtGAGACCACTAGCACTTCTCTTTCcTTCCTTATGTATgaaTTGGCCACTCAcCCTGATGTCCAGCAGAAaCTgCAGGAgGAGATTGATGcGACTTTCCCcAATAAGGCatTgCCCACtTAcGATgCCCTtgTgCAGATGGAaTATCTgGACATGGTGtTGAATGAAaCTCTCcGATTaTaCCCAATcGCTGGTAGACTTGAGAGGGTCTGTAAGAAAGATGTGGAAATCAgTGGtGTGtTcATTCCCAAAGGGACAGTGGTGATGGTGCCAACCTTTAcTCTTCATCGAGaCcagagtcTCTGGCCaGAGCCTGAGGAaTTCcGaCCTGAAAGGTTCAGTAgGAAgAACAAGGACAgCATAAATCCTTATAcATACCTGCCtTTTGGAACTGGACCcCGAAACTGCATTGGaATGAGgTTTGCgATCATGAACATGAAACTTGCccTtGTtAGgGTcCTGCAGAACTTCTCCTTCAAACCTTGTAAAGAAACACAGATCCCCCTGAAAtTAaatgcTCAAGGgaTTATTCAACCtgAaAAgCCCATTGTTCTcAAGGTTGAGcCaAGAGATGGgAgtgtaAATGGAGCCTGA

>CYP3A94_pig

ATGGACCTgATCCCAAgCTTTTCtgTGGAAACCTGGcTgCTtCTGGCTACCAGCCTGGTGCTCCTcTATCTATATGGGACCTATtCACAcGGACTTTTTAAGAAGCTGGGgATTCCcGGGCCGAaACCTCTgCCTTaTTTtGGAAgTGTTCTaGaaTACCgTAAGGATTTTGACAAtAAATGTTTTAAAAAGTATGGgAaaATGTGGGGGTATGAcGGtagACAGCCTGTGcTGGCtATgAgAGAcCCAGAaATGATCAAAgCAGTgCTtGTGAAAGAATGTTAcTCgGctTTCACAAAtCGGCGGTCTCTTGGTCCAGcGGGAgTTATGAAaAATGCCATCTCTgTGgCTaAGGATGAGCAATGGAAGcGAgTAcGAACATTGCTGTCTCCAACCTTCACCAGTGGAAAGCTCAAaGAGATGTTCCCCATCATTGcCCAGTATGGAGATaTGcTGGTGAGGAACCTGAGGAAGGAAgCAGAGAAAGGcAAgATCTTTGGGGCCTACAGCATGGAcGTGATcACaAGCACAgCATTTGGAGTGAAcATTTgATTTCCTCGATCCATTtaTTCTCTCAATAATTGAAGTAtTAgACATcacTtTGTTTCCAAgAaGTtcTgtgaAaTTTTTCACAcAgTCTGTAAAAAGGATGAAAGAAAGTCGCCTCAAAGATcAACAAACGAGTGGAccTTCTTCAGCTGATGATTAACTCcCAGAATTCCAAAGAAAcGGAtgCCCATAAAGCTCTGTCTGATCaaGAaCTTGTGGCCCAAagTATTATCTTcATTTTTGCcGGcTAtGAGACCACTAGCAgTTCTCTcTCcTTCCTggcGTATaTacTGGCCACTCAcCCTGAcGTCCAGCAGAAGCTgCAGGAgGAGATTGATGcGACcTTCCCcAgcAAGGCg

>CYP3A94_elephant

ATGGACCTgATCCCAAgCTTTTCCATGGAgACCTGGaTTCTtCTGGtcACCAtCCTGGTaCTCCTTTATagATATGGaACCcATtCACATaatgTTcTaAAGAAGCTtGGgATTCCTGGGCCcAaACCTtTgCCTTTTTTGGGAACTtcTCTGGcCTACCTcAAGTTATGATGGtCgACAGCCTGTGaTaGCCATcACAGATCCAGtcATGATCAAgACAaTACTgGTGAAAGAATGTTATTCcacCTTCACAAAtCGGaGGGGTCCAaTGGGATTTATGAAatcTGCtATtTCctTGTCcGAGGATGAagtgTGGAAGAGAgTAcGAACATTGCTGTCTCCAACtTTCACCAGTGGAAAaCTCAAGGAGATGTTCCCCATCATcaGCCAaTATGGtGATGTGTTGGTGAaGcAtCTGAGagAGGAAgaAcAGAAAGGcAAgCCTGTtgCgTTGAAAAACATCTTTGGaGCtTACAGCATGGATGTGATaACTAGCACgTCATTTGGAGTGAAcATCGATTCCCTCcACAACCCACAAGATCCCTTTGTGcAAAAcgCCAgGAAGCTCaTAAagTTTgATTTCtTgGAcCCATTtgTTtTCTtAATAACACTCTTTCCATTcCTTActcCAaTTTgTGAAGcAtTAaACATcTcTcTGTTTCCAAgAgacGcTACTGATTTTTTCACAAAATCTGTAcAAAGaATGAAAGAAAGcCGCCTtAAAGATAAtgAAAAGCACCaAGTGGATTTgCTTCAGCTGATGATcgACTCcCAGAATTCCAAAGAAATtatgtCCCATAAAGCTtTGaCTGATaTGGAGCTTGTGGCtCAATCaATTATgTTTATTTTTGCTGGcTAtGAGACCACTAGCACTaCTCTTTCcTTCgTTATGTATtTaTTGGCCACaCAcCCaGATaTtCAGgAGAAaCTgCAGaggGAGATTGATGcagCTTTtCCcgATAAGGCaCcTCCCACgTATGATggCaTGTTACAGATGGAGTATCTTGACATGGTGgTGAATGAAaCaCTCAGATTaTTCCCAATcGCTGGcAGACTTGAGAGGGTCTGTAAGAAAGATGTtGAAATCAAgGGAcTGaTcATTCCCAAAGGGACAGTGaTGATGGTGCCAAtCTTTgTTCTcCAaCGAGaCcCAGAacaCTGGCCaGAaCCTGAtaAGTTCatTCCTGAAAGGTTCAGTAAGgAgAACAAGGACAACATAgATCCTTActTATAtaTGCCCTTTGGAAtTGGACCcCGAAACTGCATcGGCATGAGgTTTGCccTtATGAACATGAAACTTGCTATCaTCAGAaTtCTGCAGgAgTTtTCCaTCAAACCTTGTAAcGAAACgCAGATCCCCtTaAAAtTAGGCAAgagAGcACTTATTgcACCAgAacgACCtgTTGTTaTAAtGtTTGAtTCaAGAGtTGGaAaagtaAtTGGAGCCTGA

>CYP3A94_human

TCCCAgACTTggCCATGGAAACCTGGcTTCTCCTGGCTgtCAGCCTGGTGCTCCTcTATCTATATGGaACCcATtCACATGGACTTTTTAAGAAGCTtGGAATTCCaGGGCCcACACCTCTgCCTTTTTTGGGAAaTaTTtTGtcCTACCaTAAGGTTTATGATGGtCaACAGCCTGTGcTGGCtATcACAGATCCtGAcATGATCAAAACAGTgCTAGTGAAAGAATGTTATTCTGTCTTCACAAACCGGaGGgCTtTTGGTCCAGTGGGATTTATGAAaAgTGCCATCTCTaTagCTGAGGATGAagAATGGAAGAGAtTAcGAtCATTGCTGTCTCCAACCTTCACCAGTGGAAAaCTCAAGGAGATGgTCCCtATCATTGcCCAGTATGGAGATGTGTTGGTGAGaAAtCTGAGGcgGGAAgCAGAGAcAGGcAAgCCTGTCACCTTGAAaAACATCTTTGGGGCCTACAGCATGGATGTGATcACTAGCACATCATTTGGAGTGAAcATCGAcTCtCTCAACAAtCCACAAGAcCCCTTTGTGGAAAAcACCAAGAAGCTtTTAAGATTTgATTTttTgGATCCATTCTTTCTCTCAATAATTTCCATTcCTcAtccCAaTTcTTGAAGTAtTAaAtATcTgTgTGTTTCCAAgAgaaGTTACaaATTTTTTaAgAAAATCTGTAAAAAGGATGAAAGAAAGTCGCCTCgAAGATAcACAAAAGCACCGAGTGGATTTcCTTCAGCTGATGATTgACTCTCAGAATTCaAAAGAAActGAgtCCCAcAAAGCTCTGTCcGATCTGGAGCTcGTGGCCCAATCaATTATCTTTATTTTTGCTGGcTAtGAaACCACgAGCAgTgtTCTcTCcTTCaTTATGTATgaacTGGCCACTCAcCCTGATGTCCAGCAGAAaCTgCAGGAgGAaATTGATGcagtTTTaCCcAATAAGGCaCcaCCCACCTATGATACtgTGcTACAGATGGAGTATCTTGACATGGTGgTGAATGAAaCgCTCAGATTaTTCCCAATTGCTatgAGACTTGAGAGGGTCTGcAAaAAAGATGTtGAgATCAATGGgaTGtTcATTCCCAAAGGGgtgGTGGTGATGaTtCCAAgCTaTgcTCTTCAcCGtGaCcCAaAGTaCTGGaCaGAGCCTGAGaAGTTCctcCCTGAAAGGTTCAGcAAGAAgAACAAGGACAACATAgATCCTTAcATATACacaCCCTTTGGAAgTGGACCcaGAAACTGCATTGGCATGAGgTTTGCTcTCATGAACATGAAACTTGCTcTaaTCAGAGTcCTtCAGAACTTCTCCTTCAAACCTTGTAAAGAAACACAGATCCCCCTGAAAtTAaGCttaggAGGACTTcTTCAACCAgAaAAACCCgTTGTTCTAAAGGTTGAGTCaAGgGATGGcACCgtaAgTGGAGCCTGA

>CYP3A94_sheep

ATGGAgCTAATCCCAAgCTTTTCCcTGGAAACCTGGGTTCTCCTGGCTACCgGCCTGGTGCTCCTcTATCTATATGGaACtTATtCACATGGACTgTTTAAGAAGCTGGGAgTTtCTGGGCCaAgACCTCTgCCTTaTTTtGGAAaTGTTCTGtcCTACCgaAAGGAATGTTTTAAAAAGTATGGgAaaATGTGGGGGCCTcTtTTGGttATcACAGATCCAGAcATGATCAAAACAGTACTAGTGAAAGAATGTTAcTCTGTCTTCACAAACCGGaGGgCTCTTGGTCCAaTGGGAaTTATGAAaAATGCtgTtTCTgTGgCTGAGGATGAaCAATGGAAGAGAATAcGgACATTGCTGTCTCCAACCTTCACCAGTGGgAAGCTCAAaGAGATGTTtCCtATCATTGGgaAGTATGGAGAcGTGTTGGTGAGGAACCTGAGGAAGGAAgCAGAGAAAGGcCATCTTTGGGGCCTACAGCATGGATGTGATTACTAGCACATCATTTGGAGTGAATATtGATTCCCTtggCAACCCACAAGATCCCTTTGTGGAgAATgCCAAGAAGCTCTTAAGATTTAATaTCCTCGATCCATTtcTTCTCTCAATAgtACTCTTTCCATTcCTTgtccCAaTcTTTGAAGTAtTAaAtATcaccATGTTTCCcAAAaGTGcTgtgGATTTTTTgACAAAATCTGTAAAAAGGATaAAAGAAAGTCGCCTCAAAGATAAtCAAAAGCACCGtGTGGAcTTTCTTCAGtTGATGATTAACTCTCAGAATTCCAAAGAAAcaGACAaCCATAAAGCTCTcTCTGAcCaaGAaCTcaTGGCCCAAagTgTTATCTTTATTTTTGCcGGcTAtGAGACCACTAGCAaTaCgCTTTCcTTCCTTtTGTATaTTTTGGCCACTCAcCCTGATGTCCAGCAGAAGCTgCAGGAgGAGATTGATGcGACTTTCCCcAATAAGGCaCcTCCgACCTAcGATgtCCTGgcACAGATGGAGTATCTTGACATGGTGgTGAATGAgaCTCTCAGAaTGTTtCCAATTGCTGtTAGACTTGAtAGGcTCTGTAAGAAgGATGTGGAAATCcAcGGgGTGtccATTCCCAAAGGGACAGcGGTGAcGGTGCCAAtCTTTgTgCTTCAcaGAGaCcCAcAGcTCTGGCCaGAGCCTGAGGAGTTtcGTCCTGAAAGGTTCAGTAAGAAgAACAAGGACAgCATAAATCCTTATgTATACCTGCCtTTTGGAACTGGACCcCGAAAtTGCATTGGCATGAGgTTTGCcATCATGAACATGAAACTTGCTATtGTCAGAGTcCTGCAGAACTTCTCCTTCAAACCTTGTAAAGAAACACAGg

>CYP3A94_mouse

ATGGACCTgATCCCAAACTTTTCCATGGAAACCTGGaTgCTCCTGGCTACCAGCCTGGTcCTgCTTcATagATATGGaACtcATtCACATGGAaTTTTTAAaAAGtTGGGAATTCCTGGGCCaAaACCTCTgCCTTTcTTGGGgACgaTTCTtGctTACCagAAGGTATGAcGGtCgACAGCCTGTGcTGGCtATcACAGATCCAGAcATaATCAAAACAGTgCTgGTGAAgGAATGTTAcTCTacCTTCACAAACCGGCGGTCTCTTGGTCCAGTGGGtaTTtTGAAaAAgGCCATCTCTaTcTCTGAGaATGAagAATGGAAGAGAATccGAgCccTGCTGTCTCCAACCTTCACCAGTGGgAgGCTCAAGGAGATGTTCCCCATCATTaaCCAGTtTacAGATGTGTTGGTGAGCATCTTTGGGGCCTACAGCATGGATGTGATcACagcCACcTCATTTGGAGTGAATATtGATTCCCTCAACAACCCACAgGATCCtTTTGTGGAgAAaAtCAAGAAGCTCTTAAaATTTgATaTCtTtGATCCAcTgTTcCTCTCAgTgACACTCTTTCCATTcCTTAcAGCAaaGAGTGGATTTTCTTCAGCTGATGATaAACTCcCAGAATTaCAAAaGCatTaTCTGATgTGGAGaTTGTGGCtCAgTCagTTATCTTTATTTTTGCTGGcTAtGAGACCACaAGCAgTgCTCTTTCcTTtgcatTGTATtTgcTGGCCAtTCAcCCTGATGTaCAGaAGAAaCTTCAGGATGAaATTGATGcGgCTcTgCCcAATAAGCCACCTATGATACCCTGcTACAGATGGAGTATCTaGACATGGTGgTGAATGAAaCTCTCAGATTaTatCCAATcGCTGGaAGACTTGAGAGGGTCTGTAAGAcAGATGTtGAAATCAATGGgcTatTcATTCCCAAAGGGACtGTGGTGATGaTaCCAACCTTTgcTCTTCAcaaAGaCcCgaAGTaCTGGCCaGAGCCTGAGGAaTTCcGcCCTGAAAGGTTCAGcAAGAAgAAtcAGGACAgCATcAATCCTTAcATgTACCTGCCCTTTGGgAgTGGACCaaGgAACTGCATTGGCATGAGgTTTGCTcTCATaAACATGAAAgTTGCTcTtGTCAGAGTcCTGCAGAACTTCaCtgTCcAgCCTTGTAAgGAAACt

CYP3A95

>CYP3A95_horse

ATGGACCTGATCCCAAGCCTTTCCATGGAAACGTGGGTTCTCCTAGCTACCAGTCTAGTGCTCCTCTATCTGTATGGGACCTATTCACATGGACATTTTAAGAAACTGGGAATTCCTGGGCCAACACCTCTGCCATTTTTTGGAACTGTTCTGGGCTACATTAAGGGTGTTTTGGCTTTTGACAAGAAATGTTTTAAGAAATATGGAAAAATGTGGGGGTTTTATGATGGTCGACAGCCCGTGTTGGCTATCACAGATCCAGACATGATCAAAACAGTACTAGTGAAAGAATGTTATTCTGTCTTCACAAACCGGCGGTCTTTTGGTCCAATGGGACTTATGAAAAATGCCATCACTATAGCTGAGGATGAAAAATGGAAGAGAATACGAACATTACTGTCTCCAACCTTCACCAGTGGGAAGCTCAAGGAGATGTTCCCTATCATTGGCCAGTATGGAGATATGTTGGTGAGGAACCTGAGGAAGGAAGCAGAGAAAGGCAAACCCATCACCATGAAAGACATCTTTGGGGCCTACAGCATGGATGTGATTACTAGCACATCATTTGGAGTGAACATAGATTCCCTCAATAACCCACAAGATCCTTTTGTGGAAAATACTAAGAAGCTCTTAAGATTTgATTTCCTTGATCCATTCTTCCTCTCAATACTATTCTTTCCATTTCTTATCCCAGTTTATGAAGCATTAAATATCTTTcTTTTTCCAAAATGTGTTACTGATTTCTTCATAAAATCTGTAAAAAAGATGAAAGAAAGTCGTCTCAAAGATAAAGCAAAGCAACGAGTGGATTTTCTTCAGCTAATGATTAACTCCCAAAATTCCAAAGAAATAGACAACCTTAAAGTTCTGTCTGATCTAGAACTTGTGGCCCAATCTATTACGTTTATCTTTGCTGGCTATGAGCCTGTTAGCACTTCTCTTTCCTTCCTTCTGTATCTTTTGGCCACTCACCCTGATGTCCAGCAGAAGCTACAGGAGGAGATTGATGCGACTTTCCCCAATAAGGCACCTCCTACCTATGATGCCCTGGTACAGATGGAATATCTTGATATGGTGTTGAATGAAACTCTCAGATTATTCCCAATTGCTGATAGACTTGAGAGGGTCTGTAAGAAAGATGTGGAAATCAATGGGGTGTTCATTCCCAAAGGGtCAACAGTGATGATGCCAATCTTTGTTCTTCACCAACACCCAGAGTTCTGGTCGGAACCTGAGGAGTTCCGTCCTGAAAGaTTCAGTAAGAAGAACAAGGACAGCATAAATCCTTATACATACCTGCCCTTTGGAACTGGACCCCGAAACTGCATTGGCATGAGGTTCGCTATCGTAAACATAAAACTTGCGGTTGTCAGAGTGCTGCAGAACTTCTCCTTCAAACCTTGTAAAGAAACGCAGATTCCCCTTAAATTAGTCAGTCAAGGACTTATTCGACCGGAAAAACCCATTGTTCTGAAAGTTGAGTCGAGAGATGGGACCATGAGTGGAGCCTGA

>CYP3A95_rhino

ATGGACCTGATCCCAAGCtTTTCCATGGAAACGTGGGTTCTCCTgGCTACCAGcCTgGTGCTtCTCTATCTGTATGGGACCTATTCACATGGACtTTTTAAGAAgCTGGGAATTCCTGGGCCgACACCTCTGCCATTTTTTGGAACaGTTCTGGGCTACcTTAAGTTTGACAAGAAATGTTTTAAaAAATATGGAAAtATGTGGGGGTTTTATGATGGcCcACAGCCtGTaTTGGCTATCACAGATCCAGACATGATCAAAACAGTACTAGTGAAAGAATGTTAcTCTGTCTTCACAAACCGGCGGTCTTTTGGTCCAATGGGAtTTATGAAAAAaGCCATCtCTcTgtCTGAGGATGAAcAATGGAAGAGAATACGAAatTTgCTGTCTCCAACCTTCACCAGTGGaAAGCTCAAGGAGATGTTCCCcATCATTGGCCAGTATGGAaATATGTTGGTGAaGAACCTGAGGAAGGAAGCAGAGAAAGGCAAgCCCgTtACCtTGAAAGCATCTTgGGGGCCTACAGCATGGATGTGATTACTAGCACATCATTTGGAGTGAACATcGATTCCCTCAAcAACCCACAAGATCCcTTTGTGGAAAActgcAAGAAGCTCTTAAGATTTGATTTCCTTGATCCATTCaTtCTCTCAATATTTCCATTTCTTAatCCAGTTTtTGAAGCATTAAATATCTTTCTgTTTCCAAAAaGTGTTACTGATTTtTTtgcAAAATCTGTAcAAAgGATaAAAGAAAGTCGcCTCAAAGATAAAGaAAAGCAgCGAGTtGATTTTCTTCAGCTgATGATTAACTCCCAgAATTCCAAAGAAcTgGACCCTTttAGcTCTGTCTGATCTgGAgCTcaTaGCCCAATCTATTAtcTTTATtTTTGCTGGCTATGAGCCcaTTAGCACTTCTCTTTCCTTCCTTaTaTATtTTTTGGCCACTCACCCTGATGTCCAGCAGAAGCTgCAGGAGGAGATTGATGCaACTTTCCCCgATAAGGCACCTgtcACtTATGATGCCCTGaTACAGATGGAgTATCTTGAcATGGTGTTGAATGAAtCTCTCAGATTATTCCCAATTGCTGgTAGACTTGAGAGGGTCTGTAAGAAAGATtTGGAAcTCggTGGGGTGTTCATTCCCAAAGGGaCAgtAGTGATGgTGCCAAcCTTTtcTCTTCACCgAgAaCCAGAGTTCTGGcCtGAgCCTGAGGAGTTCCaTCCTGAAAGgTTCAGTAAGgAcAACAAGGACAGtATAAATCCTTATAtATACaTGCCgTTTGGAAgTGGACCCCGAAACTGCATTGGCATGAGaTTtGCTgTCGTgAACATgAAACTTGCtGTTGTCAGAcTGCTGCAGAACTTCTCCTTCAAACCTTGcAAAGAAACGCAGATTCCCCTgAAATTAGgCAcTCAAGGACTcATTCaACCGGAAAAACCaATTGTTCTaAAAGTTGAGTCGAGAGcTGGGACtgTGAaTGGAGCCTGA

>CYP3A95_cow

ATGGAgCTaATCCCAAGCtTTTCCATGGAAACcTGGGTTCTCCTgtCTAtCAGcCTgGTGCTCCTtTATCTGTATGGaACtTATTCACAcGGACtaTTTAAGAAgCTGGGAgTTCCTGGGCCAAgACCTCTGCCtTaTTTTGGAAaTGTTCTGtcCTACcgaAAGGGTGTTTgtGagTTTGAtgAagAATGTTTTAAaAAgTATGGgAAAATGTGGGGGaTTTtTGAaGGTaaACAtCCtcTGTTGGtTATCACgGATCCAGACATGATCAAAACAGTACTAGTGAAAGAATGTTATTCTGTCTTCACAAACCGgaGGgTTTTGGTCCAATGGGAgTTATGAAAAATGCtgTttCTgTgGCTGAGGATGAAcAATGGAAGAGAATACGgACATTgCTGTCTCCAACCTTCACCAGTGGGAAGCTCAAGGAGATGTTCCCTATCATTGGgaAGTATGGAGATgTGTTGGTGAGGAACCTGAGGAAGGAAGCAGAGAAAGGCAcgtCCgTCgaCATGAAAGATCTTTGGGGCCTACAGCATGGATGTGATTACTAGCACATCATTTGGAGTGAAtATtGATTCCCTtggcAACCCACAAGATCCcTTTGTGGAgAATgCcAAGAAGCTCTTAAGATTTGATaTCCTTGATCCATTtcTaCTCTCtAcAgTAcTCTTTCCATTcCTTgTCCCAaTcctTGAtGtATTAAATATCaccaTaTTTCCAAAAaGTGcTgtgaATTTtTTtAcAAAATCTGTAAAAAgGATaAAAGAAAGTCGcCTCAAAGATAAtcaAAAGgtACGtGTGGAcTTTCTTCAGCTgATGATTAACTCCCAgAATTCCAAAGAAAcAGACAAtCaTAAAGcTCTcTCTGAcCaAGAACTcaTaGCCCAgagTATTAtcTTTATtTTTGCTGGCTATGAGaCcacTAGCAgTaCTCTTTCCTTCCTTCTGTATaTTTTGGCCACTCACCCTGATGTCCAGCAGAAGCTgCAGGAGGAGATTGATGCaACTTTCCCCAATAAGGCcCCTCCgACCTATGATGtCCTGGcACAGATGGAgTAcCTTGAcATGGTGgTGAATGAgACTCTCAGAaTgTTtCCtATTGCTatTAGACTTGAGAGGcTCTGTAAGAAgGATGTGGAAATCcATGGGGTGTcCATTCCCAAAGGGaCAACAGTGATGgTGCCAATCTccGTgCTgCACaAAgACCCAcAGcTCTGGcCaGAgCCTGAGGAGTTCCGTCCTGAAAGgTTCAGTAAGAAGAACAAGGACAGCATAAATCCTTAcgtcTACCTGCCtTTTGGAACTGGACCCCGAAAtTGCATTGGCATGAGGTTtGCTATCaTgAACATgAAACTTGCtaTcGTCAGAGTcCTGCAGAACTTCTCCTTCAAACCTTGTAAAGAAACGCAGATcCCCtTgAAAaTAagCAGTCAAGGAgTTtTaaGACCGGAAAAACCtgTTGTcCTaAAgGTTGtGctcAGAGATGGaACCATaAGTGGAGCCTGA

>CYP3A95_dog

TATagGTATGGGACCTAcaCACATGGggtTTTTAAGAAgCTaGGAATTCCTGGaCCAACACCTCTGCCtTTTgTgGGAACTGcTCTGGGCTACcgTAAGGGTTTTATGATGGgCGACAGCCtGTGcTGGCTATCACAGATCCgGACATGATCAAAACAGTgCTAGTGAAAGAATGTTATTCTGTCTTCACAAACCGGCGGTCTTTTGGTCCAgTGGGAtTTATGAAAAgTGCCATCtCTcTgtCTGAGGATGAAgAATGGAAGAGAATACGAACATTgCTGTCcCCAACCTTCACCAGTGGaAAGCTCAAGGAGATGTTCCCcATCATTGGCCAGTATGGAGATgTGTTGGTGAGGAACCTGAGGAAGGAgGCAGAGAAAGGCAAAtCCATCAaCtTGAAAGACATCTTTGGaGCCTACAGCATGGATGTGATTACcAGCACATCATTTGGAGTGAACATtGATTCCCTCAAcAACCCACAAGATCCcTTTGTGGAAAATAtcAAGAAGCTCTTAAaATTTGATTTCCTTGATCCATTtTTCtTCTCAATAtCGAGTGGAcTTTCTTCAGCTgATGATTAACTCCCAgAATTCtAAAGAAAcAGACCTaTCTGATtTgGAgCTgGTGGCCCAATCTATTAtcTTTATtTTTGCTGGCTATGAGaCcacTAGCACTTCTCTTTCCTTCCTTaTGTATgaaTTGGCCACTCACCCTGATGTCCAGCAGAAaCTgCAGGAGGAGATTGATGCGACTTTCCCCAATAAGGCAttgCCcACtTAcGATGCCCTtGTgCAGATGGAATATCTgGAcATGGTGTTGAATGAAACTCTCcGATTATaCCCAATcGCTGgTAGACTTGAGAGGGTCTGTAAGAAAGATGTGGAAATCAgTGGtGTGTTCATTCCCAAAGGGaCAgtgGTGATGgTGCCAAcCTTTacTCTTCAtCgAgACCagagtcTCTGGcCaGAgCCTGAGGAaTTCCGaCCTGAAAGgTTCAGTAgGAAGAACAAGGACAGCATAAATCCTTATACATACCTGCCtTTTGGAACTGGACCCCGAAACTGCATTGGaATGAGGTTtGCgATCaTgAACATgAAACTTGCccTTGTtAGgGTcCTGCAGAACTTCTCCTTCAAACCTTGTAAAGAAACGCAGATcCCCCTgAAATTAaatgcTCAAGGgaTTATTCaACCtGAAAAgCCCATTGTTCTcAAgGTTGAGcCaAGAGATGGGAgtgTaAaTGGAGCCTGA

>CYP3A95_pig

ATGGACCTGATCCCAAGCtTTTCtgTGGAAACcTGGcTgCTtCTgGCTACCAGcCTgGTGCTCCTCTATCTGTATGGGACCTATTCACAcGGACtTTTTAAGAAgCTGGGgATTCCcGGGCCgAaACCTCTGCCtTaTTTTGGAAgTGTTCTaGaaTACcgTAAGGtTTTGACAAtAAATGTTTTAAaAAgTATGGgAAAATGTGGGGGTTTTATGAcGGTaGACAGCCtGTGcTGGCTATgAgAGAcCCAGAaATGATCAAAgCAGTgCTtGTGAAAGAATGTTAcTCgGctTTCACAAAtCGGCGGTCTTTTGGTCCAgcGGGAgTTATGAAAAATGCCATCtCTgTgGCTaAGGATGAgcAATGGAAGcGAgTACGAACATTgCTGTCTCCAACCTTCACCAGTGGaAAGCTCAAaGAGATGTTCCCcATCATTGcCCAGTATGGAGATATGcTGGTGAGGAACCTGAGGAAGGAAGCAGAGAAAGGCAAgtCCATCAaCAcGAAAGAATCTTTGGGGCCTACAGCATGGAcGTGATcACaAGCACAgCATTTGGAGTGAACTTTGATTTCCTcGATCCATTtaTtCTCTCAATACTATTCTTTCCATTcCTcAgCCCAtTcTtTGAAGtATTAgAcATCacTtTgTTTCCAAgAaGTtcTgtgaAaTTtTTCAcAcAgTCTGTAAAAAgGATGAAAGAAAGTCGcCTCAAAGATCGAGTGGAccTTCTTCAGCTgATGATTAACTCCCAgAATTCCAAAGAAAcgGAtAGcTCTGTCTGATCaAGAACTTGTGGCCCAAagTATTAtcTTcATtTTTGCcGGCTATGAGaCcacTAGCAgTTCTCTcTCCTTCCTggcGTATaTacTGGCCACTCACCCTGAcGTCCAGCAGAAGCTgCAGGAGGAGATTGATGCGACcTTCCCCAgcAAGGCg

>CYP3A95_mouse

ATGGACCTGATCCCAAaCtTTTCCATGGAAACcTGGaTgCTCCTgGCTACCAGcCTgGTcCTtCTCTActTGTATGGaACtcATTCACATGGAatTTTTAAaAAgtTGGGAATTCCTGGGCCAAaACCTCTGCCtTTcTTgGGgACgaTTCTtGctTACcagAAGTGTcaTAAaAAATATGGgAAAATGTGGGGGTTgTATGAcGGTCGACAGCCtGTGcTGGCTATCACAGATCCAGACATaATCAAAACAGTgCTgGTGAAgGAATGTTAcTCTacCTTCACAAACCGGCGGgTTTGGTCCAgTGGGtaTTtTGAAAAAgGCCATCtCTATctCTGAGaATGAAgAATGGAAGAGAATcCGAgCccTgCTGTCTCCAACCTTCACCAGTGGGAgGCTCAAGGAGgCATCTTTGGGGCCTACAGCATGGATGTGATcACagcCACcTCATTTGGAGTGAAtATtGATTCCCTCAAcAACCCACAgGATCCTTTTGTGGAgAAaAtcAAGAAGCTCTTAAaATTTGATaTCtTTGATCCAcTgTTCCTCTCAgAGCAAaGAGTGGATTTTCTTCAGCTgATGATaAACTCCCAgAATTaCAAAatTaTCTGATgTgGAgaTTGTGGCtCAgTCagTTAtcTTTATtTTTGCTGGCTATGAGaCcacaAGCAgTgCTCTTTCCTTtgcatTGTATtTgcTGGCCAtTCACCCTGATGTaCAGaAGAAaCTtCAGGAtGAaATTGATGCGgCTcTgCCCAATAAGGCACCTgCcACCTATGATaCCCTGcTACAGATGGAgTATCTaGAcATGGTGgTGAATGAAACTCTCAGATTATatCCAATcGCTGgaAGACTTGAGAGGGTCTGTAAGAcAGATGTtGAAATCAATGGGcTaTTCATTCCCAAAGGGaCtgtgGTGATGATaCCAAcCTTTGcTCTTCACaAAgACCCgaAGTaCTGGcCaGAgCCTGAGGAaTTCCGcCCTGAAAGgTTCAGcAAGAAGAAtcAGGACAGCATcAATCCTTAcAtgTACCTGCCCTTTGGgAgTGGACCaaGgAACTGCATTGGCATGAGGTTtGCTcTCaTAAACATgAAAgTTGCtcTTGTCAGAGTcCTGCAGAACTTCaCtgTCcAgCCTTGTAAgGAAACt

>CYP3A95_sheep

ATGGAgCTaATCCCAAGCtTTTCCcTGGAAACcTGGGTTCTCCTgGCTACCgGcCTgGTGCTCCTCTATCTGTATGGaACtTATTCACATGGACtgTTTAAGAAgCTGGGAgTTtCTGGGCCAAgACCTCTGCCtTaTTTTGGAAaTGTTCTGtcCTACcgaAAGGGTGTTTgtGaaTTTGAtgAagAATGTTTTAAaAAgTATGGgAAAATGTGGGGGCCtcTtTTGGtTATCACAGATCCAGACATGATCAAAACAGTACTAGTGAAAGAATGTTAcTCTGTCTTCACAAACCGGaGGgTTTTGGTCCAATGGGAaTTATGAAAAATGCtgTttCTgTgGCTGAGGATGAAcAATGGAAGAGAATACGgACATTgCTGTCTCCAACCTTCACCAGTGGGAAGCTCAAaGAGATGTTtCCTATCATTGGgaAGTATGGAGAcgTGTTGGTGAGGAACCTGAGGAAGGAAGCAGAGAAAGGCAcgtCCgTCAaCATGAAAGACATCTTTGGGGCCTACAGCATGGATGTGATTACTAGCACATCATTTGGAGTGAAtATtGATTCCCTtggcAACCCACAAGATCCcTTTGTGGAgAATgCcAAGAAGCTCTTAAGATTTaATaTCCTcGATCCATTtcTtCTCTCAgAAAATCTGTAAAAAgGATaAAAGAAAGTCGcCTCAAAGATAAtcaAAAGCAACGtGTGGAcTTTCTTCAGtTgATGATTAACTCtCAgAATTCCAAAGAAAcAGACAACCaTAAAGTTCTcTCTGAcCaAGAACTcaTGGCCCAAagTgTTAtcTTTATtTTTGCcGGCTATGAGaCcacTAGCAaTaCgCTTTCCTTCCTTtTGTATaTTTTGGCCACTCACCCTGATGTCCAGCAGAAGCTgCAGGAGGAGATTGATGCGACTTTCCCCAATAAGGCACCTCCgACCTAcGATGtCCTGGcACAGATGGAgTATCTTGAcATGGTGgTGAATGAgACTCTCAGAaTgTTtCCAATTGCTGtTAGACTTGAtAGGcTCTGTAAGAAgGATGTGGAAATCcAcGGGGTGTcCATTCCCAAAGGGaCAgCgGTGAcGgTGCCAATCTTTGTgCTTCACagAgACCCAcAGcTCTGGcCaGAgCCTGAGGAGTTtCGTCCTGAAAGgTTCAGTAAGAAGAACAAGGACAGCATAAATCCTTATgtATACCTGCCtTTTGGAACTGGACCCCGAAAtTGCATTGGCATGAGGTTtGCcATCaTgAACATgAAACTTGCtaTTGTCAGAGTcCTGCAGAACTTCTCCTTCAAACCTTGTAAAGAAACGCAGATcCCCtTgAAgaTAaaCAGTCAAGGACTgATaaGACCaGAAAAACCCATTtTTCTGAAgGTTGtGctcAGAGATGaGACCATaAGTGGAGCCTGA

>CYP3A95_elephant

ATGGACCTGATCCCAAGCtTTTCCATGGAgACcTGGaTTCTtCTgGtcACCAtcCTgGTaCTCCTCTActTGTATGGaACCcATTCACATaatgtTcTaAAGAAgCTtGGgATTCCTGGGCCcAaACCTtTGCCtTTTTTgGGAACTtcTCTGGcCTACcTcAAGGGTTTTATGATGGTCGACAGCCtGTGaTaGCcATCACAGATCCAGtCATGATCAAgACAaTACTgGTGAAAGAATGTTATTCcacCTTCACAAAtCGGaGGgGGTCCAATGGGAtTTATGAAAtcTGCtATttCctTgtCcGAGGATGAAgtgTGGAAGAGAgTACGAACATTgCTGTCTCCAACtTTCACCAGTGGaAAaCTCAAGGAGCATCTTTGGaGCtTACAGCATGGATGTGATaACTAGCACgTCATTTGGAGTGAACATcGATTCCCTCcAcAACCCACAAGATCCcTTTGTGcAAAAcgCcAgGAAGCTCaTAAagTTTGATTTCtTgGAcCCATTtgTttTCTtAATTTTCCATTcCTTActCCAaTTTgTGAAGCATTAAAcATCTcTCTgTTTCCAAgAgacGcTACTGATTTtTTCAcAAAATCTGTAcAAAgaATGAAAGAAAGcCGcCTtAAAGATAAtGaAAAGgtAaGAGTGGATTTgCTTCAGCTgATGATcgACTCCCAgAATTCCAAAGAAATttTGaCTGATaTgGAgCTTGTGGCtCAATCaATTAtGTTTATtTTTGCTGGCTATGAGaCcacTAGCACTaCTCTTTCCTTCgTTaTGTATtTaTTGGCCACaCACCCaGATaTtCAGgAGAAaCTgCAGagGGAGATTGATGCagCTTTtCCCgATAAGGCACCTCCcACgTATGATGgCaTGtTACAGATGGAgTATCTTGAcATGGTGgTGAATGAAACaCTCAGATTATTCCCAATcGCTGgcAGACTTGAGAGGGTCTGTAAGAAAGATGTtGAAATCAAgGGacTGaTCATTCCCAAAGGGaCAgtgaTGATGgTGCCAATCTTTGTTCTcCAaCgAgACC

CAGAacaCTGGcCaGAACCTGAtaAGTTCatTCCTGAAAGgTTCAGTAAGgAGAACAAGGACAaCATAgATCCTTActtATAtaTGCCCTTTGGAAtTGGACCCCGAAACTGCATcGGCATGAGGTTtGCccTtaTgAACATgAAACTTGCtaTcaTCAGAaTtCTGCAGgAgTTtTCCaTCAAACCTTGTAAcGAAACGCAGATcCCCtTaAAATTAGgCAagagAGcACTTATTgcACCaGAAcgACCtgTTGTTaTaAtgtTTGAtTCaAGAGtTGGaAaagTaAtTGGAGCCTGA

>CYP3A95_human

TCCCAgaCtTggCCATGGAAACcTGGcTTCTCCTgGCTgtCAGcCTgGTGCTCCTCTATCTGTATGGaACCcATTCACATGGACtTTTTAAGAAgCTtGGAATTCCaGGGCCcACACCTCTGCCtTTTTTgGGAAaTaTTtTGtcCTACcaTAAGGTTTTATGATGGTCaACAGCCtGTGcTGGCTATCACAGATCCtGACATGATCAAAACAGTgCTAGTGAAAGAATGTTATTCTGTCTTCACAAACCGGaGggCTTTTGGTCCAgTGGGAtTTATGAAAAgTGCCATCtCTATAGCTGAGGATGAAgAATGGAAGAGAtTACGAtCATTgCTGTCTCCAACCTTCACCAGTGGaAAaCTCAAGGAGGTCTTTGGGGCCTACAGCATGGATGTGATcACTAGCACATCATTTGGAGTGAACATcGAcTCtCTCAAcAAtCCACAAGAcCCcTTTGTGGAAAAcACcAAGAAGCTtTTAAGATTTGATTTttTgGATCCATTCTTtCTCTCAATACTATTCTTTCCATTcCTcATCCCAaTTctTGAAGtATTAAATATCTgTgTgTTTCCAAgAgaaGTTACaaATTTtTTaAgAAAATCTGTAAAAAgGATGAAAGAAAGTCGcCTCgAAGAtCGAGTGGATTTcCTTCAGCTgATGATTgACTCtCAgAATTCaAAAGAACTGTCcGATCTgGAgCTcGTGGCCCAATCaATTAtcTTTATtTTTGCTGGCTATGAaaCcacgAGCAgTgtTCTcTCCTTCaTTaTGTATgaacTGGCCACTCACCCTGATGTCCAGCAGAAaCTgCAGGAGGAaATTGATGCagtTTTaCCCAATAAGGCACCaCCcACCTATGATaCtgTGcTACAGATGGAgTATCTTGAcATGGTGgTGAATGAAACgCTCAGATTATTCCCAATTGCTatgAGACTTGAGAGGGTCTGcAAaAAAGATGTtGAgATCAATGGGaTGTTCATTCCCAAAGGGgtggtgGTGATGATtCCAAgCTaTGcTCTTCACCgtgACCCAaAGTaCTGGaCaGAgCCTGAGaAGTTCCtcCCTGAAAGATTCAGcAAGAAGAACAAGGACAaCATAgATCCTTAcAtATACacaCCCTTTGGAAgTGGACCCaGAAACTGCATTGGCATGAGGTTtGCTcTCaTgAACATgAAACTTGCtcTaaTCAGAGTcCTtCAGAACTTCTCCTTCAAACCTTGTAAAGAAACGCAGATcCCCCTgAAATTAagCttaggAGGACTTcTTCaACCaGAAAAACCCgTTGTTCTaAAgGTTGAGTCaAGgGATGGcACCgTaAGTGGAGCCTGA

>CYP3A96_ horse

ATGGACCTGATCCCAAGCTTTTCTGTGGAAACATGGGTTCTCCTGGCTACCAGCCTGGCTCTCCTCTATCTATATGGGATCTATACACATGGACTTTTTAAGAAGCTGGGAATTCCAGGGCCAAAACCTCTCCCTTTTTTGGGAACTCTCCTGGGCTACCGTAAGGGTTTCGGGGGTTTTGACACGGAATGTCTTAAAAAATACAGAAAAATGTGGGGGTTTTATGATGGTCGACAGCCTGTGTTGGCTATCACAGATCCAGACTTGATCAAAACTGTACTAGTGAAAGAATGTTATTCTGTCTTCACAAACCGGCGATCTTTTGGTCCAGTGGGGTTTATGAAAAATGCCATCTCTATATCTGAGGATGAACAATGGAAGAGAATACGAACTTTGCTGTCTCCAACCTTCACCAGTGGAAAGCTCAAGGAGATGTTCCCCATCATTGGCCAGTATGGAGATGTGTTGGTGAGGAACCTGAAGAAAGAAGCAGAGAAAGGCAAGCCCATTGCCTTGAAAGACATTTTTGGGGCCTACAGCATGGATGTGATTACTAGTACATCATTTGGAGTGAACATCGATTCCCTCAACAACCCACAAGATCCCTTTGTGGAAAATACAAAGAAACTCTTAAGATTTGATTTCCTTGATCCATTCATTCTCTCAATAACAATTTTTCCATTTCTTAATCCAGTTTTTGAATTATTAAATATATTTCTATTTCCAAAGAGTGTTACTGATTTTTTCACAAAATCTGTAAAAAGGATAAAAGAAAGTCGCCTCAAAGATAAAGAAAAGCAACGAGTGGATTTCCTTCAGCTGATGATTAACTCCCAAAACTCCAAAGAAATGGACACCCATAAAGCTCTGTCTGATCTGGAGCTTGTAGCCCAATCTATTATATTTATTTTTGCTGGCTATGAGCCCGTCAGCAGTTCTCTTTCTTTCCTTCTGTATCTTTTGGCCACTCACCCTGATGTCCAGCAGAAGCTGCAGGAGGAGATTGATGCAACTTTCCCCAATATGGCACCTCCCACCTATGATGCTCTGGTACAGATGGAGTATCTTGACATGGTGTTGAATGAGTCTCTCAGATTATTCCCAGTTGCTGGTAGAATTGAAAGGACCTGTAAGAAAGATGTGGAACTTGGTGGGGTATTCATTCCCAAAGGAACAGTGGTGATGGTGCCATCCTTTGCTCTTCACCGAGACACAGAGCTCTGGCCACAACCTGAGGAGTTCCATCCAGAAAGGTTCAGCAAGGAGAACAAGGACAGCATAAATCCTTATATATATATGCCCTTTGGAAATGGACCCCGAAACTGCATTGGCATGAGGTTTGCTCTGATGAACATGAAAGTTGCTGTTGTCAGAGTGCTGCAGAACTTCTCCTTCAAACCTTGTAAAGAAACACAGATTCCCCTGAAATTAGTCACTTATGGATTTCTTCAACCAGAAAAACCAATTGTTCTAAAAGTTGAGTCCAGAGCTGGGACCGTGAGTGGAGCCTGACTTTCCCTGAGGTCTTCTGCTTTGGTCTTCAAGGAAGCTATATCCCAGAACAGGAGAGACCTCGATTTACTTTGTGAATGAAATCCAGAATAAAGATTATCTTAACCT

>CYP3A96_rhino

ATGGACCTGATCCCAAGCTTTTCcacaGAAACcTGGGTTCTCCTGGCTACCAGCCTGGtgCTCCTCTATCTgTATGGGAcCTATACtCATGGcCTTTTTAAGAAGCTGGGAATTCCtGGGCCgAcACCTCTgCCTTTTTTGGGAcaTaTtCTGaGCTACCaTAAGGGTTTttGGGaTTTTGACAataAATGTtTTAAAAAgTAtgGAAAAATGTGGGGGTTTTATGATGGTaGACgGCCTGTGTTGGCTATCACAGATCCAGACaTGATCAAAACaGTACTAGTGAAAGAATGTTATTtTGTtTTCACAAACCGGCaCTTTTGGTCCAcTGGGaTTTATGAAAAcTGCCgTCTCTcTgTCTGAGGATGAAaAATGGAAGAGAATACGAAtgTTGCTGTCTCCAACCTTCACgAGTGGAAAGCTCAAGGAGATGTTCCCCATCATTGGCCAGTATGcAGAcaTGTTGGTGAGtAACCTGAgGAAgGcAGCAGAGAAAGGCAAGCCCATcaCCTTGAAAGACATcTTTGGGGCCTACAGCATGGATGTGATTACcAGcAtATCATTTGGAGTGAACATCGATTtCCTCAACAACCCACAAGATCCCTTTGTGGAAAATgCcAAGAAgCTCTTtAGATTTGATTTCCTcaATCCATTCATTCTCTCAATAACtcTcTTTCCAaTTgTTAATCCAGTTTTTGAATTATTAAATATcTcTCTATTTCCAAAaAGTGTTACTGATTTTTTCACAAAATCTGTAAAAAGGATgAAAGAAAGcCGCCTCAAAGATAAgGAAAAGCAgCGAGTGGATTTtCTTCAGCTGATGATTAACTCCCAgAAtTCCAAAGAAcTGGACACCCATAAAGCTCTGTCTGATCTGGAGCTTGTgGCCCAATCTATTATcTTTATTaTTGCTGGCTATGAGaCCactAGCAGTTCTCTTTCcTTCCTTaTGTATCTTTTGGCCACTCACCCTGATGTCCAGCAGAAGCTGCAGGAGGAGATTGATGagACTTTtCCCAATAaGGaACCTCCCACCTATGATGCTCTGGTACAGATGGAGTATCTTGACATGGTGTTGAATGAaagTCTCAGATTATTCCCAaTTGCTGtcAGAcTacAgAGGgtCTGTAAGAAAGATGTGGAAaTccaTGGGGTgTTCATTCCCAAAGGgACAGTGGTGATGGTGCCcaCaTTTGCTCTTCACCGAGcCtCgGAGtTCTGGCCAgAgCCTGAGGAGTTCCgTCCtGAAAGGTTCAGtAAGaAGAAtAAGGACAatATAAATCCTTATATATAccTGCCCTTTGGAAcTGGACCCCGAAACTGCATTGGCtTGAGGTTcGCTaTcATGAACATGAAAcTTGCTGTcGTCAaAaTGCTGCAGAACTTCTCCTTCAgACCTTGTAAAGAAACACAGATcCCCCTGAAATTAGgCACTcAaGGAcTTaTTCAACCAcAgAAACCcATTGTTCTAAAgGTTGAGTCCAGAGaTGGGACCGTGAGTGGAGCCTGACTcTCCCTaAGGaCTTCcatTTTGtTgTTCAAGGAAGCTgTATCCCAGAACAccgagGACCTCagTTTACTTgGTGAATaAAATCCAGAATAAAGATTtTCTTAACCT

>CYP3A96_cow

ATGGAgCTaATCCCAAaCTTTTCcGTGGAAACcTGGGTTCTCCTGGCTAtCAGCCTGGtgCTCCTCTATCTATATGGaActTATtCACATGGACTgTTTAAGAAGCTGGGggTTCCtGGcCCAAgACCTCTgCCTcTTTTtGGAAacgTtCTatcCTACCGaAAGGGaTTTtTGAaGGTaaACAtCCTcTGTTGGtTATCACgGATCCAGACaTGATCAAAACaGTACTAGTGAAAGAATGTTATTCTGTCTTCACAAACCGGaGgTTTTGGTCCAaTGGGagTTATGAAAAATGCtgTtTCTgTggCTGAGGATGAACAATGGAAGAGAATACGgACaTTGCTGTCTCCAACCTTCACCAGTGGgAAGCTCAAGGAGATGTTCCCtATCATTGGgaAGTATGGAGATGTGTTGGTGAGGAACCTGAgGAAgGAAGCAGAGAAAGGCTTTGGGGCCTACAGCATGGATGTGATTACTAGcACATCATTTGGAGTGAAtATtGATTCCCTtggCAACCCACAAGATCCCTTTGTGGAgAATgCcAAGAAgCTCTTAAGATTTGATaTCCTTGATCCATTtcTaCTtTCAgTAgCAATTTTTCCATTcCTTAtcCCAaTcTTTGAAgTATTAAATATcagcaTATTTCCAAAaAGTGcTgtgaATTTTTTgACAAcATCcGTAAAAAaGATAAAAGAAAGTCGCCTCAAAGATActcAAACGtGTGGAcTTtCTTCAGCTGATGATTAACTCCCAgAAtTCCAAAGAAAcaGACAatCATAAAGCTCTcTCTGAcCaaGAaCTcaTgGCCCAgagTATTATcTTTATTTTTGgTGGCTATGAGaCCactAGCAcTTCTCTTTCcTTCaTTaTaTATgaaTTGGCCACTCACCCTGATGTCCAGCAGAAGCTGCAGGAGGAGATTGATGCgACTTTCCCCAATAaGGCgCCTCCgACCTATGATGtcCTGGcACAGATGGAGTATCTTGACATGGTGgTGAATGAGaCTCTCAGAaTgTTtCCtaTTGCTGtTAGAcTTGAgAGGttCTGTAAGAAgGATGTGGAAaTccaTGGGGTgTcCATTCCCAAAGGgACAacGGTGAcGGTGCCAatCTcTGtgCTTCACaGAGACcCAcAGCTCTGGCCAgAgCCTGAGGAGTTCCgTCCtGAAAGGTTCAGtAAGaAGAACAAGGACAGCATAAATCCTTAcgTcTAccTGCCtTTTGGAAcTGGACCCCGAAAtTGCATTGGCATGAGGTTTGCTaTcATGAACATGAAAcTTGCTGTTGTCAGAGTcCTGCAGAACTTCTCCTTCAAACCTTGTAAAGAAACACAGATcCCCtTGAAAaTAaaaAgTcAaGGAcTTtTaagACCgGAAAAACCcATTGTTCTgAAgGTTGtGctCAGAGaTGaGACCaTaAGTGGAGCtTGACTTcCCCTaAGGaCcTggGCTTTGtTCTTCAAGGAg

>CYP3A96_dog

ATGGACCTaATCCCAAGCTTTTCcaTGGAAACcTGGcTTCTCCTGGCTACCAGCCTGGtgCTCCTCTATCTgTATGGGAcCTAcACACATGGggTTTTTAAGAAGCTaGGAATTCCtGGaCCAAcACCTCTgCCTTTTgTGGGAACTgctCTGGGCTACCGTAAGGGTTTTATGATGGgCGACAGCCTGTGcTGGCTATCACAGATCCgGACaTGATCAAAACaGTgCTAGTGAAAGAATGTTATTCTGTCTTCACAAACCGGCGgTCTTTTGGTCCAGTGGGaTTTATGAAAAgTGCCATCTCTcTgTCTGAGGATGAAgAATGGAAGAGAATACGAACaTTGCTGTCcCCAACCTTCACCAGTGGAAAGCTCAAGGAGATGTTCCCCATCATTGGCCAGTATGGAGATGTGTTGGTGAGGAACCTGAgGAAgGAgGCAGAGAAAGGCAAatCCATcaaCTTGAAAGACATcTTTGGaGCCTACAGCATGGATGTGATTACcAGcACATCATTTGGAGTGAACATtGATTCCCTCAACAACCCACAAGATCCCTTTGTGGAAAATAtcAAGAAgCTCTTAAaATTTGATTTCCTTGATCCATTttTctTCTCAATAACAATTTTTCCATTcCTTAccCCAGTTTTTGAAgTATTAAATATcTggCTcTTTCCAAAaAGTGTTACTGATTTTTTCACAAAATCTGTAAAAAGaATgAAgGAAAaTCGCCTCAAAGATAAAcAAAAGCAACGAGTGGAcTTtCTTCAGCTGATGATTAACTCCCAgAAtTCtAAAGAAAcaGACACtCATAAAGCTCTaTCTGATtTGGAGCTgGTgGCCCAATCTATTATcTTTATTTTTGCTGGCTATGAGaCCactAGCAcTTCTCTTTCcTTCCTTaTGTATgaaTTGGCCACTCACCCTGATGTCCAGCAGAAaCTGCAGGAGGAGATTGATGCgACTTTCCCCAATAGCAgCaCCtACtTATGATaCTCTtGTACAGATGGAGTATCTTGACATGGTGTTGAATGAaagTCTCAGATTATaCCCAaTcaCTGGTAGAcTTGtAAGGgtCTGTAAGAAAGATGTGGAAaTcaGTGGtGTgTTCATTCCtAAAGGgACAGTGGTGATGGTGCCAaCCTTTaCTCTTCACCaAGACcCgGAtaTCTGGCCAgAgCCTGAGaAGTTCCAaCCtGAAAGGTTCAGtAAGaAGAACAAGGACAGCATAAATCCTTATAcATAccTGCCCTTTGGAAcTGGACCCCGAAACTGCcTTGGaATGAGaTTcGCaaTcATGAACATGAAAcTTGCccTcaTCAaAGTcCTGCAGAACTTCTCCTTCAAACCTTGTAAAGAAACACAGATTCCCCTGAAATTAagttCTcAaGGgTTaaTTCgACCAGAAgAACCcATTaTTCTcAAcGTTGAGcCaAGAGaTGGGAgtGTGcGTGGAGCCTGAaTTTCCCTaAGGaCTTCcctTTTGtTCTTCGAAGCTATATCCCAGAACAccAcAGACCTCaATTTcCTaTGTGAgTaAAAcCCAGAA

>CYP3A96_pig

ATGGACCTGATCCCAAGCTTTTCTGTGGAAACcTGGcTgCTtCTGGCTACCAGCCTGGtgCTCCTCTATCTATATGGGAcCTATtCACAcGGACTTTTTAAGAAGCTGGGgATTCCcGGGCCgAAACCTCTgCCTTaTTTtGGAAgTgTtCTaGaaTACCGTAAGGGTTTTATGAcGGTaGACAGCCTGTGcTGGCTATgAgAGAcCCAGAaaTGATCAAAgCaGTgCTtGTGAAAGAATGTTAcTCgGctTTCACAAAtCGGCGgTTTTGGTCCAGcGGGagTTATGAAAAATGCCATCTCTgTggCTaAGGATGAgCAATGGAAGcGAgTACGAACaTTGCTGTCTCCAACCTTCACCAGTGGAAAGCTCAAaGAGATGTTCCCCATCATTGcCCAGTATGGAGATaTGcTGGTGAGGAACCTGAgGAAgGAAGCAGAGAAAGGCAAGtCCATcATcTTTGGGGCCTACAGCATGGAcGTGATcACaAGcACAgCATTTGGAGTGAACTTTGATTTCCTcGATCCATTtATTCTCTCAATATTTCCATTcCTcAgcCCAtTcTTTGAAgTATTAgAcATcacTtTgTTTCCAAgaAGTtcTgtgaAaTTTTTCACAcAgTCTGTAAAAAGGATgAAAGAAAGTCGCCTCAAAGATcAAcAAAAGCAACGAGTGGAccTtCTTCAGCTGATGATTAACTCCCAgAAtTCCAAAGAAAcGGAtgCCCATAAAGCTCTGTCTGATCaaGAaCTTGTgGCCCAAagTATTATcTTcATTTTTGCcGGCTATGAGaCCactAGCAGTTCTCTcTCcTTCCTggcGTATaTacTGGCCACTCACCCTGAcGTCCAGCAGAAGCTGCAGGAGGAGATTGATGCgACcTTCCCCAgcAaGGC

>CYP3A96_mouse

ATGGACCTGATCCCAAaCTTTTCcaTGGAAACcTGGaTgCTCCTGGCTACCAGCCTGGtcCTtCTCTATagATATGGaActcATtCACATGGAaTTTTTAAaAAGtTGGGAATTCCtGGGCCAAAACCTCTgCCTTTcTTGGGgACgaTtCTtGctTACCagAAGGGTTgTATGAcGGTCGACAGCCTGTGcTGGCTATCACAGATCCAGACaTaATCAAAACaGTgCTgGTGAAgGAATGTTAcTCTacCTTCACAAACCGGCGgTTTGGTCCAGTGGGtaTTtTGAAAAAgGCCATCTCTATcTCTGAGaATGAAgAATGGAAGAGAATcCGAgCccTGCTGTCTCCAACCTTCACCAGTGGgAgGCTCAAGGAGATGTTCCCCATCATTaaCCAGTtTacAGATGTGTTGGTGAGaAACaTGAgGcAgGgAttAGgagAAGGaAAGCCCAccagCaTGAAAGACATcTTTGGGGCCTACAGCATGGATGTGATcACagccACcTCATTTGGAGTGAAtATtGATTCCCTCAACAACCCACAgGATCCtTTTGTGGAgAAaAtcAAGAAgCTCTTAAaATTTGATaTCtTTGATCCAcTgtTcCTCTCAgTgACAAaGAGTGGATTTtCTTCAGCTGATGATaAACTCCCAgAAtTaCAAAGGCACCTgCCACCTATGATaCcCTGcTACAGATGGAGTATCTaGACATGGTGgTGAATGAaaCTCTCAGATTATatCCAaTcGCTGGaAGAcTTGAgAGGgtCTGTAAGAcAGATGTtGAAaTcaaTGGGcTATTCATTCCCAAAGGgACtGTGGTGATGaTaCCAaCCTTTGCTCTTCACaaAGACcCgaAGtaCTGGCCAgAgCCTGAGGAaTTCCgcCCtGAAAGGTTCAGCAAGaAGAAtcAGGACAGCATcAATCCTTAcATgTAccTGCCCTTTGGgAgTGGACCaaGgAACTGCATTGGCATGAGGTTTGCTCTcATaAACATGAAAGTTGCTcTTGTCAGAGTcCTGCAGAACTTCaCtgTCcAgCCTTGTAAgGAAACtCAGATcCCttTaAAATTAagCAaacAaGGAcTTCTTCAACCAGAAAAcCCAcTccTTCTAAAAGTTGtGTCCAGAGaTGaGACtGTaAGT

>CYP3A96_sheep

ATGGAgCTaATCCCAAGCTTTTCcGTGGAAACcTGGGTTCTCCTGGCTACCgGCCTGGtgCTCCTCTATCTgGGaTTTtTGAaGGTaaACAaCCTcTGTTGGtTATCACAGATCCAGcCgTGATCAAAACaGTACTAGTGAAAGAATGTTATTCTGTCTTCACAAACCGGaGgTTTTGGTCCAtTGGGaaTTATGAAAAATGCCATCaCTgTggCTGAGGATGAACAtTGGAAGAGAATACGgACaTTGCTGTCTCCAACCTTCACCAGTGGgAAGCTCAAaGAGATGTTtCCtATCATTGGgaAGTATGGAGATGTGTTGGTGAGGAACCTGAgGAAgGAAGCAGAGAAAGGCAcGtCCgTcaaCaTGAAAGCATcTTTGGGGCCTACAGCATGGATGTGATTACTAGcACATCATTTGGAGTGAAtATtGATTCCCTCggCAACCCACAAGATCCaTTTGTGGAgAATgtccAGAAgCTCTTAAGATTTagTaTCCTTGATCCATTtcTTCTCTCAATAgtAcTcTTTCCATTcaTTAtcCCAaTccTTGAAgTATTAAATATcaccgTATTTCCAAAaAGTGcTctgaATTTTTTgACAAAATCTGTAAAAAGGATAAAAGAAAGTCGCCTCAAAGATAAtcAAAAGTGGAcTTtCTTCAGCTGATGATTAACTCCCAgAAtTCCAAAGAAAcaGACAaCCATAAAGCTCTcTCTGAcCaaGAaCTcaTAGCCCAAagTgTTATcTTTATTTTTGCcGGCTATGAGaCCactAGCAGTaCTCTTTCcTTCCTTCTGTATaTTTTGGCCACTCACCCTGATGTCCAGCAGAAGCTGCAGGAGGAGATTGATGCgACTTTCCCCAATAaGGCACCTCCaACCTAcGATGtcCTGGcACAGATGGAGTATCTTGACATGGTGgTGAATGAaaCTCTCAGATTgTTtCCAaTTGCTGtTAGAcTTGAtAGGttCTGTAAGAAgGATGTGGAAaTccaTGGGGTgTcCATTCCCAAAGGgACAacaGTGATGGTGCCAatCTccGtgCTTCACaGAGACcCAcAGCTCTGGCCAgAgCCTGAGGAGTTCCgTCCtGAAAGGTTCAGtAAGaAGAACAAGGACAGCATAAATCCTTAcgTcTAccTGCCtTTTGGAAcTGGACCCCGAAACTGCATTGGCATGAGGTTTGCcaTcATGAACATGAAAcTTGCTGTcGTCAGAGTcCTGCAGAACTTCTCCTTCAAACCTTGTAAAGAAACACAGg

>CYP3A96_elephant

ATGGACCTGATCCCAAGCTTTTCcaTGGAgACcTGGaTTCTtCTGGtcACCAtCCTGGtaCTCCTCTATagATATGGaAcCcATtCACATaatgTTcTaAAGAAGCTtGGgATTCCtGGGCCcAAACCTtTgCCTTTTTTGGGAACTtctCTGGcCTACCtcAAGGGTTTTATGATGGTCGACAGCCTGTGaTaGCcATCACAGATCCAGtCaTGATCAAgACaaTACTgGTGAAAGAATGTTATTCcacCTTCACAAAtCGGaGgTCTTTTGGTCCAaTGGGaTTTATGAAAtcTGCtATtTCctTgTCcGAGGATGAAgtgTGGAAGAGAgTACGAACaTTGCTGTCTCCAACtTTCACCAGTGGAAAaCTCAAGGAGATGTTCCCCATCATcaGCCAaTATGGtGATGTGTTGGTGAaGcAtCTGAgagAgGAAGaAcAGAAAGGCAAGCCtgTTGCgTTGAAACATcTTTGGaGCtTACAGCATGGATGTGATaACTAGcACgTCATTTGGAGTGAACATCGATTCCCTCcACAACCCACAAGATCCCTTTGTGcAAAAcgCcAgGAAgCTCaTAAagTTTGATTTCtTgGAcCCATTtgTTtTCTtAATAACAATTTTTCCATTcCTTAcTCCAaTTTgTGAAgcATTAAAcATcTcTCTgTTTCCAAgagacGcTACTGATTTTTTCACAAAATCTGTAcAAAGaATgAAAGAAAGcCGCCTtAAAGATAAtGAAAAGgtAaGAGTGGATTTgCTTCAGCTGATGATcgACTCCCAgAAtTCCAAAGAAATtatgtCCCATAAAGCTtTGaCTGATaTGGAGCTcGTAGCCCAATCaATTATcTTTATTTTTGCTGGtTATGAGaCCactAGCAcTaCTCTTTCcTTCCTTaTGTATtTaTTGGCCACcCACCCTGATaTtCAGCAGAAaCTGCAGaAGGAGATTGATGCggCTTTCCCCAATATGGCAtCTCCCACaTATGATGtcaTGtTgCAGATGGAaTATCTTGACATGGTGgTGAATGAaaCaCTCAGATTATTCCCAaTTGtTGGcAGAATTGAgAGGgtCTGcAAGAAAGATGTtGAAaTcaGTGGaGTgacCATTCCCAAAGGggCAGTGGcaATGGTGCCAgCCTTTGCTCTTCACCGAGACcCAGAaCaCTGGCCAgAgCCTGAGaAGTTCatTCCtGAgAGGTTCAGtAAGGAGAACAAGGACAGCATAgATCCTTActTATAccTGCCCTTTGGAAtTGGACCCCGAAAtTGCATcGGtATGAGGTTTGCTCTcATGAACATGAAAcTTGCTaTcaTCAGAGTtCTGCAGgAgTTCTCCgTCAAACCTTGTAAAGAAACACAggCAGAAgtACCtgTTGTTaTAAtgtTTGAGTCaAGAGacGGaAatGcaAGTGGAGCCTGACTTTCCCTagGGaaTTCTGCTTTGtTCTTCAAGGAAGtTgTtTtgCAGAtaAccAcAGACCTCtATT

>CYP3A96_human

ATGGAtCTcATCCCAAaCTTggCcGTGGAAACcTGGcTTCTCCTGGCTgtCAGCCTGataCTCCTCTATCTATATGGaAcCcgTACACATGGACTTTTTAAGAAGCTtGGAATTCCAGGGCCcAcACCTCTgCCTTTTTTGGGAAaTgcttTGtcCTtCCGTAAGTTTATGActGTCaACAGCCTaTGcTGGCTATCACAGATCCcGACaTGATCAAAACaGTgCTAGTGAAAGAATGTTATTCTGTCTTCACAAACCGGaGgTCTTTaGGcCCAGTGGGaTTTATGAAAAgTGCCATCTCTtTAgCTGAGGATGAAgAATGGAAGAGAATACGgtCaTTGCTGTCTCCAACCTTCACCAGcGGAAAaCTCAAGGAGATGTTCCCCATCATTGcCCAGTATGGAGATGTaTTGGTGAGaAACtTGAgGcggGAAGCAGAGAAAGGCAAGCCtgTcaCCTTGAAAGACATcTTTGGGGCCTACAGCATGGATGTGATTACTgGcACATCATTTGGAGTGAACATCGAcTCtCTCAACAAtCCACAAGAcCCCTTTGTGGAgAgcACtAAGAAgtTCcTAAaATTTGgTTTCtTaGATCCATTatTTCTCTCAATATCCATTcCTTAccCCAGTTTTTGAAgcATTAAATgTcTcTCTgTTTCCAAAagaTaccAtaaATTTTTTaAgtAAATCTGTAAAcAGaATgAAgaAAAGTCGCCTCAAcGACGAcTaGATTTCCTTCAGCTGATGATTgACTCCCAgAAtTCgAAAGAAActGAgtCCCAcAAAGCTCTGTCTGATCTGGAGCTcGcAGCCCAgTCaATaATcTTcATTTTTGCTGGCTATGAaaCCacCAGCAGTgtTCTTTCcTTCacTtTaTATgaacTGGCCACTCACCCTGATGTCCAGCAGAAaCTGCAaaAGGAGATTGATGCAgtTTTgCCCAATAGGCACCaCCtACCTATGATGCcgTGGTACAGATGGAGTAcCTTGACATGGTGgTGAATGAaaCaCTCAGATTATTCCCAGTTGCTatTAGAcTTGAgAGGACtTGcAAGAAAGATGTtGAAaTcaaTGGGGTATTCATTCCCAAAGGgtCAaTGGTGgTGaTtCCAaCtTaTGCTCTTCACCatGACcCAaAGtaCTGGaCAgAgCCTGAGGAGTTCCgcCCtGAAAGGTaCAaCAgtaAGAAgAAGGACAGCATAgATCCTTAcATATAcAcaCCCTTTGGAAcTGGACCCaGAAACTGCATTGGCATGAGGTTTGCTCTcATGAACATGAAAcTTGCTcTaaTCAGAGTcCTtCAGAACTTCTCCTTCAAACCTTGTAAAGAAACACAGATcCCCtTGAAATTAGaCACgcAaGGAcTTCTTCAACCAGAAAAACCcATTGTTCTAAAgGTgGAtTCaAGAGaTGGaACCcTaAGTGGAGaaTGAgTTattCTaAGGatTTCTaCTTTGGTCTTCAAGaAAGCTgTgcCCCAGAACAccAGAGAttTCa

>CYP3A97_horse

ATGGACCTGATCCCCAACTTTTCTACAGAAACCTGGGTTCTCCTGGCTACCAGCCTTGTGTTCCTCTATCTATTTGGCACCTATACACATGGACTTTTTAAGAAGCTCGGAATTCCTGGGCCGACTCCTCTGCCTTTCTTTGGAAATATTCTGAGCTACCGTAAGGGTATTTGGGATTTTGACAAGAAATGTTTTAAAAAGTATGGAAAAACGTGGGGGTTTTATGAAGGCCGGCTTCCTGTATTGGCTATCACAGATCCAGACATGATCAAAACCGTACTAGTGAAAGAATGTTATTCTGTCTTCACAAACCGGCGAACTTTTGGTCCAGAGGGATTTATGAAAAATGCCATCACTCGGTCTGAGGATGAACAATGGAAGAGAATACGAACTTTGCTGACGCCAACCTTCACCAGTGGAAAGCTCAAGGAGATGTTCCCCATCATTGGCCATTATGGAGATGTGTTGGTGAGGAACCTAAGGAATGAAGCAGAGAAAGGCAAACCCGTCACCTTGAAAAACATCTTTGGGGCCTACAGCATGGATGTGATTACTAGCACATCATTTGGAGTGAACATAGATTCCCTCAACAACCCACAAGATCCCTTTGTGGACAATGCTAAGAGGCTCTTAAGACTTGATTTTCTTGATCCACTCATTCTCTCAATAACTCTCTTTCCATTTCTTCGCCCAGTTTATGAAGCATTAAATATCAGTGTGTTTCCAAAAAGTGTAACTGATTTTTTCATAAAATCTGTAAAAAGGATGAAAGAAAGTCGCCTCAAAAATAAGGAAACGAACCGAGTGGATTTTCTTCAGTTGATGATTAACTCCCAGAATTCCAAAGAAATGGACACCCATAAAGCTCTGTCTGATCTCGAGCTTGTAGCCCAATCTATTGTGTTTATTTTTGCTGGCTATGAGACTACGAGCACTTCTCTCTCCTTCCTTATGTATCTTTTGGCCACTCACCCTGATGTCCAGCAGAAGCTGCAGAAGGAGATTGATGTGACTTTCCCCAATAAGGTACCTCCCACCTATGATGCCCTGCTACAGATGGACTATCTTGACATGGTGTTGAATGAATCTCTCAGATTATTCCCAGTTGCTGGTAGACTTCAGAGGATCTGTAAGAAAGATGTGGAACTCAATGGGGTGTTCATTCCCAAAAGGACACTGGTGACTGTGCCAACCTTTGTTCTTCACCGAGCCTCAGAGTTCTGGCCAGAGCCTGAAGAGTTTCGTCCTGAAAGGTTCAGTAAGGAGAACAAGGACAGCATAAATCCTTATATATACCTGCCTTTTGGAACCGGACCCCGAAACTGCATTGGCATGAGATTTGCTCTGATGAACATGAAACTTGCTGTCGTCAGAGTGCTGCAGAACTTCTCCTTCAAACCTTGTAAAGAAACAGAGATACCCATAAAATTAGGCACTGAAGCAATTGTGAAACCACAAAAGCCCATTGTTCTAAAAGTTGAGCCCAGAGATGGGACCGTGACTGGAGCCTGACTTTCCCTAAGGACTTCCACTGTTCTTCAAGGAAGCTGTATCCCAGAACACCAGAGATCTTAATTTACTTTGTGAATACAATTCAGAATGAAGGTGGGCTTAACCTACTGCATGTGATGGATGCCTGAGGATTCTTACATTTATTGATTTTCCAAGTGTCTATGTAGAGTATTACATGTTATGTGATATGAAGCAGGGGACAAGTGAGTGCCGGGTATGTGAACTCAGCTTGTCTGGTTCTTGCAGGATGATCTCCATCCTCCCACACTTAGTACTATCTACTTCTGCTGAACACTGATCGAGAATAAAATTTCTCAACAATTTTTAATGAACACAA

>CYP3A97_rhino

ATGGACCTGATCCCaAgCTTTTCcACAGAAACCTGGGTTCTCCTGGCTACCAGCCTgGTGcTCCTCTATCTgTaTGGgACCTATACtCATGGtCTTTTTAAGAAGCTgGGAATTCCTGGGCCGACaCCTCTGCCTTTtTTgGGAAATATTCTaAGCTACCaTAAGGGTgTTTGGGATTTTGACAAGAAATGTTTTAAAAAGTATGGAAAAAtGTGGGGGTTTTATGAtGGtaGaCggCCTGTgTTGGCTATCACAGATCCAGACATGATCAAAACaGTACTAGTGAAAGAATGTTATTCTGTCTTCACAAACCGGccCTTTTGGTCCAGtGGGATTTATGAAAAATGCCATCgCTCtGTCTacaGATGAACAATGGAAGAGAATACGAACaTTGCTGtCtCCAACCTTCACCAGTGGAAAGCTCAAGGAGATGTTCCCCATCATTGGCCgaTATGGAGATGTGTTGGTGAGGAACCTgAGaAAgGAAGCAGAGAAAGGCAggCCtGTCACtTTGAAAgCATCTTTGGGGCCTAtAGCATGGATGTGATTACTAGCACATCATTTGGAGTGAACgTcGATTCCCTCAACAACCCACAAGATCCCTTTGTGGAaAATaCcAAGAaGCTCTTAAaAtTTGATTTcCTcGATCCAtTCtTTCTCTCAATAACTCTTTCCATTTCTTaGCCCAGTTTtTGAAGtATTAAATATCttTcTcTTTCCAAAAAGTGTtACTGATTTTTTCAcAAAATCTGTAAAAAGGATGAAAGAAAGTCGCCTCAAAgATAAaGAAAaGAACCGAGTGGATTTTCTTCAGcTGATGATTAACTCCCAGAATTCCAAAGAAATGGACACCCATAAAGCTCTGTCTGATCTgGAGCTgGTgGCCCAATCTATTaTcTTTATTTTTGCTGGCTAcGAGACcACaAGCACTTCTCTtTCCTTCCTTATGTATCTcTTGGCtACTCACCCTGATGTCCAaCAGAAGtTGCAGgAGGAGATTGATGctACTTTCCCCAATAAGGtgCCaCCCACaTATGATaCCCTGtTACAGATGGAgTATCTTGACATGcTGTTGAATGAATCTCTCAGATTATTCCCAaTTGCTGGTAGACTTgAGAGGgTtTGTAAGAAcGATGTGGAAaTCAATGGaGTGTTCATTCCtAAAgGcACAgTaGTGAtgGTGCCAACaTTTaTTCTTCACCGAGCCTCAGAGTTCTGGCCAGAtCCTGAgGAGTTcCaTCCTGAAAGGTTCAGTAAGaAGAAtAAGGACAatATAAATCCTTATATATACCTGCCcTTTGGAACtGGACCCCGAAACTGCATTGGCtTGAGgTTcGCTaTcATGAACATGAAACTTGCTGTCGTCAaAaTGCTGCAGAACTTCTCCTTCAgACCTTGTAAAGAAACAcAGATcCCCcTgAAATTAGGCACTcAAGgAcTTaTtcAACCACAgAAaCCCATTGTTCTAAAgGTTGAGtCCAGAGATGGGACCGTGAgTGGAGCCTGACTcTCCCTAAGGACTTCCAtTGTTgTTCAAGGAAGCTGTATCCCAGAACACCgagGAcCTcAgTTTACTTgGTGAATAaAATcCAGAATaAAGaTtttCTTAACCTACTGCcctTGATGGATGCCTatGcAgTCTTACATTcATTGcaTTTCtcAGTGTCTgTGTAGAGTATTACgTGTTgTGTGATATaAAGgAGGGGcCttGTaAGTGCCaGaTATGTGgACTtAcCTTaTCTtGTTCTcaCAGaATGATCTCCATCCaCCCcCAgTTAGTACcATCTACTctccCTGAgCtCTGATCaAGAATAAAATTTCTCAACAATTTT

>CYP3A97_cow

ATGGAgCTaATCCCaAACTTTTCcgtgGAAACCTGGGTTCTCCTGGCTAtCAGCCTgGTGcTCCTCTATCTATaTGGaACtTATtCACATGGACTgTTTAAGAAGCTgGGggTTCCTGGcCCaAgaCCTCTGCCTcTtTTTGGAAAcgTTCTatcCTACCGaAAGTTTGAtgAagAATGTTTTAAAAAGTATGGgAAAAtGTGGGGGaTTTtTGAAGGtaaaCaTCCTcTgTTGGtTATCACgGATCCAGACATGATCAAAACaGTACTAGTGAAAGAATGTTATTCTGTCTTCACAAACCGGaGgTTTTGGTCCAatGGGAgTTATGAAAAATGCtgTttCTgtGgCTGAGGATGAACAATGGAAGAGAATACGgACaTTGCTGtCtCCAACCTTCACCAGTGGgAAGCTCAAGGAGATGTTCCCtATCATTGGgaAgTATGGAGATGTGTTGGTGAGGAACCTgAGGAAgGAAGCAGAGAAAGGCTCTTTGGGGCCTACAGCATGGATGTGATTACTAGCACATCATTTGGAGTGAAtATtGATTCCCTtggCAACCCACAAGATCCCTTTGTGGAgAATGCcAAGAaGCTCTTAAGAtTTGATaTcCTTGATCCAtTtcTaCTtTCAgTAggTCTCTTTCCATTcCTTatCCCAaTcTtTGAAGtATTAAATATCAGcaTaTTTCCAAAAAGTGctgtgaATTTTTTgAcAAcATCcGTAAAAAaGATaAAAGAAAGTCGCCTCAAAgATActcAAAaGgCGtGTGGAcTTTCTTCAGcTGATGATTAACTCCCAGAATTCCAAAGAAAcaGACAatCATAAAGCTCTcTCTGAcCaaGAaCTcaTgGCCCAgagTATTaTcTTTATTTTTGgTGGCTATGAGACcACtAGCACTTCTCTtTCCTTCaTTATaTATgaaTTGGCCACTCACCCTGATGTCCAGCAGAAGCTGCAGgAGGAGATTGATGcGACTTTCCCCAATAAGGTgCCTCCgACCTATGATGtCCTGgcACAGATGGAgTATCTTGACATGGTGgTGAATGAgaCTCTCAGAaTgTTtCCtaTTGCTGtTAGACTTgAGAGGtTCTGTAAGAAgGATGTGGAAaTCcATGGGGTGTcCATTCCCAAAgGGACAacGGTGACgGTGCCAAtCTcTGTgCTTCACaGAGaCcCAcAGcTCTGGCCAGAGCCTGAgGAGTTcCGTCCTGAAAGGTTCAGTAAGaAGAACAAGGACAGCATAAATCCTTAcgTcTACCTGCCTTTTGGAACtGGACCCCGAAAtTGCATTGGCATGAGgTTTGCTaTcATGAACATGAAACTTGCTGTtGTCAGAGTcCTGCAGAACTTCTCCTTCAAACCTTGTAAAGAAACAcAGgCCggAAAAaCCCATTGTTCTgAAgGTTGtGCtCAGAGATGaGACCaTaAgTGGAGCtTGACTTcCCCTAAGGACc

>CYP3A97_dog

TGGACCTaATCCCaAgCTTTTCcAtgGAAACCTGGcTTCTCCTGGCTACCAGCCTgGTGcTCCTCTATCTATTtGGgACCTAcACACATGGggTTTTTAAGAAGCTaGGAATTCCTGGaCCaACaCCTCTGCCTTTtgTgGGAAcTgcTCTGgGCTACCGTAAGTTTTGAtgAGAAcTGTTTTAggAAGTATGGAAgAAtGTGGGGGTTTTATGAtGGgCGaCagCCTGTgcTGGCTATCACAGATCCgGACATGATCAAAACaGTgCTAGTGAAAGAATGTTATTCTGTCTTCACAAACCGGCGgCTTTTGGTCCAGtGGGATTTATGAAAAgTGCCATCtCTCtGTCTGAGGATGAAgAATGGAAGAGAATACGAACaTTGCTGtCcCCAACCTTCACCAGTGGAAAGCTCAAGGAGATGTTCCCCATCATTGGCCAgTATGGAGATGTGTTGGTGAGGAACCTgAGGAAgGAgGCAGAGAAAGGCAAAtCCaTCAaCTTGAAAAgCATCTTTGGaGCCTACAGCATGGATGTGATTACcAGCACATCATTTGGAGTGAACATtGATTCCCTCAACAACCCACAAGATCCCTTTGTGGAaAATatcAAGAaGCTCTTAAaAtTTGATTTcCTTGATCCAtTttTctTCTCAATAACTCTgTTTCCATTcCTTacCCCAGTTTtTGAAGtATTAAATATCtGgcTcTTTCCAAAAAGTGTtACTGATTTTTTCAcAAAATCTGTAAAAAGaATGAAgGAAAaTCGCCTCAAAgATAAaCGAGTGGAcTTTCTTCAGcTGATGATTAACTCCCAGAATTCtAAAGAAAcaGACACtCATAAAGCTCTaTCTGATtTgGAGCTgGTgGCCCAATCTATTaTcTTTATTTTTGCTGGCTATGAGACcACtAGCACTTCTCTtTCCTTCCTTATGTATgaaTTGGCCACTCACCCTGATGTCCAGCAGAAaCTGCAGgAGGAGATTGATGcGACTTTCCCCAATAAGGTAttgCCCACtTAcGATGCCCTtgTgCAGATGGAaTATCTgGACATGGTGTTGAATGAAaCTCTCcGATTATaCCCAaTcGCTGGTAGACTTgAGAGGgTCTGTAAGAAAGATGTGGAAaTCAgTGGtGTGTTCATTCCCAAAgGGACAgTGGTGAtgGTGCCAACCTTTacTCTTCAtCGAGaCcagagtcTCTGGCCAGAGCCTGAgGAaTTcCGaCCTGAAAGGTTCAGTAgGaAGAACAAGGACAGCATAAATCCTTATAcATACCTGCCTTTTGGAACtGGACCCCGAAACTGCATTGGaATGAGgTTTGCgaTcATGAACATGAAACTTGCccTtGTtAGgGTcCTGCAGAACTTCTCCTTCAAACCTTGTAAAGAAACAcAGATcCCCcTgAAATTAaatgCTcAAGggATTaTtcAACCtgAAAAGCCCATTGTTCTcAAgGTTGAGCCaAGAGATGGGAgtGTaAaTGGAGCCTGACTTTCCCTAAGGACTTCCTCCCAGAACACCAGAGAcCTcAATTTcCTT

>CYP3A97_pig

ATGGACCTGATCCCaAgCTTTTCTgtgGAAACCTGGcTgCTtCTGGCTACCAGCCTgGTGcTCCTCTATCTATaTGGgACCTATtCACAcGGACTTTTTAAGAAGCTgGGgATTCCcGGGCCGAaaCCTCTGCCTTatTTTGGAAgTgTTCTagaaTACCGTAAGGGcATgTGGcATTTTGACAAtAAATGTTTTAAAAAGTATGGgAAAAtGTGGGGGTTTTATGAcGGtaGaCagCCTGTgcTGGCTATgAgAGAcCCAGAaATGATCAAAgCaGTgCTtGTGAAAGAATGTTAcTCgGctTTCACAAAtCGGCGgTTTTGGTCCAGcGGGAgTTATGAAAAATGCCATCtCTgtGgCTaAGGATGAgCAATGGAAGcGAgTACGAACaTTGCTGtCtCCAACCTTCACCAGTGGAAAGCTCAAaGAGATGTTCCCCATCATTGcCCAgTATGGAGATaTGcTGGTGAGGAACCTgAGGAAgGAAGCAGAGAAAGGCAAgtCCaTCAATCTTTGGGGCCTACAGCATGGAcGTGATcACaAGCACAgCATTTGGAGTGAACGATTTcCTcGATCCAtTtATTCTCTCAATAACTtTaTTTCCATTcCTcaGCCCAtTcTtTGAAGtATTAgAcATCAcTtTGTTTCCAAgAAGTtctgtgaAaTTTTTCAcAcAgTCTGTAAAAAGGATGAAAGAAAGTCGCCTCAAAgATcAacAAACCGAGTGGAccTTCTTCAGcTGATGATTAACTCCCAGAATTCCAAAGAAAcGGAtgCCCATAAAGCTCTGTCTGATCaaGAaCTTGTgGCCCAAagTATTaTcTTcATTTTTGCcGGCTATGAGACcACtAGCAgTTCTCTCTCCTTCCTggcGTATaTacTGGCCACTCACCCTGAcGTCCAGCAGAAGCTGCAGgAGGAGATTGATGcGACcTTCCCCAgcAAGG

>CYP3A97_elephant

ATGGACCTGATCCCaAgCcTTTCcACAGAgACtTGGaTTCTCCTGGCcACCAGCCTgGTGcTCCTCTAcCTATaTGGaACCTATtCACATaatgTTcTaAAGAAGCTCGGgATTCCTGGGCCcAagCCTCTGCCTTTtgTgGGAgcTcTTCTGgcCTACCGcAAGGGTtTgTGGGATTTTGACAtGAAATGTTcTAAAAAGTATGGAAAAAtaTGGGGGTTTTATcAtGGtCtaCagCCTGTgaTaGCcATCACAGATCCtGgCATGATCAAgACaaTAaTgGTGAAAGAATGTTATTCcacCTTCACAAACCGGaGgACTTTTGtTCCAatGGGATTTATGAAAtcTGCCATttCcttGTCTaAGGATGAAgAATGGAgGAGAgTACGAACgTTGCTGtCtCCAACCTTCACCAGTGGAAAaCTCAAGGAGATGcTCCCCATCATcGGCCAgTATGGAGAaGTaTTGtTGAaGcAtCTgAGagAgGAAGCAGAaAAAGGCAAgCCtGTCACaTTGAAgCATCTTcGGGGCtTACAGCATGGATGTGATcACaAGCACgTCATTTGGAGTGAACATcGATTCCCTCAACAACCCACAAGATCCCTTTGTGcAaAAcatcAgGAaGCTCaTgAGAtTTaATaTctTcGAcCCAtTgATTtTCaCAATAACTCTCTTTCCATTcCTTactCCAaTTctTGAAGCAcTAAgTATCtcTGTGTTTCCAAgAgcTGTtACTGATTTTTTtAcAAAATCTGTAAAAAcaATaAAAGAAAGcCGCCTtAAACGAGTGGAcTTTCTTCAGcTGATGATcgAtTCCCAGAATTCCAAgGAAActatgtCCCATAAAGCTtTGaCTGATaTgGAGCTcGTAGCCCAATCaATTaTcTTTATTTTTGCTGGtTATGAGACcACtAGCACTaCTCTtTCCTTCCTTATGTATtTaTTGGCCACcCACCCTGATaTtCAGCAGAAaCTGCAGAAGGAGATTGATGcGgCTTTCCCCAATAAGGTgCTCCCACaTATGATGtCaTGtTgCAGATGGAaTATCTTGACATGGTGgTGAATGAAaCaCTCAGATTATTCCCAaTTGtTGGcAGAaTTgAGAGGgTCTGcAAGAAAGATGTtGAAaTCAgTGGaGTGacCATTCCCAAAgGGgCAgTGGcaAtgGTGCCAgCCTTTGcTCTTCACCGAGaCcCAGAacaCTGGCCAGAGCCTGAgaAGTTcatTCCTGAAAAGGTgcAGGTTCAGTAAGGAGAACAAGGACAGCATAgATCCTTActTATACCTGCCcTTTGGAAttGGACCCCGAAAtTGCATcGGtATGAGgTTTGCTCTcATGAACATGAAACTTGCTaTCaTCAGAGTtCTGCAGgAgTTCTCCgTCAAACCTTGTAAAGAAACAcAGATcCCCATAAAAgTAGGCcaTGgAGCAATTaTtgcACCAgAAgtaCCtgTTGTTaTAAtgtTTGAGtCaAGAGAcGGaAatGcaAgTGGAGCCTGACTTTCCCTAgGGAtTGTTCTTCAAGGAAGtTGTtTtgCAGAtaACCAcAGAcCTctATTgACTcTGaGAA

>CYP3A97_human

AAACCTGGcTTCTCCTGGCTgtCAGCCTgGTGcTCCTCTATCTATTTGGaACCcATtCACATGGACTTTTTAAGAAGCTtGGAATTCCaGGGCCcACaCCTCTGCCTTTtTTgGGAAATATTtTGtcCTACCaTAAGGTTTTATGAtGGtCaaCagCCTGTgcTGGCTATCACAGATCCtGACATGATCAAAACaGTgCTAGTGAAAGAATGTTATTCTGTCTTCACAAACCGGaGgCTTTTGGTCCAGtGGGATTTATGAAAAgTGCCATCtCTatagCTGAGGATGAAgAATGGAAGAGAtTACGAtCaTTGCTGtCtCCAACCTTCACCAGTGGAAAaCTCAAGGAgATGgTCCCtATCATTGcCCAgTATGGAGATGTGTTGGTGAGaAAtCTgAGGcggGAAGCAGAGAcAGGCAAgCCtGTCACCTTGAAAAACATCTTTGGGGCCTACAGCATGGATGTGATcACTAGCACATCATTTGGAGTGAACATcGAcTCtCTCAACAAtCCACAAGAcCCCTTTGTGGAaAAcaCcAAGAaGCTtTTAAGAtTTGATTTTtTgGATCCAtTCtTTCTCTCAATACagTCTTTCCATTcCTcatCCCAaTTctTGAAGtATTAAATATCtGTGTGTTTCCAAgAgaaGTtACaaATTTTTTaAgAAAATCTGTAAAAAGGATGAAAGAAAGTCGCCTCCGAGTGGATTTcCTTCAGcTGATGATTgACTCtCAGAATTCaAAAGAAActGAgtCCCAcAAAGCTCTGTCcGATCTgGAGCTcGTgGCCCAATCaATTaTcTTTATTTTTGCTGGCTATGAaACcACGAGCAgTgtTCTCTCCTTCaTTATGTATgaacTGGCCACTCACCCTGATGTCCAGCAGAAaCTGCAGgAGGAaATTGATGcagtTTTaCCCAATAAGGTACCaCCCACCTATGATaCtgTGCTACAGtTGGAgTATCTTGACATGGTGgTGAATGAAaCaCTCAGATTATTCCCAGTTGCTatgAGACTTgAGAGGgTCTGcAAaAAAGATGTtGAAaTCAATGGGaTGTTtATTCCCAAAgGGgtggTGGTGAtgaTtCCAAgCTaTGTTCTTCAtCatGaCcCAaAGTaCTGGaCAGAGCCTGAgaAGTTcCtcCCTGAAAGGTTCAGTAAaaAGAACAAGGACAaCATAgATCCTTAcATATACacaCCcTTTGGAAgtGGACCCaGAAACTGCATTGGCATGAGgTTTGCTCTcgTGAACATGAAACTTGCTcTaGTCAGAGTcCTtCAGAACTTCTCCTTCAAACCTTGTAAAGAAACAcAGgCCAgAAAAaCCCATTGTTCTAAAgGTTGAGtCaAGgGATGGGACCGTaAgTGGAaCCTGAaTTTCCCTAAGGACTTCtTGcTCTTCAAaaAAGCTGTgaCCtAGAACACCAGAGAcCTcAAaTTACTTTGTGAAT

>CYP3A97_sheep

ATGGAgCTaATCCCaAgCTTTTCcctgGAAACCTGGGTTCTCCTGGCTACCgGCCTgGTGcTCCTCTATCTgTTTGGaACtTATtCACATGGACTgTTTAAGAAGCTgGGAgTTtCTGGGCCaAgaCCTCTGCCTTatTTTGGAAATgTTCTGtcCTACCGaAAGTTTGAtgAagAATGTTTTAAAAAGTATGGgAAAAtGTGGGGGaTTTtTGAAGGtaaaCaTCCTcTtTTGGtTATCACAGATCCAGACATGATCAAAACaGTACTAGTGAAAGAATGTTAcTCTGTCTTCACAAACCGGaGgACTTTTGGTCCAatGGGAaTTATGAAAAATGCtgTttCTgtGgCTGAGGATGAACAATGGAAGAGAATACGgACaTTGCTGtCtCCAACCTTCACCAGTGGgAAGCTCAAaGAGATGTTtCCtATCATTGGgaAgTATGGAGAcGTGTTGGTGAGGAACCTgAGGAAgGAAGCAGAGAAAGGCCATCTTTGGGGCCTACAGCATGGATGTGATTACTAGCACATCATTTGGAGTGAAtATtGATTCCCTtggCAACCCACAAGATCCCTTTGTGGAgAATGCcAAGAaGCTCTTAAGAtTTaATaTcCTcGATCCAtTtcTTCTCTCAATAgtaCTCTTTCCATTcCTTgtCCCAaTcTtTGAAGtATTAAATATCAccaTGTTTCCcAAAAGTGctgtgGATTTTTTgAcAAAATCTGTAAAAAGGATaAAAGAAAGTCGCCTCAAAgATAACGtGTGGAcTTTCTTCAGTTGATGATTAACTCtCAGAATTCCAAAGAAAcaGACAaCCATAAAGCTCTcTCTGAcCaaGAaCTcaTgGCCCAAagTgTTaTcTTTATTTTTGCcGGCTATGAGACcACtAGCAaTaCgCTtTCCTTCCTTtTGTATaTTTTGGCCACTCACCCTGATGTCCAGCAGAAGCTGCAGgAGGAGATTGATGcGACTTTCCCCAATAAGGTACCTCCgACCTAcGATGtCCTGgcACAGATGGAgTATCTTGACATGGTGgTGAATGAgaCTCTCAGAaTgTTtCCAaTTGCTGtTAGACTTgAtAGGcTCTGTAAGAAgGATGTGGAAaTCcAcGGGGTGTcCATTCCCAAAgGGACAgcGGTGACgGTGCCAAtCTTTGTgCTTCACaGAGaCcCAcAGcTCTGGCCAGAGCCTGAgGAGTTTCGTCCTGAAAGGTTCAGTAAGaAGAACAAGGACAGCATAAATCCTTATgTATACCTGCCTTTTGGAACtGGACCCCGAAAtTGCATTGGCATGAGgTTTGCcaTcATGAACATGAAACTTGCTaTtGTCAGAGTcCTGCAGAACTTCTCCTTCAAACCTTGTAAAGAAACAcAGgTTgATTTACTTTGTGgtaAaAcTcagaAATaAAGaTGGGCTTcAtCTAtTGCActTGcTGGATGaCTagGGt

>CYP3A97_mouse

ATGGACCTGATCCCaAACTTTTCcAtgGAAACCTGGaTgCTCCTGGCTACCAGCCTgGTccTtCTCTActTgTTTGGaACtcATtCACATGGAaTTTTTAAaAAGtTgGGAATTCCTGGGCCaAaaCCTCTGCCTTTCTTgGGgAcgATTCTtgctTACCagAAGGTTgTATGAcGGtCGaCagCCTGTgcTGGCTATCACAGATCCAGACATaATCAAAACaGTgCTgGTGAAgGAATGTTAcTCTacCTTCACAAACCGGCGgACTTTTGGTCCAGtGGGtaTTtTGAAAAAgGCCATCtCTatcTCTGAGaATGAAgAATGGAAGAGAATcCGAgCccTGCTGtCtCCAACCTTCACCAGTGGgAgGCTCAAGGAGATGTTCCCCATCATTaaCCAgTtTacAGATGTGTTGGTGAGaAACaTgAGGcAgGgAttAGgagAAGGaAAgCCCCATCTTTGGGGCCTACAGCATGGATGTGATcACagcCACcTCATTTGGAGTGAAtATtGATTCCCTCAACAACCCACAgGATCCtTTTGTGGAgAAaatcAAGAaGCTCTTAAaAtTTGATaTctTTGATCCACTgtTcCTCTCAgTgAaGAGTGGATTTTCTTCAGcTGATGATaAACTCCCAGAATTaCAAAGCatTaTCTGATgTgGAGaTTGTgGCtCAgTCagTTaTcTTTATTTTTGCTGGCTATGAGACcACaAGCAgTgCTCTtTCCTTtgcatTGTATtTgcTGGCCAtTCACCCTGATGTaCAGaAGAAaCTtCAGgAtGAaATTGATGcGgCTcTgCCCAATAAGGTACCTgCCACCTATGATaCCCTGCTACAGATGGAgTATCTaGACATGGTGgTGAATGAAaCTCTCAGATTATatCCAaTcGCTGGaAGACTTgAGAGGgTCTGTAAGAcAGATGTtGAAaTCAATGGGcTaTTCATTCCCAAAgGGACtgTGGTGAtgaTaCCAACCTTTGcTCTTCACaaAGaCcCgaAGTaCTGGCCAGAGCCTGAgGAaTTcCGcCCTGAAAGGTTCAGcAAGaAGAAtcAGGACAGCATcAATCCTTAcATgTACCTGCCcTTTGGgAgtGGACCaaGgAACTGCATTGGCATGAGgTTTGCTCTcATaAACATGAAAgTTGCTcTtGTCAGAGTcCTGCAGAACTTCaCtgTCcAgCCTTGTAAgGAAACtGAG
